# The Synthesis of Organic Molecules of Intrinsic Microporosity Designed to Frustrate Efficient Molecular Packing

**DOI:** 10.1002/chem.201504212

**Published:** 2016-01-11

**Authors:** Rupert G. D. Taylor, C. Grazia Bezzu, Mariolino Carta, Kadhum J. Msayib, Jonathan Walker, Rhys Short, Benson M. Kariuki, Neil B. McKeown

**Affiliations:** ^1^ EaStCHEM School of ChemistryUniversity of EdinburghDavid Brewster RoadEdinburghEH9 3FJUK; ^2^ School of ChemistryCardiff UniversityCardiffCF10 3ATUK

**Keywords:** adsorption, aromatic nucleophilic substitution, microporous materials, molecular packing, triptycene

## Abstract

Efficient reactions between fluorine‐functionalised biphenyl and terphenyl derivatives with catechol‐functionalised terminal groups provide a route to large, discrete organic molecules of intrinsic microporosity (OMIMs) that provide porous solids solely by their inefficient packing. By altering the size and substituent bulk of the terminal groups, a number of soluble compounds with apparent BET surface areas in excess of 600 m^2^ g^−1^ are produced. The efficiency of OMIM structural units for generating microporosity is in the order: propellane>triptycene>hexaphenylbenzene>spirobifluorene>naphthyl=phenyl. The introduction of bulky hydrocarbon substituents significantly enhances microporosity by further reducing packing efficiency. These results are consistent with findings from previously reported packing simulation studies. The introduction of methyl groups at the bridgehead position of triptycene units reduces intrinsic microporosity. This is presumably due to their internal position within the OMIM structure so that they occupy space, but unlike peripheral substituents they do not contribute to the generation of free volume by inefficient packing.

## Introduction

There is increasing interest in the study of microporous materials made using organic components,^1^ driven by potential applications in gas storage,^2^ selective gas separation membranes,^3^ heterogeneous catalysis,^4^ and nanoparticle encapsulation.^5^ Broadly, microporous materials can be subdivided into two structural classes: ordered materials, the porosity of which typically arises from their crystalline structure (for example, zeolites,^6^ metal–organic frameworks (MOFs),4a, 7 covalent organic frameworks (COFs),^8^ and porous molecular crystals^9^), and amorphous materials, the porosity of which arises from a disordered framework (for example, activated carbons,^10^ hypercrosslinked polymers,^11^ and other porous polymer networks^12^). The majority of both ordered and amorphous microporous materials are based on network structures, which are inherently insoluble. However, demand for solution‐processable porous materials has led to interest in non‐network polymeric and molecular materials. For example, microporosity can be generated from the inability of soluble polymer chains to pack together efficiently in the solid state, as demonstrated by the polymers of intrinsic microporosity (PIMs).^3^, ^12^, ^13^ Ordered porous molecular materials may be obtained from the self‐assembly of the component molecules into a crystalline porous packing arrangement,^9^, ^14^ or from the crystallisation of molecular cages, which act as prefabricated pores. For example, the groups of Cooper and Mastalerz have demonstrated that molecular cages, produced from multiple Schiff base condensations between simple amine and aldehyde precursors, can produce soluble materials with apparent BET surface areas (SA_BET_) approaching 1400 m^2^ g^−1^.^15^ Recently, Mastalerz et al. have created highly porous cages from triptycene‐based precursors that were suitably functionalised to undergo Schiff base condensations^16^ or boronic ester formation.^17^ Using the latter, a crystalline molecular material with a SA_BET_ of 3758 m^2^ g^−1^ was produced. The prefabricated pore structure of macrocycles^18^ and cages15c,15h, 19 can also generate microporosity from amorphous packing in the solid state. However, amorphous microporous materials derived from discrete organic molecules that are neither cages nor macrocycles are still relatively rare.^20^

**Figure 1:**
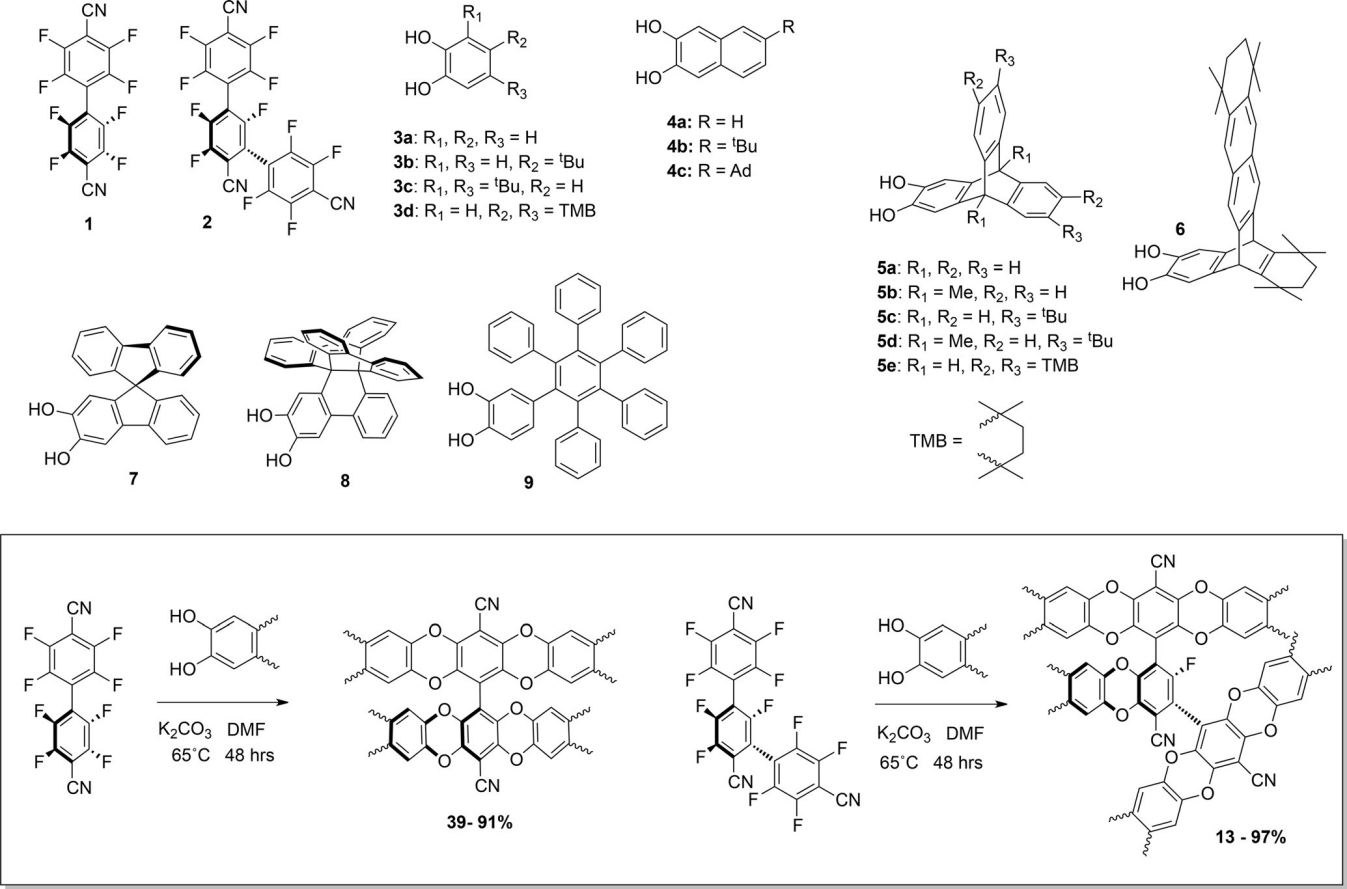
Precursors **1**–**9** (top) and general reaction scheme (box) for reaction of catechol terminal units with fluorinated cores **1** (left) and **2** (right).

**Figure 2:**
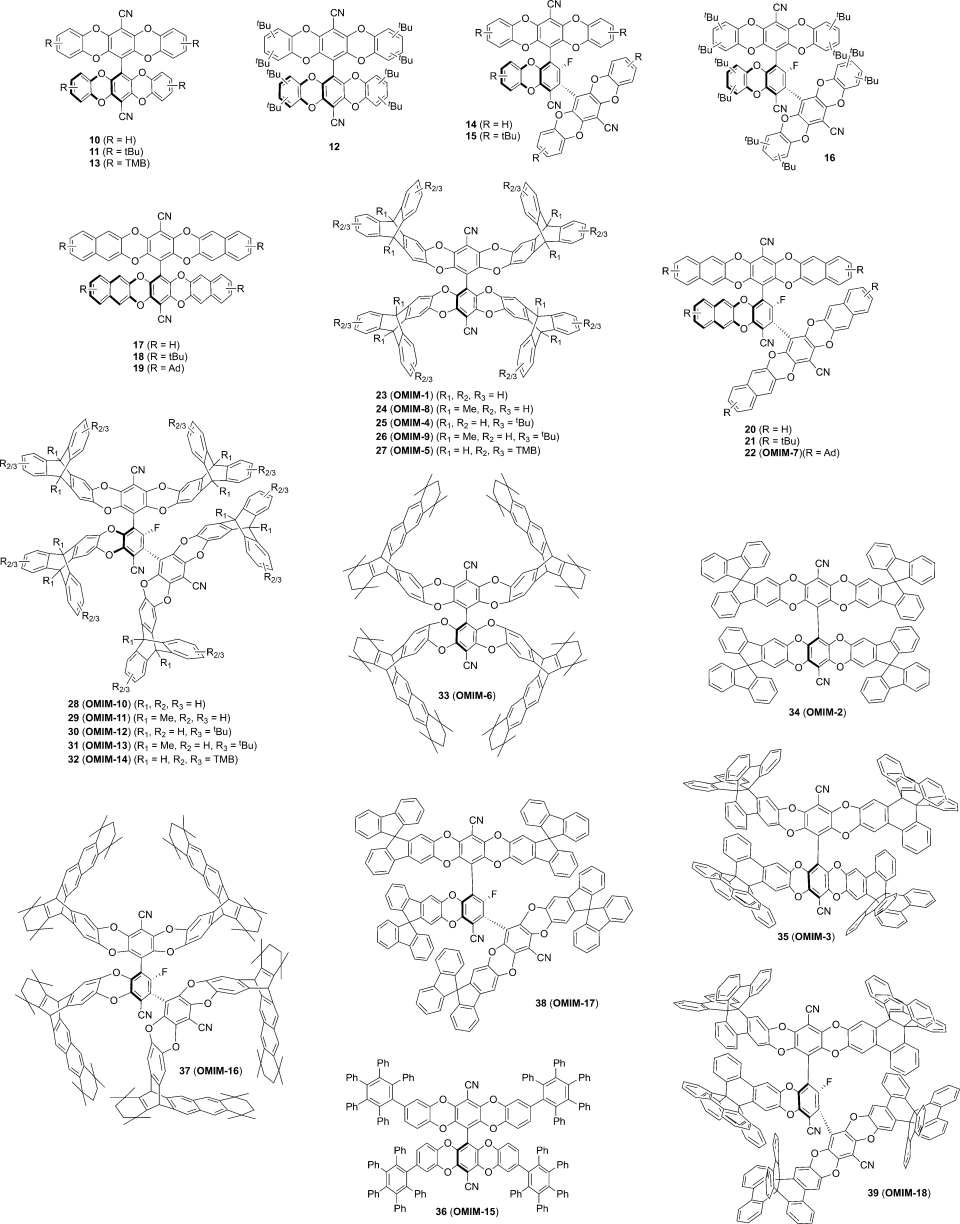
Structures of compounds **10**–**39**, including OMIM‐1 to OMIM‐18.

We are engaged in a joint modelling^21^ and synthesis20b programme to investigate organic molecules of intrinsic microporosity (OMIMs), which we define as discrete molecular compounds that are designed to generate microporous materials solely from their inability to pack efficiently in the solid state. Theory suggests that for both two‐22 and three‐dimensional^23^ objects, the most inefficient packing is produced when the constituent shapes possess highly concave faces. Accordingly, our design strategy is to combine rigid, aromatic precursors (Figure 1) to form large molecules with multiple concavities (Figure 2). Precursors are composed of core units (**1**, **2**) that possess *ortho*‐difluorine functionality and terminal units (**3**‐**9**) with catechol functionality, allowing for efficient combination by a double nucleophilic aromatic substitution to give dioxan fused units, a reaction previously utilised successfully in the synthesis of PIMs.^13^, ^24^ We selected 4,4′‐dicyano‐2,2′,3,3′,5,5′,6,6′‐octafluorobiphenyl (**1**)^25^ as an OMIM core owing to the two electron‐withdrawing nitrile groups, encouraging efficient dioxan formation. By serendipity, 2,2′,2′′,3,3′′,5,5′,5′′,6,6′,6′′‐undecafluoro‐[1,1′:3′,1′′‐terphenyl]‐4,4′,4′′‐tricarbonitrile (**2**) was obtained as a by‐product during the synthesis of **1**, and was investigated owing to its higher reactive functionality, allowing access to highly substituted materials. Catechol (**3**)‐ and naphthalene (**4**)‐based arms were selected as base‐line controls to examine the effect of increasing arm length and adding substituents. Triptycene (**5** and **6**)^26^, ^27^ and bulkier arm groups, based on spirobifluorene (**7**),^28^ propellane (**8**),^29^ and hexaphenylbenzene (**9**),^30^ were selected owing to their high internal free volume26b and proven porosity‐enhancing properties in PIMs and other microporous polymers.

## Results and Discussion

### Synthesis

The biphenyl fluorinated core precursor **1** was prepared in moderate yield (34 %) by the hexaethylphosphorous triamide mediated coupling of pentafluorobenzonitrile by adapting a procedure described in a patent.^25^ The terphenyl core precursor **2** was isolated as an unexpected by‐product in low yield (6 %) but in sufficient quantity to facilitate its use as a core for adduct synthesis. Each adduct was prepared by mixing the core precursor **1** or **2** with an excess of an arm unit chosen from **3**–**9** (Figure 1) in anhydrous DMF at 65 °C for 48 h in the presence of potassium carbonate. Each adduct (Figure 2) was purified either by column chromatography or by repeated trituration to remove more soluble impurities. Owing to the lack of symmetry of many of the substituted precursors, several of the reported OMIMs/adducts were isolated as mixtures of inseparable regioisomers (Table 1). The molecular mass, homogeneity and discrete nature of each adduct was confirmed by matrix‐assisted laser desorption ionisation mass spectrometry (MALDI‐MS) and gel permeation chromatography (GPC). For each adduct, the polydispersity index obtained from GPC was less than 1.1.

**Table 1:** OMIM/adduct data for compounds **10**–**39**.

Compound number	OMIM number^[a]^	Precursors	Apparent BET surface area [m^2^ g^−1^]^[b]^	Pore volume [cm^3^ g^−1^]	Yield [%]	Isomerically pure (Y/N)	CCDC number
**10** ^[c]^	–	**1**+**3 a**	7	0.03	74	Y	1406071
**11** ^[c]^	–	**1**+**3 b**	41	0.15	80	N	–
**12** ^[c]^	–	**1**+**3 c**	67	0.23	70	N	–
**13**	–	**1**+**3 d**	51	0.13	90	Y	–
**14**	–	**2**+**3 a**	13	0.05	87	Y	1406073
**15**	–	**2**+**3 b**	7	0.01	64	N	–
**16**	–	**2**+**3 c**	102	0.37	88	N	–
**17** ^[c]^	–	**1**+**4 a**	25	0.05	44	Y	1406072
**18** ^[c]^	–	**1**+**4 b**	260	0.33	64	N	–
**19** ^[c]^	–	**1**+**4 c**	132	0.25	39	N	–
**20**	–	**2**+**4 a**	29	0.11	13	Y	–
**21**	–	**2**+**4 b**	259	0.35	43	N	–
**22**	OMIM‐7	**2**+**4 c**	347	0.41	97	N	–
**23** ^[c,d,e]^	OMIM‐1	**1**+**5 a**	485	0.40	73	Y	955894
**24**	OMIM‐8	**1**+**5 b**	462	0.33	51	Y	1406070
**25** ^[d,e]^	OMIM‐4	**1**+**5 c**	654	0.54	91	N	–
**26**	OMIM‐9	**1**+**5 d**	599	0.42	47	N	–
**27** ^[e]^	OMIM‐5	**1**+**5 e**	702	0.60	83	Y	973327
**28**	OMIM‐10	**2**+**5 a**	423	0.44	30	Y	–
**29**	OMIM‐11	**2**+**5 b**	351	0.30	79	Y	–
**30**	OMIM‐12	**2**+**5 c**	726	0.72	81	N	–
**31**	OMIM‐13	**2**+**5 d**	651	0.47	65	N	–
**32**	OMIM‐14	**2**+**5 e**	698	0.44	77	Y	–
**33** ^[e]^	OMIM‐6	**1**+**6**	622	0.64	86	N	–
**34** ^[d]^	OMIM‐2	**1**+**7**	333	0.28	90	N	–
**35** ^[d]^	OMIM‐3	**1**+**8**	595	0.45	56	N	–
**36**	OMIM‐15	**1**+**9**	407	0.46	73	N	–
**37**	OMIM‐16	**2**+**6**	591	0.49	78	N	–
**38**	OMIM‐17	**2**+**7**	471	0.36	88	N	–
**39**	OMIM‐18	**2**+**8**	612	0.49	84	N	–

[a] We only classify those materials with an apparent BET surface area of more than 300 m^2^ g^−1^ as OMIMs. [b] Measured experimentally at 77 K following degassing at 135 °C under vacuum for 15 h. [c] Structure previously reported for packing simulation.21b [d] Structure previously reported for packing simulation.21a, 21c [e] Structure and experimental data previously reported.20b

**Figure 3:**
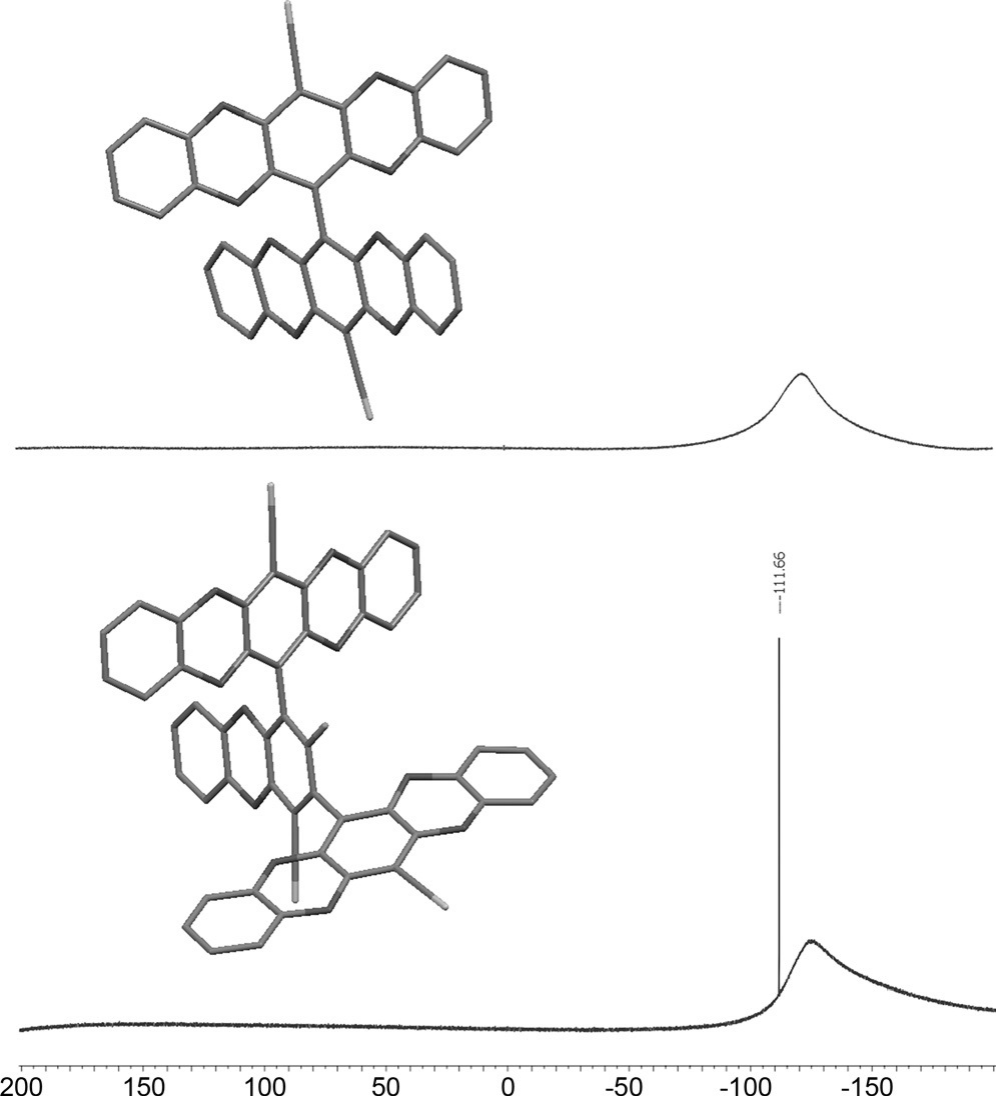
Molecular structures of **10** (top) and **14** (bottom) obtained from XRD analysis with their respective ^19^F NMR spectra, indicating total fluorine substitution in **10** and the retention of a single fluorine atom on the middle phenyl ring of the terphenyl unit in **14**.

The potential of **1** and **2** as OMIM cores was first tested by their reaction with excess **3 a** to ascertain their reactivity towards aromatic nucleophilic substitution. After purification, crystals of the resultant adducts (**10** and **14**) were achieved by slow diffusion of methanol into chloroform solutions. X‐ray crystallography coupled with ^19^F NMR spectroscopy (Figure 3) revealed these adducts to be the tetra‐substituted adduct (**10**), with a near orthogonal relationship between the two long struts of the molecule, and the penta‐substituted adduct (**14**), with one residual fluorine atom on the central aryl ring. This substitution pattern was found consistently with all catechol adducts of **1** and **2** in this study. Bridged products, in which a single arm bridges two cores (Supporting Information, Figure S1) were often found as trace impurities; however, these were readily removed by column chromatography. Detailed synthetic procedures and spectroscopic data for all novel precursors and adducts are given in the Supporting Information.

## Discussion

The simplest adduct (**10**) was found to be effectively non‐microporous, with an apparent BET surface area (SA_BET_) of 7 m^2^ g^−1^ as measured by nitrogen sorption at 77 K and it was highly insoluble, suggesting that the constituent molecules are able to pack together in an efficient manner, despite its awkward displaced cruciform structure. As predicted by molecular modelling,21b the introduction of bulky *tert*‐butyl groups to the periphery of the adducts (**11**, **12**) improved apparent microporosity (SA_BET_=41 and 67 m^2^ g^−1^, respectively). Both adducts were isolated as mixtures of regioisomers and were found to be highly soluble in common solvents (for example dichloromethane and THF), which combined with the increased porosity, suggests a disruption of the cohesive interactions between the constituent molecules. To further study this effect, 2,3‐dihydroxy‐5,5,8,8‐tetramethyl‐6,6,7,7‐tetrahydronapthalene (**3 d**) was prepared and combined with **1** to give **13**, which demonstrates comparable properties to **12**. Simple adducts of core **2** were also prepared using three catechol precursors **3 a–c**. Much like its biphenyl analogue, terphenyl‐based adduct **14** was poorly soluble in common solvents and possesses a negligible apparent SA_BET_ of 13 m^2^ g^−1^. However, the addition of two *tert*‐butyl groups per arm gave a highly soluble material (**16**) with a greater apparent SA_BET_ of 102 m^2^ g^−1^ over its biphenyl analogue (**12**, 67 m^2^ g^−1^), suggesting that the higher functionality of the terphenyl core generates a more porous material.

**Figure 4:**
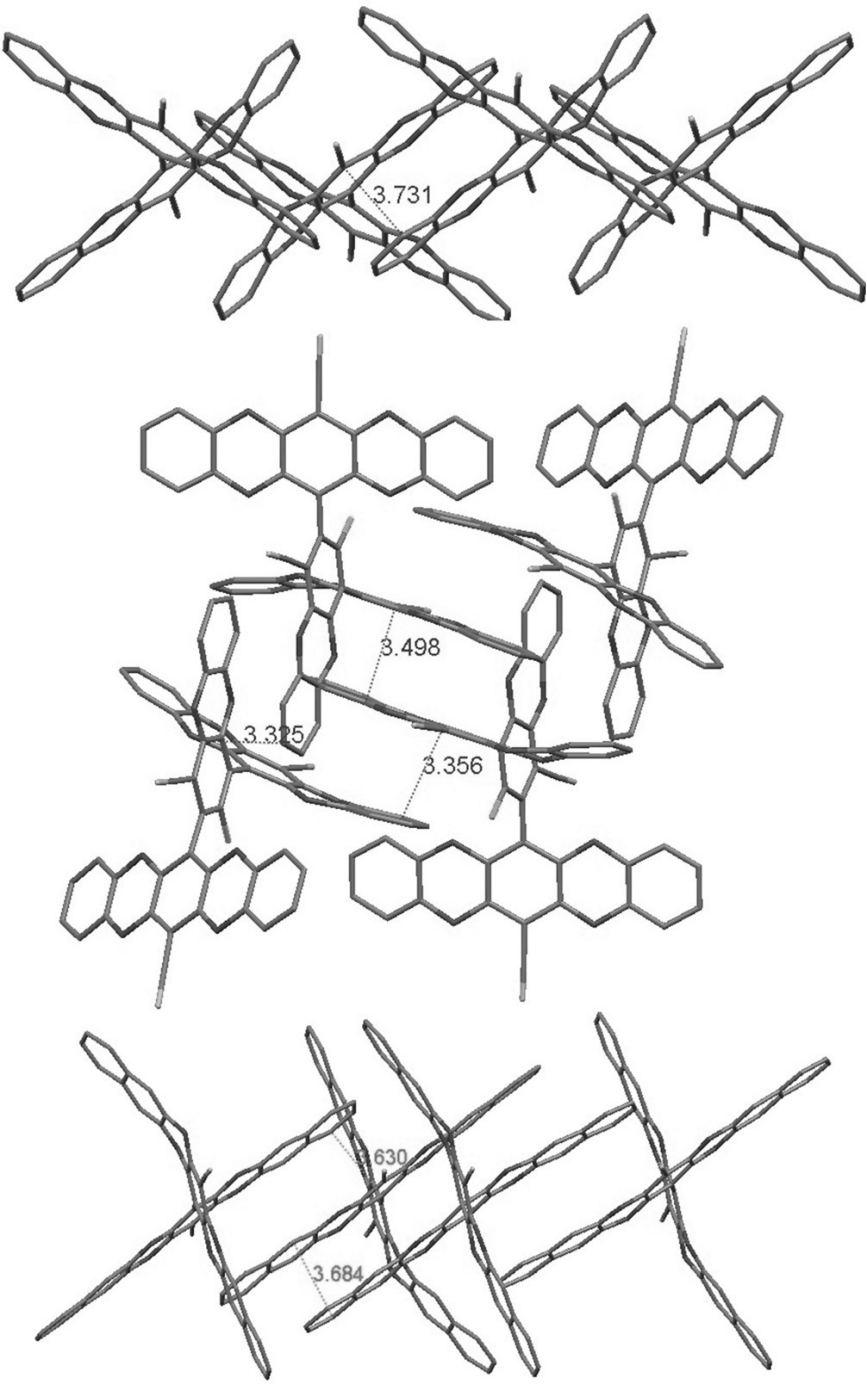
X‐ray crystal structures of **10** (top), **14** (middle), and **17** (bottom), revealing π–π induced staking of the constituent molecules. Protons and solvent molecules are removed for clarity.

Adducts **17** and **20**, derived from the reaction between unsubstituted naphthalene‐2,3‐diol precursor (**4 a**) and cores **1** and **2**, respectively, gave poorly soluble materials with no apparent microporosity (Table 1), similar to adducts **10** and **14** prepared using unsubstituted catechol (**3 a**). X‐ray crystallographic analysis of **10**, **14**, and **17** (Figure 4) demonstrates that for each of these adducts there are extensive π–π interactions between the aromatic arms causing the molecules to pack efficiently, resulting in poor solubility and negligible porosity. However, the use of *tert*‐butyl substituted naphthalene‐2,3‐diol (**4 b**) as a precursor gave biphenyl **18** and terphenyl **21** with significant apparent SA_BET_ of 260 and 259 m^2^ g^−1^, respectively. This enhanced porosity is attributed to the *tert*‐butyl substituents prohibiting close interactions between aromatic arms of adducts resulting in less efficient packing.

Given its success as a component for making organic microporous materials,^26^ triptycene derived arms were also investigated for making OMIMs. Hence, cores **1** and **2** were combined with triptycene‐2,3‐diol (**5 a**)^27^ to give **23** (OMIM‐1)20b and **28** (OMIM‐10), respectively. Both OMIM‐1 and OMIM‐10 proved to be highly soluble and gave amorphous solids with apparent SA_BET_ of 485 and 423 m^2^ g^−1^, respectively. The addition of bulky terminal substituents to give triptycene precursors **5 c** and **5 e** enhances porosity in the resulting OMIMs, irrespective of whether they are attached to a biphenyl (OMIM‐4 and OMIM‐5) or terphenyl core (OMIM‐12 and OMIM‐14), with each demonstrating a high apparent SA_BET_ in the range 654–726 m^2^ g^−1^. OMIM‐12 possesses intrinsic microporosity greater than many PIMs and comparable to that of the archetypal PIM‐1 (ca. 780 m^2^ g^−1^).13a OMIM‐12 is of a similar porosity to a π‐extended triptycene recently reported by the Mastalerz group (SA_BET_=754 m^2^ g^−1^),20c which were similarly designed to pack inefficiently.

**Figure 5:**
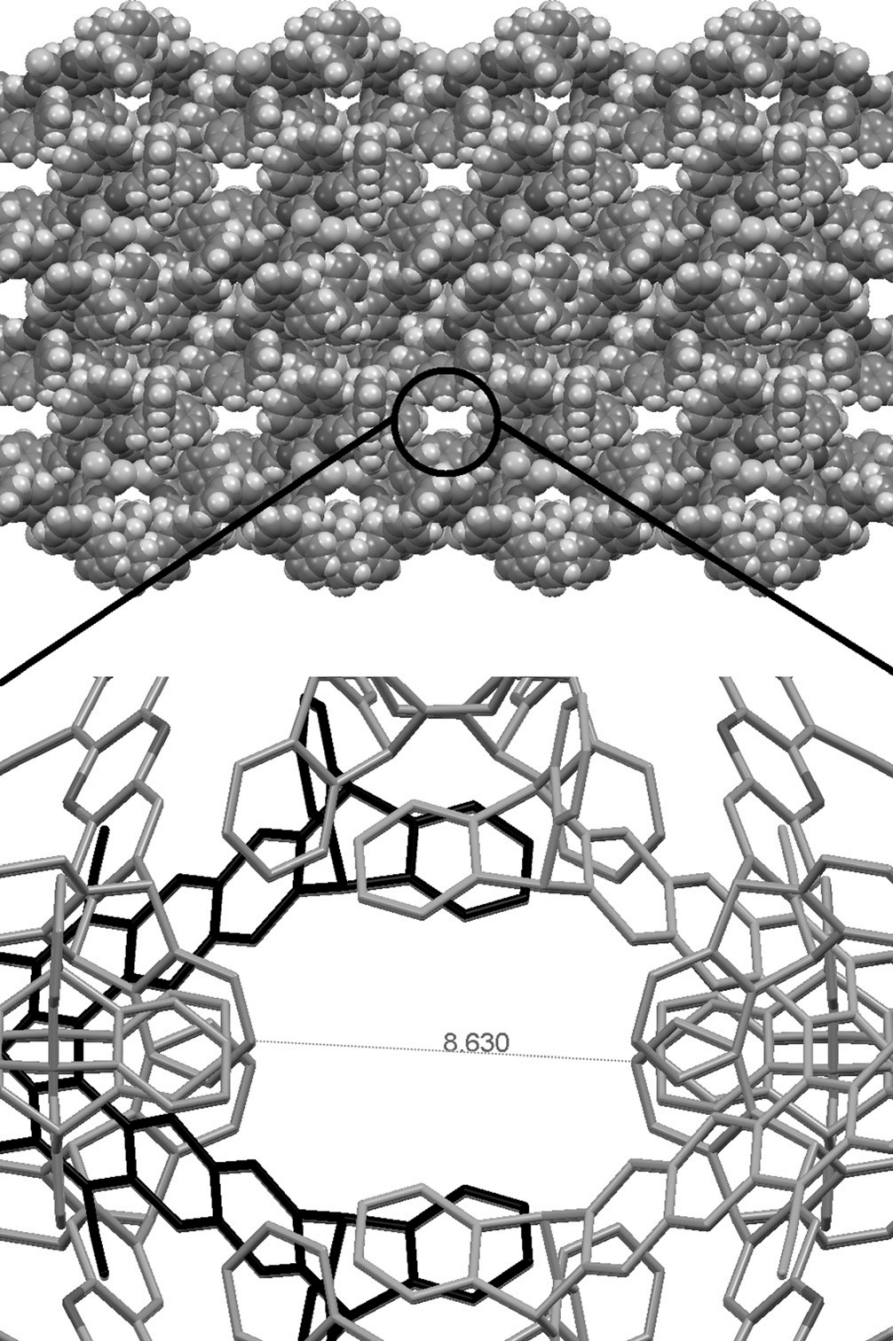
Space filling view (top) and expanded view (bottom) of channels running through OMIM‐**1**.

Notably, triptycene peripheral units substituted with methyl groups at their bridgehead positions (**5 b, d**) gave OMIMs with lower apparent values of SA_BET_ relative to their non‐methyl containing counterparts. For example, OMIM‐11 has an apparent SA_BET_ of 351 m^2^ g^−1^, as compared to 423 m^2^ g^−1^ for OMIM‐10. The values of SA_BET_ for the *tert*‐butyl substituted analogues (OMIM‐12 and OMIM‐13) also differ by a similar amount (75 m^2^ g^−1^). It appears that the space adjacent to the bridgehead in triptycene terminated OMIMs is directly contributing to the porosity of the material, hence, filling this space with a methyl group reduces the amount of intrinsic microporosity that can be generated during the amorphous packing of the molecules. Indirect evidence for this feature of amorphous packing comes from the single‐crystal XRD analysis of OMIM‐1 (Figure 5), which shows solvent‐filled channels defined partially by the triptycene bridgehead positions. Similar local ordering may occur within the amorphous packing of triptycene‐based OMIMs. Further XRD analysis of OMIM‐1, OMIM‐5, and OMIM‐8 is presented in the Supporting Information, Figure S2–S4.

An investigation to determine the suitability of various rigid structural building units as peripheral arms for OMIM construction was performed by combining catechol derivatives of benzenopentacene (**6**)20b spirobifluorene (**7**),^28^ propellane (**8**),^29^ and hexaphenylbenzene (**9**)^30^ with cores **1** and **2**. Benzenopentacene containing OMIM‐6 and OMIM‐16, were found to possess apparent SA_BET_ in the range 10–15 % lower than their more symmetrical isomeric triptycene‐based counterparts OMIM‐5 and OMIM‐14. It is probable that this is due to greater intermolecular interactions between the long struts of the benzenopentacene arms as compared to those of triptycene. From an OMIM comparison, it can be concluded that the efficiency of unsubstituted structural units for generating microporosity is in the following order: propellane>triptycene>hexaphenylbenzene>spirobifluorene>naphthyl=phenyl. This is consistent with findings from previously reported packing simulation studies.^21^

## Conclusions

Molecular adducts, designed to possess well‐defined concavities, were synthesised using the reaction between fluorinated biphenyl and terphenyl cores and peripheral arms chosen from a diverse range of rigid structural units. The intrinsic microporosity generated by the inefficient packing of these adducts was evaluated using nitrogen sorption at 77 K. The use of small, planar, non‐substituted arms gave insoluble materials with negligible surface areas (<30 m^2^ g^−1^). However, by using arms composed of bulky rigid structural units, OMIMs were prepared with apparent SA_BET_ in the range 333–612 m^2^ g^−1^. The efficiency of unsubstituted structural units for generating microporosity is in the following order: propellane>triptycene>hexaphenylbenzene>spirobifluorene>naphthyl=phenyl. Substitution of these arms with bulky groups further enhanced microporosity (up to 726 m^2^ g^−1^), which is presumably due to reducing intermolecular cohesive interactions. In contrast, the introduction of methyl groups at the bridgehead position of triptycene units reduced intrinsic microporosity. In this case, the internal position of the methyl groups within the OMIM structure means that they occupy space but, unlike peripheral substituents, cannot contribute to the generation of free volume by frustrating packing.

## Experimental Section

### Example synthesis of adduct 10

4,4′‐Dicyano‐2,2′,3,3′,5,5′,6,6′‐octafluorobiphenyl (**1**; 0.230 g, 0.661 mmol) and catechol (**3 a**) were added to an oven‐dried flask and purged with nitrogen. Anhydrous DMF (10 mL) was then added via syringe and the reaction mixture heated to form a solution, at which point, oven dried potassium carbonate (0.306 g, 2.78 mmol) was quickly added, the reaction sealed under nitrogen flow, heated to 65 °C and left to stir for 48 h. After cooling to room temperature, the reaction mixture was poured into water (200 mL), acidified with 2 m HCl, and allowed to stir as a suspension for 2 h. The crude product was then collected by filtration, washed with water (200 mL) and methanol (200 mL), then dried under suction. Purification of the crude material was achieved by column chromatography (dichloromethane/hexane, 1:1, *R*_f_=0.4) to give **10** (0.307 g, 74 %) as a yellow powder (m.p.>300 °C); IR (CH_2_Cl_2_ film) 2234, 1495, 1440, 1309, 1272, 1253 cm^−1^; ^1^H NMR (500 MHz, CDCl_3_): *δ*=7.00 (d, *J*=8.0 Hz, 4 H, Ar*H*), 6.96–6.93 (m, 4 H, Ar*H*), 6.88–6.85 (m, 4 H, Ar*H*), 6.66 ppm (d, *J*=8.0 Hz, 4 H, Ar*H*); ^13^C NMR (125 MHz, CDCl_3_): *δ*=141.1, 140.8, 140.0, 136.2, 125.3, 125.2, 117.0, 114.4, 110.6, 91.7 ppm (one carbon missing); HRMS (EI^+^, *m*/*z*) calc. for C_38_H_16_N_2_O_8_: 628.0907 [*M* ^+^], found 628.0903; GPC analysis (CHCl_3_) *M_n_*=548, *M*_w_=578 g mol^−1^ relative to polystyrene, *M*_w_/*M*_n_=1.055; BET surface area=7 m^2^ g^−1^; total pore volume=0.03 cm^3^ g^−1^ at *p*/*p* ^o^=0.98. Crystallography data (CHCl_3_/MeOH): triclinic, space group=*P* $\bar{1}$ , *a*=11.0410(6) Å, *b*=11.3324(7) Å, *c*=14.2766(7) Å, *α*=113.045(5), *β*=90.659(4), *γ*=106.663(5), *V*=1559.14 Å^3^, *Z*=2, *R_1_*=4.01.

Full experimental details and spectroscopic data for all of the new compounds are given in the Supporting Information. CCDC 955894, 973327, 1406070, 1406071, 1406072, 1406073, and 1406074 contain the supplementary crystallographic data for this paper. These data are provided free of charge by The Cambridge Crystallographic Data Centre.

## Supporting information

As a service to our authors and readers, this journal provides supporting information supplied by the authors. Such materials are peer reviewed and may be re‐organized for online delivery, but are not copy‐edited or typeset. Technical support issues arising from supporting information (other than missing files) should be addressed to the authors.

Supplementary
