# Supplementary material for: The Synthesis of Organic Molecules of Intrinsic Microporosity Designed to Frustrate Efficient Molecular Packing
Source: Chemistry. 2016 Jan 11;22(7):2466–72. doi: 10.1002/chem.201504212 (PMC4755154; doi:10.1002/chem.201504212)
Supplement: Supplementary file 1 — Supplementary [file CHEM-22-2466-s001.pdf]

CHEMISTRY
A European Journal

Supporting Information

The Synthesis of Organic Molecules of Intrinsic Microporosity Designed to Frustrate Efficient Molecular Packing

Rupert G. D. Taylor,[b] C. Grazia Bezzu,[a] Mariolino Carta,[a] Kadhum J. Msayib,[a] Jonathan Walker,[b] Rhys Short,[b] Benson M. Kariuki,[b] and Neil B. McKeown*[a]

chem_201504212_sm_miscellaneous_information.pdf

Contents

General Remarks 2
Experimental 3
Precursors 3
Biphenyl adducts/OMIMs 12
Terphenyl adducts/OMIMs 18
Full adsorption/desorption Isotherms 29
Biphenyl adducts/OMIMs 29
Terphenyl aducts/OMIMs 31
NMR Spectra 33
Mass Spectrometry Data 80
References 94

General Methods

Commercially available reagents (**3a**-**c**, **4**) were purchased from Sigma-Aldrich and used without further purification. Unless otherwise stated all reactions were carried out under an inert atmosphere of dry nitrogen gas (passed over anhydrous copper sulfate crystals). Oven-dried glassware was left in a 100°C oven for at least 2 hrs and cooled under dry nitrogen flow prior to use. Anhydrous solvents were obtained *via* distillation over calcium hydride (CH_2Cl_2), drying over well activated molecular sieves (THF), purchased from Sigma-Aldrich (DMF) or from a solvent purification system (diethyl ether, toluene). Column chromatography was performed over a silica gel (pore size 60 Å, particle size 40 – 63 µm) stationary phase.

Melting points were recorded using a Gallenkamp melting point apparatus and are uncorrected. Infrared adsorption spectra were recorded in the range 4000 – 400 cm^{-1} using a Perkin-Elmer 660 plus FTIR instrument, either as a thin film or nujol mull, between sodium chloride plates. ^{1}H NMR spectra were recorded in a suitable deuterated solvent using an Avance Bruker DPX 400 instrument (400 MHz) or an Avance Bruker DPX 500 instrument (500 MHz) or Avance Bruker III HD (500 MHz) equipped with Prodigy crio-probe or Avance Bruker III 500 (500 MHz). ^{13}C NMR spectra were recorded on the same machines at 100 MHz or 125 MHz respectively. ^{13}C-^{19}F coupling was observed in fluorinated compounds, but not assigned, due to the large coupling constants leading to ambiguity between coupled and discrete peaks. ^{19}F NMR spectra were recorded on a Jeol JNM-ECP 300 instrument (300 MHz) at 282 MHz or Avance Bruker III HD (471 MHz) equipped with Prodigy crio-probe. Chemical shifts (δ) were recorded in parts per million (ppm) and corrected according to standard solvent peaks.[1] Multiplicity is reported as singlet (s), doublet (d), doubled-doublet (dd), doubled-triplet (dt), triplet (t), quartet (q), pentet (p) or multiplet (m). Broad or complex peaks are further labelled 'br' or 'complex' respectively. Coupling constants (J) are quoted in Hz. Small molecule (Mw < 1000 g mol^{-1}) low-resolution mass spectrometric (LRMS) and high resolution mass spectrometric (HRMS) data were obtained using a Waters GCT Premier E1 instrument, utilising either electron impact (EI) or electrospray (ES) ionisation. Large molecule (Mw ≥ 1000 g mol^{-1}) low-resolution mass spectrometric data were obtained using a Waters Micromass Q-TOF micro mass spectrometer or Bruker UltraflexExtreme MALDI TOFTOF, utilising matrix assisted laser desorption ionisation (MALDI) and calibrated to poly-ethylene glycol standards (Mw 1000 – 3000 g mol^{-1}).

Low-temperature (77 K) nitrogen adsorption/desorption isotherms were obtained using a Coulter SA3100 surface area analyser. Accurately weighed samples of roughly 0.10 g were degassed for 900 mins at 135°C under high vacuum prior to analysis. Thermo-gravimetric analyses were performed on a Thermal Analysis SDT Q600 system, heating samples at a rate of 10°C/min from room temperature to 1000°C. Single crystal X-ray structures were recorded either at Cardiff University using a Bruker-Nonius Kappa CCD area-detector diffractometer equipped with an Oxford Cryostream low temperature cooling device operating at 150(2) K (λ = 0.71073 Å), or at station I19 of the Diamond Light Source using synchrotron radiation and a Rigaku Saturn 724 CCD diffractometer (graphite monochromated radiation). All structures were solved by direct methods and all calculations were carried out using the SHELX-97 package. CCDC-955894, CCDC-973327, CCDC-1406070, CCDC-1406071, CCDC-1406072 and CCDC-1406073 contain the supplementary crystallographic data for this paper. These data can be obtained free of charge from the Cambridge Crystallographic Data Centre *via* http://www.ccdc.cam.ac.uk/pages/Home.aspx.

Gel permeation chromatography (GPC) analyses were performed on chloroform solutions (1 mg ml^{-1}) using a GPC MAX variable loop equipped with two KF-805L SHODEX columns and a RI(VE3580) detector, operating at a flow rate of 1 ml min^{-1}. Calibration was achieved using Viscotek polystyrene standards (Mw 1000 –

1,000,000 g mol^{-1}). Due to the linear nature of the calibration standards, analysis of large, non-linear materials often results in an underestimation of molecular weight.[2] As such, M_w and M_n values reported for larger OMIMs are often substantially lower than the expected values.

Experimental

1,[3] **4c**[4] and **5a**[5] were prepared according to the relevant literature. Syntheses of **5c**, **5e**, **6**, **23** (**OMIM-1**), **25** (**OMIM-4**), **27** (**OMIM-5**) and **33** (**OMIM-6**) have been previously been reported by our group.[6]

Precursors

4,4'-Dicyano-2,2',3,3',5,5',6,6'-octafluorobiphenyl (**1**) and 4,4',4''-tricyano-2,2',2'',3,3'',5,5',5'',6,6',6''-undecafluoro-[1,1':3',1''-terphenyl] (**2**)

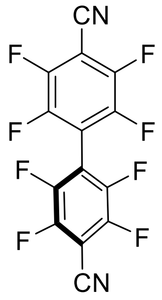

1

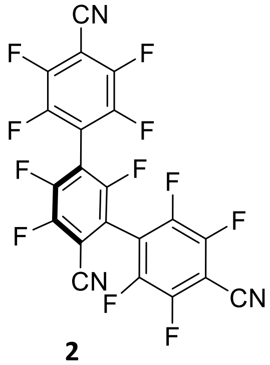

2

According to the literature procedure by Kaneko,[3] hexaethylphosphorous triamide (6.40 g, 25.9 mmol) was added dropwise to a solution of pentafluorobenzonitrile (10.0 g, 51.8 mmol) in anhydrous diethyl ether (30 ml). The reaction mixture was stirred for 3 hrs at room temperature then concentrated under reduced pressure and the crude reaction products purified *via* column chromatography (hexane/CH_2Cl_2, 3/2) to give:

4,4'-Dicyano-2,2',3,3',5,5',6,6'-octafluorobiphenyl (**1**): (R_f = 0.5, 3.05 g, 34%) as a white powder (mp 108 – 110°C) [lit. mp 129 – 131°C[7]]; IR (CH_2Cl_2 film) 2251, 1490, 1291, 1266, 1001 cm^{-1}; ^{19}F NMR (282 MHz, $CDCl_3$) δ -129.5 (m, 4F, Ar*F*), -133.4 (m, 4F, Ar*F*) [lit. ^{19}F NMR ($CDCl_3$) δ -129.8 (4F), -133.7 (4F)[8]]; ^{13}C NMR (125 MHz, $CDCl_3$) δ 148.8 (m), 146.2 (m), 145.4 (m), 143.0 (m) 111.9 (m), 106.7, 97.5 (m) (^{19}F-^{13}C coupling not assigned); HRMS (EI$^+$, *m/z*) calc. for $C_{14}F_8N_2$: 347.9934 (M$^+$), found 347.9932.

4,4',4''-Tricyano-2,2',2'',3,3'',5,5',5'',6,6',6''-undecafluoro-[1,1':3',1''-terphenyl] (**2**): (R_f = 0.4, 0.56 g, 6%) as a white powder (mp 194 – 196°C); IR (CH_2Cl_2 film) 2247, 1494, 1470, 1312, 1263, 1032 cm^{-1}; ^{19}F NMR (282 MHz, $CDCl_3$) δ -109.4 (m, 1F, Ar*F*), -125.0 (m, 1F, Ar*F*), -131.4 (m, 1F, Ar*F*), -133.5 (m, 2F, Ar*F*), -133.7 (m, 2F, Ar*F*), -136.3 (m, 2F, Ar*F*), -136.9 (m, 2F, Ar*F*); ^{13}C NMR (125 MHz, $CDCl_3$) δ 153.9 (m), 151.9 (m), 148.7 (m), 146.6 (m), 145.3 (m), 143.3 (m), 114.9 (m), 114.1 (m), 111.8 (m), 109.5 (m), 107.7 (m), 106.7 (m), 97.8 (m) (^{19}F-^{13}C coupling not assigned); HRMS (EI$^+$, *m/z*), calc. for $C_{21}F_{11}N_3$: 502.9917 (M$^+$), found 502.9925.

6,7-Dimethoxy-1,1,4,4-tetramethyl-1,2,3,4-tetrahydronaphthalene

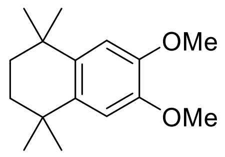

A solution of aluminium trichloride (4.19 g, 31.41 mmol) in nitromethane (30 ml) was added dropwise over the course of 10 mins to a solution of 1,2-dimethoxybenzene (1.08 g, 7.85 mmol) and 2,5-dichloro-2,5-dimethylhexane (2.42 g, 15.70 mmol) in nitromethane (30 ml). The purple solution was then stirred for 20 hrs at room temperature before being slowly poured into ice water (200 ml). The resultant precipitate was collected by filtration, refluxed in ethanol for 2 hrs and filtered to give 6,7-dimethoxy-1,1,4,4-tetramethyl-1,2,3,4-tetrahydronaphthalene (1.60 g, 80%) as a white solid (mp 60 - 62°C)[9]; ^{1}H NMR (400 MHz, $CDCl_3$) δ 6.77 (s, 2H, Ar*H*), 3.86 (s, 6H, OCH_3), 1.67 (s, 4H, CH_2), 1.27 (s, 12H, CCH_3); ^{13}C NMR (100 MHz, $CDCl_3$) δ 146.8, 137.0, 109.1, 55.8, 35.2, 34.0, 31.8; LRMS (APCI$^+$, *m/z*) calc. for $C_{16}H_{24}O_2$: 248.18 (M^+), found 248.18.

5,5,8,8-Tetramethyl-5,6,7,8-tetrahydronaphthalene-2,3-diol (3d)

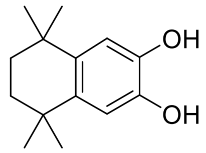

Boron tribromide (1.27 g, 5.07 mmol) was added dropwise to a solution of 6,7-dimethoxy-1,1,4,4-tetramethyl-1,2,3,4-tetrahydronaphthalene (0.630 g, 2.53 mmol) in anhydrous CH_2Cl_2 at 0°C. After stirring for 10 mins at 0°C and 3 hrs at room temperature, the reaction mixture was poured into ice water (100 ml) and allowed to stir for 20 mins. The resultant precipitate was filtered, washed with water (50 ml) and CH_2Cl_2 (150 ml) then dried to give 5,5,8,8-tetramethyl-5,6,7,8-tetrahydronaphthalene-2,3-diol (**3d**) (0.502 g, 90% as a white solid (mp 182 - 184°C)[10]; ^{1}H NMR (400 MHz, CH_3OD) δ 6.68 (s, 2H, Ar*H*), 1.64 (s, 4H, CH_2), 1.20 (s, 12H, CH_3); ^{13}C NMR (100 MHz, CH_3OD) δ 144.0, 137.3, 113.9, 36.5, 34.6, 32.4; HRMS (EI$^+$, *m/z*) calc. for $C_{14}H_{20}O$: 220.1463 (M^+), found 220.1463.

6-*tert*-Butylnaphthalene-2,3-diol (4b)

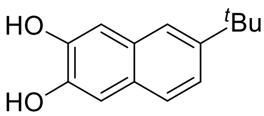

tert-Butanol (1.00 ml, 17.3 mmol) was added dropwise to a suspension of naphthalene-2,3-diol (2.77 g, 17.3 mmol) in trifluoroacetic acid (50 ml) and stirred for 72 hrs at room temperature. After quenching with water (250 ml), the precipitate was filtered, washed with water (500 ml) and dried under vacuum to give 6-*tert*-butylnaphthalene-2,3-diol (**4b**) (3.11 g, 84%) as a white powder (mp 160 – 162°C, literature mp 159 – 160°C[11]); IR (CH_2Cl_2 film) 3434, 2961, 2867, 1523, 1429, 1267, 1165, 1114 cm^{-1}; ^{1}H NMR (400 MHz, $(CD_3)_2SO$) δ 9.42 (s, 1H, O*H*), 9.39 (s, 1H, O*H*), 7.50 – 7.46 (m, 2H, Ar*H*), 7.27 (dd, *J* = 8.6, 1.8 Hz, 1H, Ar*H*), 7.05 (d, *J* = 13.1 Hz, 2H, Ar*H*), 1.32 (s, 9H, *t*Bu*H*); ^{13}C NMR (125 MHz, $(CD_3)_2SO$) δ 146.7, 146.2, 144.9, 128.6, 126.7, 125.2, 121.5, 120.5, 109.7, 109.1, 34.1, 31.1; HRMS (EI$^+$, *m/z*) calc. for $C_{14}H_{16}O_2$: 216.1150 (M^+), found 216.1152.

2,3-Dimethoxy-9,10-dimethyltriptycene

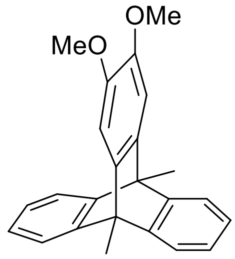

1,2-Epoxypropane (25 ml, 357 mmol) and freshly prepared 2-carboxy-4,5-dimethoxybenzenediazonium chloride[12] (12.5 g, 51.1 mmol) were added to a solution of 9,10-dimethylanthracene[13] (3.08 g, 14.9 mmol) in 1,2-dichloroethane (250 ml) and heated to reflux for 24 hrs. After cooling to room temperature, the reaction mixture was concentrated under reduced pressure then purified by column chromatography (CH_2Cl_2/hexane 4/1, R_f = 0.4) and triturated in methanol (50 ml) for 2 hrs to give 2,3-dimethoxy-9,10-dimethyltriptycene (3.17 g, 62%) as a white powder (mp 186 – 188°C); IR (CH_2Cl_2 film) 3063, 2969, 2827, 1597, 1506, 1447, 1289, 1200, 1040 cm^{-1}; ^{1}H NMR (400 MHz, $CDCl_3$) δ 7.39 (dd, J = 5.4, 3.2 Hz, 4H, ArH), 7.06 (dd, J = 5.4, 3.2 Hz, 4H, ArH), 7.01 (s, 2H, ArH), 3.88 (s, 6H, OCH_3), 2.45 (s, 6H, CCH_3); ^{13}C NMR (125 MHz, $CDCl_3$) δ 148.8, 146.1, 141.5, 124.8, 120.3, 106.5, 56.6, 48.4, 13.8; HRMS (EI^+, m/z) calc. for $C_{24}H_{22}O_2$: 342.1620 (M^+), found 342.1626.

9,10-Dimethyltriptycene-2,3-diol (5b)

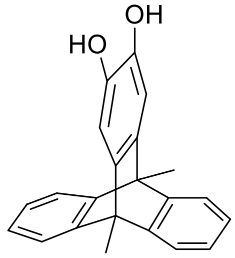

Boron tribromide (0.60 ml, 6.33 mmol) was added dropwise to a solution of 2,3-dimethoxy-9,10-dimethyltriptycene (1.01 g, 2.95 mmol) in anhydrous CH_2Cl_2 (10 ml) at 0°C. After stirring for 10 mins at 0°C and 3 hrs at room temperature, the reaction was re-cooled to 0°C and quenched with the dropwise addition of ice water (20 ml). The organic layer was collected, washed with water (3 × 20 ml) and brine (20 ml) then dried over anhydrous magnesium sulfate. After concentrating the residue down to ≈10 ml, the product was afforded as a precipitate *via* the dropwise addition of hexane, filtered, washed with hexane and dried under suction/nitrogen flow to give 9,10-dimethyltriptycene-2,3-diol (**5b**) (0.90 g, 97%) as a white solid (mp > 300°C). IR (CH_2Cl_2 film) 3372, 3063, 2970, 1695, 1446, 1304 cm^{-1}; ^{1}H NMR (400 MHz, $(CD_0)_2CO$) δ 7.55 (s, 2H, OH), 7.34 (dd, J = 8.8, 5.2 Hz, 4H, ArH), 6.99 (dd, J = 8.8, 5.2 Hz, 4H, ArH), 6.91 (s, 2H, ArH), 2.32 (s, 6H, CH_3); ^{13}C NMR (125 MHz, $(CD_3)_2CO$) δ 150.1, 142.1, 141.3, 125.3, 120.9, 110.1, 48.7, 14.1; HRMS (EI^+, m/z) calc. for $C_{22}H_{18}O_2$: 314.1307 (M^+), found 314.1304.

2,3-Dimethoxy-7,14-di-*tert*-butyl-9,10-dimethyltriptycene.

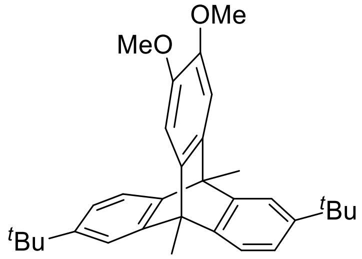

1,2-Epoxypropane (25 ml, 357 mmol) and freshly prepared 2-carboxy-4,5-dimethoxybenzenediazonium chloride[12] (11.9 g, 48.6 mmol) were added to a solution of 2,6-di-*tert*-butyl-9,10-dimethylanthracene[14] (3.03 g, 9.51 mmol) in 1,2-dichloroethane (200 ml). The mixture was heated to reflux for 24 hrs, cooled to room temperature and concentrated under reduced pressure. The crude mixture was then purified by column chromatography (CH_2Cl_2/hexane 1/1, R_f = 0.4) and triturated in methanol (50 ml) for 2 hrs to give 2,3-dimethoxy-7,14-di-*tert*-butyl-9,10-dimethyltriptycene (1.94 g, 45%) as a white powder (mp 222 – 224°C); IR (CH_2Cl_2 film) 2964, 2903, 2867, 1481, 1458, 1291, 1241, 1200, 1044 cm^{-1}; ^{1}H NMR (400 MHz, $CDCl_3$) δ 7.35 (d, *J* = 2.9 Hz, 2H, Ar*H*), 7.24 (d, *J* = 12.6 Hz, 2H, Ar*H*), 7.01 (dd, *J* = 12.6, 2.9 Hz, 2H, Ar*H*), 6.94 (s, 2H, Ar*H*), 3.83 (s, 6H, OCH_3), 2.39 (s, 6H, CCH_3), 1.25 (s, 18H, *t*Bu*H*); ^{13}C NMR (125 MHz, $CDCl_3$) δ 148.6, 147.5, 146.1, 145.9, 141.9, 121.3, 119.8, 117.6, 106.2, 56.5, 48.3, 34.8, 31.8, 13.9; HRMS (EI^+, *m/z*) calc. for $C_{32}H_{38}O_2$: 454.2872 (M^+), found 454.2874.

7,14-Di-*tert*-butyl-9,10-dimethyltriptycene-2,3-diol (5d)

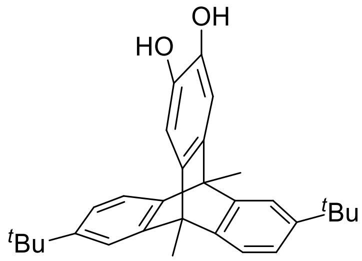

Boron tribromide (0.62 ml, 6.43 mmol) was added dropwise to a solution of 2,3-dimethoxy-7,14-di-*tert*-butyl-9,10-dimethyltriptycene (1.46 g, 2.20 mmol) in anhydrous CH_2Cl_2 (10 ml) at 0°C. After stirring for 10 mins at 0°C and 3 hrs at room temperature, the reaction was re-cooled to 0°C and quenched with the dropwise addition of ice water (20 ml). The organic layer was collected, washed with water (3 × 20 ml) and brine (20 ml), dried over anhydrous magnesium sulfate, concentrated under reduced pressure and dried under nitrogen flow to give 7,14-di-*tert*-butyl-9,10-dimethyltriptycene-2,3-diol (**5d**) (1.30 g, 95%) as a pale brown solid (mp > 300°C). IR (CH_2Cl_2 film) 3390, 2965, 2903, 2868, 1616, 1480, 1455, 1362, 1302 cm^{-1}; ^{1}H NMR (400 MHz, $(CD_3)_2CO$) δ 7.53 (s, 2H, O*H*), 7.40 (d, *J* = 1.8 Hz, 2H, Ar*H*), 7.23 (d, *J* = 7.9 Hz, 2H, Ar*H*), 7.01 (dd, *J* = 7.8, 1.9 Hz, 2H, Ar*H*), 6.89 (s, 2H, Ar*H*), 2.32 (s, 6H, CH_3), 1.24 (s, 18H, *t*Bu*H*); ^{13}C NMR (125 MHz, $(CD_3)_2CO$) δ 149.8, 147.9, 147.3, 142.0, 141.6, 121.6, 120.4, 118.0, 110.1, 48.5, 36.2, 31.9, 14.2; HRMS (EI^+, *m/z*) calc. for $C_{30}H_{34}O_2$: 426.2559 (M^+), found 426.2562.

4,5-Dimethoxy-1,1'-biphenyl

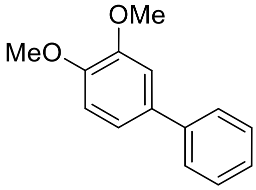

Phenylboronic acid (4.35 g, 35.7 mmol), 4-bromoveratrole (9.30 g, 42.8 mmol) and potassium carbonate (9.80 g, 71.4 mmol) were added to a degassed solution of water (100 ml) and THF (100 ml). The solution was heated to 80°C and $Pd(PPh_3)_4$ (0.28 g, 0.25 mmol) was added. After 36 hrs, the reaction mixture was cooled to room temperature, the THF removed under vacuum and the products extracted into CH_2Cl_2 (3 × 40 ml). The combined organic extracts were dried over anhydrous magnesium sulfate, concentrated under reduced pressure and filtered through a silica plug eluting first with hexane and then with CH_2Cl_2. Further purification was achieved by recrystallization from methanol to give 4,5-dimethoxy-1,1'-biphenyl (7.17 g, 93%) as colourless crystals (mp 68 – 70°C, Lit[15] 68 – 69°C); IR (CH_2Cl_2 film) 3059, 3028, 2962, 2935, 2834, 1606, 1590, 1521, 1488, 1466, 1441, 1407, 1253, 1217, 1173, 1144, 1026 cm^{-1}; ^1H NMR (400 MHz, $CDCl_3$) δ 7.57 (d, *J* = 7.3 Hz, 2H, Ar*H*), 7.43 (t, *J* = 7.6 Hz, 2H, Ar*H*), 7.32 (t, *J* = 7.3 Hz, 1H, Ar*H*), 7.16 (dd, *J* = 8.2, 1.9 Hz, 1H, Ar*H*), 7.12 (d, *J* = 1.9 Hz, 1H, Ar*H*), 6.96 (d, *J* = 8.2 Hz, 1H, Ar*H*), 3.96 (s, 3H, OCH_3), 3.93 (s, 3H, OCH_3); ^{13}C NMR (100 MHz, $CDCl_3$) δ 149.3, 148.7, 141.1, 134.3, 128.8, 126.9, 119.4, 111.5, 110.5, 56.0; LRMS, ($APCI^+$, *m/z*): calc. for $C_{14}H_{15}O_2$: 214.10 (M^+), found 215.11 (MH^+).

4,5-Dimethoxy-2-bromo-1,1'-biphenyl

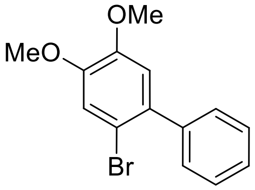

Bromine (7.60 g, 47.9 mmol) was added dropwise to a solution of 4,5-dimethoxy-1,1'-biphenyl (9.30 g, 43.5 mmol) in chloroform (150 ml) at 0°C. The reaction was stirred for 24 hrs at room temperature then poured into water (300 ml). The organic layer was separated, washed with an aqueous solution of sodium thiosulfate, dried over anhydrous magnesium sulfate and concentrated under reduced pressure. The crude material was recrystallised from methanol to give 4,5-dimethoxy-2-bromo-1,1'-biphenyl (10.4 g, 92%) as a white solid (mp 110 – 112°C, Lit[16] 110-112°C); IR (CH_2Cl_2 film) 3059, 2964, 2938, 2910, 2838, 1603, 1519, 1505, 1489, 1441, 1379, 1208, 1020 cm^{-1}; ^1H NMR (400 MHz, $CDCl_3$) δ 7.42 (m, 5H, Ar*H*), 7.13 (s, 1H, Ar*H*), 6.84 (s, 1H, Ar*H*), 3.92 (s, 3H, OCH_3), 3.87 (s, 3H, OCH_3); ^{13}C NMR (100 MHz, $CDCl_3$) δ 148.7, 148.2, 141.0, 134.7, 129.5, 127.9, 127.4, 115.7, 113.8, 112.4, 56.2, 56.0: LRMS, (EI^+, *m/z*) calc. for $C_{14}H_{13}BrO_2$: 292.01 ($^{79}BrM^+$) found 292.01.

2,3-Dimethoxy-9,9'-spirobisfluorene

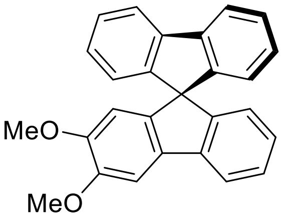

n-Butyllithium (2.5 M in hexanes, 2.90 ml, 7.25 mmol) was added dropwise to a solution of 4,5-dimethoxy-2-bromo-1,1'-biphenyl (2.00 g, 6.80 mmol) in anhydrous THF (45 ml) at -78°C. After 90 mins, 9-fluorenone (1.03 g, 5.70 mmol) was added and the reaction mixture allowed to slowly warm to room temperature, stirred for 12 hrs then quenched with water and extracted with CH_2Cl_2 (3 × 40 ml). The organic layers were combined, washed with water, dried over anhydrous magnesium sulfate and concentrated under reduced pressure. The pale yellow solid was triturated in methanol to afford a white solid and dissolved in cold glacial acetic acid (100 ml) before conc. hydrochloric acid (2 ml) was added dropwise to the solution. After refluxing for 3 hrs, the mixture was cooled to room temperature and poured into water yielding a precipitate, which was collected and triturated in methanol to afford 2,3-dimethoxy-9,9'-spirobisfluorene (1.20 g, 56%) as a white solid (mp 179 - 181°C); IR (CH_2Cl_2 film) 3061, 3002, 2952, 2934, 2856, 2831, 1605, 1500, 1475, 1445, 1344, 1273, 1205, 1176, 1130, 1084, 1034 cm^{-1}; ^{1}H NMR (400 MHz, $CDCl_3$) δ 7.85 (d, *J* = 7.6 Hz, 2H, Ar*H*), 7.73 (d, *J* = 7.6 Hz, 1H, Ar*H*), 7.35 (m, 4H, Ar*H*), 7.12 (td, *J* = 7.5, 1.0 Hz, 2H, Ar*H*), 7.02 (td, *J* = 7.5, 1.0 Hz, 1H, Ar*H*), 6.75 (d, *J* = 7.6 Hz, 2H, Ar*H*), 6.65 (d, *J* = 7.5 Hz, 1H, Ar*H*), 6.25 (s, 1H, Ar*H*) 4.04 (s, 3H, OCH_3), 3.63 (s, 3H, OCH_3); ^{13}C NMR (100 MHz, $CDCl_3$) δ 149.4, 149.3, 149.0, 148.8, 141.9, 141.6, 140.6, 134.3, 127.8, 127.6, 127.5, 126.5, 124.0, 123.6, 119.9, 118.8, 106.7, 102.8, 65.8, 56.1, 56.0; HRMS (EI^+, *m/z*) calc. for $C_{27}H_{20}O_2$: 376.1463 (M^+), found 376.1461.

2,3-Dihydroxy-9,9'-spirobifluorene (7)

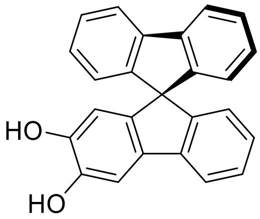

Boron tribromide (0.87 g, 3.40 mmol) was added dropwise to a solution of 2,3-dimethoxy-9,9'-spirobifluorene (0.60 g, 1.70 mmol) in anhydrous CH_2Cl_2 (15 ml) at 0°C. The reaction mixture was stirred for 1 hr at room temperature then poured into water (30 ml). The CH_2Cl_2 was allowed to evaporate and the resulting precipitate was collected, washed with water then with a mixture of CH_2Cl_2/hexane (1/2) and dried under high vacuum to give 2,3-dihydroxy-9,9'-spirobifluorene (**7**) (0.54 g, 91%) as a white powder (mp decomposes above 70°C); IR (nujol) 3523, 1607, 1495, 1365, 1291, 1181, 1117 cm^{-1}; ^{1}H NMR (400 MHz, $(CD_3)_2CO$) δ 7.95 (d, *J* = 7.6 Hz, 2H, Ar*H*), 7.77 (d, *J* = 7.6 Hz, 1H, Ar*H*), 7.43 (s, 1H, Ar*H*), 7.38 (td, *J* = 7.6, 1.0 Hz, 2H, Ar*H*), 7.31 (td, *J* = 7.5, 1.0 Hz, 1H, Ar*H*), 7.14 (td, *J* = 7.5, 1.0 Hz, 2H, Ar*H*), 7.01 (td, *J* = 7.5, 1.0 Hz, 1H, Ar*H*), 6.69 (d, *J* = 7.6 Hz, 2H, Ar*H*), 6.56 (d, *J* = 7.5 Hz, 1H, Ar*H*), 6.14 (s, 1H, Ar*H*); ^{13}C NMR (100 MHz, $(CD_3)_2CO$) δ 151.4, 151.2, 150.5, 147.2, 147.1, 144.3, 143.4, 141.9, 135.7, 129.6, 129.5, 129.4, 128.0, 125.5, 125.1, 121.9, 120.8, 112.1, 108.7, 67.3; HRMS (EI^+, *m/z*) calc. for $C_{25}H_{16}O_2$: 348.1150, found 348.1155.

Spiro[9H-fluorene-9,9'(10'H)-phenanthrene]-10'-one[17]

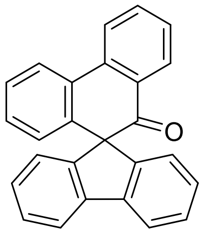

Zinc powder (30 mesh, 30.0 g, 45.9 mmol) was added to a solution of 9-fluorenone (6.00 g, 33.3 mmol) and zinc (II) chloride (6.00 g, 44.0 mmol) in THF (30 ml) and water (30 ml) with vigorous stirring. After 3 hrs, the suspension turned white and 3N HCl (30 ml) was added before the zinc was filtered off and the filtrate extracted with toluene. The solvents were removed under reduced pressure to afford 6.0 g of crude 9,9'-bifluorenyl-9,9'-diol which was used in the next step without further purification. Sulfuric acid (96%, 3 ml) was added dropwise to a solution of the crude diol (6.00 g, 16.6 mmol) in glacial acetic acid (225 ml), heated to 90°C and stirred for 45 mins. After cooling the reaction mixture to room temperature, it was poured into water (250 ml), the resulting precipitate collected, washed with water and then recrystallised from methanol to give spiro[9H-fluorene-9,9'(10'H)-phenanthrene]-10'-one (4.5 g, 78%) as colourless crystals (mp 253 - 255°C (lit[17] mp 254 - 255°C); IR (CH_2Cl_2 film) 3071, 1683, 1601, 1450, 1266, 1132 cm^{-1}; ^{1}H NMR (400 MHz,) δ 8.20 (d, J = 8.0 Hz, 1H), 8.11 (d, J = 8.0 Hz, 1H), 8.01 (dd, J = 7.8, 1.5 Hz, 1H), 7.84 – 7.75 (m, 2H), 7.46 (td, J = 7.5, 1.1 Hz, 1H), 7.45 – 7.31 (m, 2H), 7.19 (td, J = 7.5, 1.2 Hz, 2H), 7.16 – 7.00 (m, 2H), 6.64 (dd, J = 7.9, 1.4 Hz, 1H); ^{13}C NMR (100 MHz, $CDCl_3$) *δ* 197.7, 147.5, 142.1, 139.8, 138.6, 135.4, 130.9, 130.5, 129.7, 129.0, 128.8, 128.6, 128.5, 128.4, 125.2, 124.6, 123.7, 121.0, 69.2; LRMS, (APCI$^+$, *m/z*): calc. for $C_{26}H_{16}O$: 344.12 (M^+), found 345.15 (MH^+).

Dimethoxypropellane

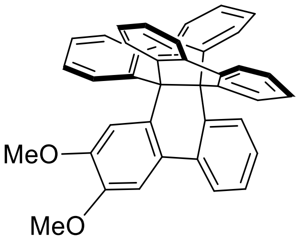

n-Butyllithium (2.5 M in hexanes, 2.56 ml, 6.40 mmol) was added dropwise to a solution of 4,5-dimethoxy-2-bromo-1,1'-biphenyl (1.88 g, 6.40 mmol) in anhydrous diethylether (60 ml) at -78°C. After 1 hr, spiro[9H-fluorene-9,9'(10'H)-phenanthrene]-10'-one (2.00 g, 5.81 mmol) was added, the reaction allowed to slowly warm to room temperature and stirred for 12 hrs. After quenching with water, the mixture was extracted with CH_2Cl_2 (3 × 40 ml), the organic extracts combined, washed with water and dried over anhydrous magnesium sulfate. After removing the solvents under reduced pressure, the afforded pale yellow solid was dissolved in cold glacial acetic acid (150 ml) and concentrated sulfuric acid (6 ml) was added dropwise then stirred at 95°C for 2 hrs. After cooling to room temperature the mixture was poured into water (150 ml), extracted with CH_2Cl_2 (3 × 50 ml), the organic extracts combined, washed with aqueous NaOH (10%, 30 ml), water (2 × 50 ml) and brine (50 ml) and dried over anhydrous magnesium sulfate The solvents were removed under reduced pressure to give a light brown solid which was re-precipitated from CH_2Cl_2 with hexane to yield dimethoxypropellane (1.40 g, 45%) as a white solid (mp > 300°C); IR (CH_2Cl_2 film) 3062, 3024, 2934, 2849, 1604, 1511, 1446, 1394, 1340, 1277, 1026 cm^{-1}; ^{1}H NMR (400 MHz, $CDCl_3$) *δ* 7.63 (m, 4H, Ar*H*), 7.52 (d,

J = 7.6 Hz, 1H, Ar*H*), 7.20 (m, 5H, Ar*H*), 7.12 (s, 1H, Ar*H*), 7.03 (m, 5H, Ar*H*), 6.68 (m, 5H, Ar*H*), 6.16 (s, 1H, Ar*H*), 3.88 (s, 3H, OC*H*$_3$), 3.51 (s, 3H, OC*H*$_3$); ^{13}C NMR (100 MHz, $CDCl_3$) δ 148.4, 148.3, 140.7, 139.9, 139.7, 139.4, 135.3, 135.2, 135.1, 132.4, 129.6, 129.4, 129.3, 129.2, 128.3, 127.9, 127.8, 127.7, 127.6, 126.9, 124.1, 124.0, 123.4, 112.9, 107.3, 56.1, 56.0, 53.6, 53.2; HRMS (ES$^+$, *m/z*) calc. for $C_{40}H_{28}O_2$: 540.2089 (M$^+$), found 540.2081.

Dihydroxypropellane (**8**)

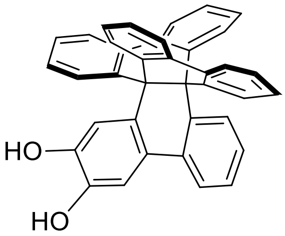

Boron tribromide (0.77 ml, 8.11 mmol) was added dropwise to a solution of dimethoxypropellane (1.44 g, 2.64 mmol) in anhydrous CH_2Cl_2 (150 ml) at 0°C. After stirring for 3 hrs at room temperature, the reaction was quenched with water and the white precipitate formed was filtered, washed with CH_2Cl_2 and dried under vacuum to give dihydroxypropellane (**8**) (1.10 g, 79%) as a white solid (mp decomposes before melting); IR (nujol) 3532, 1606, 1572, 1511, 1302, 1179 cm^{-1}; ^{1}H NMR (400 MHz, $(CD_3)_2CO$) δ 7.73 (m, 4H, Ar*H*), 7.54 (dd, J = 7.8, 0.9 Hz, 1H, Ar*H*), 7.21 (m, 6H, Ar*H*), 7.06 (m, 4H, Ar*H*), 6.96 (td, J = 7.6, 1.2 Hz, 1H, Ar*H*), 6.78 (dd, J = 7.6, 1.2 Hz, 1H, Ar*H*), 6.60 (m, 4H, Ar*H*), 6.09 (s, 1H, Ar*H*); ^{13}C NMR (125 MHz, $CDCl_3$) 145.3, 145.2, 145.0, 145.0, 140.8, 140.5, 139.5, 136.1, 135.8, 135.8, 135.7, 132.1, 130.0, 129.9, 129.8, 129.7, 126.9, 124.7, 124.7, 124.6, 124.5, 123.9, 117.3, 112.1, 54.1, 53.4, some carbons missing; HRMS (ES$^+$, *m/z*) calc. for $C_{38}H_{24}O_2$: 512.1776 (M$^+$), found 512.1792.

1,2-Dimethoxy-4-(phenylethynyl)benzene

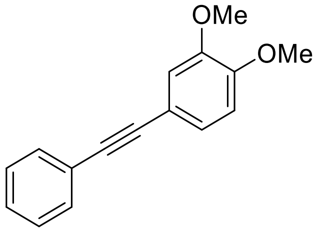

A mixture of 4-bromoveratrole (0.61 g, 2.82 mmol), triethylamine (4 ml) and DMF (4 ml) was deoxygenated before 4-ethynyl-1,2-dimethoxybenzene (0.32 g, 3.10 mmol) was added and the reaction mixture heated to 90°C. Copper (I) iodide (0.054 g, 0.28 mmol) and $Pd(PPh_3)_2Cl_2$ (0.198 g, 0.28 mmol) were then added and the reaction left to stir at 90°C 15 hrs. The reaction was then quenched with water (40 ml), allowed to stir for 1 hr then extracted with CH_2Cl_2 (3 × 30 ml). The combined organic extracts were combined, washed with water (3 × 20 ml), dried over anhydrous magnesium sulfate concentrated under reduced pressure and purified *via* column chromatography (petroleum ether/ethyl acetate) to give 1,2-dimethoxy-4-(phenylethynyl)benzene (0.47 g, 70%) as a yellow solid (mp 94 - 95˚C (lit. 93 - 95˚C)[18]; IR(CH_2Cl_2 film) 2363, 1594, 1512, 1465, 1328, 1251, 1225, 1138, 1124, 1025 cm^{-1}; ^{1}H NMR (400 MHz, $CDCl_3$) δ 7.98 (m, 1H, Ar*H*), 7.96 (m, 1H, Ar*H*), 7.64 (m, 2H, Ar*H*), 7.50 (m, 3H, Ar*H*), 6.89 (d, J = 8.2 Hz, 1H, Ar*H*), 3.96 (s, 3H, OC*H*$_3$) 3.95 (s, 3H, OC*H*$_3$); ^{13}C NMR (100 MHz, $CDCl_3$) δ 149.4, 148.5, 131.4, 128.3, 127.9, 124.8, 123.3, 115.3, 114.1, 110.9, 89.4, 87.9, 55.8, 55.8; LRMS (APCI$^+$, *m/z*) calc. for $C_{16}H_{14}O_2$: 238.19 (M$^+$), found 239.21 (MH$^+$).

1-(1,2-Dimethoxyphenyl)-2,3,4,5,6-pentaphenylbenzene

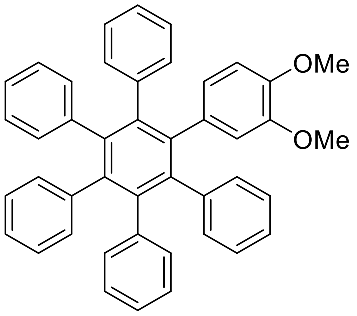

A solution of 2,3,4,5-tetraphenylcyclopenta-2,4-dienone (3.00 g, 7.81 mmol) and 1,2-dimethoxy-4-(phenylethynyl)benzene (2.23 g, 9.37 mmol) in diphenylether (8 ml) was refluxed for 15 hrs. After cooling to room temperature, the solution was poured into cold methanol to which hexane (50 ml) was added and the resultant precipitate filtered and purified *via* column chromatography (hexane/ethyl acetate, 7/3) to give 1-(1,2-dimethoxyphenyl)-2,3,4,5,6-pentaphenylbenzene (3.39 g, 73%) as a light orange powder (mp 264 – 266˚C); IR (CH_2Cl_2 film) 3062, 2896, 2735, 2467, 1536, 1477, 1437, 1265, 1041; ^{1}H NMR (500 MHz, $CDCl_3$) δ 6.84 (m, 25H, Ar*H*) 6.35 (m, 3H, Ar*H*) 3.68 (s, 3H, OCH_3) 3.42 (s, 3H, OCH_3); ^{13}C NMR (125 MHz, $CDCl_3$) δ 147.2, 146.4, 144.3, 140.8, 140.6, 140.6, 140.4, 140.4, 140.2, 139.8, 133.0, 132.9, 131.4, 131.4, 131.3, 131.2, 126.7, 126.6, 126.5, 124.2, 115.6, 109.5, 55.5, 55.4; LRMS (EI$^+$, *m/z*) calc. for $C_{44}H_{34}O_2$: 594.26 (M$^+$), found 594.27.

1-(1,2-Dihydroxyphenyl)-2,3,4,5,6-pentaphenylbenzene (**9**)

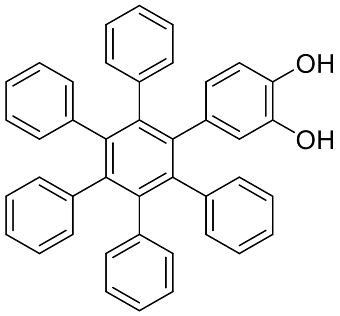

Boron tribromide (1.05 g, 4.21 mmol) was added dropwise to a solution of 1-(1,2-dimethoxyphenyl)-2,3,4,5,6-pentaphenylbenzene (1.00 g, 1.68 mmol) in anhydrous CH_2Cl_2 (30 ml) at 0°C. The reaction was then allowed to warm to room temperature and stir for a further 30 mins before being poured into ice water and left under vigorous stirring until all the CH_2Cl_2 evaporated. The resultant precipitate was filtered, washed with water and dried under vacuum before being re-precipitated from CH_2Cl_2/hexane (1/1) to give 1-(1,2-dihydroxyphenyl)-2,3,4,5,6-pentaphenylbenzene (**9**) (0.83 g, 87%) as an off-white solid (mp > 300 ˚C); IR (CH_2Cl_2 film) 3533, 3041, 1622, 1509, 1401, 1260, 1143, 1013, 1024; ^{1}H NMR (500 MHz, $(CD_3)_2CO$) δ 6.82 (m, 25H, Ar*H*) 6.39 (d, *J* = 2.0 Hz, 1H, Ar*H*) 6.32 (d, *J* = 8.1 Hz, 1H, Ar*H*) 6.22 (dd, *J* = 2.0, 8.1 Hz, 1H, Ar*H*); ^{13}C NMR (500 MHz, $(CD_3)_2CO$) δ 145.2, 144.2, 142.8, 142.7, 142.7, 142.4, 142.2, 142.1, 141.8, 134.3, 133.2, 133.1, 133.1, 128.3, 126.9, 126.9, 125.1, 120.4, 118.5, 118.3, 118.0, 115.4; HRMS calc.for $C_{42}H_{30}O_2$: 566.2246 (M$^+$), found 566.2249.

Biphenyl adducts/OMIMs

General Procedure 1 for OMIM synthesis:

Anhydrous fluorinated and catechol compounds were added to an oven-dried flask. After flushing with dry nitrogen for 5 mins, anhydrous DMF was added *via* syringe and the reaction mixture heated to form a solution, at which point, oven dried potassium carbonate was quickly added, the reaction was sealed under dry nitrogen flow, heated to 65°C and left to stir for 48 hrs. After cooling to room temperature, the reaction mixture was poured into water (20 times DMF volume), acidified with 2N HCl and allowed to stir as a suspension for 2 hrs. The crude product was then collected *via* filtration, washed with water (20 times DMF volume) and methanol (20 times DMF volume), then dried under suction. Following any required purification, the product was further dried in a 100°C vacuum oven.

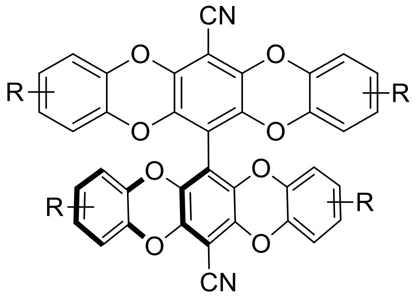

10 (R = H)
11 (R = *t*Bu)

10

4,4'-Dicyano-2,2',3,3',5,5',6,6'-octafluorobiphenyl (**1**) (0.230 g, 0.661 mmol), catechol (**3a**) (0.306 g, 2.78 mmol) and potassium carbonate (0.913 g, 6.61 mmol) were reacted together in anhydrous DMF (10 ml) in accordance with general procedure 1. Purification was achieved *via* column chromatography (CH_2Cl_2/hexane, 1/1, R_f = 0.4) to give **10** (0.307 g, 74%) as a yellow powder (mp > 300°C); IR (CH_2Cl_2 film) 2234, 1495, 1440, 1309, 1272, 1253 cm^{-1}; ^1H NMR (500 MHz, $CDCl_3$) δ 7.00 (d, J = 8.0 Hz, 4H, Ar*H*), 6.96 – 6.93 (m, 4H, Ar*H*), 6.88 – 6.85 (m, 4H, Ar*H*), 6.66 (d, J = 8.0 Hz, 4H, Ar*H*); ^{13}C NMR (125 MHz, $CDCl_3$) δ 141.1, 140.8, 140.0, 136.2, 125.3, 125.2, 117.0, 114.4, 110.6, 91.7 (one carbon missing); HRMS (EI^+, *m/z*) calc. for $C_{38}H_{16}N_2O_8$: 628.0907 (M^+), found 628.0903; GPC analysis ($CHCl_3$) M_n = 548, M_w = 578 g mol^{-1} relative to polystyrene, M_w/M_n = 1.055; BET surface area = 7 m^2 g^{-1}; total pore volume = 0.03 cm^3 g^{-1} at p/p^o = 0.98. Crystallography data ($CHCl_3$/MeOH): Triclinic, space group = $P\bar{1}$, a = 11.0410(6) Å, b = 11.3324(7) Å, c = 14.2766(7) Å, α = 113.045(5), β = 90.659(4), γ = 106.663(5), V = 1559.14 Å^3, Z = 2, R_1 = 4.01. (CCDC 1406071)

11

4,4'-Dicyano-2,2',3,3',5,5',6,6'-octafluorobiphenyl (**1**) (0.184 g, 0.529 mmol), 4-*tert*-butylcatechol (**3b**) (0.359 g, 2.11 mmol) and potassium carbonate (0.729 g, 5.27 mmol) were reacted together in anhydrous DMF (10 ml) in accordance with general procedure 1. Purification was achieved *via* column chromatography (hexane/CH_2Cl_2, 7/3, R_f = 0.3) to give **11** (mixture of regioisomers) (0.360 g, 80%) as a yellow powder (mp > 300°C); IR (CH_2Cl_2 film) 2964, 2907, 2871, 2238, 1512, 1436, 1287, 1122 cm^{-1}; ^1H NMR (400 MHz, $CDCl_3$) δ 7.04 – 6.52 (m, 12H, Ar*H*), 1.24 – 1.13 (m, 36H, *t*Bu*H*); ^{13}C NMR (125 MHz, $CDCl_3$) δ 149.11, 149.10, 149.05, 149.03, 148.97 148.93, 148.91, 148.8, 140.5, 140.43, 140.38, 140.3, 140.24, 140.20, 140.1, 140.0, 139.9 139.8, 138.82, 137.77, 138.7, 138.6, 138.5, 138.4, 138.3, 138.21, 138.16, 136.4, 136.2, 136.0, 135.9, 133.5, 133.1, 121.93, 121.88, 121.7, 116.3, 116.2, 114.30, 114.26, 114.2, 111.62, 111.59, 110.2, 91.4, 91.3, 34.74, 34.70, 34.67, 31.41, 31.39, 31.35 (extra carbons due to regioisomeric nature); LRMS (EI^+, *m/z*) calc. for

$C_{54}H_{48}N_2O_8$: 852.33 (M^+), found 852.31; GPC analysis ($CHCl_3$) M_n = 881, M_w = 922 g mol^{-1} relative to polystyrene, M_w/M_n = 1.047; BET surface area = 41 m^2 g^{-1}; total pore volume = 0.15 cm^3 g^{-1} at p/p^o = 0.98.

12

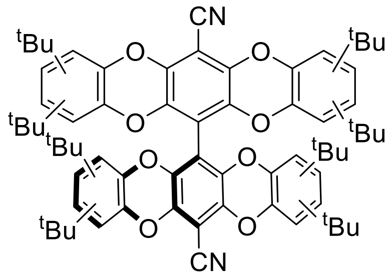

4,4'-Dicyano-2,2',3,3',5,5',6,6'-octafluorobiphenyl (**1**) (0.269 g, 0.773 mmol), 3,5-di-*tert*-butylcatechol (**3c**) (0.722 g, 3.25 mmol) and potassium carbonate (1.07 g, 7.75 mmol) were reacted together in anhydrous DMF (10 ml) in accordance with general procedure 1. Purification was achieved *via* column chromatography (CH_2Cl_2/hexane, 1/1, R_f = 0.3) to give **12** (mixture of regioisomers) (0.580 g, 70%) as a yellow powder (mp > 300°C); IR (CH_2Cl_2 film) 2963, 2907, 2871, 2237, 1451, 1413, 1304, 1276, 1231, 1037, 1018 cm^{-1}; ^{1}H NMR (400 MHz, $CDCl_3$) δ 6.97 – 6.81 (m, 5H, Ar*H*), 6.67 – 6.60 (m, 3H, Ar*H*), 1.51 – 1.07 (m, 72H, *t*Bu*H*); ^{13}C NMR (125 MHz, $CDCl_3$) δ 147.50, 147.48, 147.4, 147.28, 147.26, 147.24, 147.21, 141.0, 140.8, 140.6, 140.6, 140.4, 140.1, 139.91, 139.89, 139.8, 139.7, 139.6, 137.9, 137.8, 137.6, 137.53, 137.49, 137.46, 137.2, 137.1, 136.91, 136.86, 136.8, 136.21, 136.16, 136.1, 136.0, 135.91, 135.86, 119.6, 119.4, 112.6, 112.5, 112.4, 112.3, 111.8, 111.4, 90.5, 35.42, 35.36, 35.34, 35.33, 34.9, 31.4, 30.2, 30.08, 30.05, 30.0, 29.5, 29.43, 29.38, 29.35, 29.3 (extra carbons due to regioisomeric nature); LRMS (MALDI, *m/z*) calc. for $C_{70}H_{80}N_2O_8$: 1076.59 (M^+), found 1076.71; GPC analysis ($CHCl_3$) M_n = 1011, M_w = 1062 g mol^{-1} relative to polystyrene, M_w/M_n = 1.089; BET surface area = 67 m^2 g^{-1}; total pore volume = 0.231 cm^3 g^{-1} at p/p^o = 0.98.

13

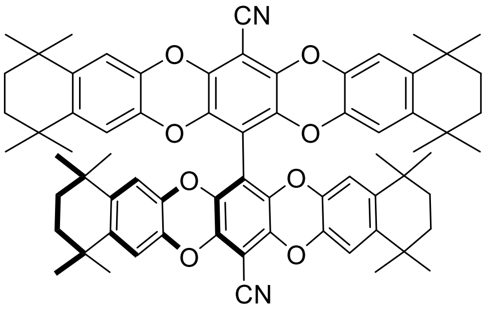

4,4'-Dicyano-2,2',3,3',5,5',6,6'-octafluorobiphenyl (**1**) (0.188 g, 0.54 mmol), 5,5,8,8-tetramethyl-5,6,7,8-tetrahydronaphthalene-2,3-diol (**3d**) (0.500 g, 2.26 mmol) and potassium carbonate (1.87 g, 13.56 mmol) were reacted together in anhydrous DMF (8 ml) in accordance with general procedure 1. Purification was achieved *via* column chromatography (petroleum ether/ethyl acetate, 4/1) to give **13** (0.520 g, 90%) as yellow solid (mp > 300°C); ^{1}H NMR (400 MHz, $CDCl_3$) δ 6.92 (s, 4H, Ar*H*), 6.64 (s, 4H, Ar*H*), 1.62 (s, 16H, C*H*$_2$), 1.24 (s, 24H, C*H*$_3$), 1.16 (s, 24H, C*H*$_3$); ^{13}C NMR (100 MHz, $CDCl_3$) δ 142.4, 142.0, 139.5, 138.5, 138.1, 135.7, 114.5, 114.2, 111.7, 111.2, 90.9, 35.0, 34.9, 34.3, 31.9, 31.8; LRMS (MALDI, *m/z*) calc. for $C_{70}H_{72}N_2O_8$: 1068.53 (M^+), found 1068.94; BET surface area = 51 m^2 g^{-1}; total pore volume = 0.14 cm^3 g^{-1} at p/p^o = 0.98. TGA analysis (nitrogen): initial weight loss due to thermal degradation commences at ~ 454°C.

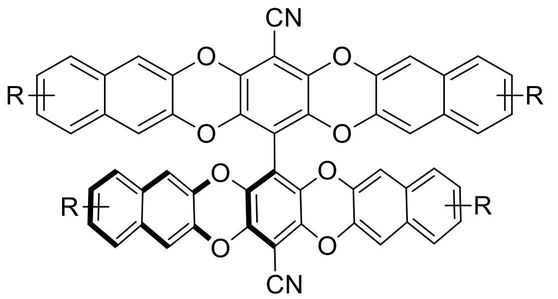

17 (R = H)
18 (R = *t*Bu)
19 (R = Ad)

17

4,4'-Dicyano-2,2',3,3',5,5',6,6'-octafluorobiphenyl (**1**) (0.208 g, 0.597 mmol), naphthalene-2,3-diol (**4a**) (0.402 g, 2.51 mmol) and potassium carbonate (0.825 g, 5.97 mmol) were reacted together in anhydrous DMF (10 ml) in accordance with general procedure 1. Purification was achieved *via* column chromatography (CH_2Cl_2/hexane, 3/2, R_f = 0.4) to give **17** (0.219 g, 44%) as a yellow powder (mp > 300°C); IR (CH_2Cl_2 film) 3056, 2921, 2850, 2236, 1516, 1475, 1447, 1430, 1396, 1362, 1264, 1169 cm^{-1}; ^1H NMR (400 MHz, C_4D_8O) δ 7.73 (d, J = 8.1 Hz, 4H, Ar*H*), 7.62 (s, 4H, Ar*H*), 7.49 (d, J = 8.1 Hz, 4H, Ar*H*), 7.35 – 7.31 (m, 4H, Ar*H*), 7.27 – 7.24 (m, 8H, Ar*H*), ^{13}C NMR (125 MHz, C_4D_8O) δ 132.5, 132.3, 128.1, 128.0, 126.9, 117.0, 114.0, 113.9, 111.2 (multiple carbons missing); ^{13}C NMR (101 MHz, solid state) 139.8, 138.1, 134.9, 130.4, 125.9, 115.3, 113.5, 111.2 (multiple carbons missing); LRMS (MALDI, *m/z*) calc. for $C_{54}H_{24}N_2O_8$: 828.15 (M^+), found 828.36. GPC analysis ($CHCl_3$) M_n = 876, M_w = 955 g mol^{-1} relative to polystyrene, M_w/M_n = 1.029; BET surface area = 25 m^2 g^{-1}; total pore volume = 0.054 cm^3 g^{-1} at p/p^o = 0.98; Crystallography data (THF): Triclinic, space group = $P\bar{1}$, a = 14.554(8) Å, b = 14.969(7) Å, c = 24.018(12) Å, α = 76.716(16), β = 80.534(13), γ = 85.658(16), V = 5019.26 $Å^3$, Z = 4, R_1 = 14.14. (CCDC 1406072).

18

4,4'-Dicyano-2,2',3,3',5,5',6,6'-octafluorobiphenyl (**1**) (0.179 g, 0.514 mmol), 6-*tert*-butylnaphthalene-2,3-diol (**4b**) (0.462 g, 2.14 mmol) and potassium carbonate (0.709 g, 5.13 mmol) were reacted together in anhydrous DMF (10 ml) in accordance with general procedure 1. Purification was achieved *via* column chromatography (hexane/CH_2Cl_2, 3/2, R_f = 0.4) to give **18** (mixture of regioisomers) (0.346 g, 64%) as a yellow powder (mp > 300°C); IR (CH_2Cl_2 film) 2961, 2907, 2869, 2238, 1514, 1434, 1250, 1002 cm^{-1}; ^1H NMR (400 MHz, $CDCl_3$) δ 7.65 – 7.61 (m, 4H, Ar*H*), 7.46 – 7.24 (m, 12H, Ar*H*), 7.13 – 6.98 (m, 4H, Ar*H*), 1.381 – 1.378 (m, 18H, *t*Bu*H*), 1.28 – 1.26 (m, 18H, *t*Bu*H*); ^{13}C NMR (125 MHz, $CDCl_3$) δ 149.08, 149.06, 149.01, 148.99, 148.95, 140.32, 140.29, 140.23, 140.21, 140.18, 140.17, 140.12, 140.08, 140.05, 140.0, 139.93, 139.87, 139.82, 139.80, 139.76, 139.74, 139.67, 139.64, 139.60, 139.55, 139.50, 139.49, 139.46, 139.4, 139.34, 139.28, 135.9, 135.84, 135.80, 135.76, 135.74, 135.69, 135.67, 135.6, 131.3, 131.20, 131.17, 131.1, 129.29, 129.26, 129.2, 127.0, 126.9, 125.1, 122.5, 122.4, 113.50, 113.47, 113.39, 113.37, 113.3, 113.0, 112.9, 112.84, 112.79, 112.77, 112.75, 112.7, 111.6, 110.7, 91.74, 91.70, 35.0, 34.9, 31.4, 31.2 (extra carbons due to regioisomeric nature); LRMS (EI^+, *m/z*) calc. for $C_{70}H_{56}N_2O_8$: 1052.40 (M^+), found 1052.44; GPC analysis ($CHCl_3$) M_n = 1063, M_w = 1112 g mol^{-1} relative to polystyrene, M_w/M_n = 1.046; BET surface area = 260 m^2 g^{-1}; total pore volume = 0.334 cm^3 g^{-1} at p/p^o = 0.98.

19

4,4'-Dicyano-2,2',3,3',5,5',6,6'-octafluorobiphenyl (**1**) (0.161 g, 0.462 mmol), 6-(1-adamantyl)naphthalene-2,3-diol (**4c**) (0.573 g, 1.94 mmol) and potassium carbonate (0.640 g, 4.63 mmol) were reacted together in anhydrous DMF (10 ml) in accordance with general procedure 1. Purification was achieved *via* column chromatography (hexane/CH_2Cl_2, 3/2, R_f = 0.4) to give **19** (mixture of regioisomers) (0.245 g, 39%) as a yellow powder (mp > 300°C); IR (CH_2Cl_2 film) 2903, 2848, 2238, 1514, 1434, 1266, 1244, 1002 cm^{-1}; ^1H NMR (500

MHz, $CDCl_3$) δ 7.63 – 7.55 (m, 4H, Ar*H*), 7.45 – 7.22 (m, 12H, Ar*H*), 7.10 – 7.00 (m, 4H, Ar*H*), 2.12 – 1.59 (m, 60H, Ad*H*); ^{13}C NMR (125 MHz, $CDCl_3$) δ 149.09, 149.08, 149.02, 149.00, 139.93, 139.89, 139.87, 139.81, 139.80, 139.77, 139.7, 139.63, 139.62, 139.59, 139.56, 139.52, 139.47, 139.43, 139.40, 139.36, 139.31, 139.28, 139.26, 139.21, 139.18, 139.1, 139.0, 135.7, 135.6, 135.51, 135.46, 135.4, 131.2, 131.12, 131.10, 131.06, 131.01, 131.00, 129.2, 129.1, 126.8, 124.32, 124.25, 122.2, 113.2, 113.13, 113.05, 112.7, 112.6, 112.4, 111.33, 111.30, 110.6, 91.5, 43.0, 42.9, 36.8, 36.7, 36.34, 36.26, 28.9, 28.8 (extra carbons due to regioisomeric nature); LRMS (MALDI, *m/z*) calc. for $C_{94}H_{80}N_2O_8$: 1365.59 (M^+), found 1365.59; GPC analysis ($CHCl_3$) M_n = 1134, M_w = 1195 g mol^{-1} relative to polystyrene, M_w/M_n = 1.050; BET surface area = 132 m^2 g^{-1}; total pore volume = 0.247 cm^3 g^{-1} at p/p^o = 0.98.

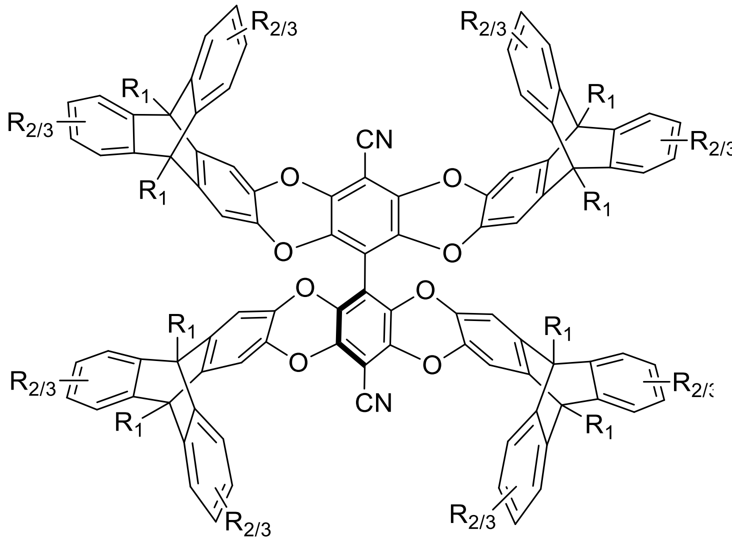

23 (OMIM-1) (R_1, R_2, R_3 = H)
24 (OMIM-8) (R_1 = Me, R_2, R_3 = H)
25 (OMIM-4) (R_1, R_2 = H, R_3 = *t*Bu)
26 (OMIM-9) (R_1 = Me, R_2 = H, R_3 = *t*Bu)
27 (OMIM-5) (R_1 = H, R_2, R_3 = TMB)

24 (**OMIM-8**)

4,4'-Dicyano-2,2',3,3',5,5',6,6'-octafluorobiphenyl (**1**) (0.153 g, 0.439 mmol), 9,10-dimethyltriptycene-2,3-diol (**5b**) (0.622 g, 1.98 mmol) and potassium carbonate (0.610 g, 4.41 mmol) were reacted together in anhydrous DMF (10 ml) in accordance with general procedure 1. Purification was achieved *via* column chromatography (CH_2Cl_2/hexane/toluene, 2/1/1, R_f = 0.2) to give **OMIM-8** (0.322 g, 51%) as a yellow powder (mp > 300°C); IR (CH_2Cl_2 film) 3062, 2974, 2238, 1612, 1446, 1290, 1169, 1006 cm^{-1}; ^1H NMR (500 MHz, $CDCl_3$) δ 7.33 (d, *J* = 7.2 Hz, 8H, Ar*H*), 7.20 (d, *J* = 7.2 Hz, 8H, Ar*H*), 7.05 – 6.98 (m, 20H, Ar*H*), 6.63 (s, 4H, Ar*H*), 2.35 (s, 12H, CH_3), 2.18 (s, 12H, CH_3); ^{13}C NMR (125 MHz, $CDCl_3$) δ 147.68, 147.66, 145.8, 145.6, 139.5, 137.4, 137.0, 135.7, 125.1, 120.7, 120.6, 111.2, 110.9, 110.5, 110.2, 91.0, 48.36, 48.35, 13.7, 13.5; LRMS (MALDI, *m/z*) calc. for $C_{102}H_{64}N_2O_8$: 1445.47 (M^+), found 1445.81; GPC analysis ($CHCl_3$) M_n = 1216, M_w = 1245 g mol^{-1} relative to polystyrene, M_w/M_n = 1.023; BET surface area = 462 m^2 g^{-1}; total pore volume = 0.33 cm^3 g^{-1} at p/p^o = 0.98; Crystallography data (chloroform/methanol): Monoclinic, space group: $P2_1/n$, *a* = 20.0299(3) Å, *b* = 24.0548(5) Å, *c* = 21.1645(3) Å, β = 91.2530(10), *V* = 10194.9 $Å^3$, *Z* = 4, R_1 = 11.52. (CCDC 1406070).

26 (**OMIM-9**)

4,4'-Dicyano-2,2',3,3',5,5',6,6'-octafluorobiphenyl (**1**) (0.057 g, 0.164 mmol), 7,14-di-*tert*-butyl-9,10-dimethyltriptycene-2,3-diol (**5d**) (0.294 g, 0.689 mmol) and potassium carbonate (0.227 g, 1.64 mmol) were reacted together in anhydrous DMF (10 ml) in accordance with general procedure 1. Purification was achieved *via* column chromatography (hexane/toluene/CH_2Cl_2, 3/1/1, R_f = 0.25) to give **OMIM-9** (mixture of

regioisomers) (0.146 g, 47%) as a yellow powder (mp > 300°C); IR (CH_2Cl_2 film) 2966, 2869, 2239, 1612, 1443, 1290, 1001 cm^{-1}; ^1H NMR (500 MHz, $CDCl_3$) δ 7.31 (s, 4H, Ar*H*), 7.24 – 7.19 (m, 8H, Ar*H*), 7.09 – 7.06 (m, 4H, Ar*H*), 7.03 – 6.99 (m, 4H, Ar*H*), 6.97 – 6.89 (m, 8H, Ar*H*), 2.33 (s, 12H, CH_3), 2.18 (s, 12H, CH_3), 1.25 – 1.15 (m, 72H, *t*Bu*H*); ^{13}C NMR (125 MHz, $CDCl_3$) δ 147.9, 147.43, 147.36, 146.2, 146.0, 145.9, 144.9, 144.82, 144.79, 139.53, 139.47, 137.32, 137.27, 137.25, 137.2, 136.9, 136.84, 136.78, 136.7, 135.8, 135.7, 135.6, 121.63, 121.58, 120.1, 120.0, 117.9, 117.73, 117.67, 111.21, 111.16, 111.0, 110.52, 110.45, 110.4, 110.3, 110.0, 90.9, 48.23, 48.19, 34.82, 34.80, 34.77, 34.7, 31.71, 31.69, 31.67, 13.8, 13.5 (extra carbons due to regioisomeric nature); LRMS (MALDI, *m/z*) calc. for $C_{134}H_{128}N_2O_8$: 1893.97 (M^+), found 1893.63; GPC analysis ($CHCl_3$) M_n = 1645, M_w = 1688 g mol^{-1} relative to polystyrene, M_w/M_n = 1.026; BET surface area = 599 m^2 g^{-1}; total pore volume = 0.42 cm^3 g^{-1} at p/p^o = 0.98.

34 (**OMIM-2**)

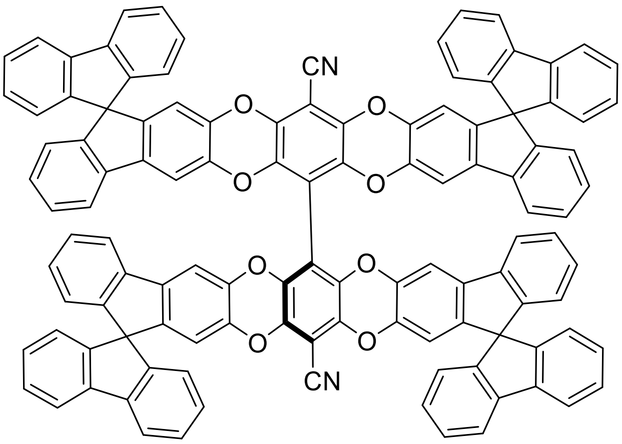

4,4'-Dicyano-2,2',3,3',5,5',6,6'-octafluorobiphenyl (**1**) (0.091 g, 0.26 mmol**)**, 2,3-dihydroxy-9,9'-spirobisfluorene (**7**) (0.40 g, 1.15 mmol) and potassium carbonate (0.58 g 4.20 mmol) were reacted together in anhydrous DMF (15 ml) in accordance with general procedure 1. Purification was achieved *via* column chromatography (hexane/ethyl acetate, 1/4) to give **OMIM-2** (mixture of regioisomers) (0.37 g, 90%) as a fluorescent yellow solid (mp > 300°C); IR (CH_2Cl_2 film) 3059, 2918, 2237, 1604, 1442, 1352, 1277, 1168, 1007 cm^{-1}; ^1H NMR (400 MHz, $CDCl_3$) δ 7.79 (m, 12H, Ar*H*), 7.35, (m, 14H, Ar*H*), 7.07 (m, 14H, Ar*H*), 6.66 (m, 12H, Ar*H*), 6.47-6.03 (cluster of peaks, 4H, Ar*H*); ^{13}C NMR (100 MHz, $CDCl_3$) (clusters of peaks) δ 148.8, 148.7, 147.9, 147.8, 147.7, 145.2, 144.9, 144.6, 141.6, 141.5, 141.4, 140.3, 140.2, 138.9, 138.7, 138.4, 138.3, 138.2, 135.1, 134.9, 134.8, 128.0, 127.9, 127.8, 127.7, 127.6, 124.0, 123.9, 123.8, 123.6, 120.3, 120.2, 120.1, 120.0, 119.7, 119.6, 112.5, 112.4, 112.3, 108.3, 108.2, 65.4; LRMS (MALDI, *m/z*) calc. for $C_{114}H_{56}N_2O_8$: 1581.41 (M^+), found 1581.08; elemental analysis calc. (%) for $C_{114}H_{56}N_2O_8$: C 86.57, H 3.57, N 1.77, found C 85.27, H 3.45, N 1.66; GPC analysis ($CHCl_3$) M_n = 1322, M_w = 1359 g mol^{-1} relative to polystyrene, M_w/M_n 1.028; BET surface area = 333 m^2 g^{-1}; total pore volume = 0.28 cm^3 g^{-1} at p/p^o = 0.98; TGA analysis (N_2): Initial weight loss due to thermal degradation commences at ~ 523°C.

35 (**OMIM-3**)

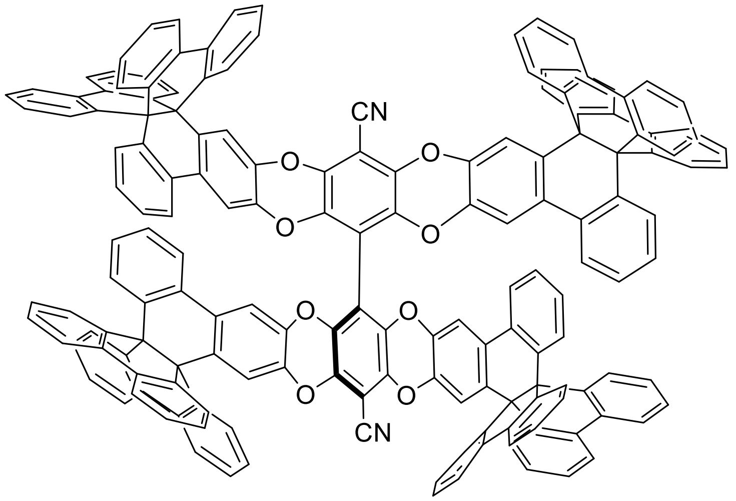

4,4'-Dicyano-2,2',3,3',5,5',6,6'-octafluorobiphenyl (**1**) (0.077 g, 0.22 mmol), dihydroxypropellane (**8**) (0.61 g, 1.20 mmol) and potassium carbonate (0.50 g 3.60 mmol) were reacted together in anhydrous DMF (20 ml) in accordance with general procedure 1. Purification was achieved *via* column chromatography (CH_2Cl_2) to give **OMIM-3** (mixture of regioisomers) (0.29 g, 56%) as a yellow solid (mp > 300°C); IR (nujol) 3064, 3027, 2239, 1917, 1600, 1573, 1443, 1320, 1279, 1208, 1019 cm^{-1}; ^1H NMR (400 MHz, $CDCl_3$) δ 7.59 (br m, 16H, Ar*H*), 7.46 (br m, 6H, Ar*H*), 7.17 (br m, 20H, Ar*H*), 7.01 (br m, 20H, Ar*H*), 6.60 (br m, 20H, Ar*H*), 6.28 (br m, 4H, Ar*H*); ^{13}C NMR (125 MHz, $CDCl_3$) (clusters of peaks) δ 139.2, 139.1, 139.1, 139.1, 139.0, 138.9, 134.8, 134.7, 134.7, 134.7, 129.2, 129.2, 129.0, 129.0, 129.0, 128.9, 128.9, 127.7, 127.6, 127.4, 127.3, 123.8, 123.8, 123.7, 53.3, 53.3, 53.3, 53.1, 53.0, 52.8, 52.6, 52.6, 52.5; LRMS (MALDI, *m/z*) calc. for $C_{166}H_{88}N_2O_8$: 2236.65 (M^+), found 2238.02 (MH^+); Elemental analysis calc. for $C_{166}H_{88}N_2O_8$: C 89.07, H 3.96, N 1.25, found C 87.47, H 3.89, N 1.20; GPC analysis ($CHCl_3$): *M*n = 1624, *M*w = 1676 g mol^{-1} relative to polystyrene, *M*w/*M*n 1.032; BET surface area = 593 m^2 g^{-1}; total pore volume = 0.45 cm^3 g^{-1} at p/p^o = 0.98; TGA analysis (nitrogen): Initial weight loss due to thermal degradation commences at ~ 556°C.

36 (**OMIM-15**)

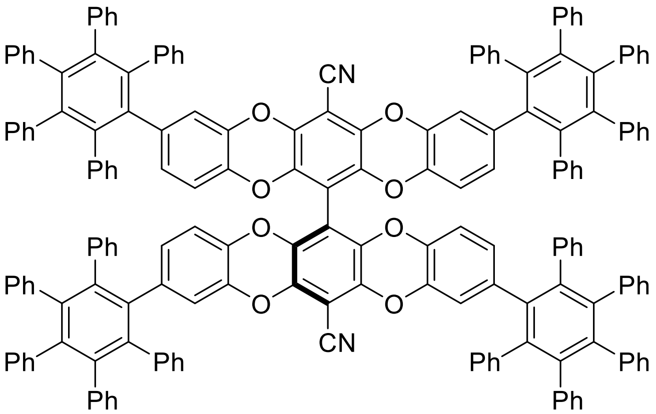

4,4'-Dicyano-2,2',3,3',5,5',6,6'-octafluorobiphenyl (**1**) (0.334 g, 0.56 mmol), 1-(1,2-dihydroxyphenyl)-2,3,4,5,6-pentaphenylbenzene (**9**) (0.046 g, 0.13 mmol) and potassium carbonate (1.94 g, 14.1 mmol) were reacted together in anhydrous DMF (5 ml) in accordance with general procedure 1. Purification was achieved *via* column chromatography (hexane/ethyl acetate, 3/2) to give **OMIM-15** (mixture of regioisomers) (0.252

g, 73%) as a yellow powder (mp > 300˚C); IR (nujol) 2339, 1303, 1055, 1026 cm^{-1}; ^{1}H NMR (400 MHz, $CDCl_3$) δ 6.86 (m, 100H, Ar*H*) 6.26 (m, 12H, Ar*H*); ^{13}C NMR (100 MHz, $CDCl_3$) δ 141.2, 141.0, 140.8, 140.7, 140.5, 139.1, 138.7, 138.6, 138.0, 131.7, 131.6, 129.9, 127.0, 125.8, 125.7, 124.3, 99.1; LRMS (MALDI, *m/z*): calc. for $C_{182}H_{112}N_2O_8$: 2454.85 (M^+), found 2454.90; BET surface area = 407 m^2 g^{-1}, total pore volume = 0.46 cm^3 g^{-1} at p/p^o = 0.98.

Terphenyl adducts/OMIMs

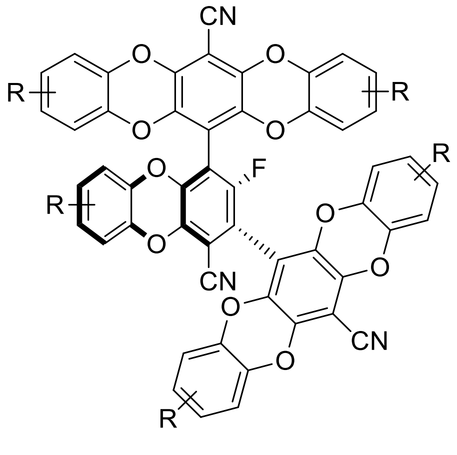

14 (R = H)
15 (R = *t*Bu)

14
4,4′,4′′-Tricyano-2,2',2'',3,3'',5,5',5'',6,6',6''-undecafluoro-[1,1':3',1''-terphenyl] (**2**) (0.146 g, 0.290 mmol), catechol (**3a**) (0.176 g, 1.60 mmol) and potassium carbonate (0.500 g, 3.62 mmol) were reacted together in anhydrous DMF (10 ml) in accordance with general procedure 1. Purification was achieved *via* column chromatography (CH_2Cl_2/hexane, 9/1, R_f = 0.35) to give **14** (0.215 g, 87%) as a yellow powder (mp > 300°C); IR (CH_2Cl_2 film) 2237, 1607, 1495, 1450, 1274, 1254, 1033 cm^{-1}; ^{1}H NMR (400 MHz, $CDCl_3$) δ 7.07 – 6.86 (m, 15H, Ar*H*), 6.79 – 6.76 (m, 3H, Ar*H*), 6.72 (dd *J* = 8.1, 1.3 Hz, 2H, Ar*H*); ^{13}C NMR (125 MHz, $CDCl_3$) δ 142.53, 142.48, 140.9, 140.81, 140.76, 140.7, 140.6, 140.1, 140.0, 136.2, 136.1, 125.8, 125.6, 125.40, 125.38, 117.2, 117.1, 117.1, 116.9, 112.5, 112.4, 110.37, 110.35, 110.3, 103.5, 103.4, 92.3, 92.1 (^{19}F-^{13}C coupling not assigned); ^{19}F NMR (282 MHz, $CDCl_3$) δ -112.2 (s, 1F, Ar*F*); LRMS (EI^+, *m/z*) calc. for $C_{51}H_{20}FN_3O_{10}$: 853.11 (M^+), found 853.15; GPC analysis ($CHCl_3$) M_n = 822, M_w = 862 g mol^{-1} relative to polystyrene, M_w/M_n = 1.048; BET surface area = 13 m^2 g^{-1}; total pore volume = 0.05 cm^3 g^{-1} at p/p^o = 0.98. Crystallography data (chloroform/methanol): Triclinic, space group: $P\bar{1}$, *a* = 14.680(7) Å, *b* = 15.072(7) Å, *c* = 23.438(12) Å, α = 101.876(9), β = 102.840(5), γ = 94.462(4), *V* = 4906.28 Å^3, *Z* = 4, R_1 = 17.2. (CCDC 1406073).

15
4,4′,4′′-Tricyano-2,2',2'',3,3'',5,5',5'',6,6',6''-undecafluoro-[1,1':3',1''-terphenyl] (**2**) (0.124 g, 0.246 mmol), 4-*tert*-butylcatechol (**3b**) (0.245 g, 1.47 mmol) and potassium carbonate (0.410 g, 2.97 mmol) were reacted together in anhydrous DMF (10 ml) in accordance with general procedure 1. Purification was achieved using column chromatography (CH_2Cl_2/hexane, 1/1, R_f = 0.45) to give **15** (mixture of regioisomers) (0.180 g, 64%) as a yellow powder (mp > 300°C); IR (CH_2Cl_2 film) 2964, 2906, 2870, 2237, 1604, 1515, 1456, 1273, 1122, 1010 cm^{-1}; ^{1}H NMR (500 MHz, $CDCl_3$) δ 7.13 – 6.62 (m, 15H, Ar*H*), 1.27 – 1.10 (m, 45H, *t*Bu*H*); ^{13}C NMR (125 MHz, $CDCl_3$) δ 149.2, 149.11, 149.10, 149.05, 141.2, 140.4, 140.3, 140.24, 140.19, 140.14, 140.10, 140.05, 140.0, 138.52, 138.45, 138.41, 138.36, 138.3, 138.23, 138.19, 136.3, 136.2, 136.1, 122.3, 122.1, 121.93, 121.87, 116.5, 116.43, 116.37, 116.2, 116.0, 114.4, 114.0, 113.09, 113.85, 111.9, 110.6, 92.0, 91.8, 34.9, 34.8, 34.69, 34.68, 34.65, 31.41, 31.36, 31.3 (^{19}F-^{13}C coupling not assigned, extra carbons due to regioisomeric nature); ^{19}F NMR (282 MHz, $CDCl_3$) δ -111.8 – -112.6 (cluster of singlets, 1F, Ar*F*); LRMS

(MALDI, *m/z*) calc. for $C_{71}H_{60}FN_3O_{10}$: 1133.43 (M^+), found 1133.83; GPC analysis ($CHCl_3$) M_n = 1106, M_w = 1131 g mol^{-1} relative to polystyrene, M_w/M_n = 1.023; BET surface area = 7 m^2 g^{-1}; total pore volume = 0.01 cm^3 g^{-1} at p/p^o = 0.98.

16

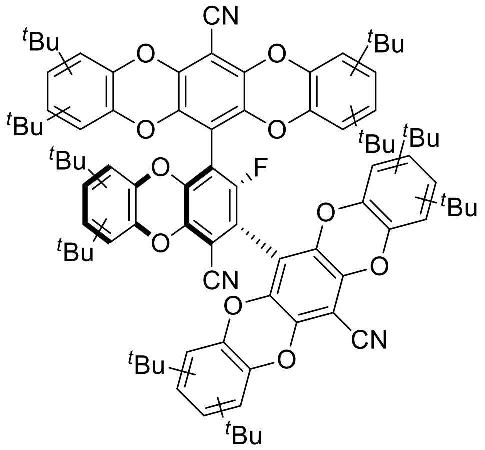

4,4',4''-Tricyano-2,2',2'',3,3'',5,5',5'',6,6',6''-undecafluoro-[1,1':3',1''-terphenyl] (**2**) (0.106 g, 0.211 mmol), 3,5-di-*tert*-butylcatechol (**3c**) (0.280 g, 1.26 mmol) and potassium carbonate (0.350 g, 2.53 mmol) were reacted together in anhydrous DMF (10 ml) in accordance with general procedure 1. Purification was achieved *via* column chromatography (hexane/CH_2Cl_2/toluene, 3/1/1, R_f = 0.4) to give **16** (mixture of regioisomers) (0.263 g, 88%) as a yellow powder (mp > 300°C); IR (CH_2Cl_2 film) 2962, 2871, 2237, 1446, 1412, 1305, 1261, 1230, 1037, 1019 cm^{-1}; ^{1}H NMR (400 MHz, $CDCl_3$) δ 7.00 – 6.22 (m, 10H, Ar*H*), 1.54 – 0.88 (m, 90H, *t*Bu*H*); ^{13}C NMR (125 MHz, $CDCl_3$) δ 148.0, 147.7 – 147.4 (m), 140.5 – 139.6 (m), 138.1 – 137.8 (m), 137.5 – 137.1 (m), 120.3 – 119.4 (m), 113.0 – 111.9 (m), 111.2, 110.6, 91.6, 91.4, 35.4 (m), 35.0 – 34.7 (m), 34.41, 34.36, 31.4 – 31.2 (m), 31.02, 30.98, 30.9, 30.4, 30.2, 30.1 – 29.5 (m) (^{19}F-^{13}C coupling not assigned, extra carbons due to regioisomeric nature); ^{19}F NMR (282 MHz, $CDCl_3$) δ -106.2 – -112.9 (cluster of singlets, 1F, Ar*F*); LRMS (MALDI, *m/z*) calc. for $C_{91}H_{100}FN_3O_{10}$: 1414.74 (M^+), found 1414.99; GPC analysis ($CHCl_3$) M_n = 1378, M_w = 1421 g mol^{-1} relative to polystyrene, M_w/M_n = 1.031; BET surface area = 102 m^2 g^{-1}; total pore volume = 0.37 cm^3 g^{-1} at p/p^o = 0.98.

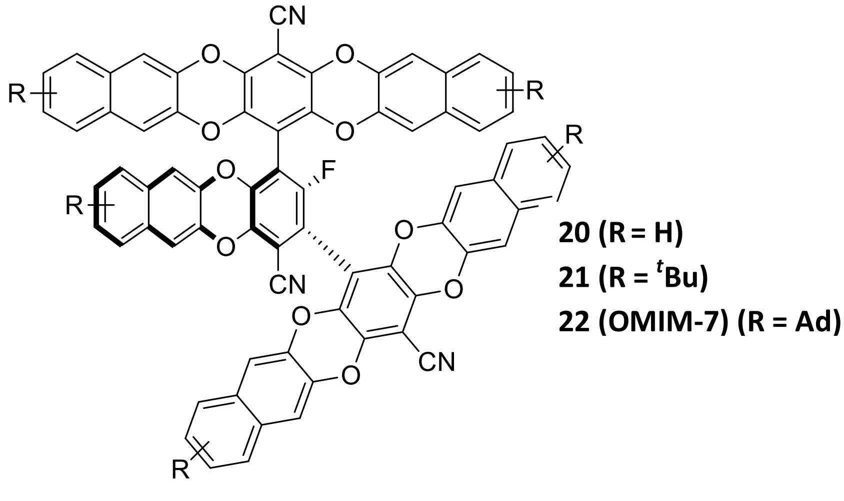

20 (R = H)
21 (R = ^{t}Bu)
22 (OMIM-7) (R = Ad)

20

4,4',4''-Tricyano-2,2',2'',3,3'',5,5',5'',6,6',6''-undecafluoro-[1,1':3',1''-terphenyl] (**2**) (0.178 g, 0.354 mmol),

naphthalene-2,3-diol (**4a**) (0.310 g, 1.94 mmol) and potassium carbonate (0.585 g, 4.23 mmol) were reacted together in anhydrous DMF (10 ml) in accordance with general procedure 1. Purification was achieved *via* column chromatography (CH_2Cl_2/hexane/toluene, 7/2/1, R_f = 0.3) to give **20** (0.049 g, 13%) as a yellow powder (mp > 300°C); IR (CH_2Cl_2 film) 2920, 2850, 2236, 1520, 1451, 1263, 1170, 1013 cm^{-1}; ^1H NMR (400 MHz, C_4D_8O) δ 7.80 – 7.72 (m, 6H, Ar*H*), 7.63 – 7.56 (m, 5H, Ar*H*), 7.46 – 7.21 (m, 19H, Ar*H*); ^{19}F NMR (282 MHz, $CDCl_3$) δ -111.72 (s, 1F, Ar*F*); LRMS (MALDI, *m/z*) calc. for $C_{71}H_{30}FN_3O_{10}$: 1103.19 (M^+), found 1103.30; GPC analysis ($CHCl_3$) M_n = 871, M_w = 889 g mol^{-1} relative to polystyrene, M_w/M_n = 1.021; BET surface area = 29 $m^2\,g^{-1}$; total pore volume = 0.11 $cm^3\,g^{-1}$ at p/p^o = 0.98.

21

4,4',4''-Tricyano-2,2',2'',3,3'',5,5',5'',6,6',6''-undecafluoro-[1,1':3',1''-terphenyl] (**2**) (0.136 g, 0.270 mmol), 6-*tert*-butylnaphthalene-2,3-diol (**4b**) (0.320 g, 1.49 mmol) and potassium carbonate (0.747 g, 5.41 mmol) were reacted together in anhydrous DMF (10 ml) in accordance with general procedure 1. Purification was achieved *via* column chromatography (CH_2Cl_2/hexane/toluene, 4/5/1, R_f = 0.3) to give **21** (mixture of regioisomers) (0.162 g, 43%) as a yellow powder (mp > 300°C); IR (CH_2Cl_2 film) 2961, 2869, 2237, 1514, 1443, 1248, 1012 cm^{-1}; ^1H NMR (500 MHz, $CDCl_3$) δ 7.69 – 7.09 (m, 25H, Ar*H*), 1.38 – 1.11 (m, 45H, *t*Bu*H*); ^{13}C NMR (125 MHz, $CDCl_3$) δ 149.6, 149.3 – 149.2 (m), 140.1 – 140.0 (m), 139.9 – 139.5 (m), 135.9 – 135.7 (m), 132.8, 132.5, 131.4 – 131.2 (m), 129.4 – 129.2 (m), 127.2 – 126.9 (m), 126.2, 125.6 – 125.5 (m), 122.7 – 122.5 (m), 122.3, 113.6 – 112.6 (m), 110.6 – 110.5 (m), 110.2, 92.3, 92.2, 35.1, 35.0, 34.68, 34.84, 31.34, 31.26, 31.22, 31.16 (^{19}F-^{13}C coupling not assigned, extra carbons due to regioisomeric nature); ^{19}F NMR (282 MHz, $CDCl_3$) δ -111.4 – -111.8 (cluster of singlets, 1F, Ar*F*); LRMS (MALDI, *m/z*) calc. for $C_{91}H_{70}FN_3O_{10}$: 1384.51 (M^+), found 1384.41; GPC analysis ($CHCl_3$) M_n = 1453, M_w = 1485 g mol^{-1} relative to polystyrene, M_w/M_n = 1.022; BET surface area = 259 $m^2\,g^{-1}$; total pore volume = 0.35 $cm^3\,g^{-1}$ at p/p^o = 0.98.

22 (**OMIM-7**)

4,4',4''-Tricyano-2,2',2'',3,3'',5,5',5'',6,6',6''-undecafluoro-[1,1':3',1''-terphenyl] (**2**) (0.156 g, 0.310 mmol), 6-(1-adamantyl)naphthalene-2,3-diol (**4c**) (0.480 g, 1.63 mmol) and potassium carbonate (0.700 g, 5.07 mmol) were reacted together in anhydrous DMF (10 ml) in accordance with general procedure 1. Purification was achieved *via* column chromatography (CH_2Cl_2/hexane/toluene, 7/2/1, R_f = 0.3) to give **OMIM-7** (mixture of regioisomers) (0.531 g, 97%) as a yellow powder (mp > 300°C); IR (CH_2Cl_2 film) 2903, 2848, 2351, 1514, 1446, 1265, 1243, 1012 cm^{-1}; ^1H NMR (500 MHz, $CDCl_3$) δ 7.68 – 7.10 (m, 25H, Ar*H*), 2.10 – 1.68 (m, 75H, Ad*H*); ^{13}C NMR (125 MHz, $CDCl_3$) δ 149.6, 149.5 – 149.3 (m), 142.4 – 142.1 (m), 140.1 – 140.0 (m), 139.8 – 139.3 (m), 135.9 – 135.6 (m), 131.5 – 131.3 (m), 129.6 – 129.4 (m), 127.1 – 126.9 (m), 125.0 – 124.6 (m), 122.6 – 122.3 (m), 113.6 – 113.4 (m), 113.0 – 112.6 (m), 110.6 – 110.4 (m), 92.3 – 92.1 (m), 42.2 – 43.0 (m), 37.0, 36.9, 36.6 – 36.4 (m), 29.13 – 29.06 (m) (^{19}F-^{13}C coupling not assigned, extra carbons due to regioisomeric nature); ^{19}F NMR (282 MHz, $CDCl_3$) δ -111.1 – -111.5 (cluster of singlets, 1F, Ar*F*); LRMS (MALDI, *m/z*) calc. for $C_{121}H_{100}FN_3O_{10}$: 1775.10 (M^+), found 1777.57 (MH^+); GPC analysis ($CHCl_3$) M_n = 1403, M_w = 1543 g mol^{-1} relative to polystyrene, M_w/M_n = 1.100; BET surface area = 347 $m^2\,g^{-1}$; total pore volume = 0.41 $cm^3\,g^{-1}$ at p/p^o = 0.98.

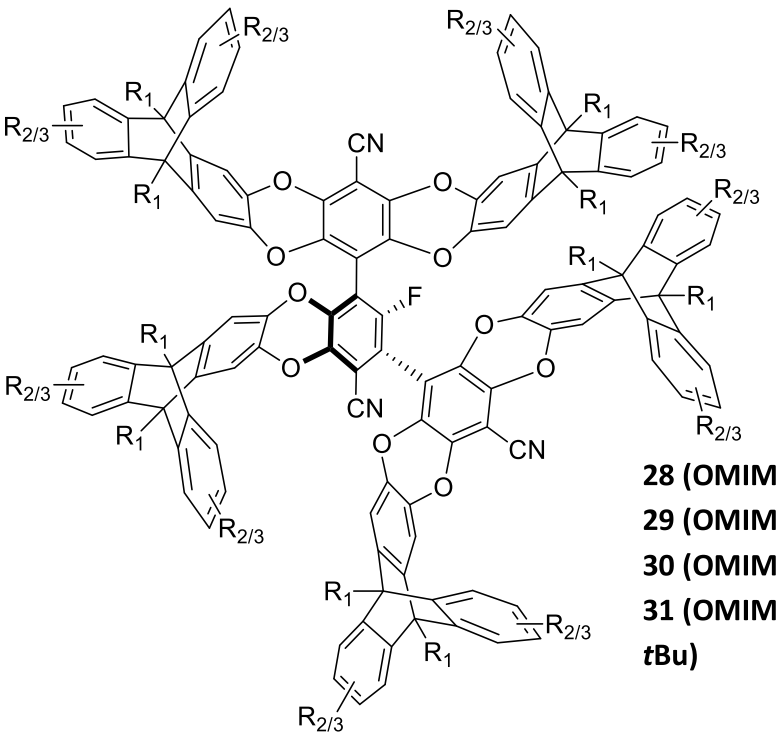

28 (OMIM-10) (R_1, R_2, R_3 = H)
29 (OMIM-11) (R_1 = Me, R_2, R_3 = H)
30 (OMIM-12) (R_1, R_2 = H, R_3 = *t*Bu)
31 (OMIM-13) (R_1 = Me, R_2 = H, R_3 = *t*Bu)

28 (**OMIM-10**)

4,4',4''-Tricyano-2,2',2'',3,3'',5,5',5'',6,6',6''-undecafluoro-[1,1':3',1''-terphenyl] (**2**) (0.060 g, 0.119 mmol), triptycene-2,3-diol (**5a**) (0.200 g, 0.698 mmol) and potassium carbonate (0.200 g, 1.45 mmol) were reacted together in anhydrous DMF (10 ml) in accordance with general procedure 1. Purification was achieved *via* column chromatography (CH_2Cl_2/hexane/toluene, 3/1/1, R_f = 0.35) to give **OMIM-10** (0.062 g, 30%) as a yellow powder (mp > 300°C); IR (CH_2Cl_2 film) 3065, 2956, 2236, 1489, 1445, 1285, 1139, 1009 cm^{-1}; ^1H NMR (400 MHz, $CDCl_3$) δ 7.41 (d, *J* = 7.0 Hz, 2H, Ar*H*), 7.37 – 7.34 (m, 8H, Ar*H*), 7.26 – 7.14 (m, 10H, Ar*H*), 7.07 – 6.91 (m, 25H, Ar*H*), 6.84 (s, 1H, Ar*H*), 6.78 (s, 2H, Ar*H*), 6.72 (s, 2H, Ar*H*), 5.38 (s, 1H, C*H*), 5.32 (s, 2H, C*H*), 5.31 (s, 2H, C*H*), 5.18 (s, 5H, C*H*); ^{13}C NMR (125 MHz, $CDCl_3$) δ 144.9, 144.8 – 144.6 (m), 143.02, 142.96, 142.7, 142.6 – 142.5 (m), 140.0, 139.8, 138.8, 137.7, 137.6, 137.5, 137.40, 137.36, 136.0, 135.9, 125.8 – 125.6 (m), 123.9 – 123.7 (m), 113.2, 113.04, 112.99, 112.8, 112.0, 110.0, 91.9, 91.7, 53.6, 53.5, 53.4 – 53.2 (m) (^{19}F-^{13}C coupling not assigned); ^{19}F NMR (282 MHz, $CDCl_3$) δ -111.7 (s, 1F, Ar*F*); LRMS (MALDI, *m/z*) calc. for $C_{121}H_{60}FN_3O_{10}$: 1734.43 (M^+), found 1734.99; GPC analysis ($CHCl_3$) M_n = 1493, M_w = 1533 g mol^{-1} relative to polystyrene, M_w/M_n = 1.027; BET surface area = 423 $m^2\,g^{-1}$; total pore volume = 0.44 $cm^3\,g^{-1}$ at p/p^o = 0.98.

29 (**OMIM-11**)

4,4',4''-Tricyano-2,2',2'',3,3'',5,5',5'',6,6',6''-undecafluoro-[1,1':3',1''-terphenyl] (**2**) (0.101 g, 0.201 mmol), 9,10-dimethyltriptycene-2,3-diol (**5b**) (0.348 g, 1.11 mmol) and potassium carbonate (0.306 g, 2.21 mmol) were reacted together in anhydrous DMF (10 ml) in accordance with general procedure 1. Purification was achieved *via* column chromatography (CH_2Cl_2/hexane/toluene, 2/2/1, R_f = 0.3) to give **OMIM-11** (0.296 g, 79%) as a yellow powder (mp > 300°C); IR (CH_2Cl_2 film) 3054, 2977, 2920, 2237, 1612, 1446, 1291, 1264, 1169, 1011 cm^{-1}; ^1H NMR (400 MHz, $CDCl_3$) δ 7.36 (d, *J* = 7.3 Hz, 2H, Ar*H*), 7.32 – 7.28 (m, 8H, Ar*H*), 7.24 – 7.23 (m, 4H, Ar*H*), 7.19 – 7.14 (m, 4H, Ar*H*), 7.08 (d, *J* = 8.6 Hz, 4H, Ar*H*), 7.05 – 6.97 (m, 24H, Ar*H*), 6.75 (s, 1H, Ar*H*), 6.64 (s, 4H, Ar*H*), 2.39 – 2.32 (m, 15H, C*H*$_3$), 2.22 (s, 3H, C*H*$_3$), 1.99 (s, 6H, C*H*$_3$), 1.91 (s, 6H, C*H*$_3$); ^{13}C NMR (125 MHz, $CDCl_3$) δ 147.9, 147.8, 147.7 – 147.5 (m), 146.5, 146.4, 146.1, 146.0, 145.9, 142.4, 140.1, 139.9, 137.5, 137.4 – 137.2 (m), 136.0, 125.3 – 125.1 (m), 120.8 – 120.5 (m), 110.6 – 110.0 (m), 91.8, 91.6, 48.5 – 48.3 (m), 13.9 – 13.8 (m), 13.4, 13.3; (^{19}F-^{13}C coupling not assigned); ^{19}F NMR (282 MHz, $CDCl_3$) δ -

111.7 (s, 1F, Ar*H*); LRMS (MALDI, *m/z*) calc. for $C_{131}H_{80}FN_3O_{10}$: 1874.59 (M^+), found 1875.91 (MH^+); GPC analysis ($CHCl_3$) M_n = 1571, M_w = 1611 g mol^{-1} relative to polystyrene, M_w/M_n = 1.025; BET surface area = 351 m^2 g^{-1}; total pore volume = 0.30 cm^3 g^{-1} at p/p^o = 0.98.

30 (**OMIM-12**)
4,4',4''-Tricyano-2,2',2'',3,3'',5,5',5'',6,6',6''-undecafluoro-[1,1':3',1''-terphenyl] (**2**) (0.097 g, 0.193 mmol), 7,14-di-*tert*-butyltriptycene-2,3-diol (**5c**) (0.410 g, 1.03 mmol) and potassium carbonate (0.320 g, 2.32 mmol) were reacted together in anhydrous DMF (10 ml) in accordance with general procedure 1. Purification was achieved *via* column chromatography (CH_2Cl_2/hexane/toluene, 2/1/1, R_f = 0.25) to give **OMIM-12** (mixture of regioisomers) (0.360 g, 81%) as a yellow powder (mp > 300°C); IR (CH_2Cl_2 film) 2962, 2905, 2869, 2237, 1446, 1285, 1264, 1140, 1010 cm^{-1}; ^{1}H NMR (500 MHz, $CDCl_3$) δ 7.46 – 6.61 (m, 40H, Ar*H*), 5.34 – 5.13 (m, 10H, C*H*), 1.32 – 1.14 (m, 90H, *t*Bu*H*); ^{13}C NMR (125 MHz, $CDCl_3$) δ 148.8 – 148.5 (m), 144.8 – 144.6 (m), 143.2, 143.0, 142.1 – 141.8 (m), 137.4 – 137.1 (m), 135.92, 135.86, 123.3 – 123.0 (m), 122.3 – 122.0 (m), 121.2 – 121.1 (m), 112.9 – 112.7 (m), 91.8, 53.5 – 53.2 (m), 34.9 – 34.7 (m), 31.8 – 31.7 (m) (^{19}F-^{13}C coupling not assigned, extra carbons due to regioisomeric nature); ^{19}F NMR (282 MHz, $CDCl_3$) δ -111.5 – -112.1 (cluster of singlets, 1F, Ar*F*); LRMS (MALDI, *m/z*) calc. for $C_{161}H_{140}FN_3O_{10}$: 2295.05 (M^+), found 2295.19; GPC analysis ($CHCl_3$) M_n = 2227, M_w = 2304 g mol^{-1} relative to polystyrene, M_w/M_n = 1.035; BET surface area = 726 m^2 g^{-1}; total pore volume = 0.72 cm^3 g^{-1} at p/p^o = 0.98.

31 (**OMIM-13**)
4,4',4''-Tricyano-2,2',2'',3,3'',5,5',5'',6,6',6''-undecafluoro-[1,1':3',1''-terphenyl] (**2**) (0.064 g, 0.127 mmol), 7,14-di-*tert*-butyl-9,10-dimethyltriptycene-2,3-diol (**5d**) (0.298 g, 0.699 mmol) and potassium carbonate (0.210 g, 1.52 mmol) were reacted together in anhydrous DMF (10 ml) in accordance with general procedure 1. Purification was achieved *via* column chromatography (hexane/CH_2Cl_2/toluene, 5/3/1, R_f = 0.25) to give **OMIM-13** (mixture of regioisomers) (0.202 g, 65%) as a yellow powder (mp > 300°C); IR (CH_2Cl_2 film) 2966, 2869, 2237, 1612, 1446, 1291, 1265, 1011 cm^{-1}; ^{1}H NMR (400 MHz, $CDCl_3$) δ 7.39 – 6.45 (m, 40H, Ar*H*), 2.40 – 1.68 (m, 30H, C*H*$_3$), 1.31 – 1.15 (m, 90H, *t*Bu*H*); ^{13}C NMR (125 MHz, $CDCl_3$) δ 148.1 – 147.9 (m), 147.5 – 147.2 (m), 146.79 – 146.78 (m), 146.5, 146.4, 146.2 – 146.1 (m), 145.9, 145.0 – 144.5 (m), 144.3, 139.7 – 139.6 (m), 137.0 – 136.7 (m), 136.0, 135.8, 121.8 – 121.4 (m), 120.2 – 119.8 (m), 117.8 – 117.5 (m), 117.13 – 117.07 (m), 110.3 – 109.9 (m), 48.4 – 48.1 (m), 34.9 – 34.8 (m), 31.7, 13.9 – 13.7 (m) (^{19}F-^{13}C coupling not assigned, extra carbons due to regioisomeric nature); ^{19}F NMR (282 MHz, $CDCl_3$) δ -111.3 – -112.5 (cluster of singlets, 1F, Ar*F*); LRMS (MALDI, *m/z*) calc. for $C_{171}H_{160}FN_3O_{10}$: 2435.21 (M^+), found 2434.75; GPC analysis ($CHCl_3$) M_n = 2147, M_w = 2214 g mol^{-1} relative to polystyrene, M_w/M_n = 1.031; BET surface area = 651 m^2 g^{-1}; total pore volume = 0.47 cm^3 g^{-1} at p/p^o = 0.98.

32 (**OMIM-14**)
4,4',4''-Tricyano-2,2',2'',3,3'',5,5',5'',6,6',6''-undecafluoro-[1,1':3',1''-terphenyl] (**2**) (0.0326 g, 0.0656 mmol), (6r,13r)-18,19-dimethoxy-1,1,4,4,8,8,11,11-octamethyl-1,2,3,4,6,8,9,10,11,13-decahydro-6,13-[1,2]benzenopentacene (**5e**)[6] (0.180 g, 0.355 mmol) and potassium carbonate (0.150 g, 1.09 mmol) were reacted together in anhydrous DMF (10 ml) in accordance with general procedure 1. Purification was achieved *via* column chromatography (hexane/CH_2Cl_2/toluene, 7/2/1, R_f = 0.3) to give **OMIM-14** (0.144 g, 77%) as a yellow powder (mp > 300°C); IR (CH_2Cl_2 film) 2959, 2925, 2860, 2238, 1473, 1443, 1284, 1139, 1008 cm^{-1}; ^{1}H NMR (500 MHz, $CDCl_3$) δ 7.29 (s, 2H, Ar*H*), 7.24 – 7.21 (m, 13H, Ar*H*), 7.15 (s, 2H, Ar*H*), 7.11 (s, 4H, Ar*H*), 7.01 (s, 2H, Ar*H*), 7.00 (s, 2H, Ar*H*), 6.82 (s, 3H, Ar*H*), 6.74 (s, 2H, Ar*H*), 5.24 (s, 1H, C*H*), 5.17 (s, 2H, C*H*), 5.15 (s, 2H, C*H*), 5.12 (m, 4H, C*H*), 5.04 (s, 1H, C*H*), 1.62 – 1.54 (m, 40H, C*H*$_2$), 1.29 – 1.10 (m, 120H, C*H*$_3$); ^{13}C NMR (125 MHz, $CDCl_3$) δ 143.9, 143.7, 143.4 – 143.3 (m), 141.9 – 141.5 (m), 139.9, 139.4, 137.9, 137.5,

137.3, 137.2, 137.1, 136.8, 136.0, 135.6, 121.8 – 121.5 (m), 113.1, 112.7, 112.6, 122.2, 111.9, 110.5, 109.8, 92.6, 91.9, 91.3, 91.0, 53.1 – 53.0 (m), 52.74, 52.68, 35.4, 34.49, 34.45, 34.41, 34.40, 32.8, 32.6, 32.51, 32.46, 32.32, 32.29, 32.2, 32.1 – 32.0 (m) (^{19}F-^{13}C coupling not assigned); ^{19}F NMR (282 MHz, $CDCl_3$) *δ* -112.6 (s, 1F, Ar*F*); LRMS (MALDI, *m/z*) calc. for $C_{201}H_{200}FN_3O_{10}$: 2836.53 (M^+), found 2838.54 (MH^+); GPC analysis ($CHCl_3$) M_n = 1973, M_w = 2043 g mol^{-1} relative to polystyrene, M_w/M_n = 1.036; BET surface area = 698 m^2 g^{-1}; total pore volume = 0.44 cm^3 g^{-1} at p/p^o = 0.98.

37 (**OMIM-16**)

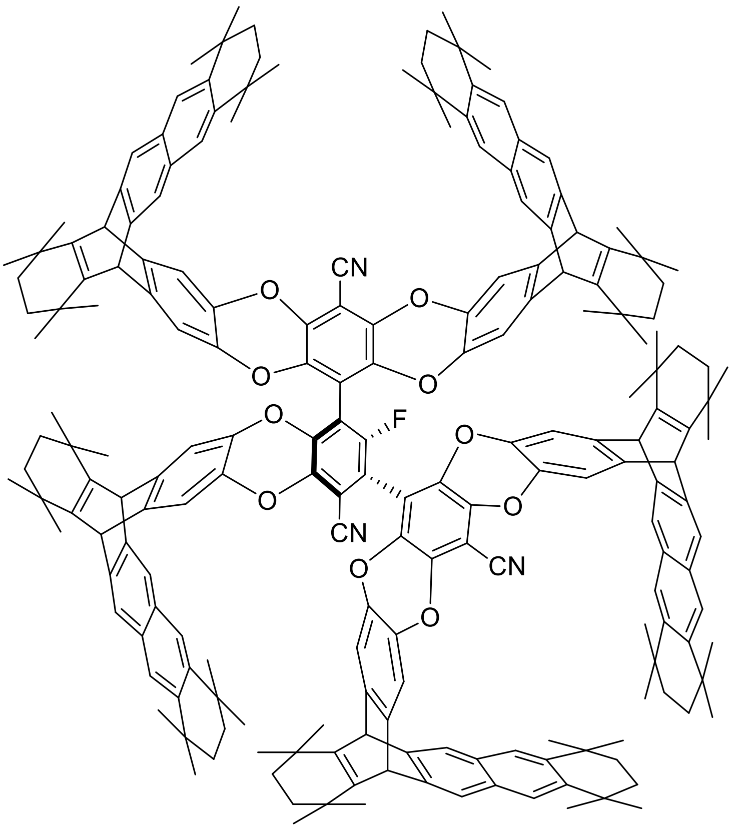

4,4',4''-Tricyano-2,2',2'',3,3'',5,5',5'',6,6',6''-undecafluoro-[1,1':3',1''-terphenyl] (**2**) (0.0363 g, 0.0721 mmol), (5R,14S)-2,3-dimethoxy-8,8,11,11,17,17,20,20-octamethyl-5,8,9,10,11,14,17,18,19,20-decahydro-5,14-[1,2]benzenopentacene (**6**)[6] (0.201 g, 0.397 mmol) and potassium carbonate (0.165 g, 1.19 mmol) were reacted together in anhydrous DMF (10 ml) in accordance with general procedure 1. Purification was achieved *via* column chromatography (hexane/CH_2Cl_2/toluene, 7/2/1, R_f = 0.3) to give the **OMIM-16** (mixture of regioisomers) (0.160 g, 78%) as a yellow powder (mp > 300°C); IR (CH_2Cl_2 film) 2957, 2925, 2861, 2237, 1610, 1447, 1284, 1141, 1009 cm^{-1}; ^{1}H NMR (500 MHz, $CDCl_3$) *δ* 7.96 – 7.20 (m, 20H, Ar*H*), 7.11 – 6.37 (m, 10H, Ar*H*), 5.07 – 4.44 (m, 10H, C*H*), 1.74 – 1.67 (m, 20H, C*H*$_2$), 1.39 – 0.30 (m, 140H, C*H*$_2$/C*H*$_3$); ^{13}C NMR (125 MHz, $CDCl_3$) *δ* 146.9 – 146.4 (m), 144.1 – 143.5 (m), 142.8 – 142.4 (m), 140.2 – 140.1 (m), 137.7 – 137.1 (m), 130.2 – 130.0 (m), 124.9 – 124.7 (m), 119.6 – 119.4 (m), 111.7 – 111.5 (m), 91.5, 91.3, 50.1 – 49.7 (m), 35.5 – 35.4 (m), 34.7 – 34.6 (m), 34.2 – 34.0 (m), 32.8 – 32.6 (m), 27.2 – 26.7 (m) (^{19}F-^{13}C coupling not assigned, extra carbons due to regioisomeric nature); ^{19}F NMR (282 MHz, $CDCl_3$) *δ* -109.9 – -112.2 (cluster of singlets, 1F, Ar*F*); LRMS (MALDI, *m/z*) calc. for $C_{201}H_{200}FN_3O_{10}$: 2836.53 (M^+), found 2861.81 (MNa^+); GPC analysis ($CHCl_3$) M_n = 2123, M_w = 2205 g mol^{-1} relative to polystyrene, M_w/M_n = 1.039; BET surface area = 591 m^2 g^{-1}; total pore volume = 0.49 cm^3 g^{-1} at p/p^o = 0.98.

38 (**OMIM-17**)

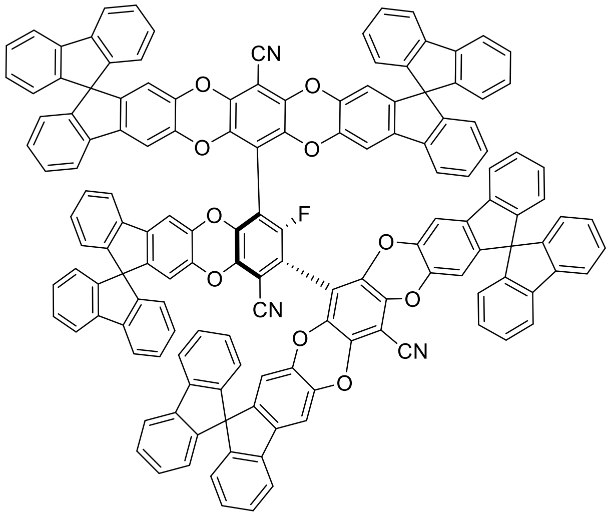

4,4’,4’’-Tricyano-2,2',2'',3,3'',5,5',5'',6,6',6''-undecafluoro-[1,1':3',1''-terphenyl] (**2**) (0.050 g, 0.099 mmol), 2,3-dihydroxy-9,9’-spirobifluorene (**7**) (0.207 g, 0.596 mmol) and potassium carbonate (0.218 g, 1.58 mmol) were reacted together in anhydrous DMF (5 ml) in accordance with general procedure 1. The crude material was reprecipitated twice with methanol from CH_2Cl_2 solution, and then refluxed in methanol to give **OMIM-17** (mixture of regioisomers) (0.178 g, 88%) as a fluorescent yellow solid; IR (CH_2Cl_2 film) 3058, 1495, 1599, 1353, 1277, 1162, 1012 cm^{-1}; ^{1}H NMR (500 MHz, $CDCl_3$) δ 7.70 (m, 15H Ar*H*), 7.40 (m, 12H, Ar*H*), 7.00 (m, 18H, Ar*H*), 6.63 (m, 17H, Ar*H*), 6.20 (m, 8H Ar*H*); ^{13}C NMR (125 MHz, $CDCl_3$) δ 149.1, 148.1, 148.0, 147.9, 147.8, 147.7, 146.0, 145.7, 145.2, 145.1, 141.8, 141.7, 141.6, 141.4, 140.8, 140.7, 140.6, 140.4, 140.3, 140.2, 140.1, 139.9, 139.8, 139.1, 138.9, 138.8, 138.7, 128.3, 128.1, 127.9, 124.2, 124.1, 123.9, 120.4, 120.2, 120.1, 112.6, 112.5, 108.6, 108.5, 91.9, 91.8, 91.6, 91.5, 91.3, 65.8, 65.8, 65.7 (clusters of peaks, ^{19}F-^{13}C coupling not assigned); ^{19}F NMR (471 MHz, $CDCl_3$) δ -112.0 (clusters of singlets, 1F, Ar*F*); LRMS (MALDI, *m/z*) calc. for $C_{146}H_{70}FN_3O_{10}$: 2044.51 (M^+), found: 2046.47 (MH^+); GPC analysis ($CHCl_3$) M_n = 1283, M_w = 1334 g mol^{-1} relative to polystyrene, M_w/M_n = 1.039; BET surface area = 472 m^2 g^{-1}; total pore volume = 0.3563 cm^3 g^{-1} at p/p^o = 0.98.

39 (OMIM-18)

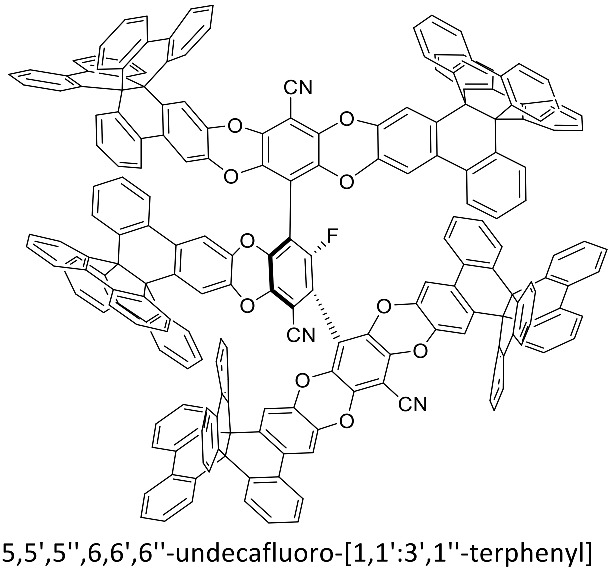

4,4’,4’’-Tricyano-2,2',2'',3,3'',5,5',5'',6,6',6''-undecafluoro-[1,1':3',1''-terphenyl] (**2**) (0.035 g, 0.068 mmol), dihydroxypropellane (**8**) (0.210 g, 0.417 mmol) and potassium carbonate (0.150 g, 1.09 mmol) were reacted

together in anhydrous DMF (5 ml) in accordance with general procedure 1. The crude material was reprecipitated twice with methanol from CH_2Cl_2 solution, and then refluxed in methanol for 12 hrs to give **OMIM-18** (mixture of regioisomers) (0.164 g, 84%) as a fluorescent yellow solid; IR (CH_2Cl_2 film) 3065, 1600, 1439, 1300, 1277, 1016 cm^{-1}; ^1H NMR (500 MHz, $CDCl_3$) δ 7.60 (br m, 24H, Ar*H*), 7.20 (br m, 28H, Ar*H*), 7.02 (br m, 24H, Ar*H*), 6.40 (br m, 32H, Ar*H*); ^{13}C NMR (126 MHz, $CDCl_3$) δ 139.6, 139.2, 139.1, 138.9, 138.7, 138.6, 138.5, 138.4, 135.0, 134.7, 134.4, 129.4, 129.3, 129.2, 129.1, 128.8, 127.8, 127.6, 127.5, 127.3, 124.2, 124.1, 123.9, 123.7, 117.6, 117.5, 112.2, 112.1, 112.0, 53.3, 53.2, 53.1, 52.9, 52.9 (clusters of peaks, ^{19}F-^{13}C coupling not assigned); ^{19}F NMR (470 MHz, $CDCl_3$) δ -111.0 (clusters of singlets, 1F, Ar*F*); LRMS (MALDI, *m/z*) calc. $C_{211}H_{110}FN_3O_{10}$: 2865.82 (M^+), found: 2867.23 (MH^+); GPC analysis ($CHCl_3$) M_n = 1428, M_w = 1481 g mol^{-1} relative to polystyrene, M_w/M_n = 1.037; BET surface area = 613 m^2 g^{-1}; total pore volume = 0.4886 cm^3 g^{-1} at p/p^o = 0.98.

SI Figures

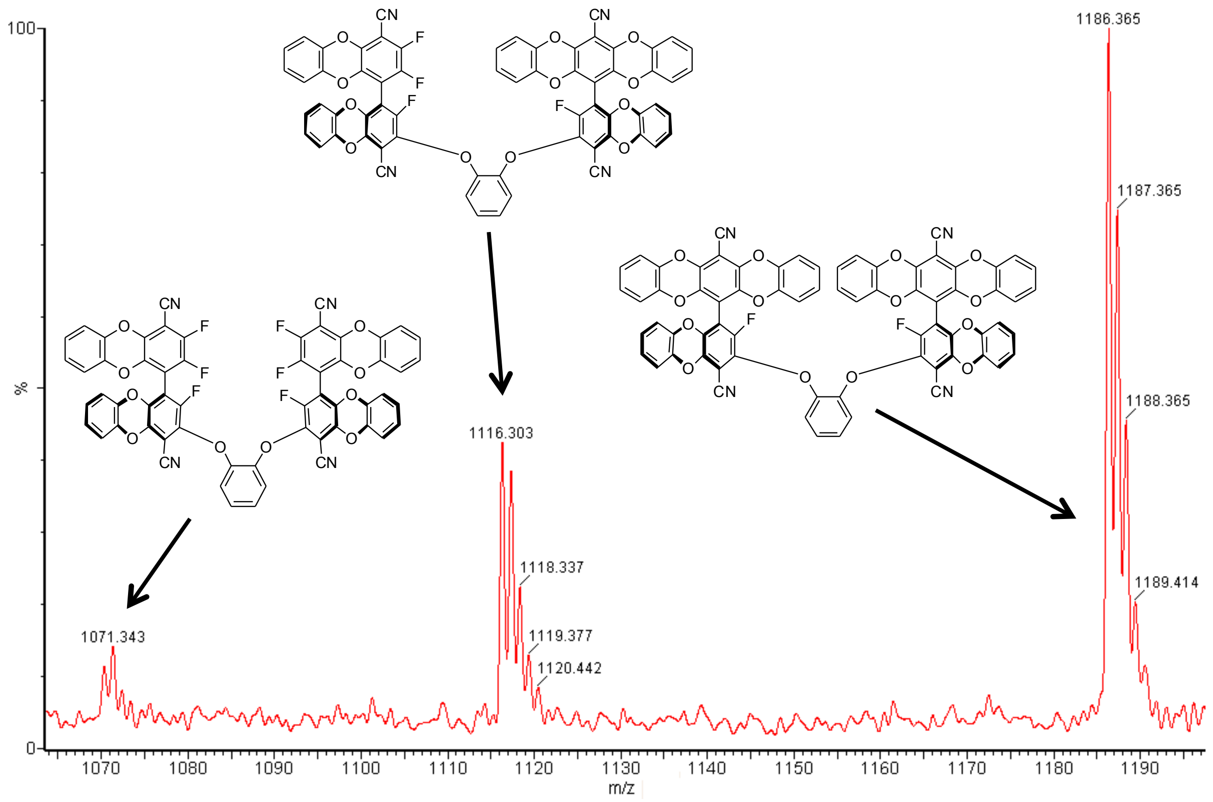

Figure SI1. MALDI mass spectrum of *bridged products*, found during the synthesis of **10**. Larger *multi-bridged* compounds were also visible.

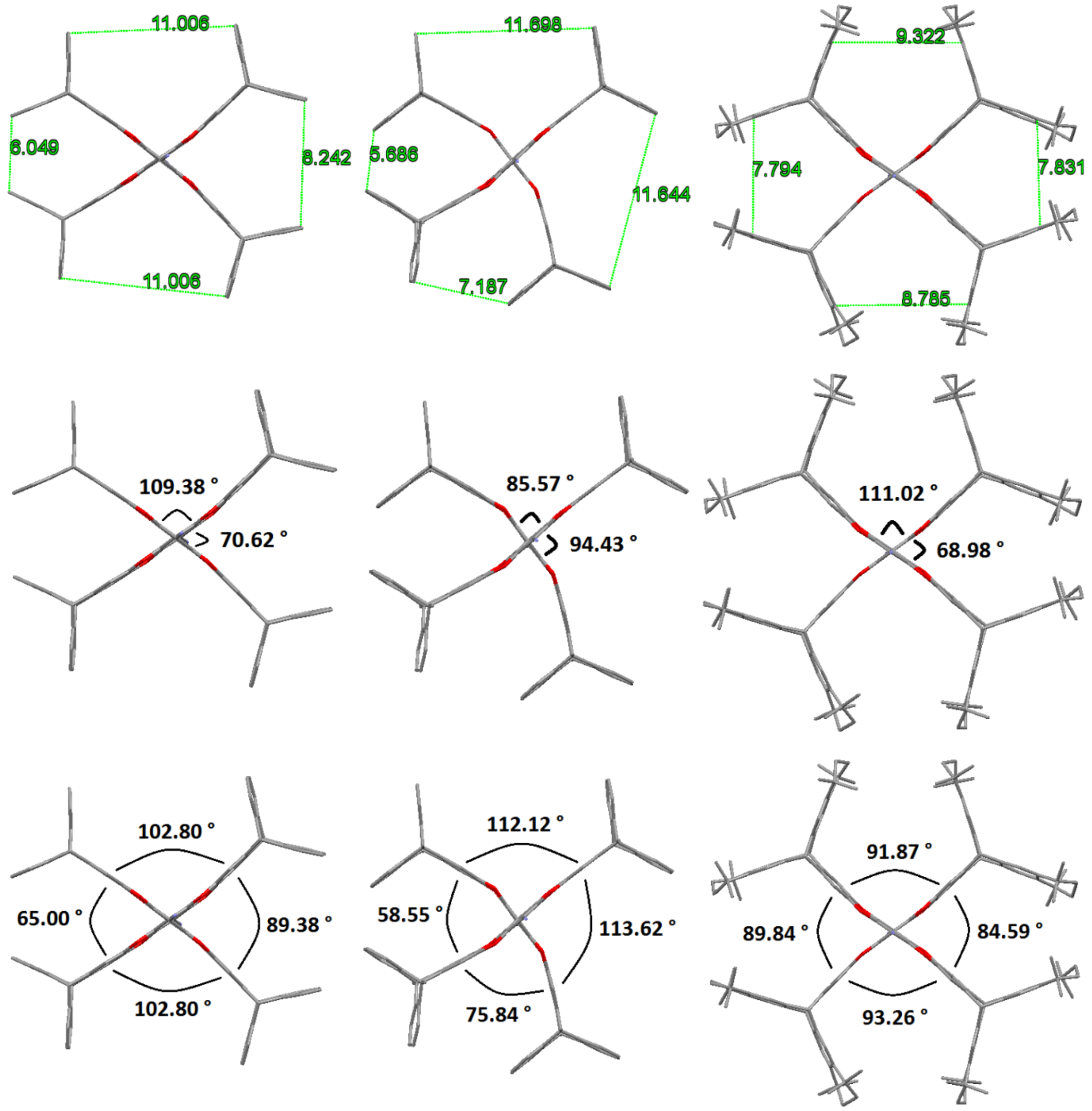

Figure SI2. Crystal structures of **OMIM-1** (left), **OMIM-8** (center) and **OMIM-5**[6] (right), showing various interatomic distances (Å) and angles. Protons and solvent molecules removed for clarity.

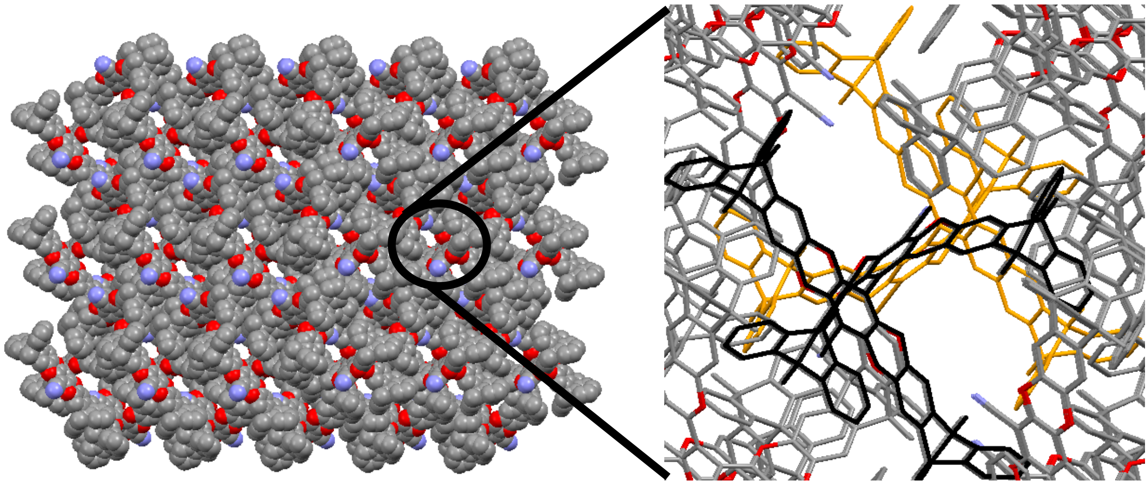

Figure SI3. Packing of **OMIM-8** molecules. Space filling display (left) showing channels and expanded view (right) showing relative location and size of channel. Protons and solvent molecules removed for clarity.

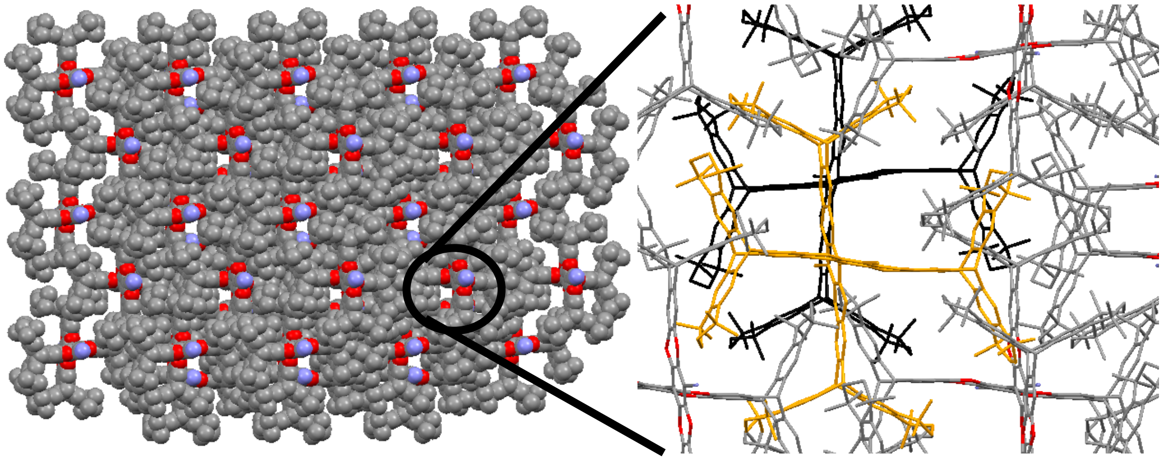

Figure SI4 Packing of **OMIM-5** molecules.[6] Space filling display (left) showing channels and expanded view (right) showing relative location of the channels. Protons and solvent molecules removed for clarity.

Full adsorption/desorption Isotherms

Biphenyl adducts/OMIMs

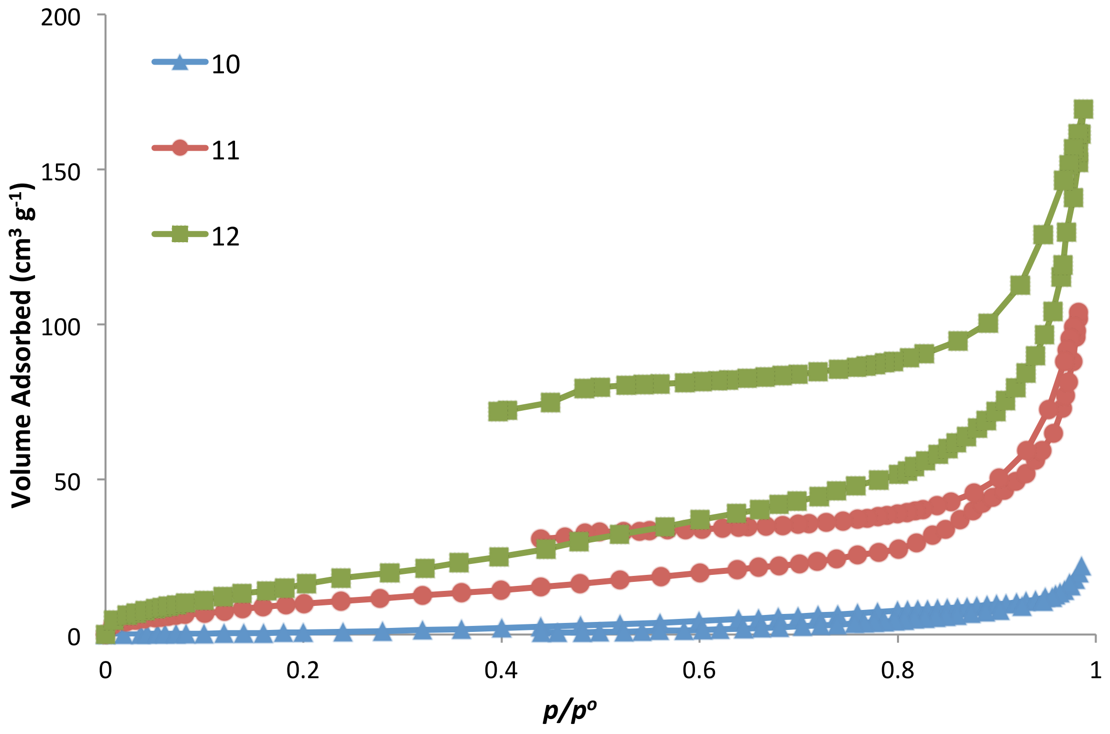

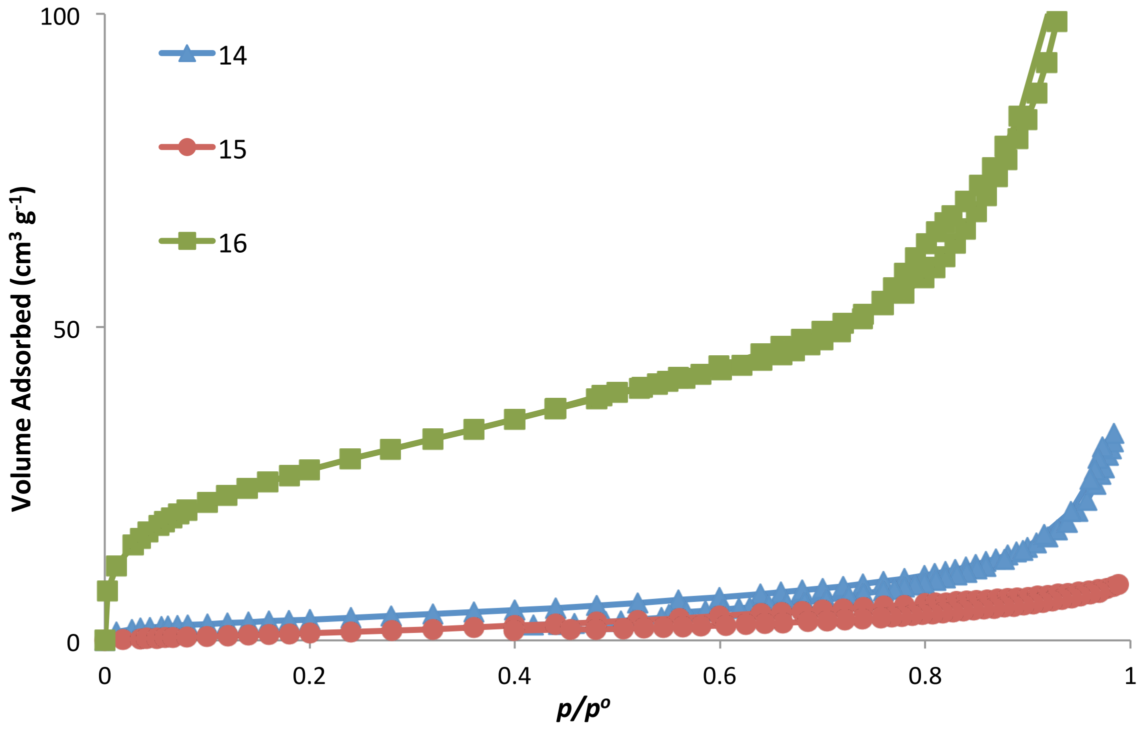

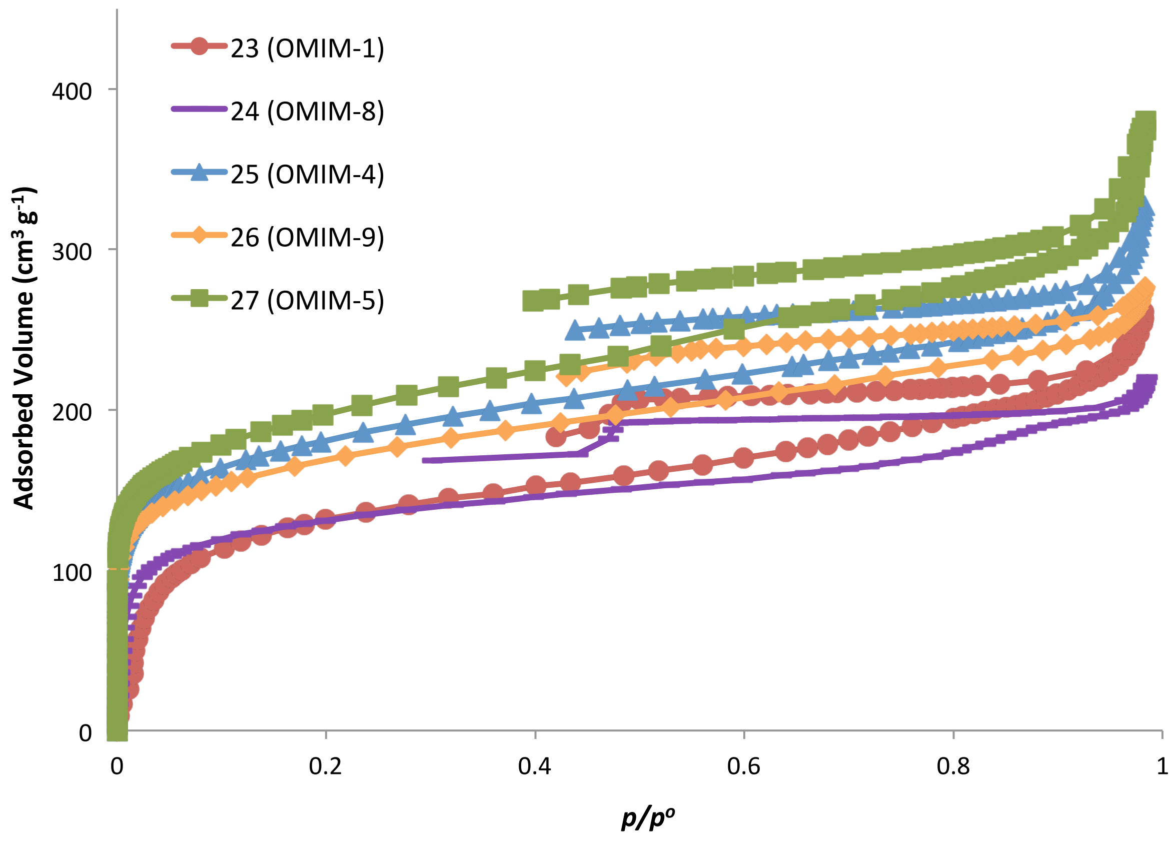
23 (OMIM-1)
24 (OMIM-8)
25 (OMIM-4)
26 (OMIM-9)
27 (OMIM-5)
Adsorbed Volume (cm3 g-1)
p/po
0
100
200
300
400
0
0.2
0.4
0.6
0.8
1

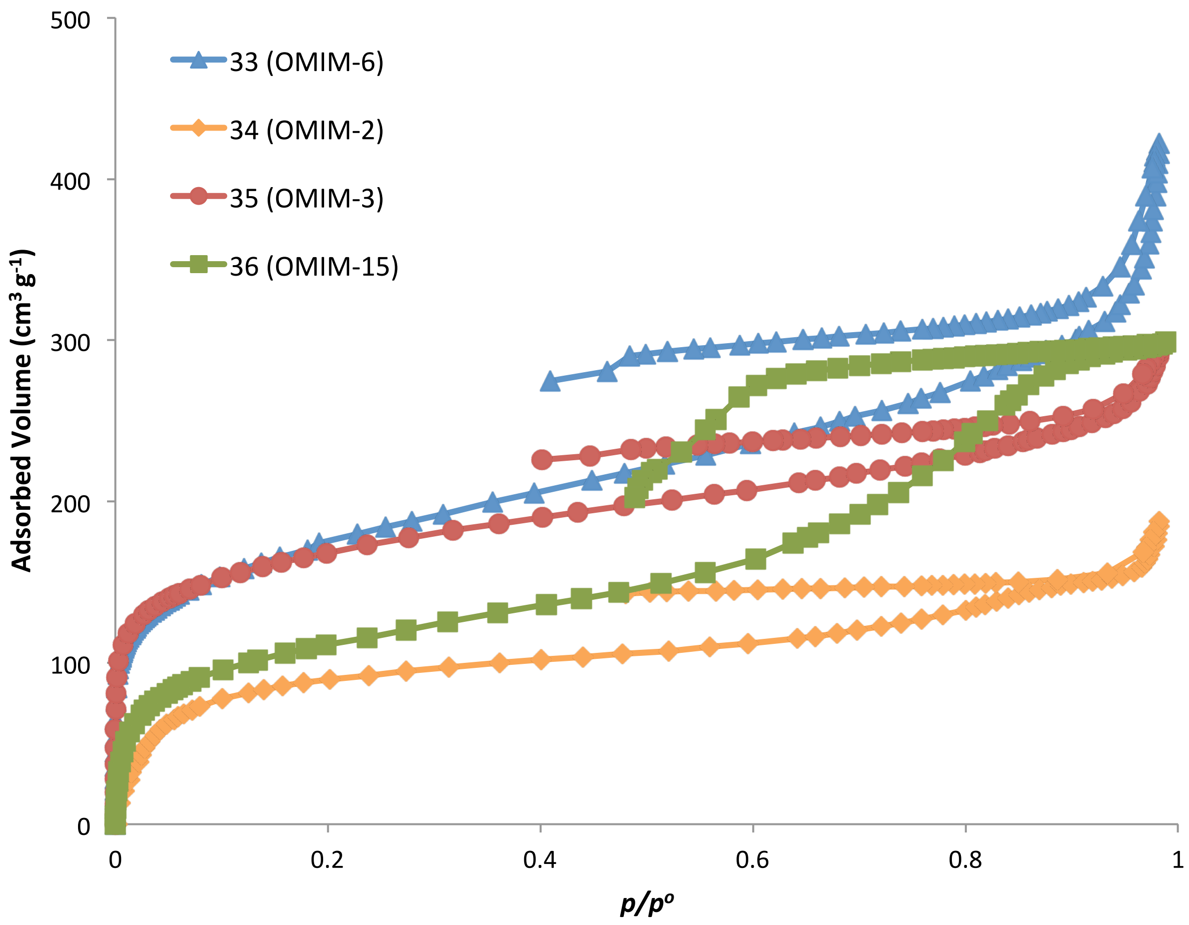
33 (OMIM-6)
34 (OMIM-2)
35 (OMIM-3)
36 (OMIM-15)
Adsorbed Volume (cm3 g-1)
p/po
0
100
200
300
400
500
0
0.2
0.4
0.6
0.8
1

Terphenyl adducts/OMIMs

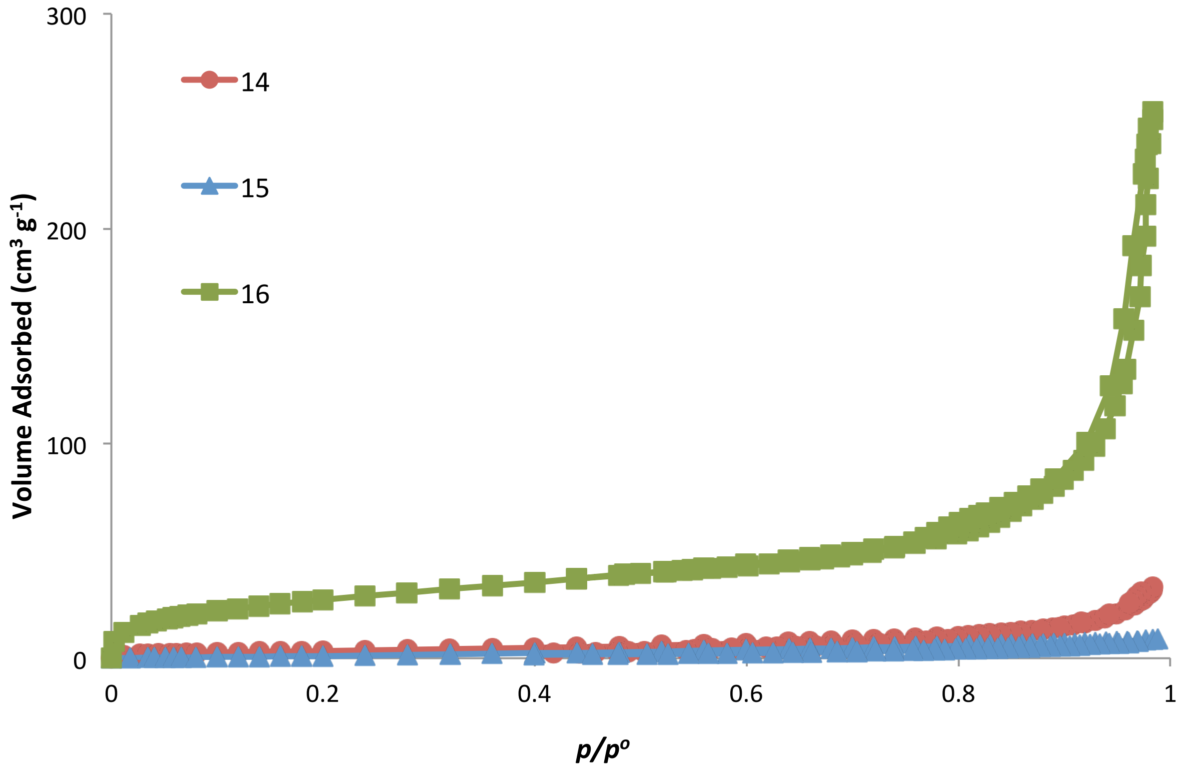

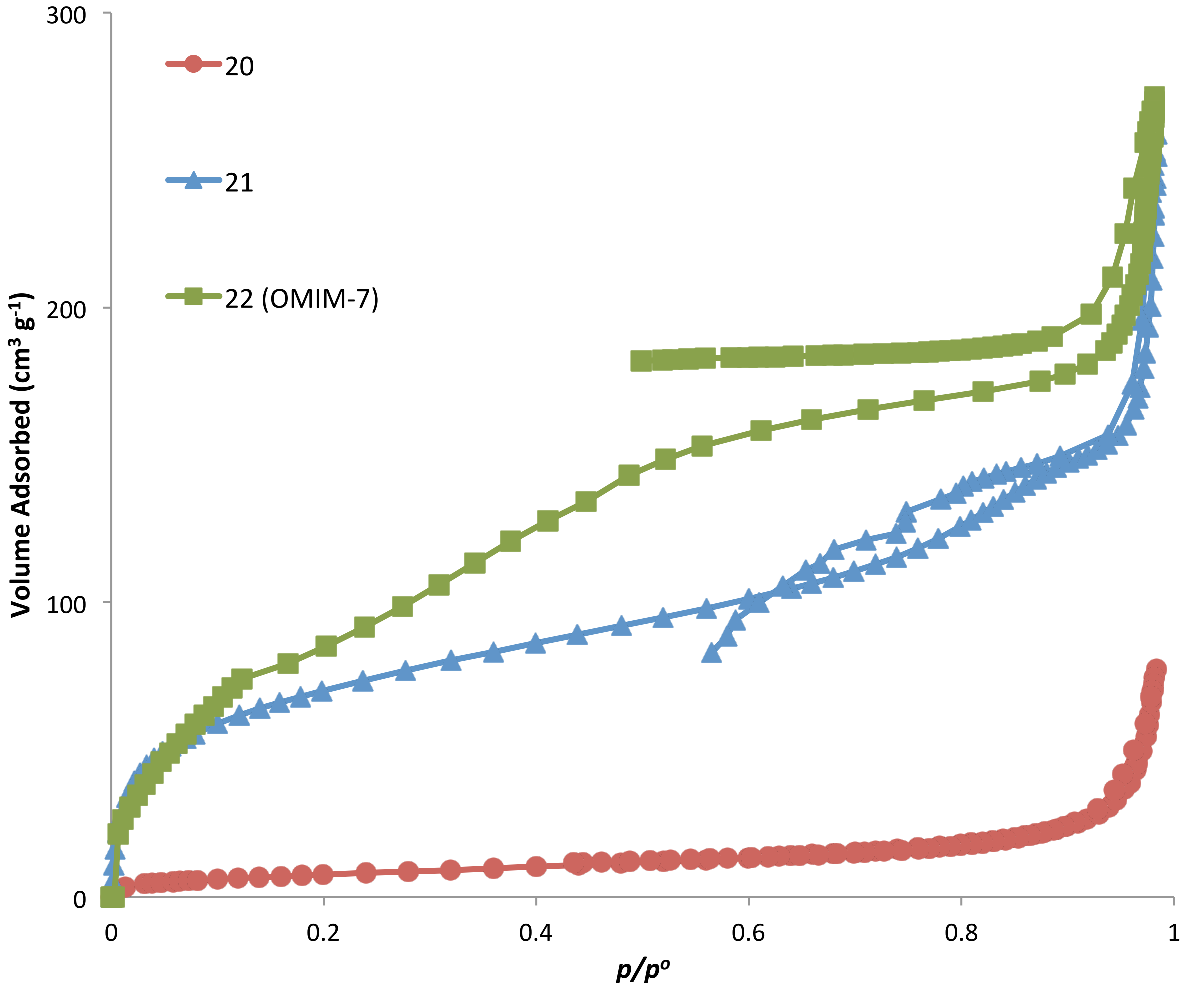

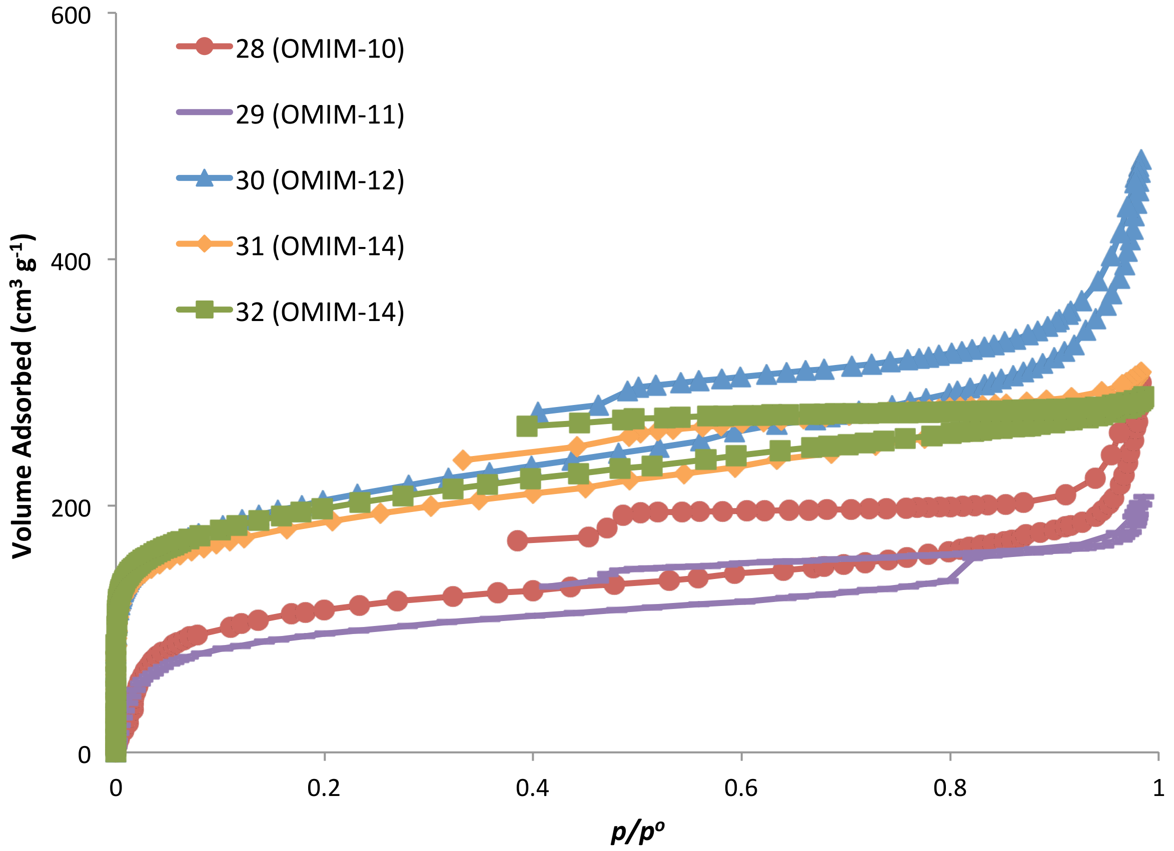

600
400
200
0
0
0.2
0.4
0.6
0.8
1
Volume Adsorbed (cm3 g-1)
p/po
28 (OMIM-10)
29 (OMIM-11)
30 (OMIM-12)
31 (OMIM-14)
32 (OMIM-14)

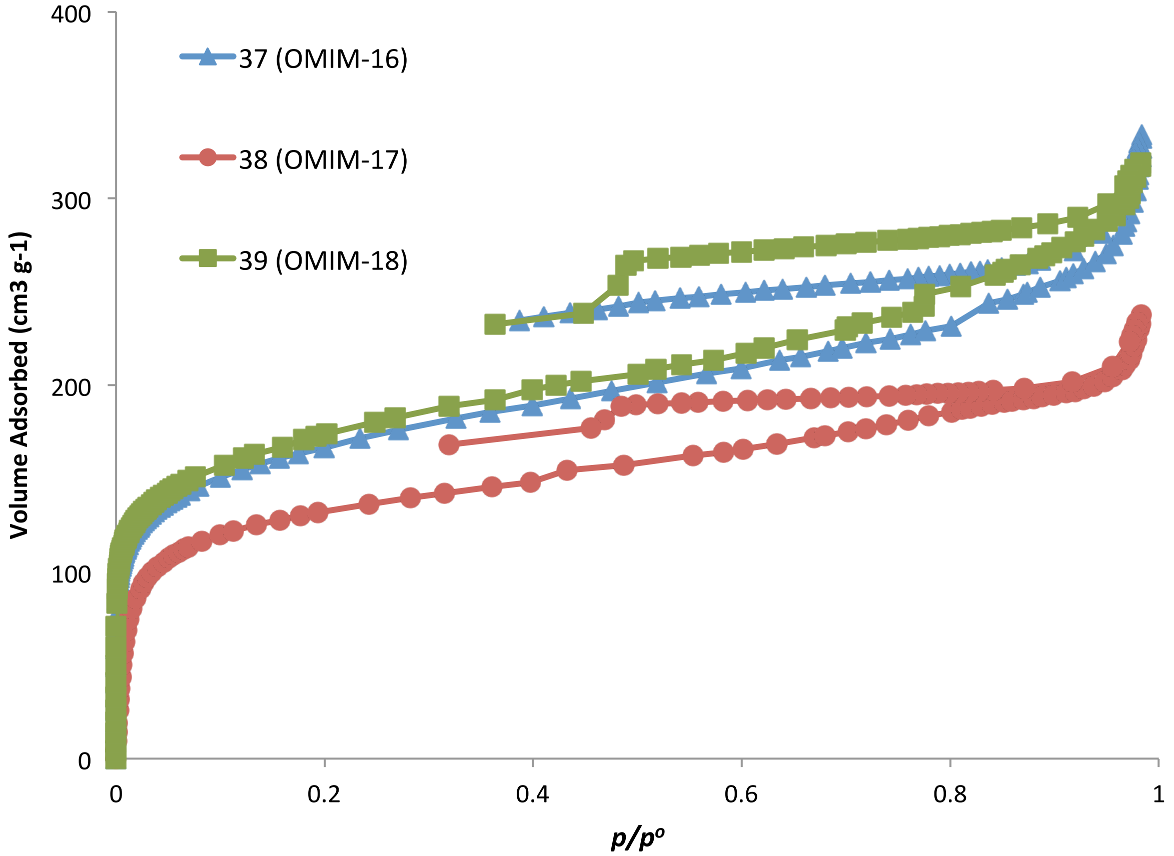

400
300
200
100
0
0
0.2
0.4
0.6
0.8
1
Volume Adsorbed (cm3 g-1)
p/po
37 (OMIM-16)
38 (OMIM-17)
39 (OMIM-18)

NMR Spectra

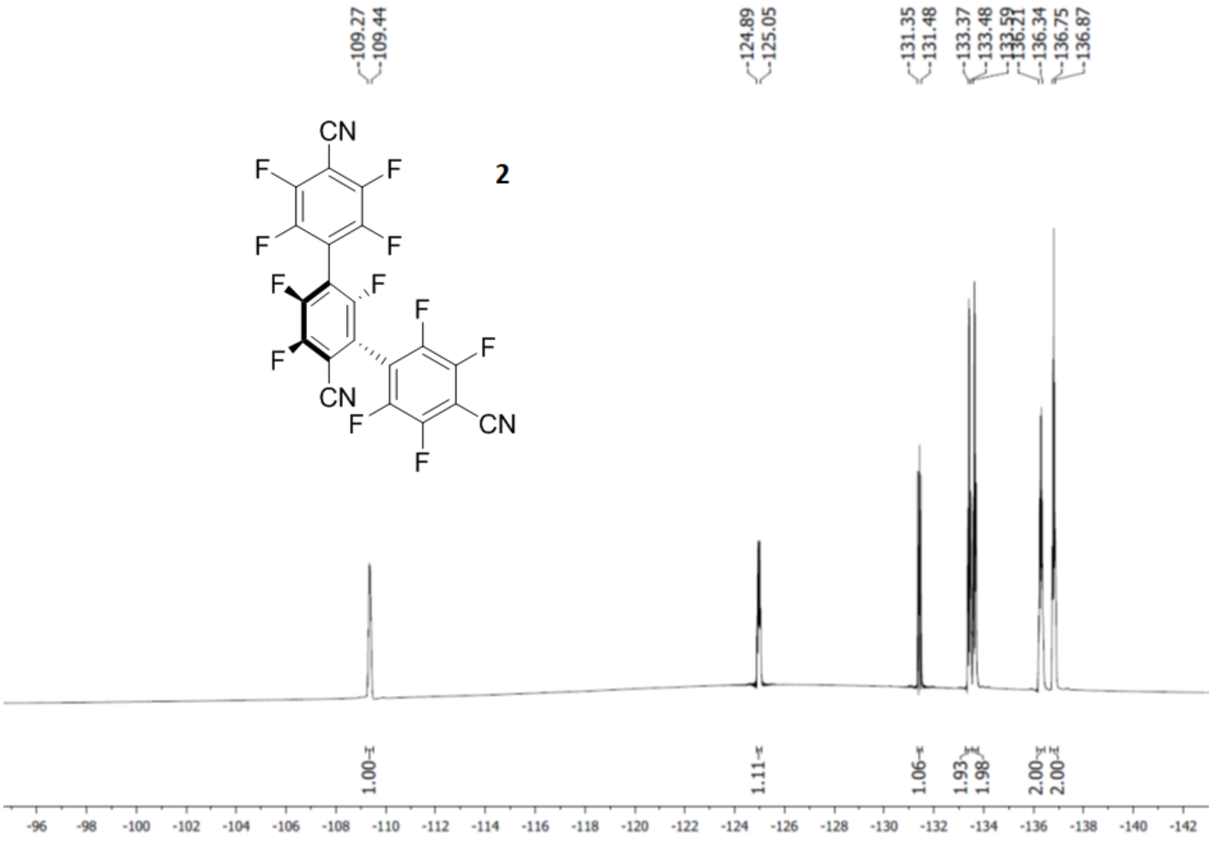

-109.27
-109.44
-124.89
-125.05
-131.35
-131.48
-133.37
-133.48
-133.59
-136.21
-136.34
-136.75
-136.87
CN
F
CN
CN
2
1.00
1.11
1.06
1.93
1.98
2.00
2.00
-96 -98 -100 -102 -104 -106 -108 -110 -112 -114 -116 -118 -120 -122 -124 -126 -128 -130 -132 -134 -136 -138 -140 -142

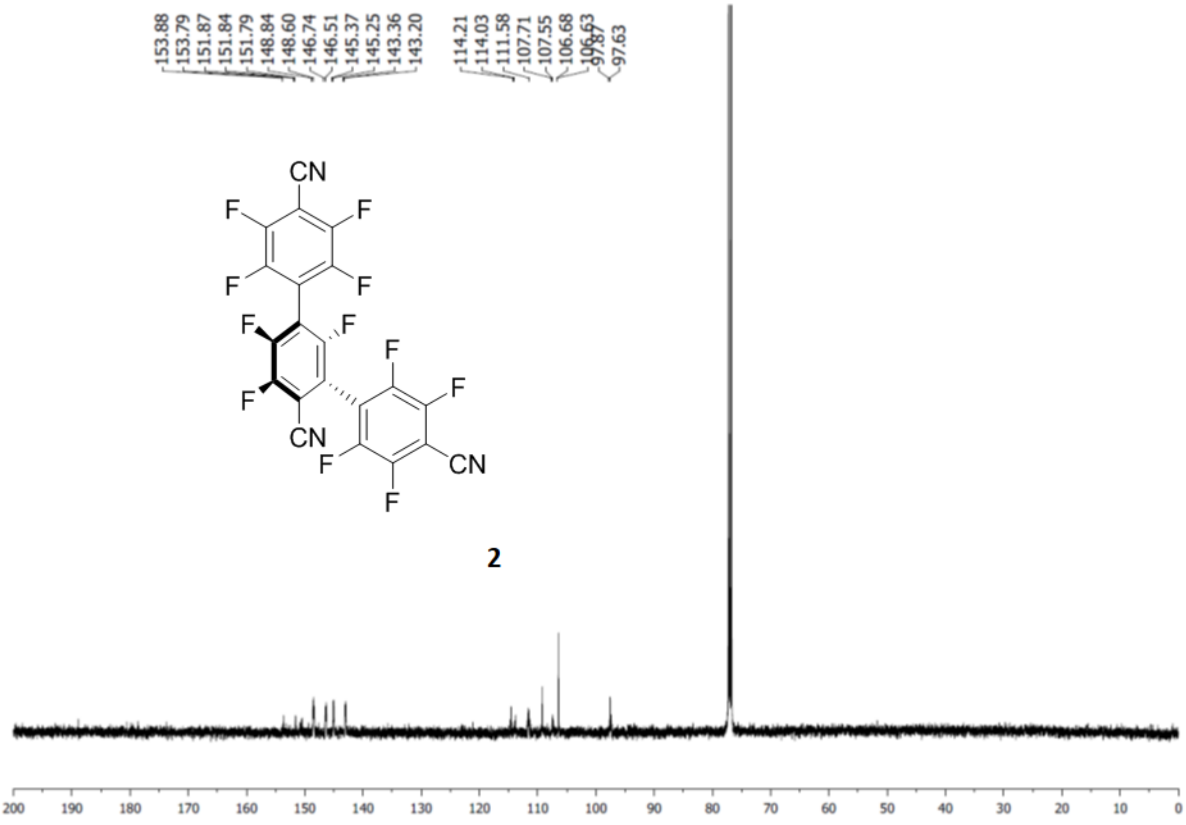

153.88
153.79
151.87
151.84
151.79
148.84
148.60
146.74
146.51
145.37
145.25
143.36
143.20
114.21
114.03
111.58
107.71
107.55
106.68
106.63
97.87
97.63
CN
F
CN
CN
2
200 190 180 170 160 150 140 130 120 110 100 90 80 70 60 50 40 30 20 10 0

6,7-Dimethoxy-1,1,4,4-tetramethyl-1,2,3,4-tetrahydronaphthalene

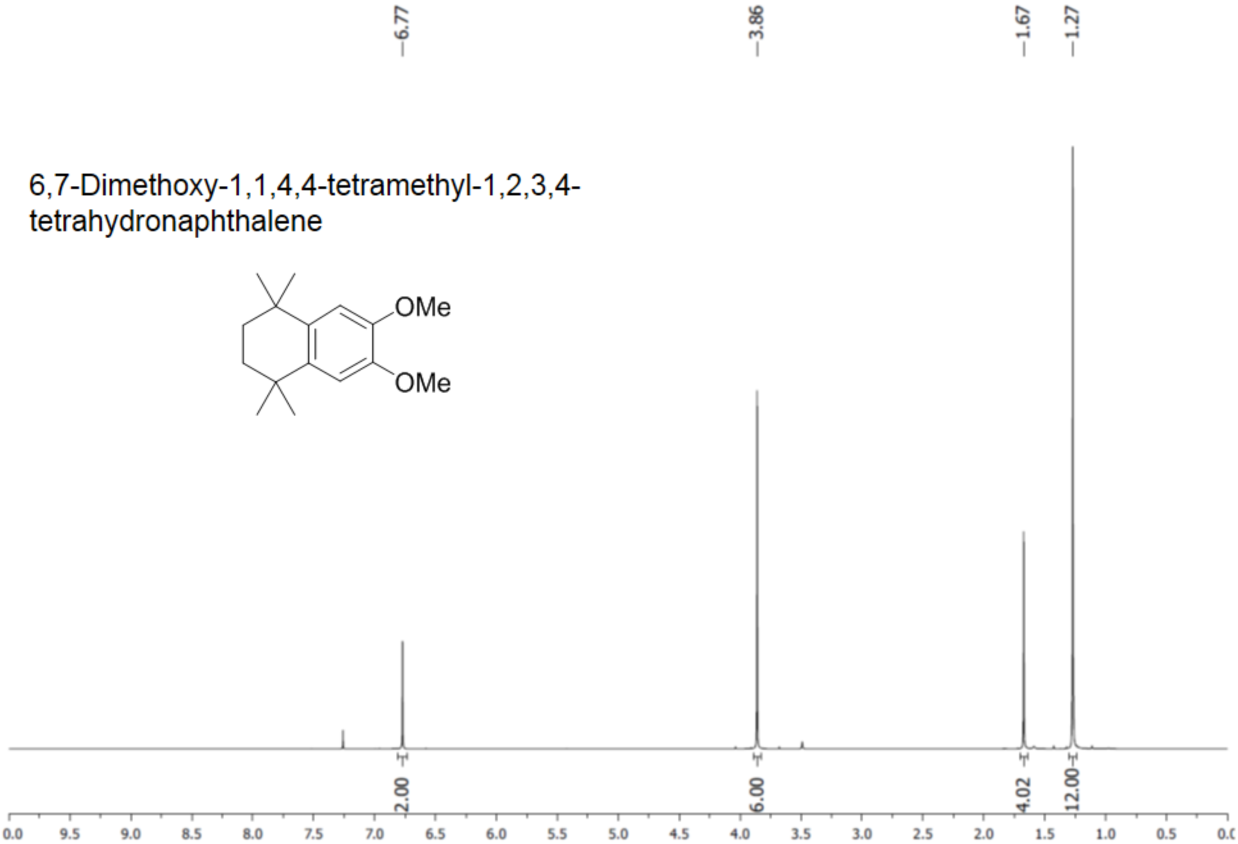

6,7-Dimethoxy-1,1,4,4-tetramethyl-1,2,3,4-tetrahydronaphthalene

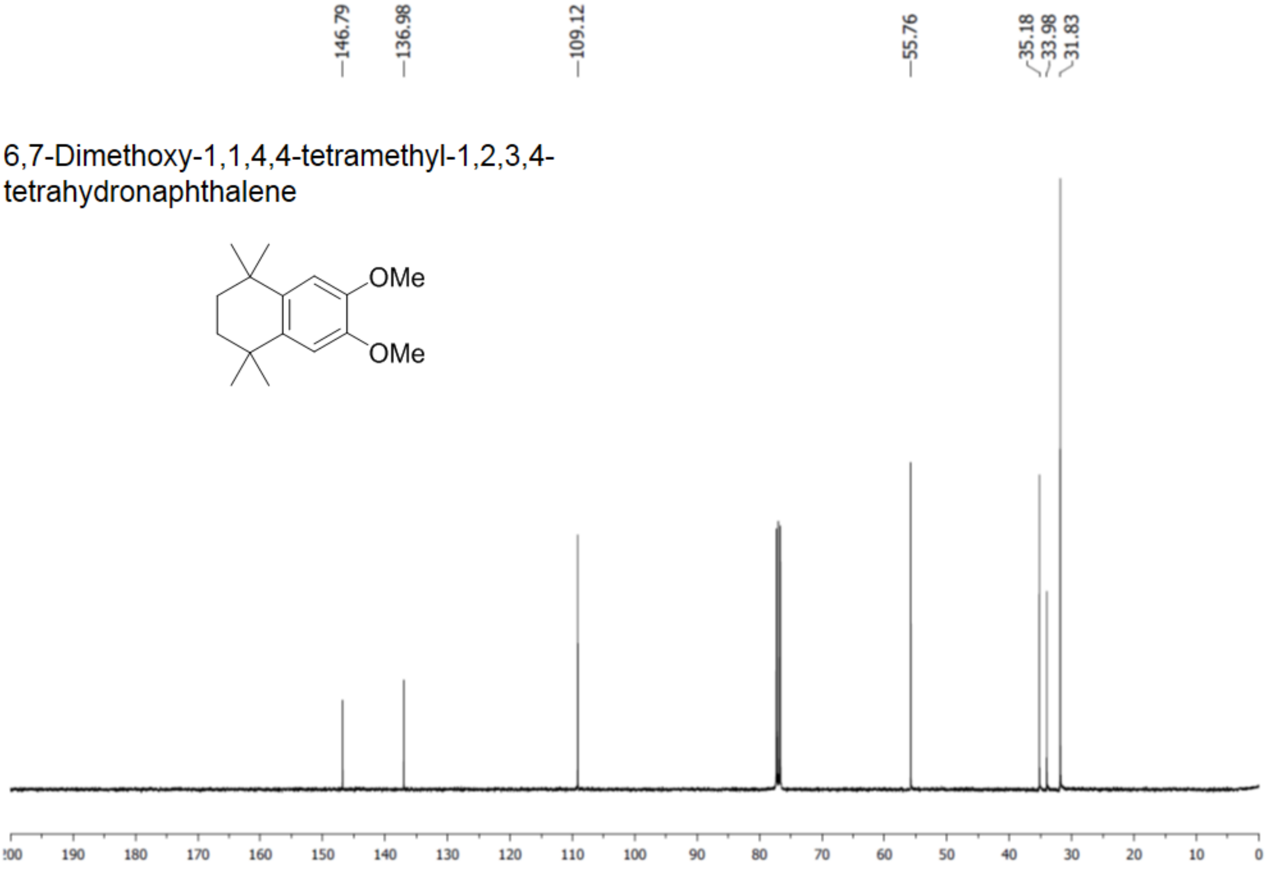

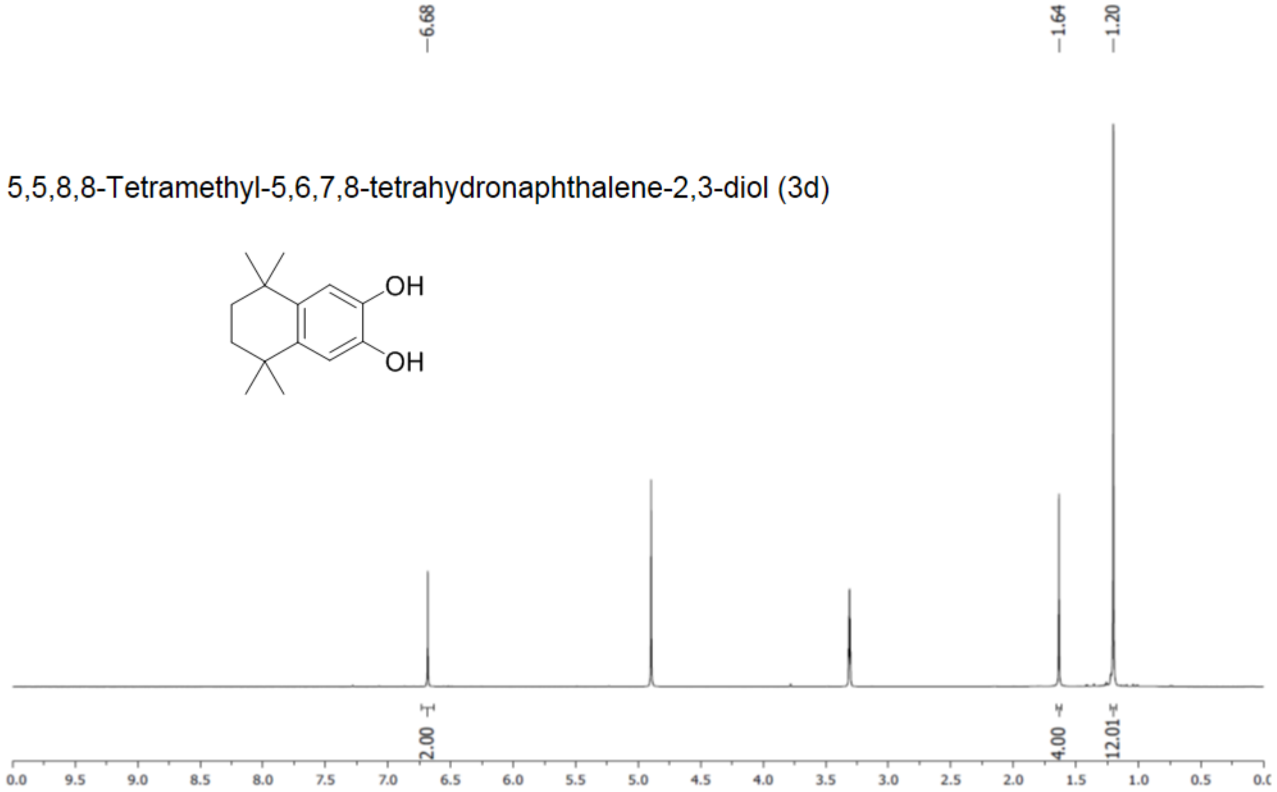
5,5,8,8-Tetramethyl-5,6,7,8-tetrahydronaphthalene-2,3-diol (3d)
OH
OH
6.68
1.64
1.20
2.00
4.00
12.01
10.0 9.5 9.0 8.5 8.0 7.5 7.0 6.5 6.0 5.5 5.0 4.5 4.0 3.5 3.0 2.5 2.0 1.5 1.0 0.5 0.0

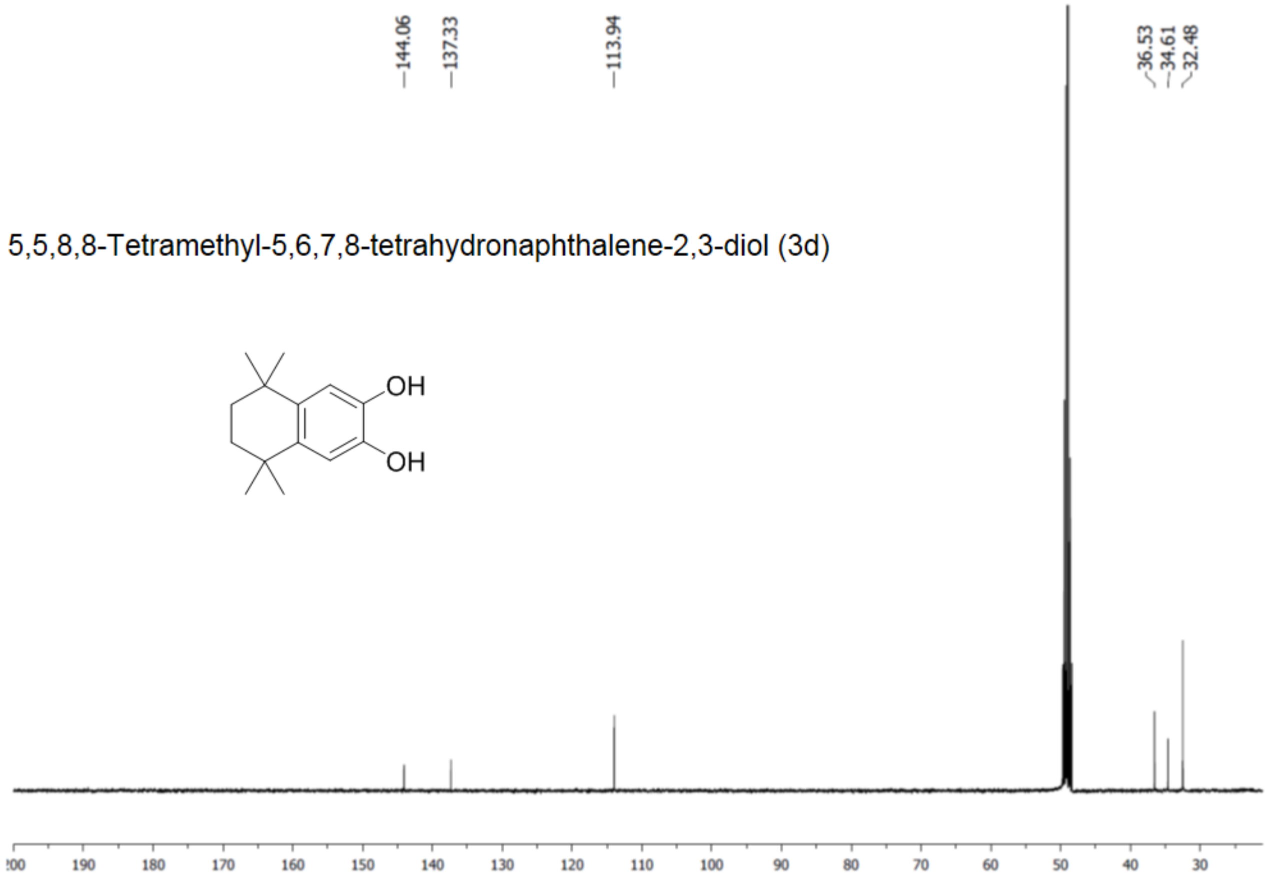
5,5,8,8-Tetramethyl-5,6,7,8-tetrahydronaphthalene-2,3-diol (3d)
OH
OH
144.06
137.33
113.94
36.53
34.61
32.48
200 190 180 170 160 150 140 130 120 110 100 90 80 70 60 50 40 30

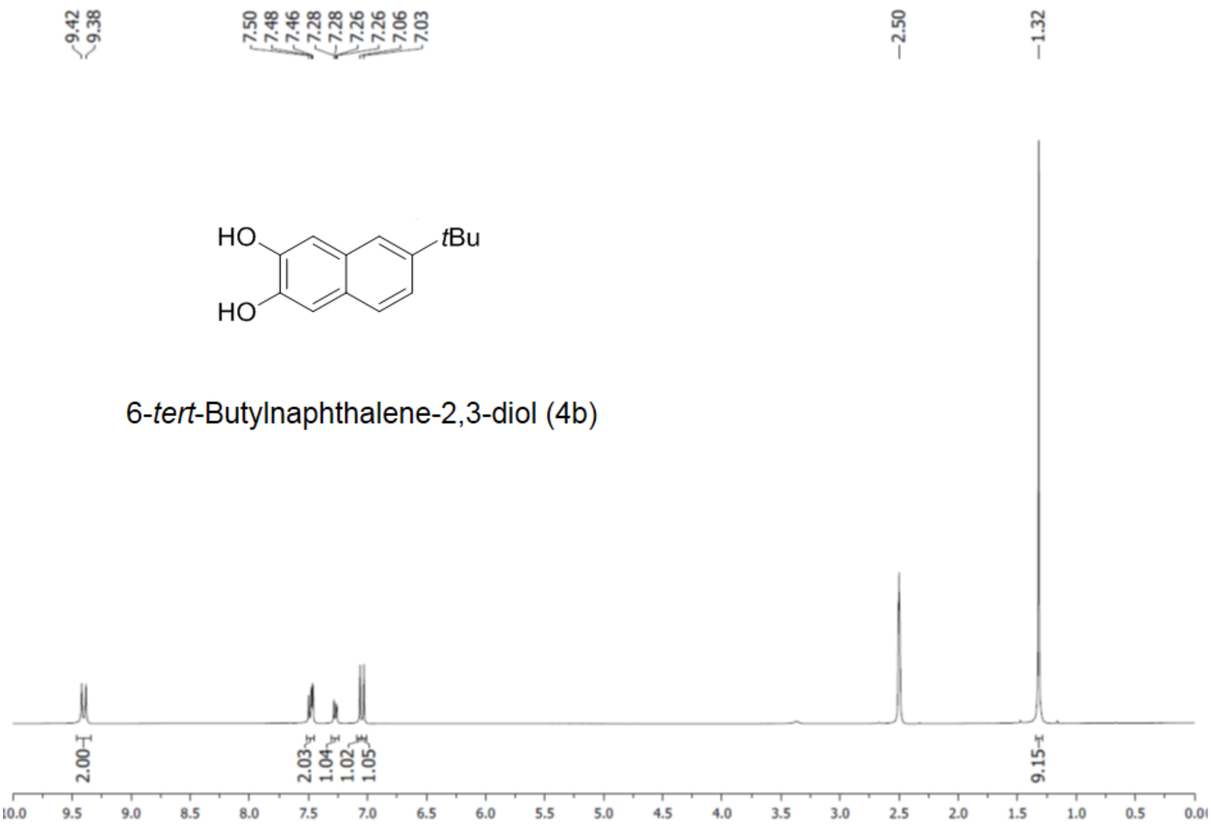

6-*tert*-Butylnaphthalene-2,3-diol (4b)

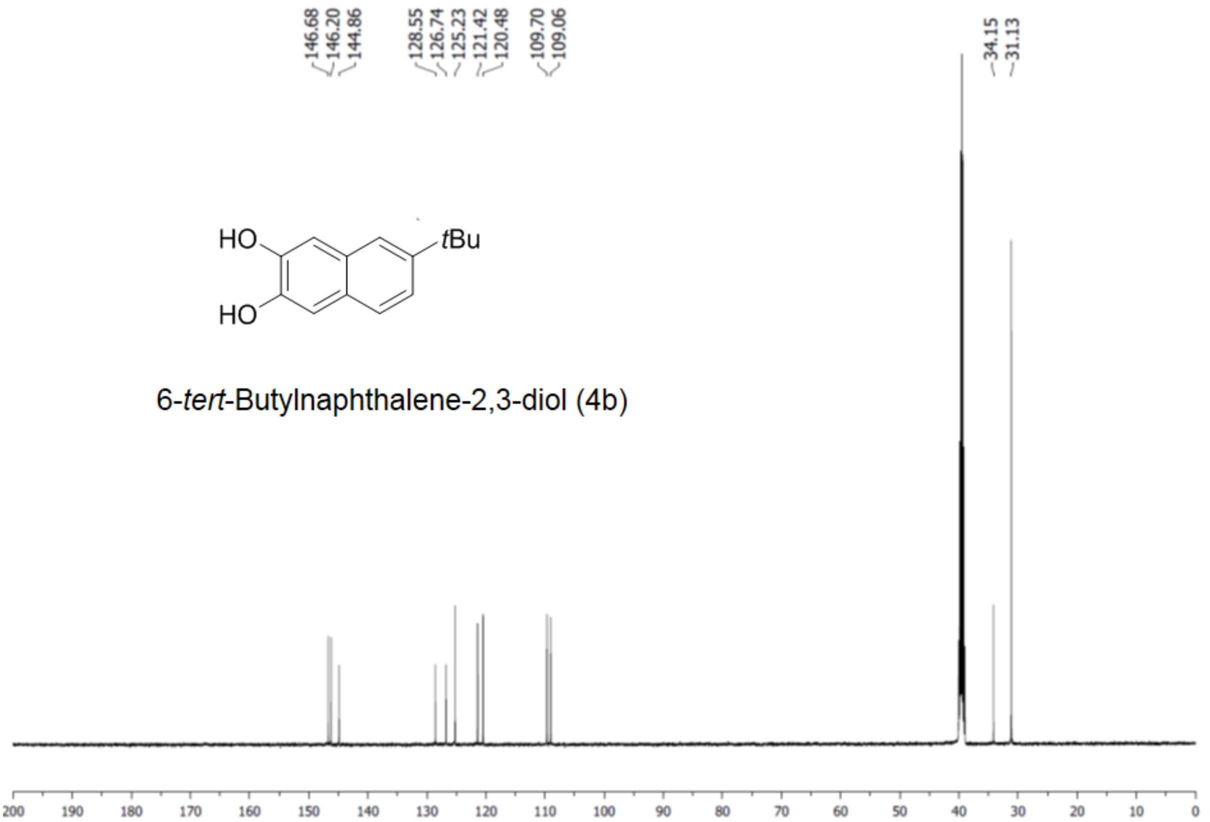

6-*tert*-Butylnaphthalene-2,3-diol (4b)

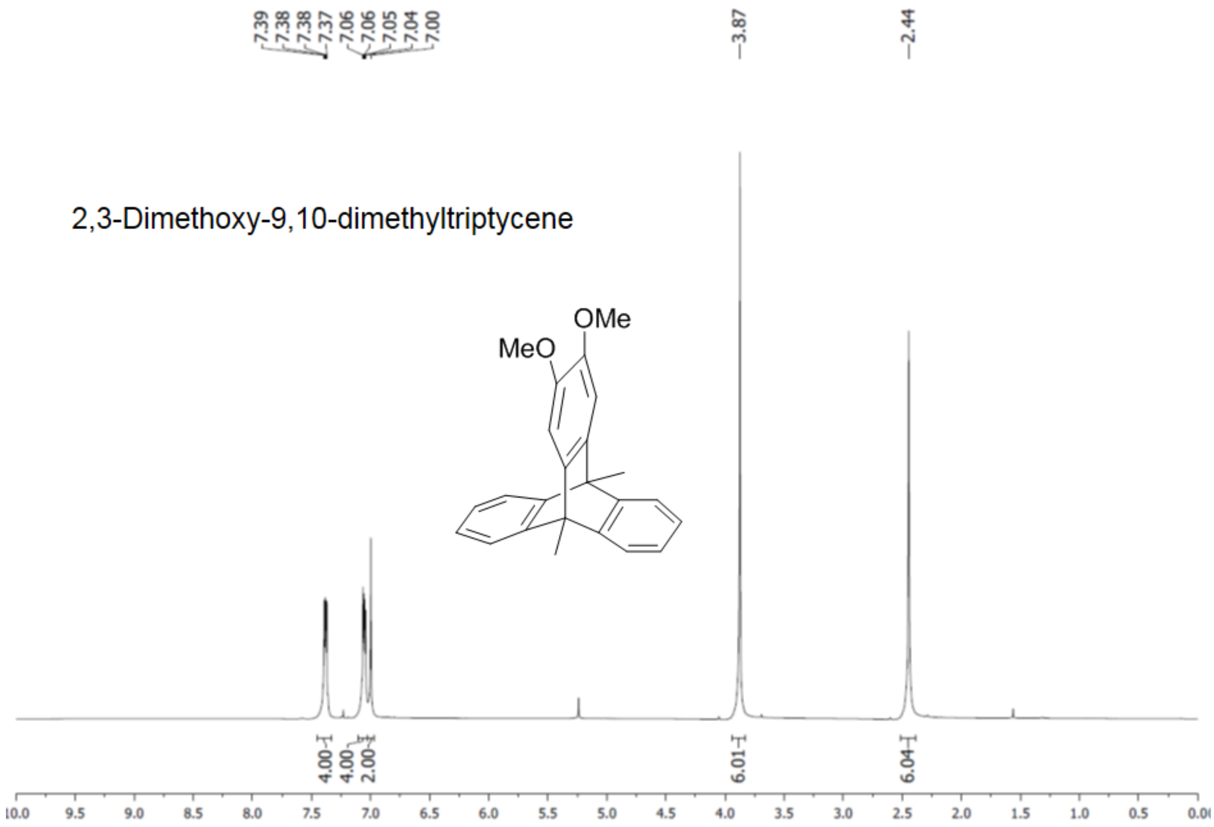
7.39
7.38
7.38
7.37
7.06
7.06
7.05
7.04
7.00
3.87
2.44
2,3-Dimethoxy-9,10-dimethyltriptycene
OMe
MeO
4.00
4.00
2.00
6.01
6.04
10.0
9.5
9.0
8.5
8.0
7.5
7.0
6.5
6.0
5.5
5.0
4.5
4.0
3.5
3.0
2.5
2.0
1.5
1.0
0.5

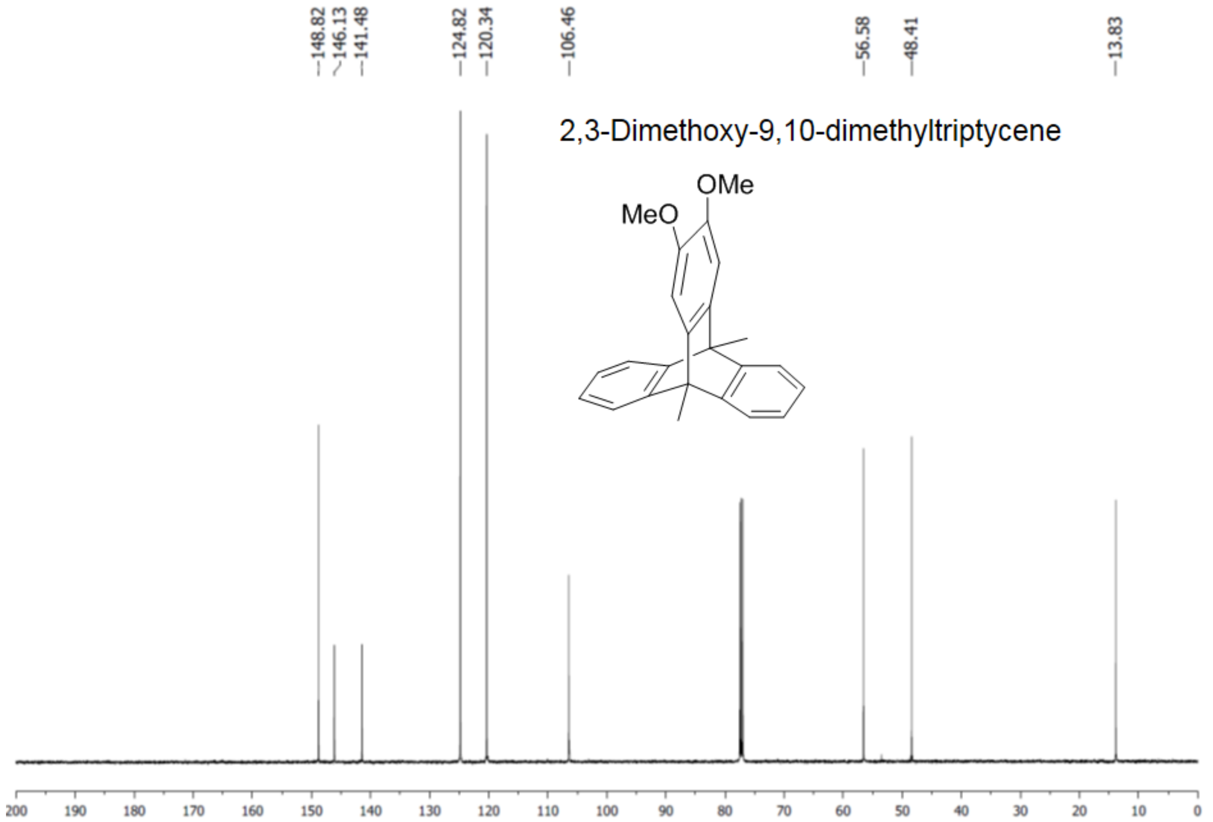
148.82
146.13
141.48
124.82
120.34
106.46
56.58
48.41
13.83
2,3-Dimethoxy-9,10-dimethyltriptycene
OMe
MeO
200
190
180
170
160
150
140
130
120
110
100
90
80
70
60
50
40
30
20
10
0

9,10-Dimethyltriptycene-2,3-diol (5b)

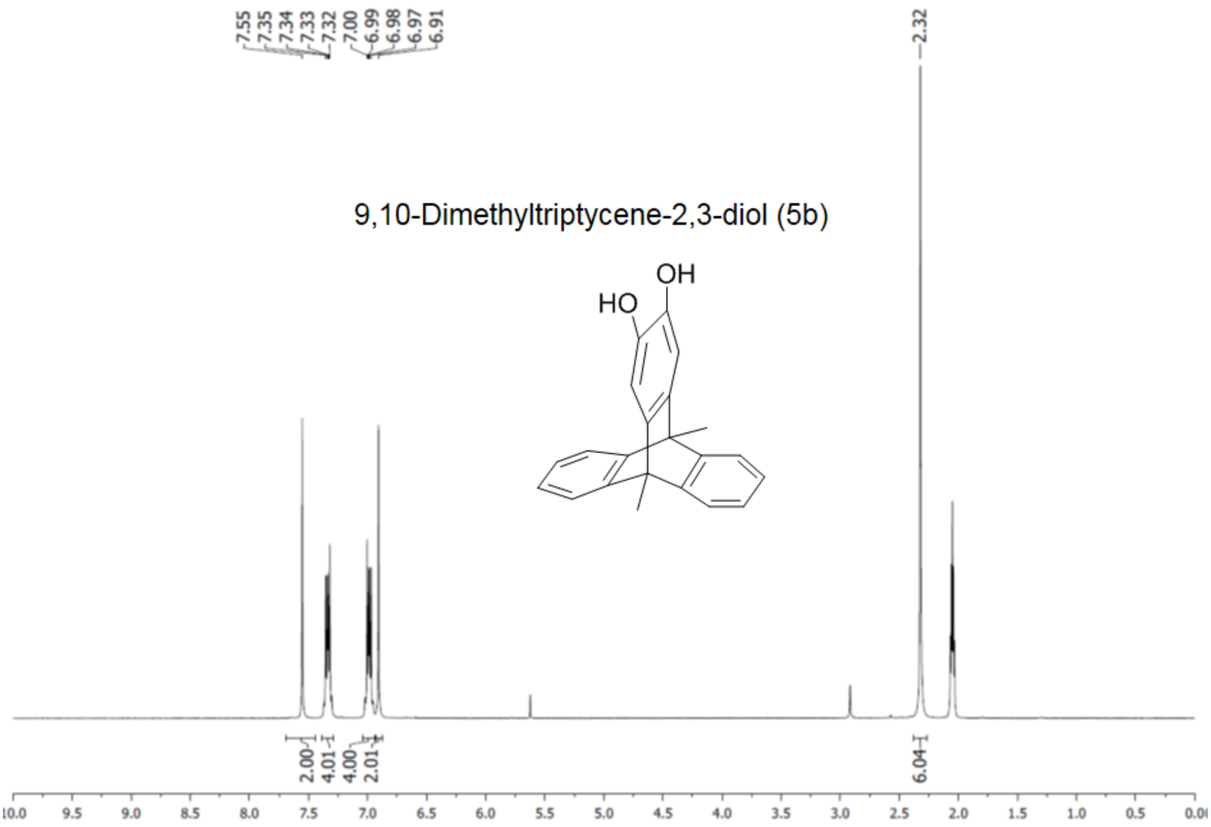

9,10-Dimethyltriptycene-2,3-diol (5b)

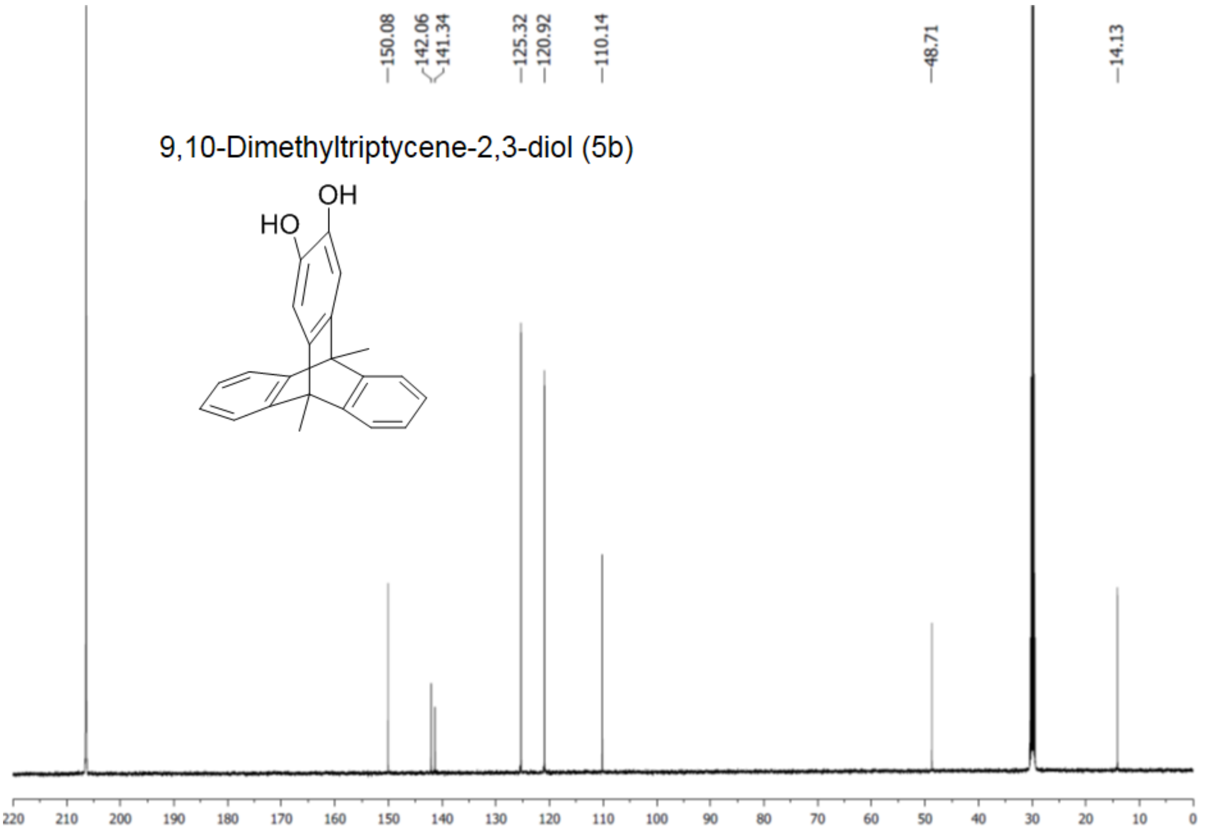

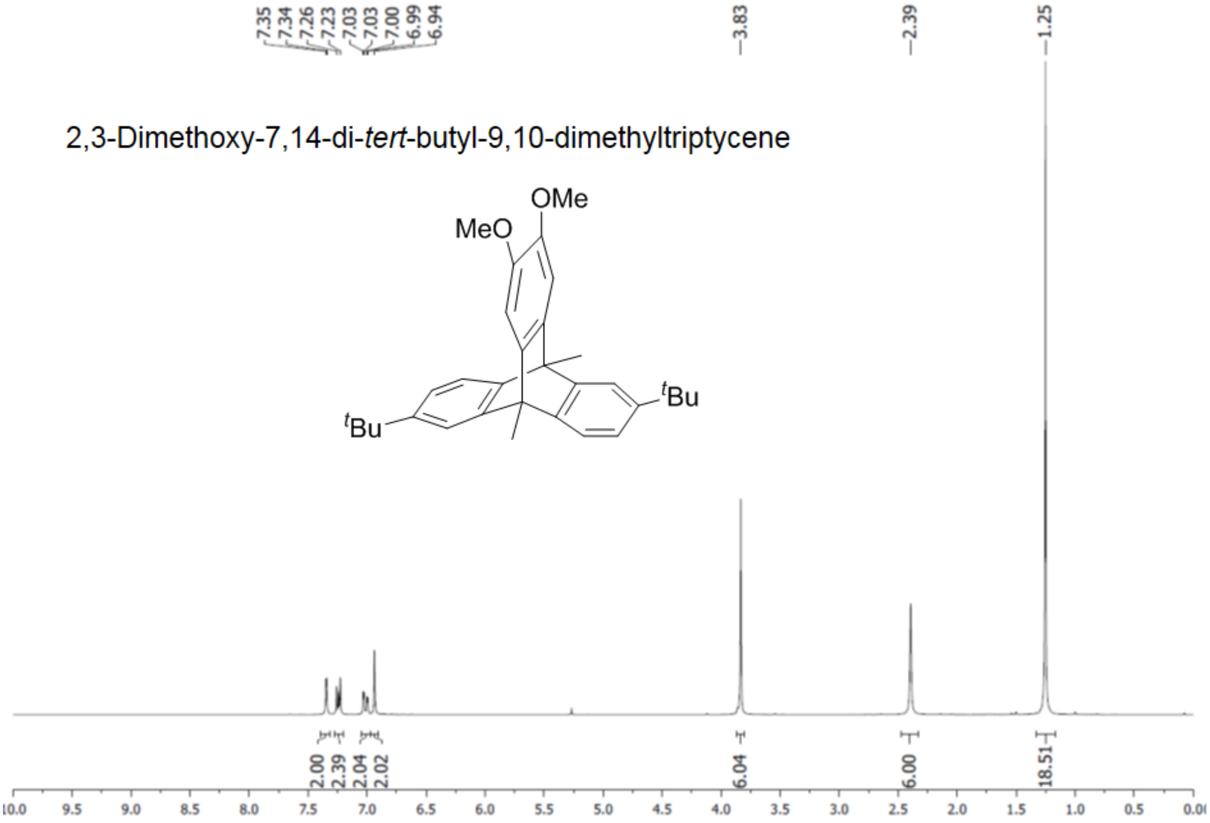
7.35
7.34
7.26
7.23
7.03
7.03
7.00
6.99
6.94
3.83
2.39
1.25
2,3-Dimethoxy-7,14-di-tert-butyl-9,10-dimethyltriptycene
OMe
MeO
tBu
tBu
2.00
2.39
2.04
2.02
6.04
6.00
18.51
10.0
9.5
9.0
8.5
8.0
7.5
7.0
6.5
6.0
5.5
5.0
4.5
4.0
3.5
3.0
2.5
2.0
1.5
1.0
0.5

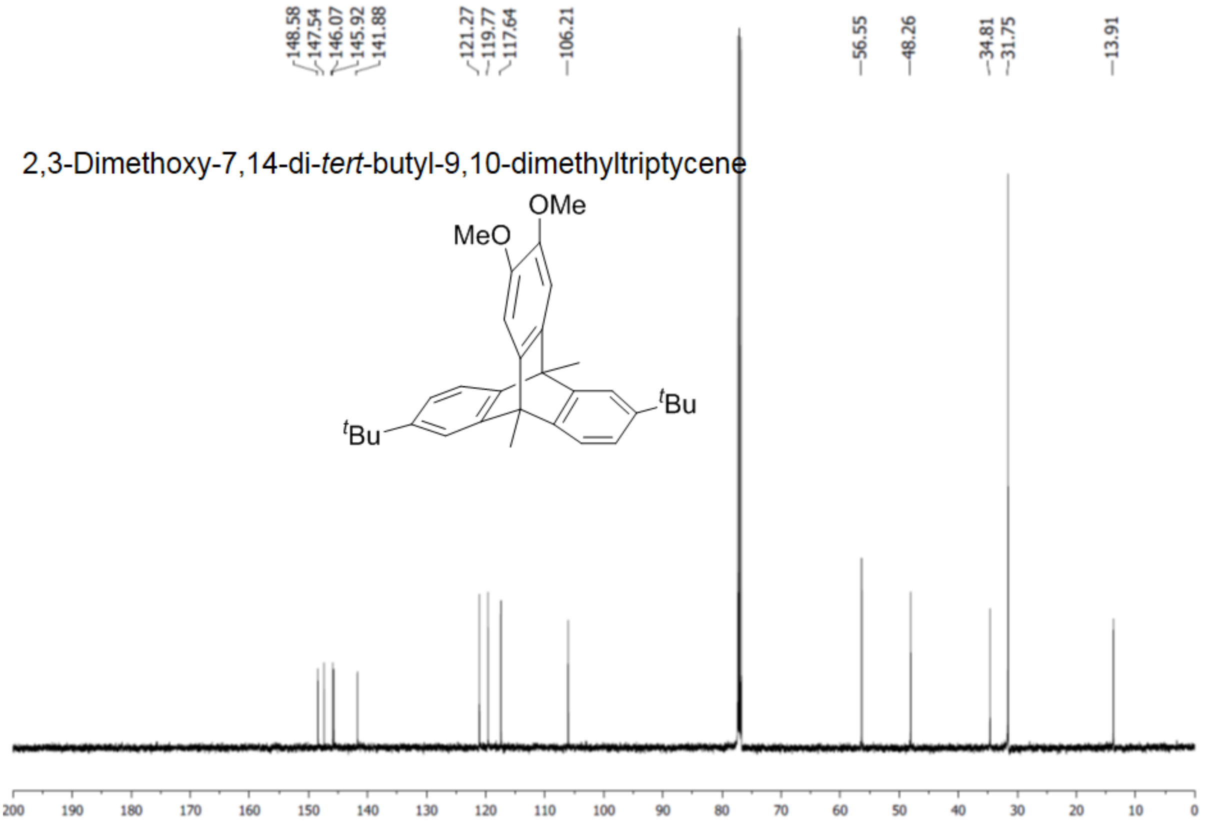
148.58
147.54
146.07
145.92
141.88
121.27
119.77
117.64
106.21
56.55
48.26
34.81
31.75
13.91
2,3-Dimethoxy-7,14-di-tert-butyl-9,10-dimethyltriptycene
OMe
MeO
tBu
tBu
200
190
180
170
160
150
140
130
120
110
100
90
80
70
60
50
40
30
20
10
0

7,14-Di-*tert*-butyl-9,10-dimethyltriptycene-2,3-diol

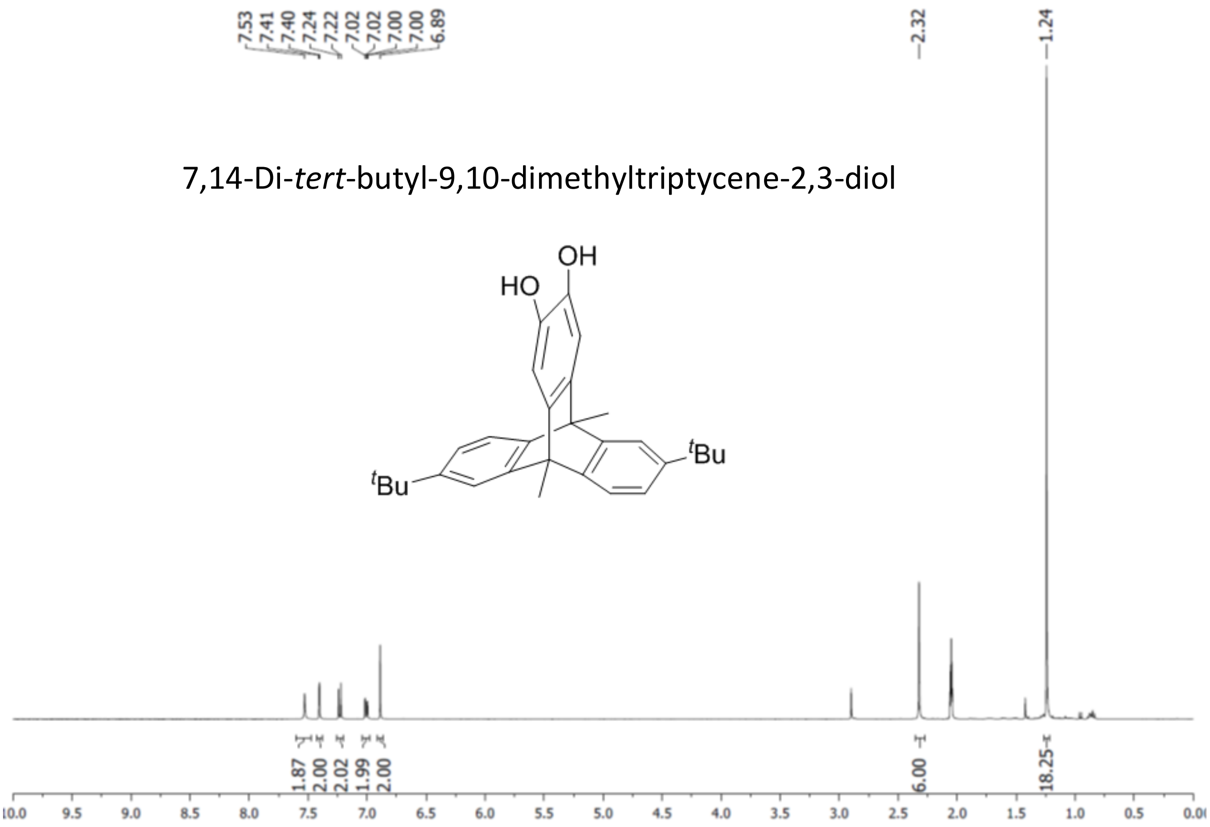

7,14-Di-*tert*-butyl-9,10-dimethyltriptycene-2,3-diol

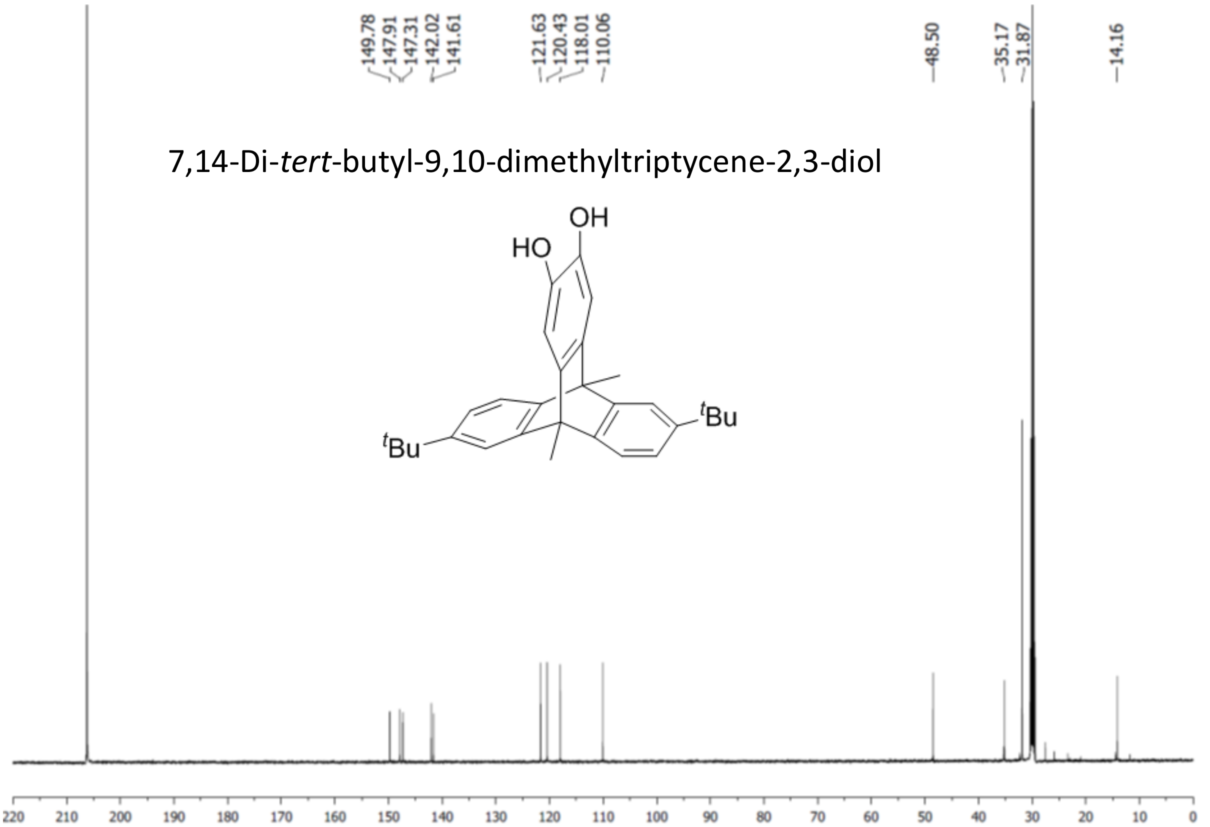

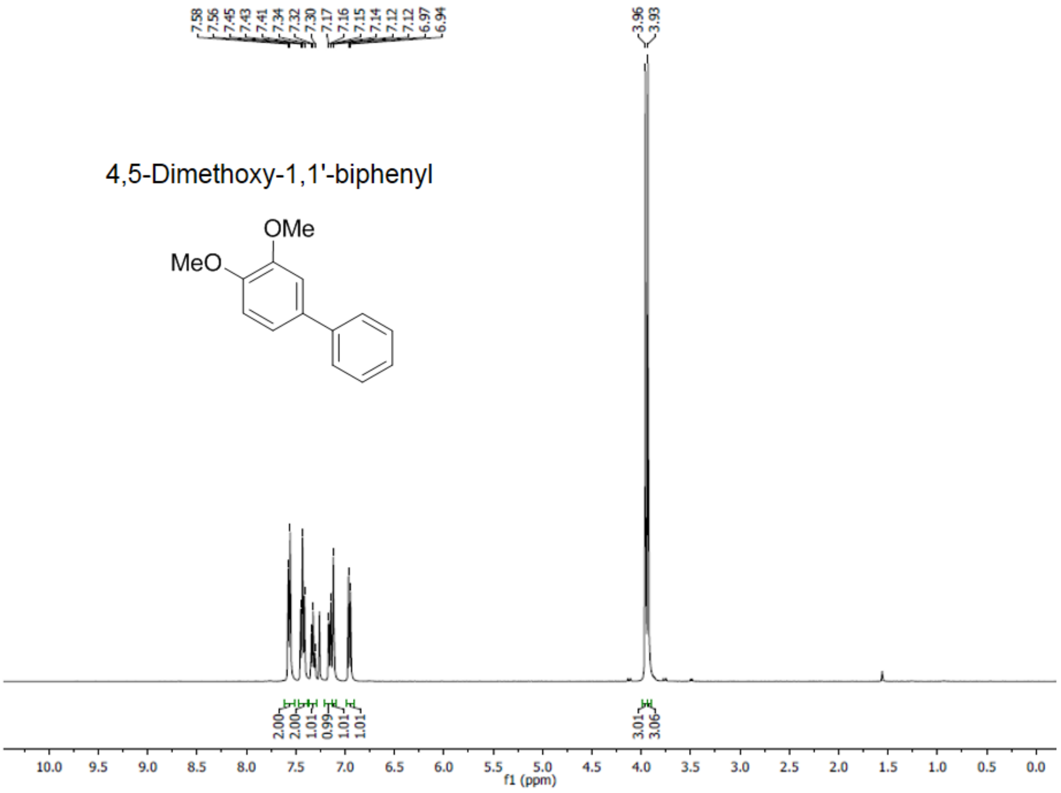
7.58 7.56 7.45 7.43 7.41 7.34 7.32 7.30 7.17 7.16 7.15 7.14 7.12 7.12 6.97 6.94
3.96 3.93
4,5-Dimethoxy-1,1'-biphenyl
OMe
MeO
2.00 2.00 1.01 0.99 1.01 1.01
3.01 3.06
10.0 9.5 9.0 8.5 8.0 7.5 7.0 6.5 6.0 5.5 5.0 4.5 4.0 3.5 3.0 2.5 2.0 1.5 1.0 0.5 0.0
f1 (ppm)

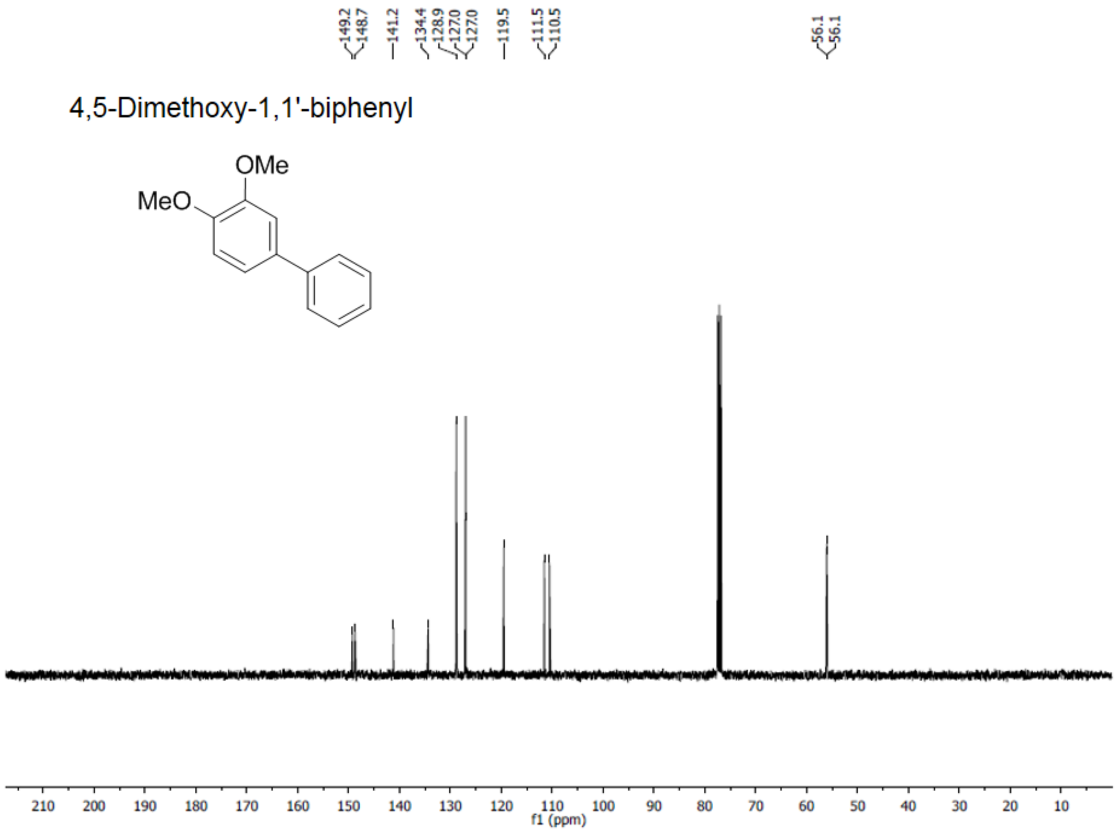
149.2 148.7 141.2 134.4 128.9 127.0 127.0 119.5 111.5 110.5
56.1 56.1
4,5-Dimethoxy-1,1'-biphenyl
OMe
MeO
210 200 190 180 170 160 150 140 130 120 110 100 90 80 70 60 50 40 30 20 10
f1 (ppm)

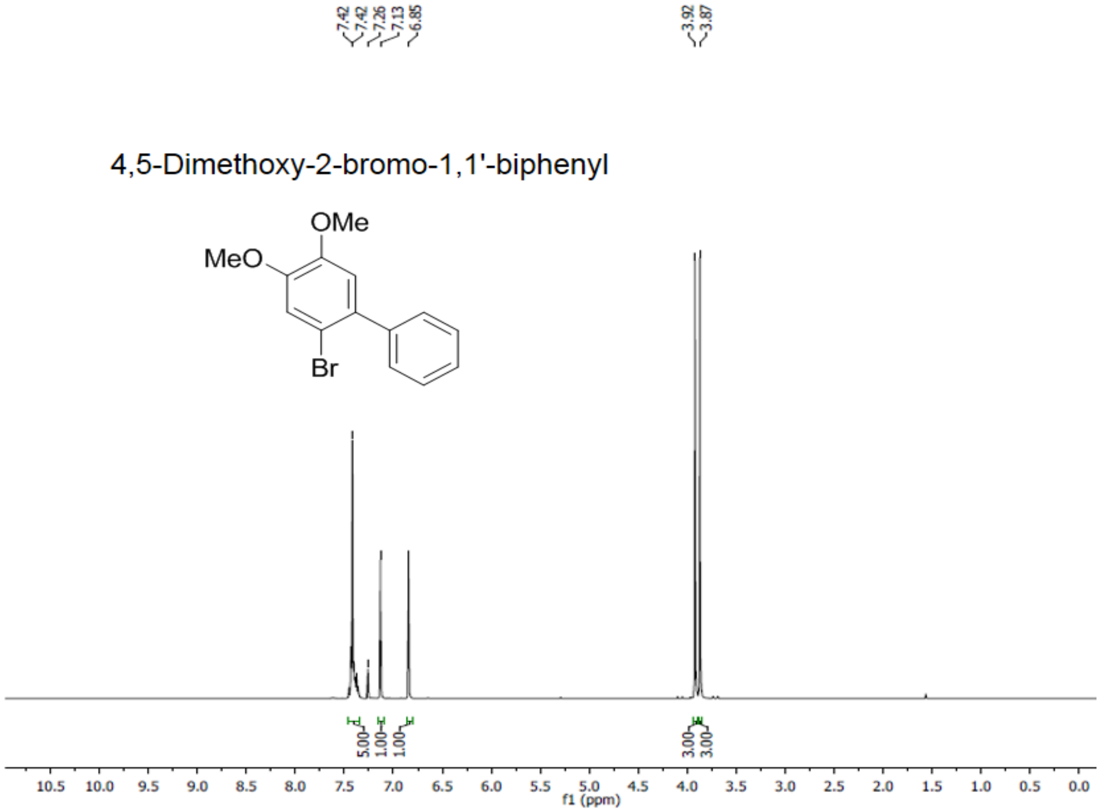
7.42
7.42
7.26
7.13
6.85
3.92
3.87
4,5-Dimethoxy-2-bromo-1,1'-biphenyl
OMe
MeO
Br
5.00
1.00
1.00
3.00
3.00
f1 (ppm)

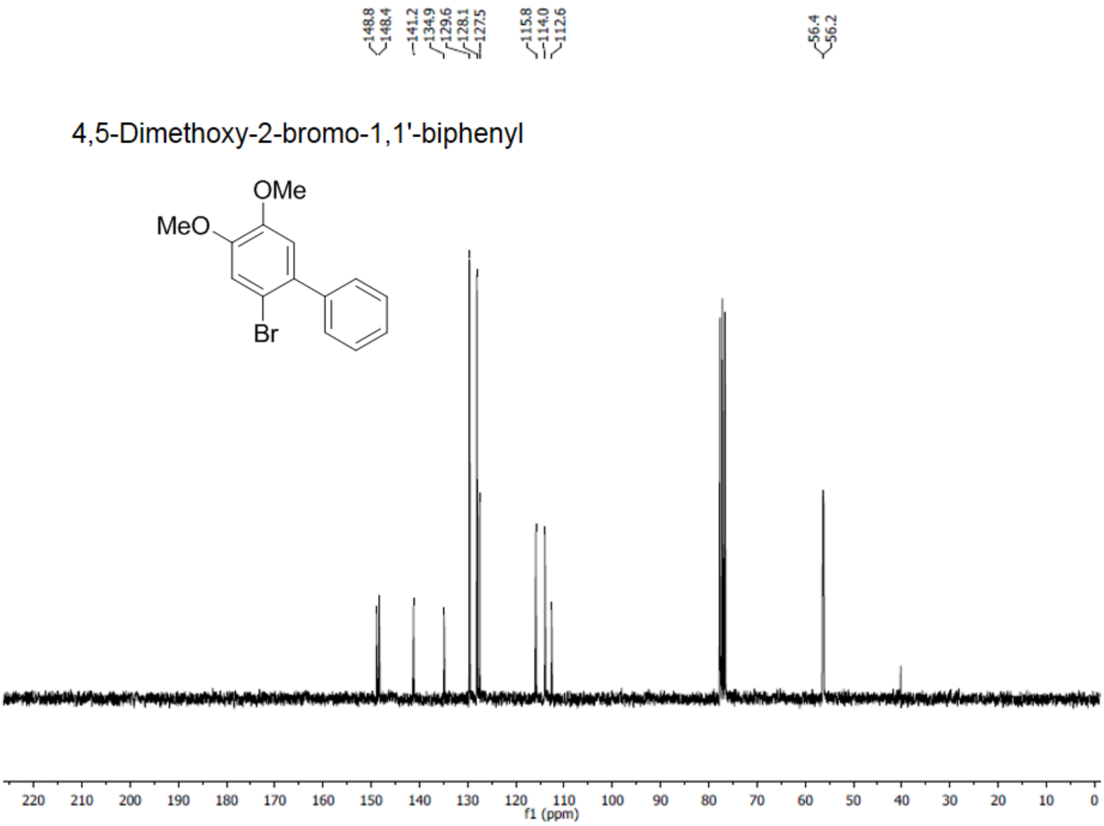
148.8
148.4
141.2
134.9
129.6
128.1
127.5
115.8
114.0
112.6
56.4
56.2
4,5-Dimethoxy-2-bromo-1,1'-biphenyl
OMe
MeO
Br
f1 (ppm)

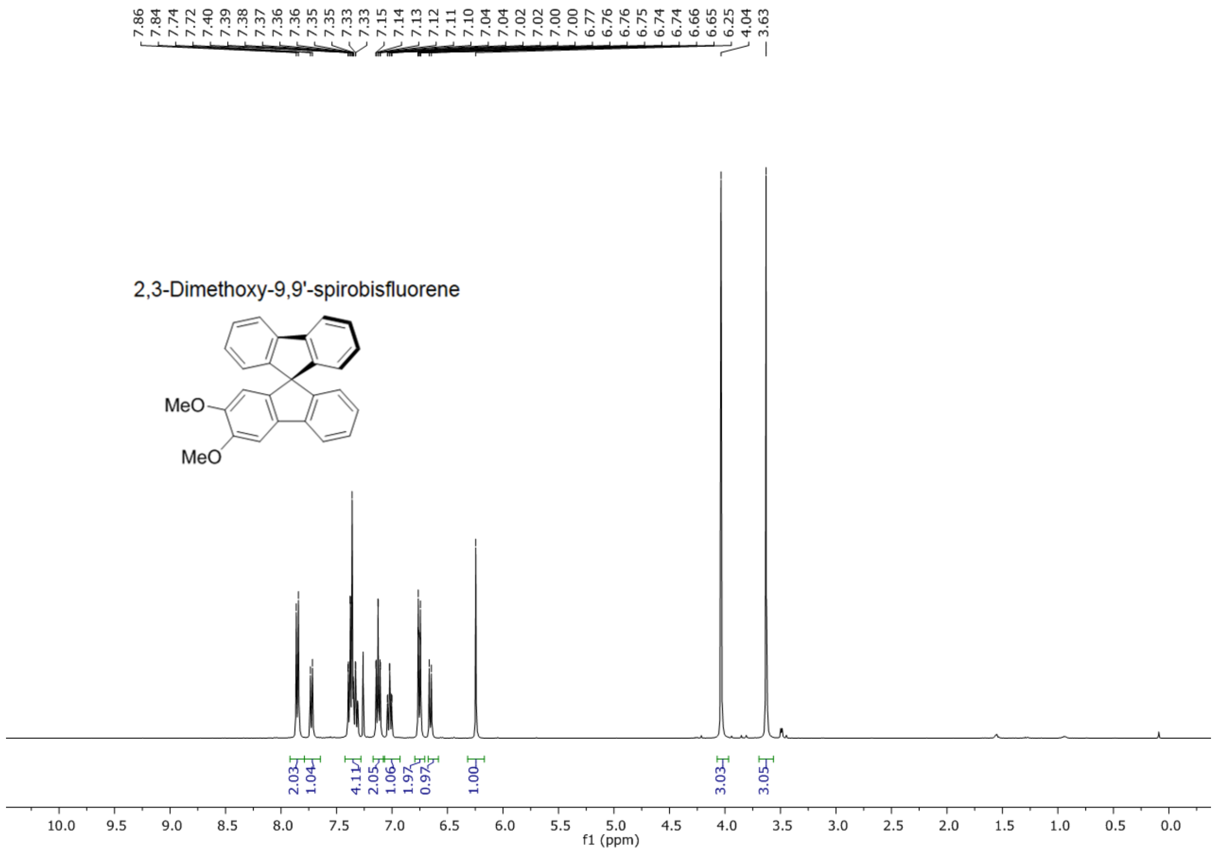
2,3-Dimethoxy-9,9'-spirobisfluorene
MeO
MeO
f1 (ppm)

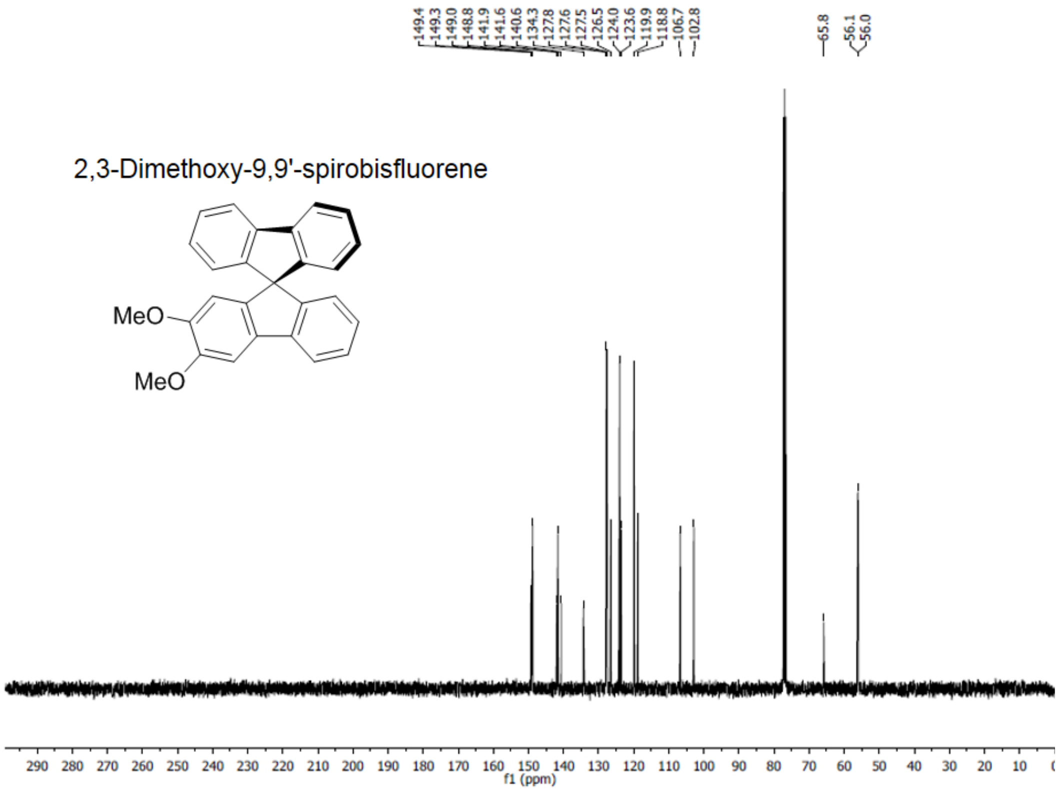
2,3-Dimethoxy-9,9'-spirobisfluorene
MeO
MeO
f1 (ppm)

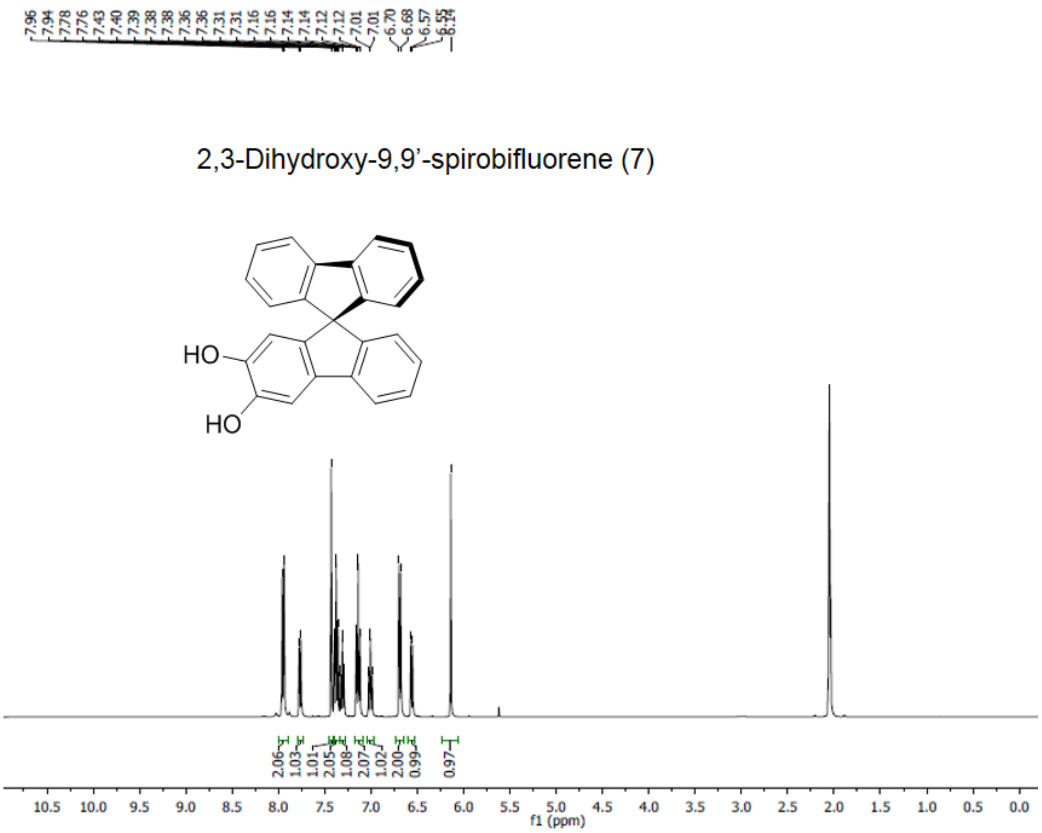
2,3-Dihydroxy-9,9'-spirobifluorene (7)
HO
HO
f1 (ppm)

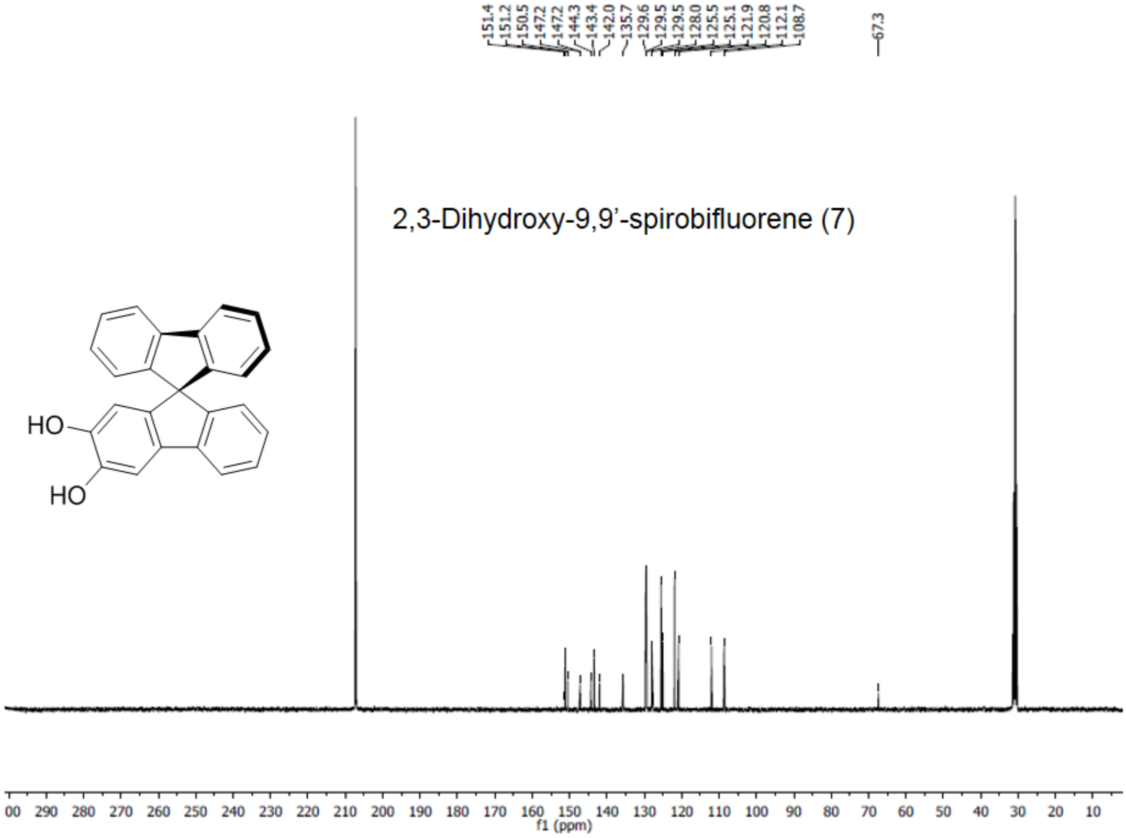
151.4
151.2
150.5
147.2
147.2
144.3
143.4
142.0
135.7
129.6
129.5
129.5
128.0
125.5
125.1
121.9
120.8
112.1
108.7
67.3
2,3-Dihydroxy-9,9'-spirobifluorene (7)
HO
HO
f1 (ppm)

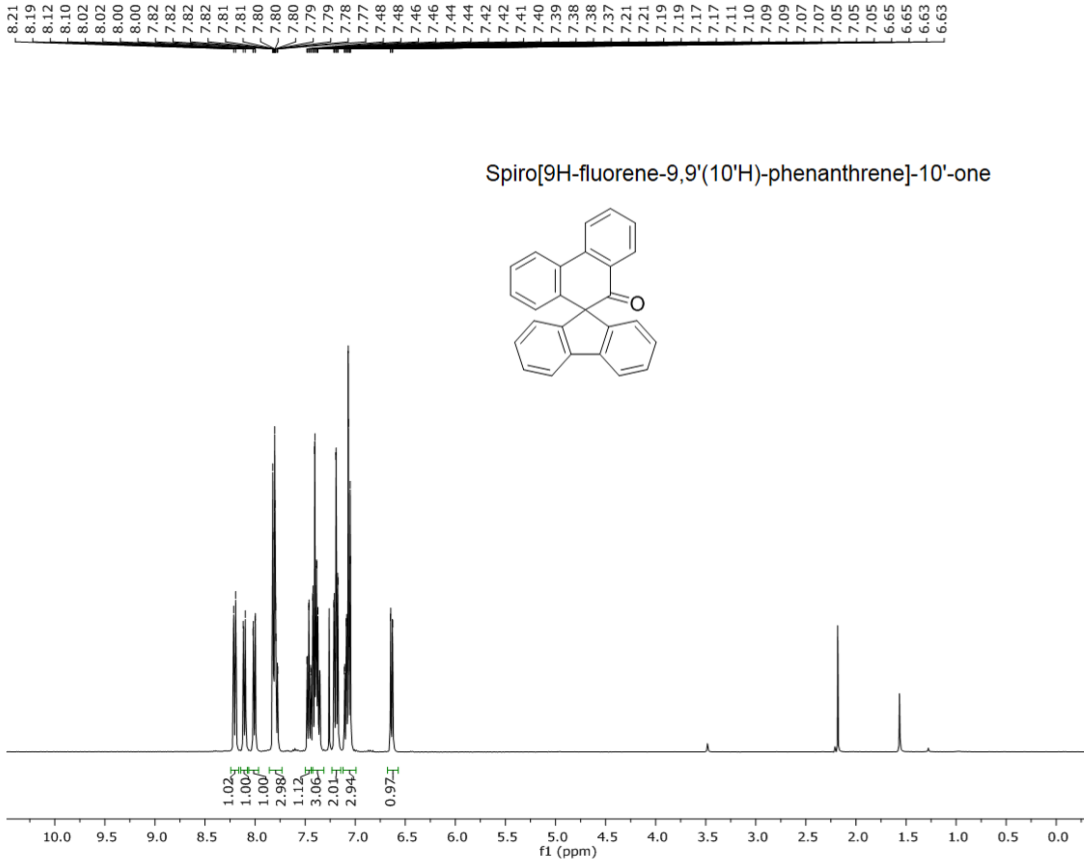
8.21 8.19 8.12 8.10 8.02 8.02 8.00 8.00 7.82 7.82 7.82 7.82 7.81 7.81 7.80 7.80 7.80 7.79 7.79 7.78 7.77 7.48 7.48 7.46 7.46 7.44 7.44 7.42 7.42 7.41 7.40 7.39 7.38 7.38 7.37 7.21 7.21 7.19 7.19 7.17 7.17 7.11 7.10 7.09 7.09 7.07 7.07 7.05 7.05 7.05 6.65 6.65 6.63 6.63
Spiro[9H-fluorene-9,9'(10'H)-phenanthrene]-10'-one
1.02 1.00 1.00 2.98 1.12 3.06 2.01 2.94 0.97
f1 (ppm)

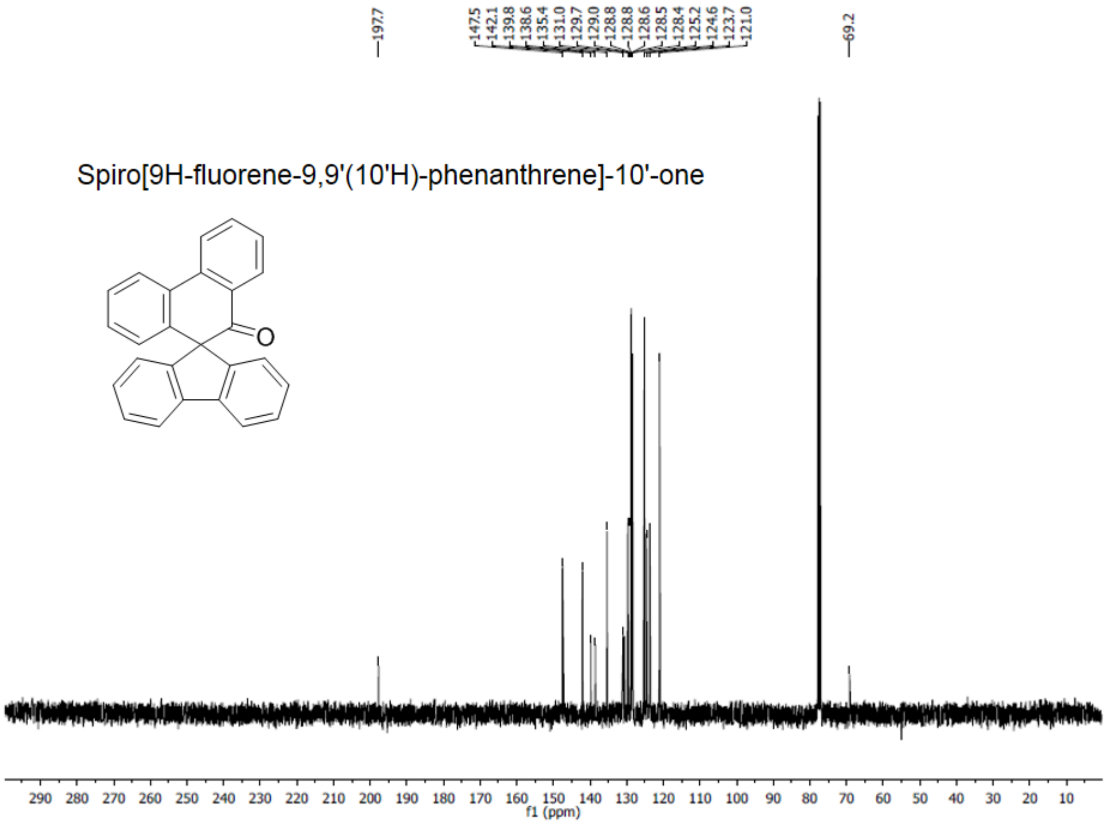
197.7
147.5 142.1 139.8 138.6 135.4 131.0 129.7 129.0 128.8 128.8 128.6 128.5 128.4 125.2 124.6 123.7 121.0
69.2
Spiro[9H-fluorene-9,9'(10'H)-phenanthrene]-10'-one
f1 (ppm)

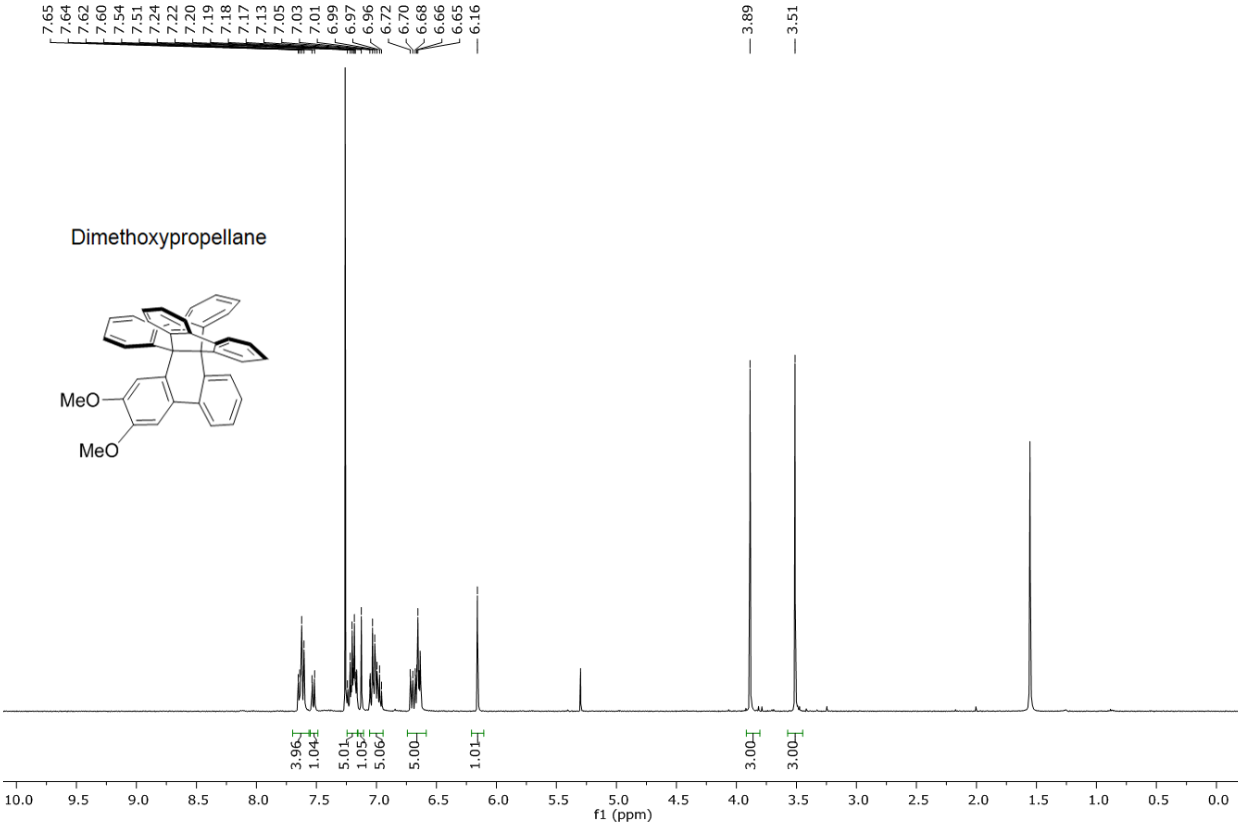

7.65
7.64
7.62
7.60
7.54
7.51
7.24
7.22
7.20
7.19
7.18
7.17
7.13
7.05
7.03
7.01
6.99
6.97
6.96
6.72
6.70
6.68
6.66
6.65
6.16
3.89
3.51
Dimethoxypropellane
MeO
MeO
3.96
1.04
5.01
1.05
5.06
5.00
1.01
3.00
3.00
10.0 9.5 9.0 8.5 8.0 7.5 7.0 6.5 6.0 5.5 5.0 4.5 4.0 3.5 3.0 2.5 2.0 1.5 1.0 0.5 0.0
f1 (ppm)

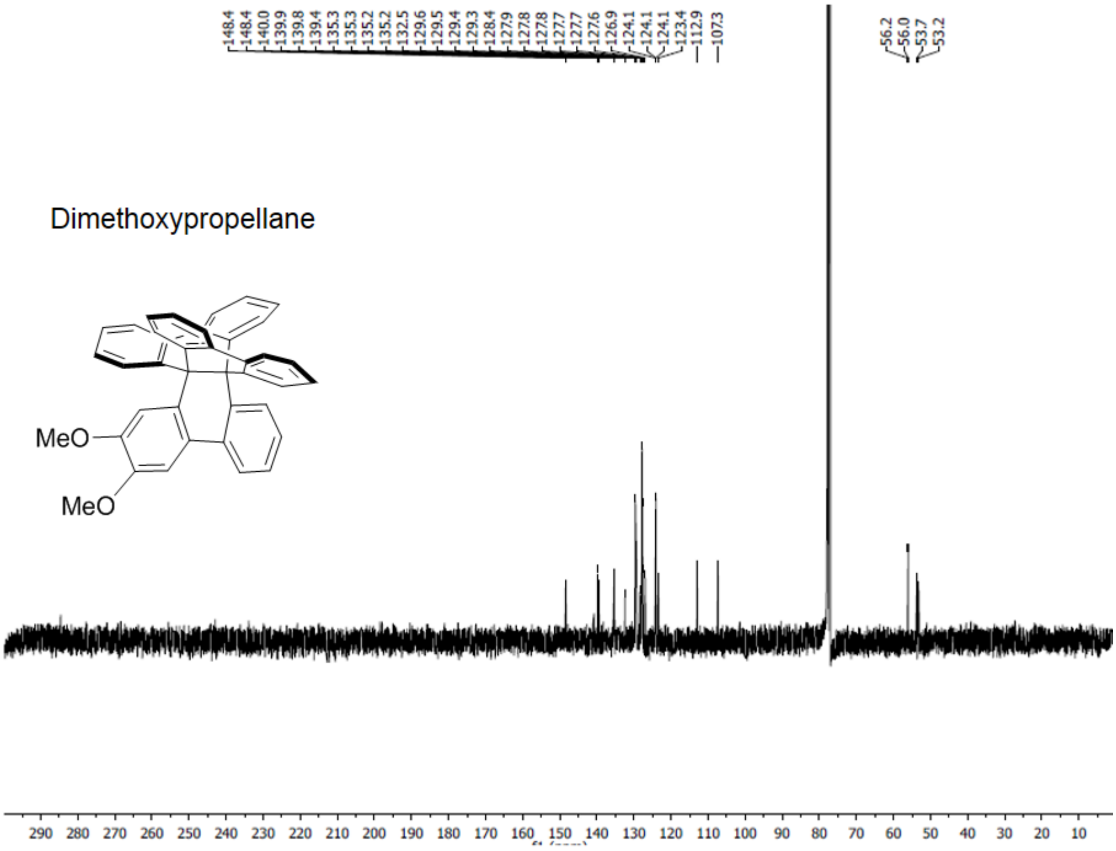

148.4
148.4
140.0
139.9
139.8
139.4
139.4
135.3
135.3
135.2
135.2
132.5
129.6
129.5
129.4
129.3
128.4
127.9
127.8
127.8
127.7
127.7
127.6
126.9
124.1
124.1
124.1
123.4
112.9
107.3
56.2
56.0
53.7
53.2
Dimethoxypropellane
MeO
MeO
290 280 270 260 250 240 230 220 210 200 190 180 170 160 150 140 130 120 110 100 90 80 70 60 50 40 30 20 10

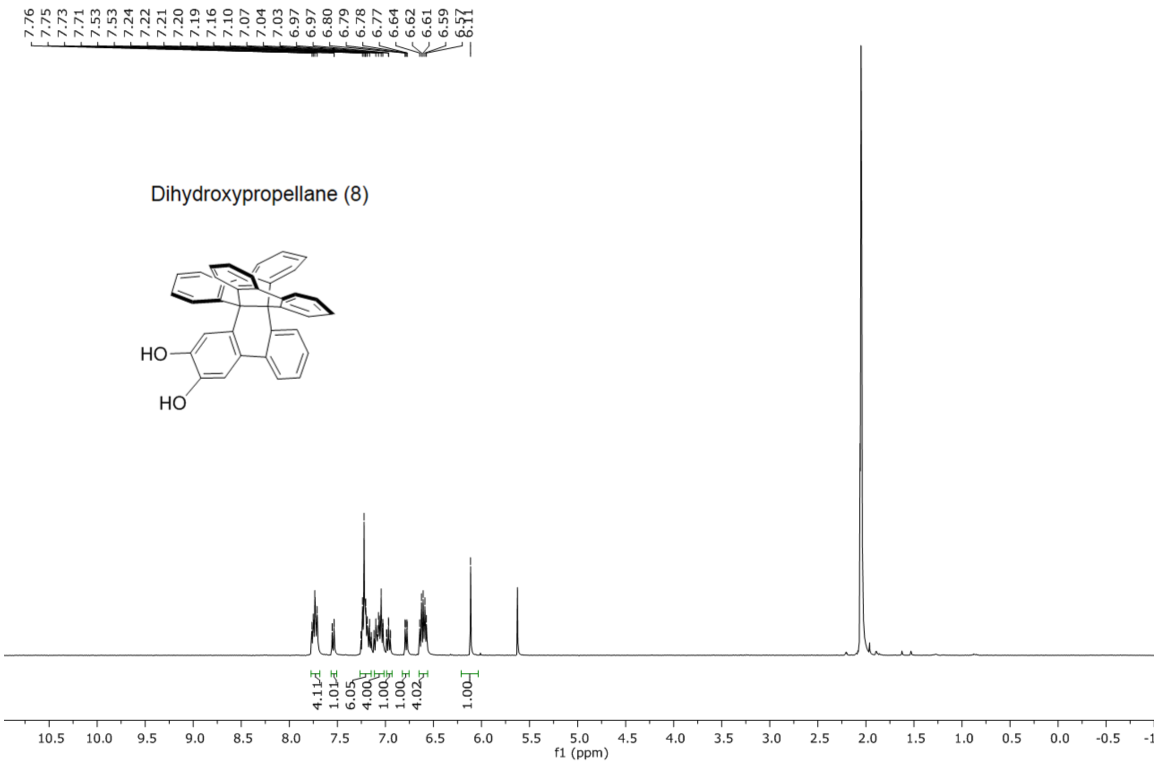

Dihydroxypropellane (8)
HO
HO
f1 (ppm)

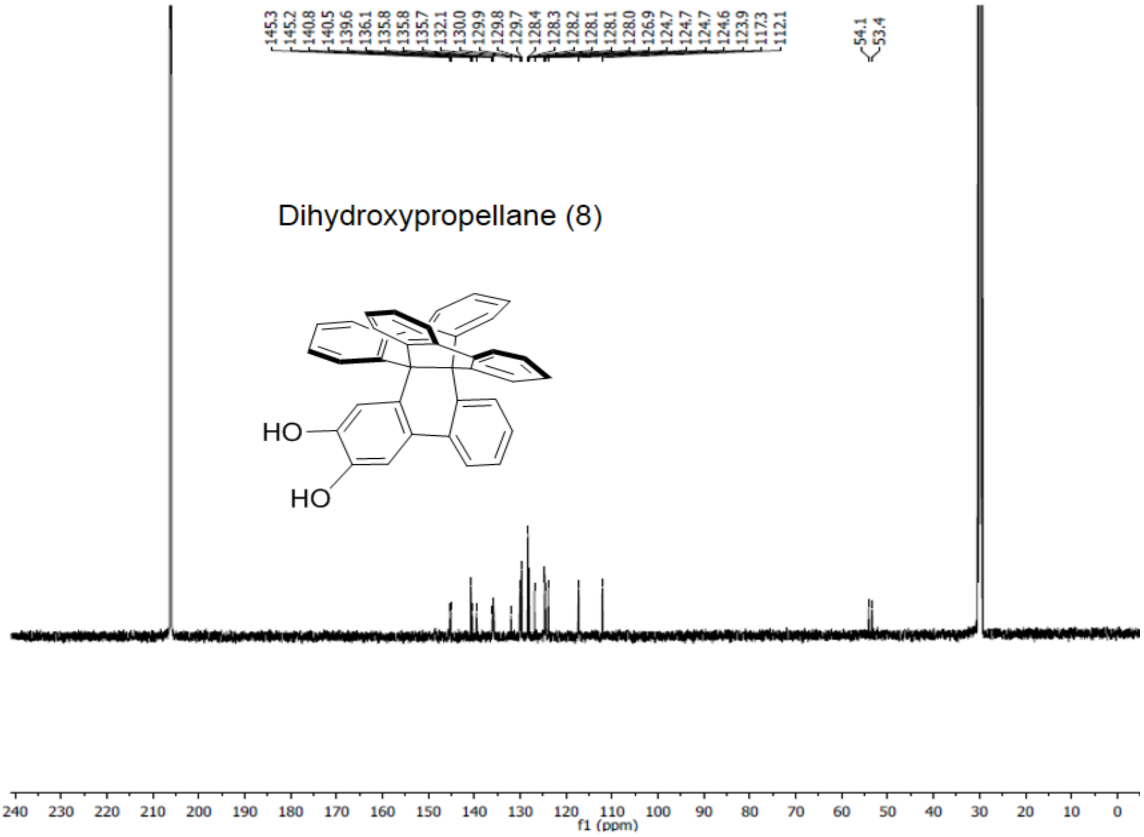

Dihydroxypropellane (8)
HO
HO
f1 (ppm)

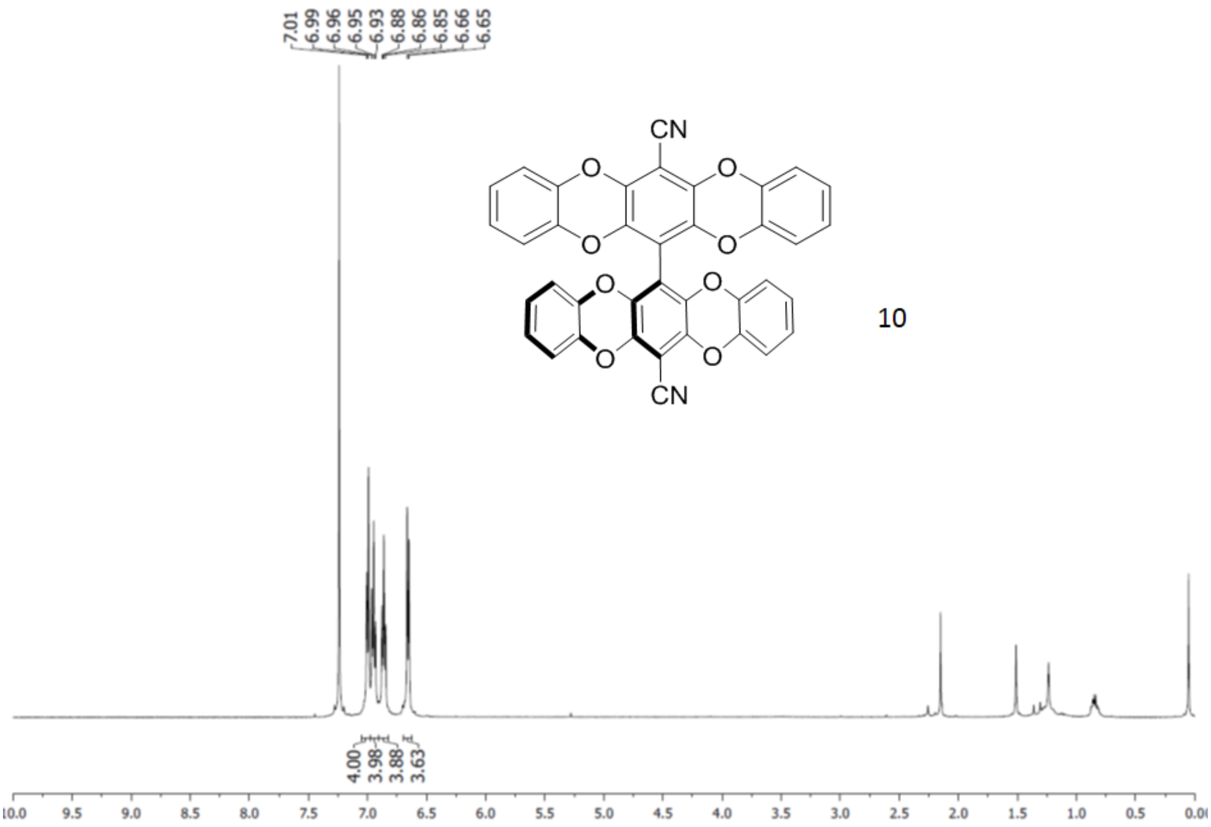
7.01
6.99
6.96
6.95
6.93
6.88
6.86
6.85
6.66
6.65
CN
CN
10
4.00
3.98
3.88
3.63
10.0 9.5 9.0 8.5 8.0 7.5 7.0 6.5 6.0 5.5 5.0 4.5 4.0 3.5 3.0 2.5 2.0 1.5 1.0 0.5 0.00

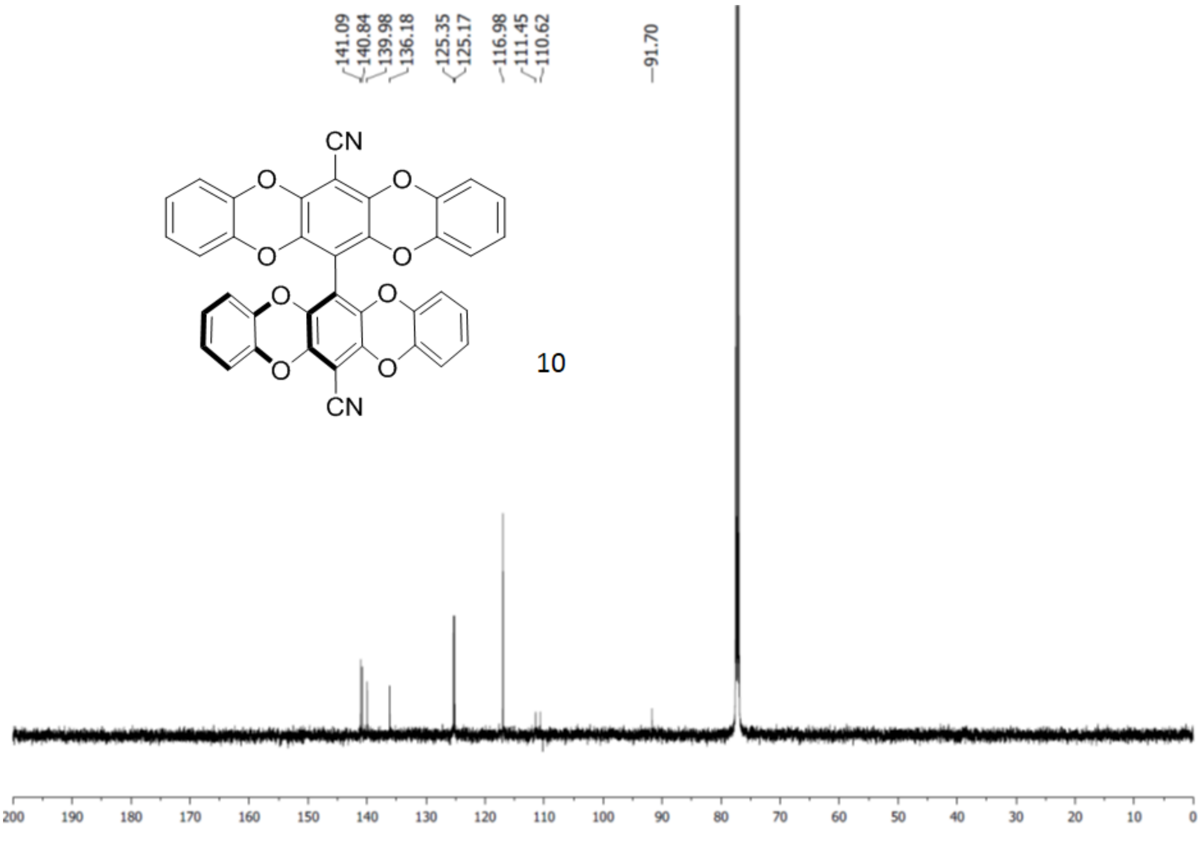
141.09
140.84
139.98
136.18
125.35
125.17
116.98
111.45
110.62
91.70
CN
CN
10
200 190 180 170 160 150 140 130 120 110 100 90 80 70 60 50 40 30 20 10 0

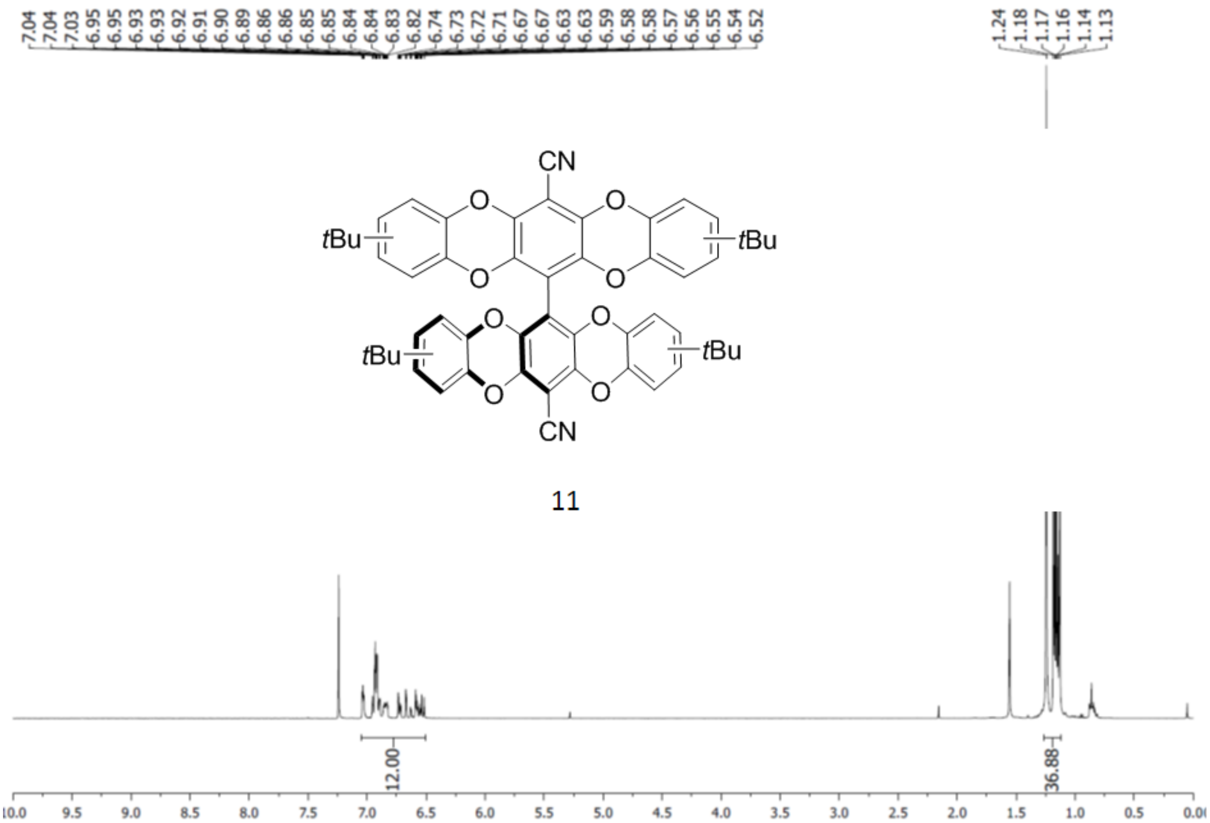
7.04 7.04 7.03 6.95 6.95 6.93 6.93 6.93 6.92 6.91 6.90 6.89 6.86 6.86 6.85 6.85 6.84 6.84 6.83 6.82 6.74 6.73 6.72 6.71 6.67 6.63 6.63 6.59 6.58 6.58 6.57 6.56 6.55 6.54 6.52
1.24 1.18 1.17 1.16 1.14 1.13
CN
O
tBu
CN
12.00
36.88
10.0 9.5 9.0 8.5 8.0 7.5 7.0 6.5 6.0 5.5 5.0 4.5 4.0 3.5 3.0 2.5 2.0 1.5 1.0 0.5 0.0

11

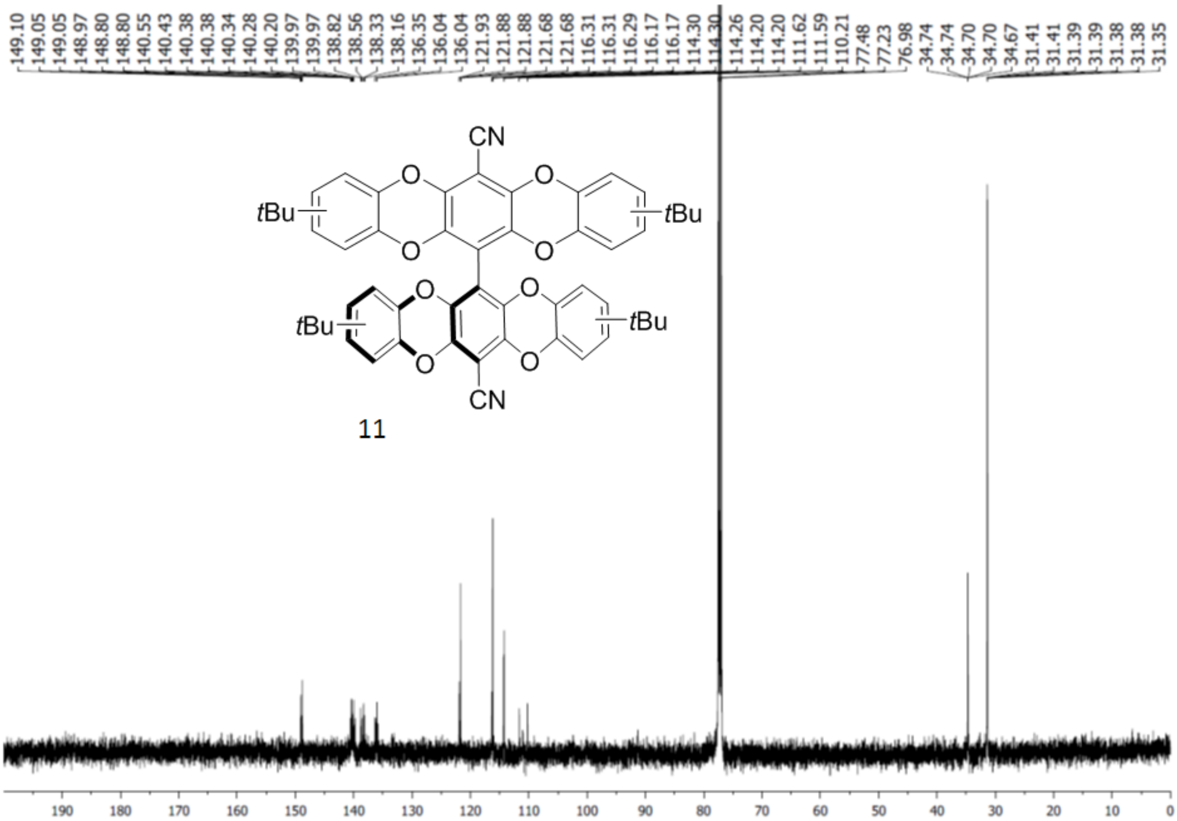
149.10 149.05 149.05 148.97 148.80 148.80 140.55 140.43 140.38 140.38 140.34 140.28 140.20 139.97 139.97 138.82 138.56 138.33 138.16 136.35 136.04 136.04 121.93 121.88 121.88 121.68 121.68 116.31 116.31 116.29 116.17 116.17 114.30 114.30 114.26 114.20 114.20 111.62 111.59 110.21 77.48 77.23 76.98 34.74 34.74 34.70 34.70 34.67 31.41 31.41 31.39 31.39 31.38 31.38 31.35
CN
O
tBu
CN
190 180 170 160 150 140 130 120 110 100 90 80 70 60 50 40 30 20 10 0

11

12

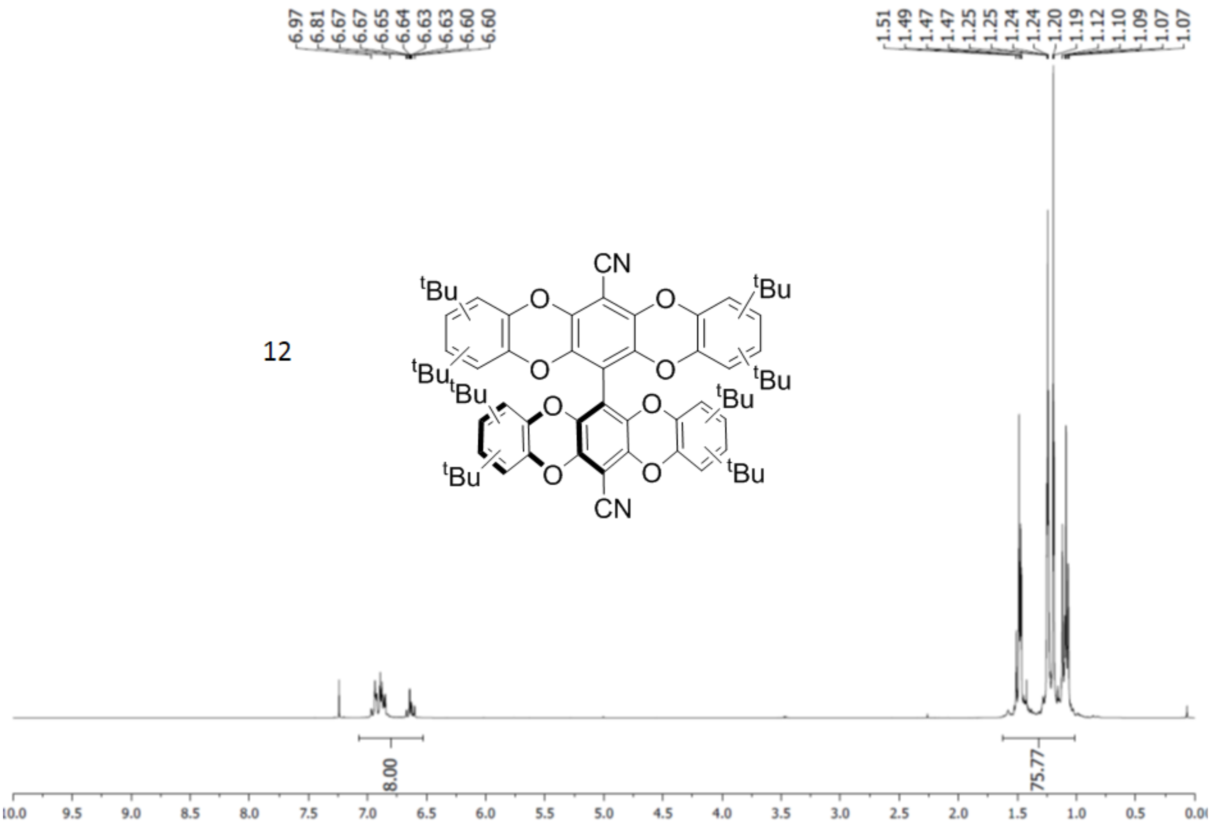

6.97 6.81 6.67 6.67 6.65 6.64 6.63 6.63 6.60 6.60
1.51 1.49 1.47 1.47 1.25 1.25 1.24 1.24 1.20 1.19 1.12 1.10 1.09 1.07 1.07
CN
O
tBu
8.00
75.77
10.0 9.5 9.0 8.5 8.0 7.5 7.0 6.5 6.0 5.5 5.0 4.5 4.0 3.5 3.0 2.5 2.0 1.5 1.0 0.5 0.00

12

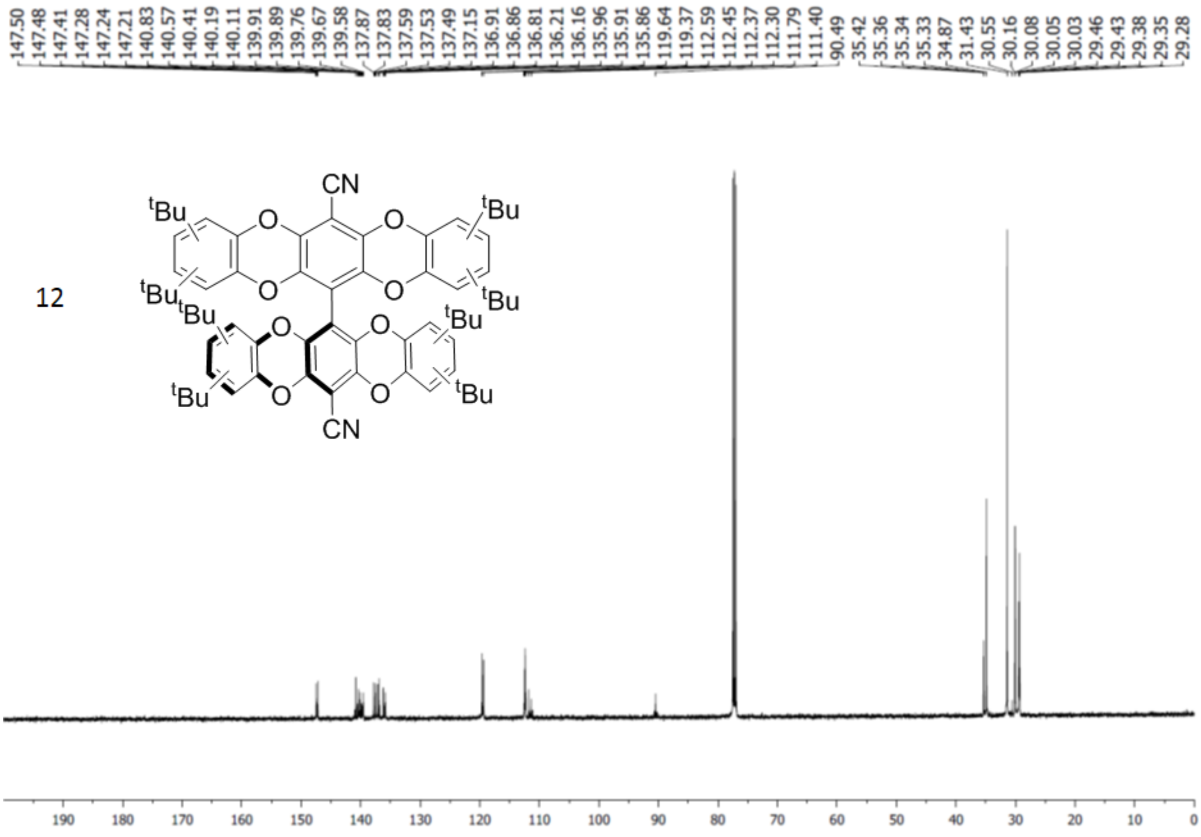

147.50 147.48 147.41 147.28 147.24 147.21 140.83 140.57 140.41 140.19 140.11 139.91 139.89 139.76 139.67 139.58 137.87 137.83 137.59 137.53 137.49 137.15 136.91 136.86 136.81 136.21 136.16 135.96 135.91 135.86 119.64 119.37 112.59 112.45 112.37 112.30 111.79 111.40 90.49
35.42 35.36 35.34 35.33 34.87 31.43 30.55 30.16 30.08 30.05 30.03 29.46 29.43 29.38 29.35 29.28
CN
O
tBu
190 180 170 160 150 140 130 120 110 100 90 80 70 60 50 40 30 20 10 0

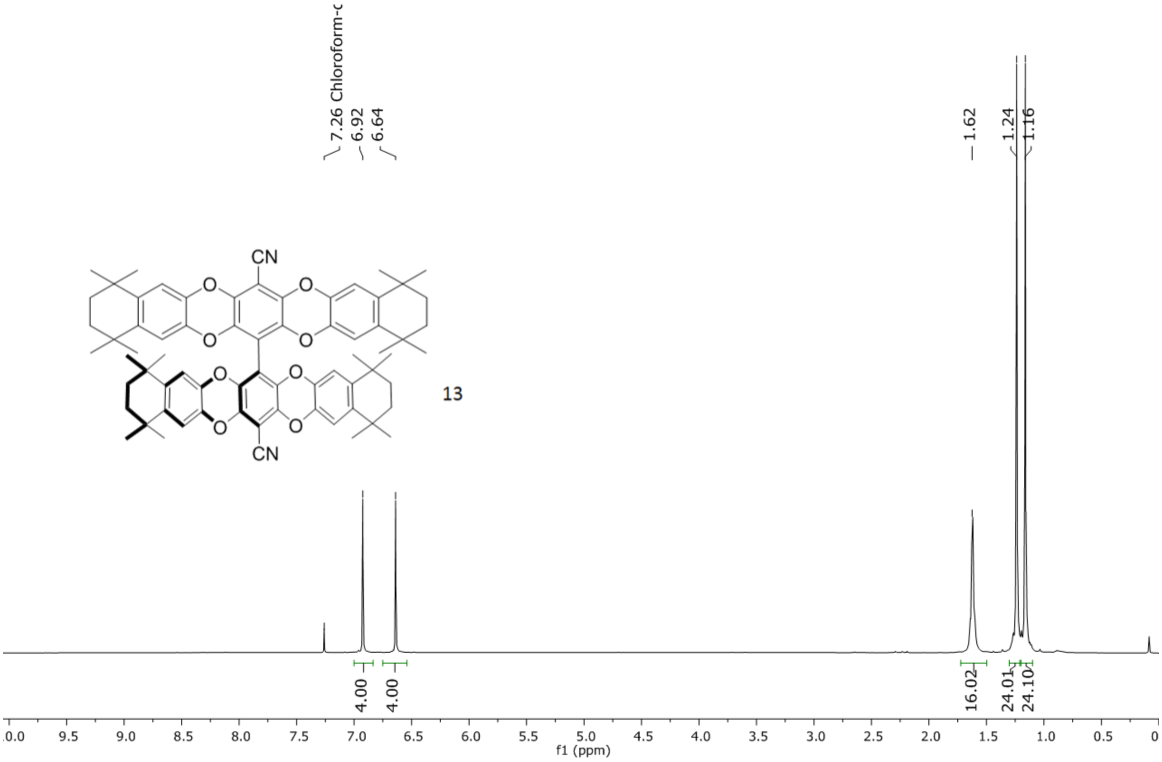
7.26 Chloroform-d
6.92
6.64
1.62
1.24
1.16
CN
O
CN
13
4.00
4.00
16.02
24.01
24.10
f1 (ppm)

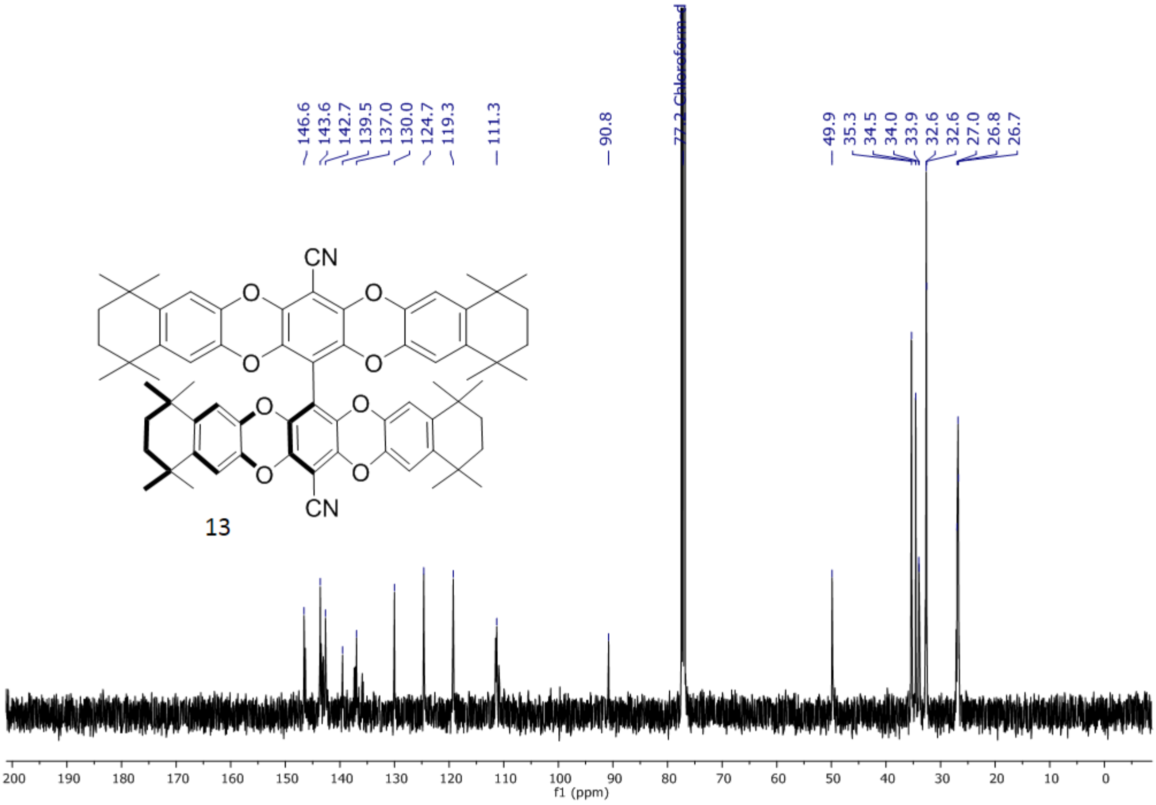
146.6
143.6
142.7
139.5
137.0
130.0
124.7
119.3
111.3
90.8
77.2 Chloroform-d
49.9
35.3
34.5
34.0
33.9
32.6
32.6
27.0
26.8
26.7
CN
O
CN
13
f1 (ppm)

17

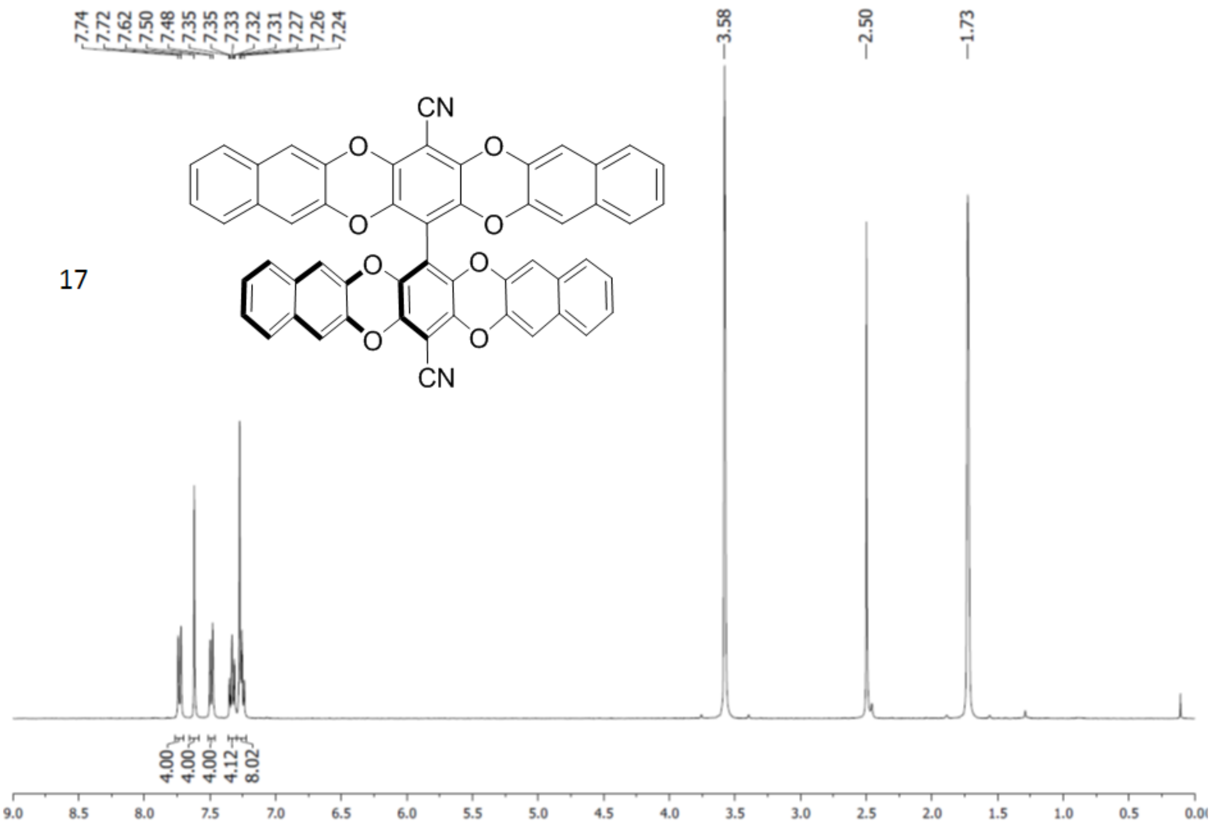
7.74
7.72
7.62
7.50
7.48
7.35
7.35
7.33
7.32
7.31
7.27
7.26
7.24
3.58
2.50
1.73
CN
O
CN
4.00
4.00
4.00
4.12
8.02
9.0 8.5 8.0 7.5 7.0 6.5 6.0 5.5 5.0 4.5 4.0 3.5 3.0 2.5 2.0 1.5 1.0 0.5

17

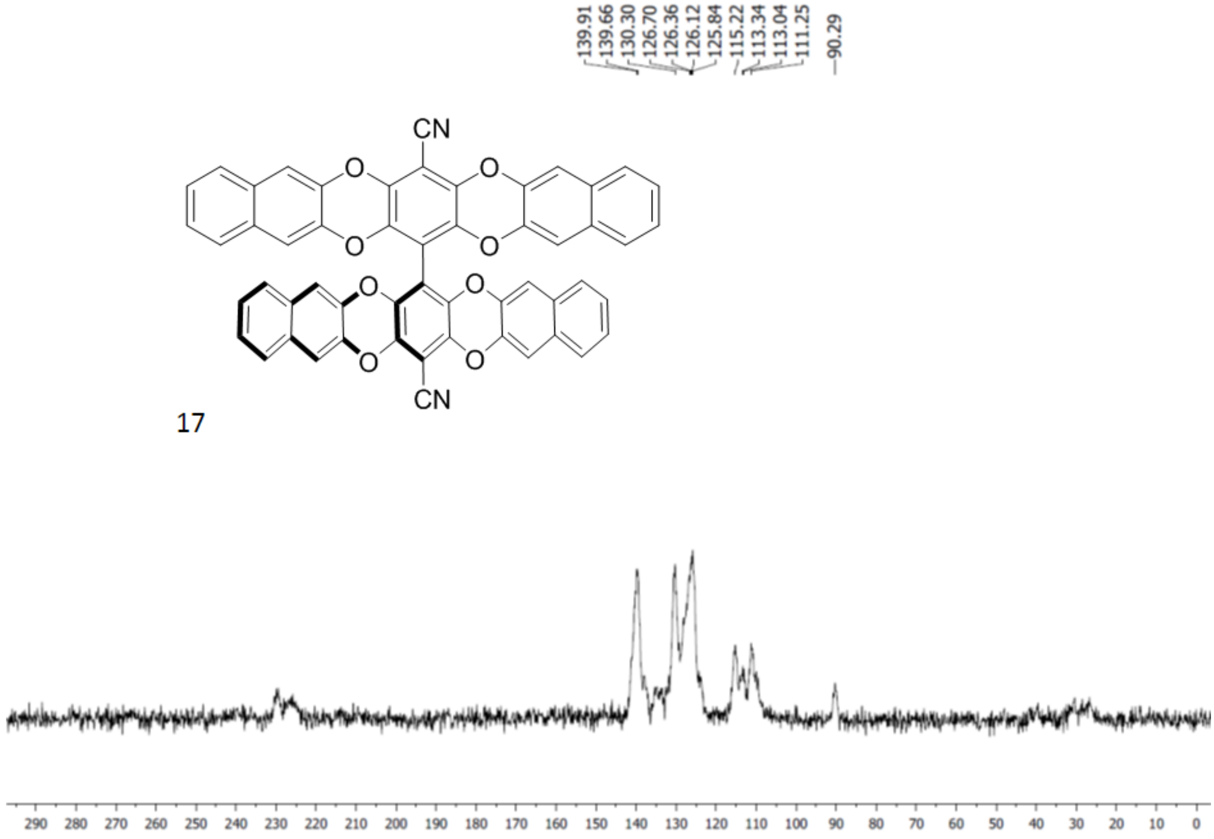
139.91
139.66
130.30
126.70
126.36
126.12
125.84
115.22
113.34
113.04
111.25
90.29
CN
O
CN
290 280 270 260 250 240 230 220 210 200 190 180 170 160 150 140 130 120 110 100 90 80 70 60 50 40 30 20 10 0

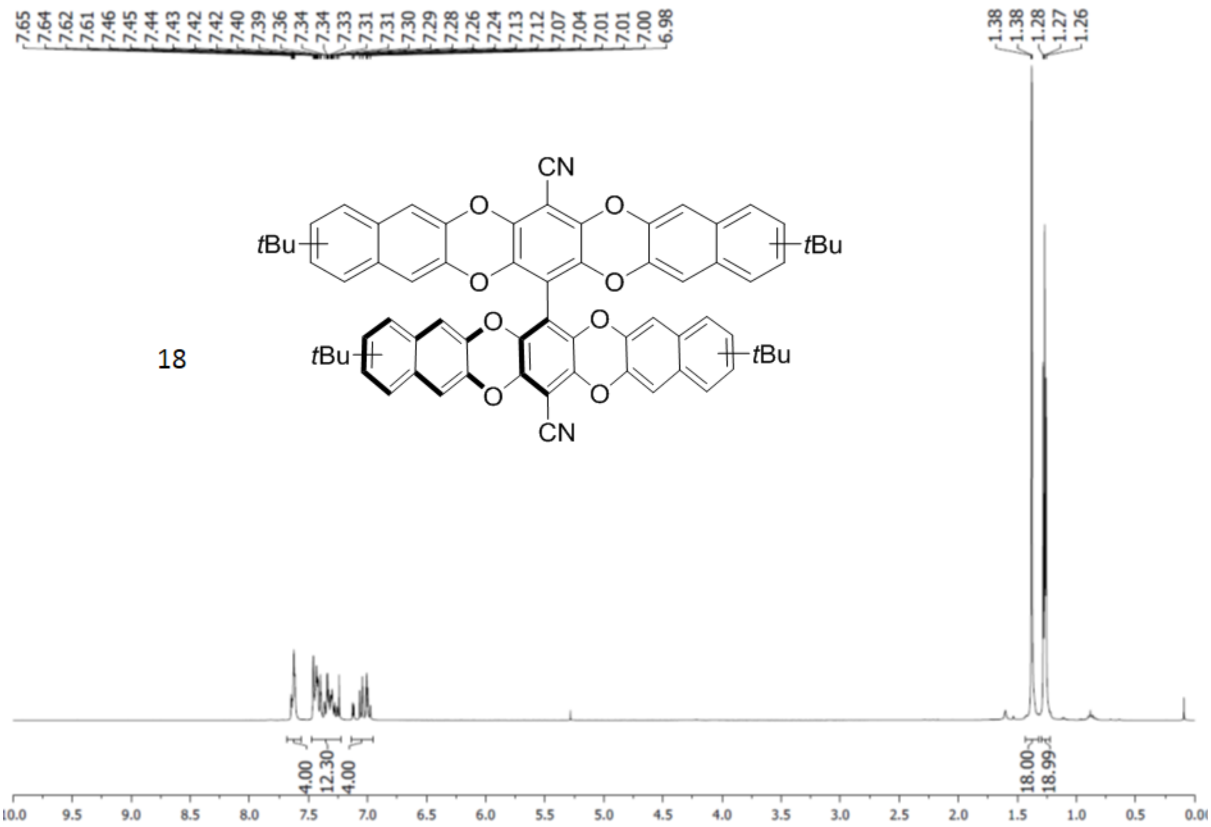

18

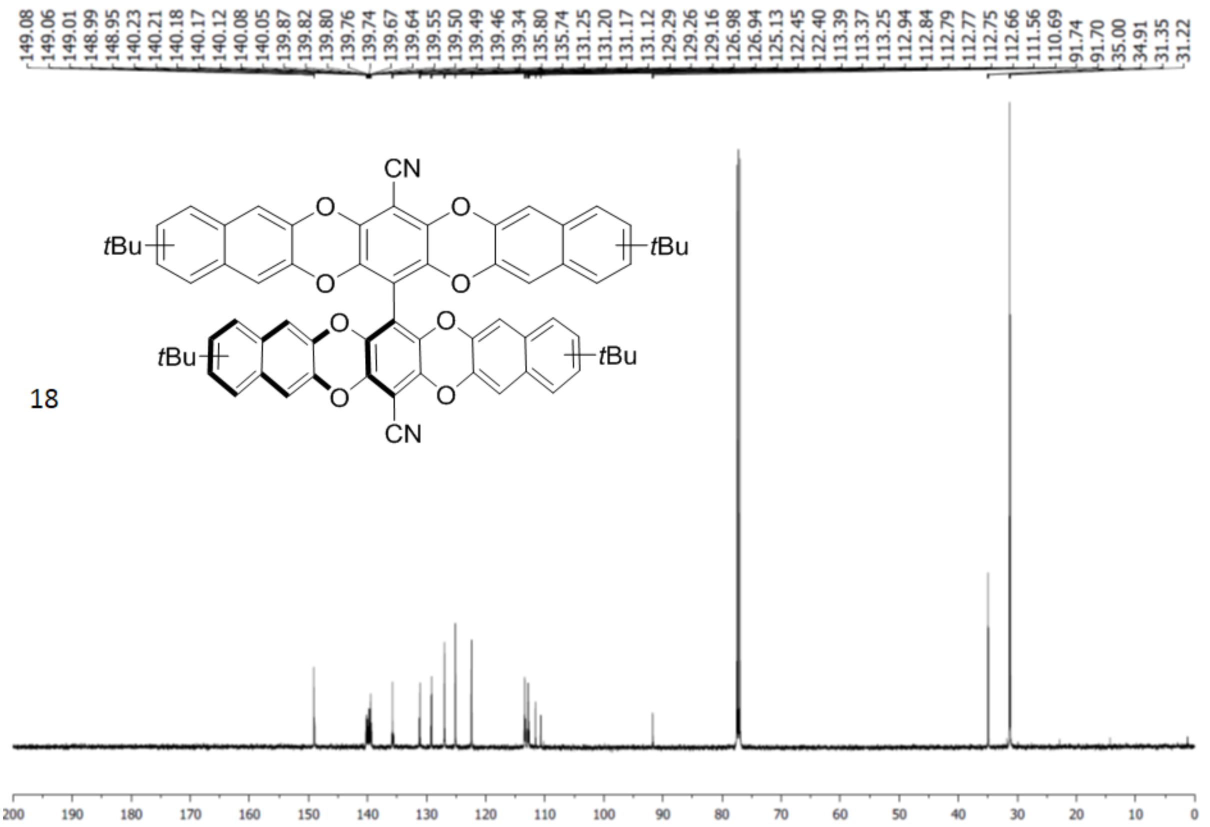

18

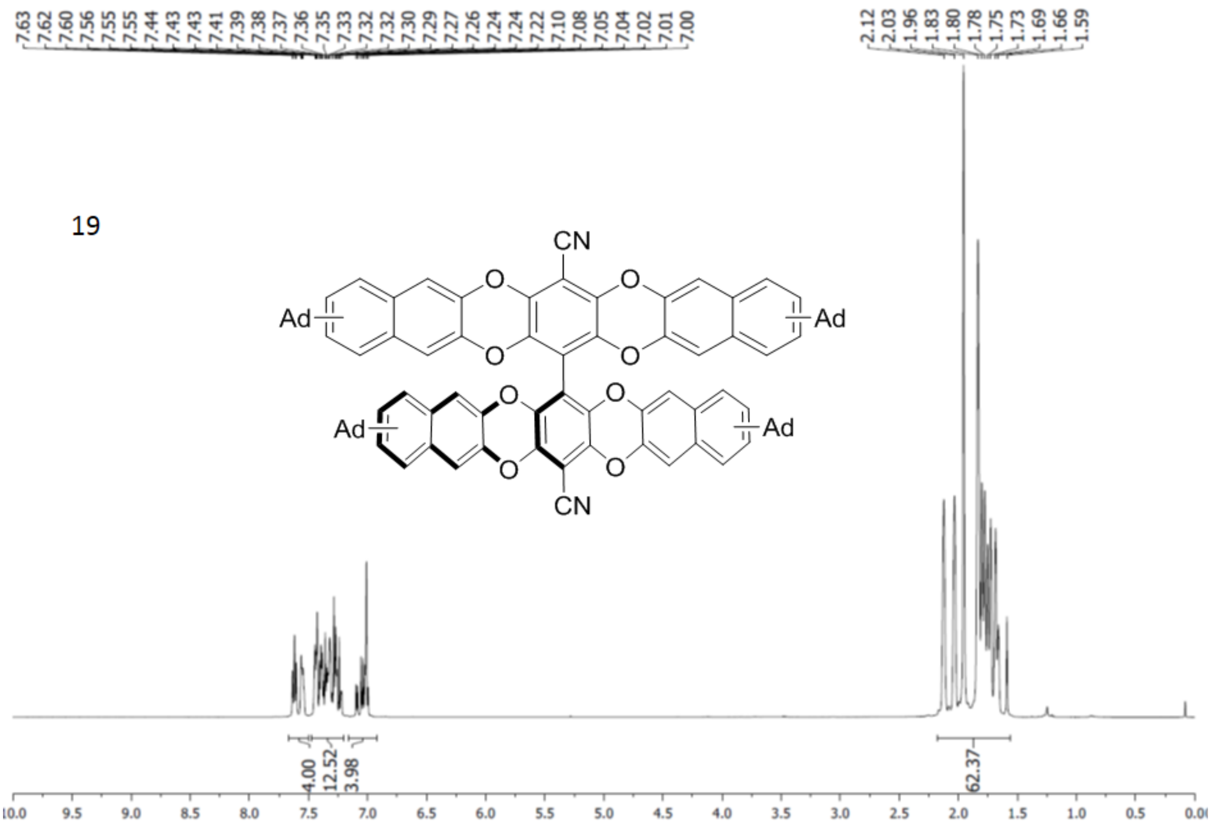
19
CN
Ad
O
CN

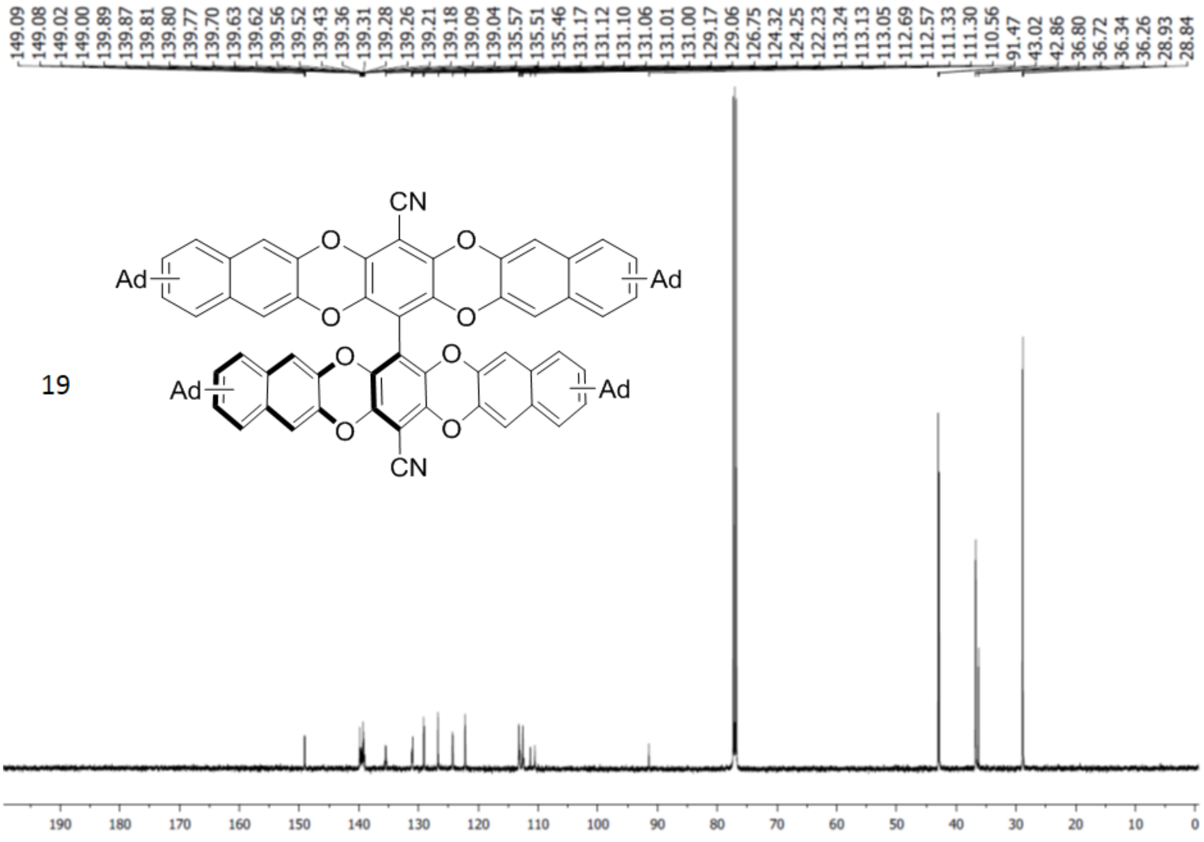
19
CN
Ad
O
CN

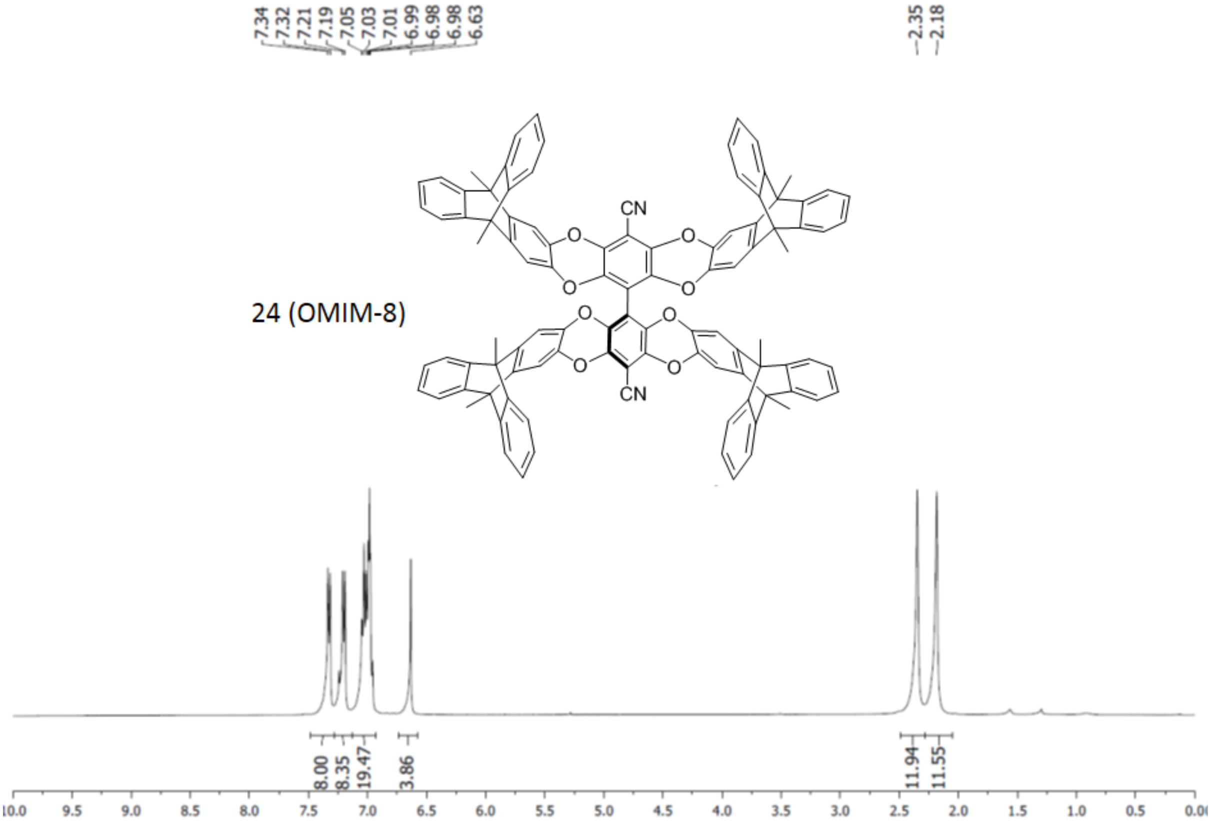
7.34
7.32
7.21
7.19
7.05
7.03
7.01
6.99
6.98
6.98
6.63
2.35
2.18
CN
24 (OMIM-8)
8.00
8.35
19.47
3.86
11.94
11.55

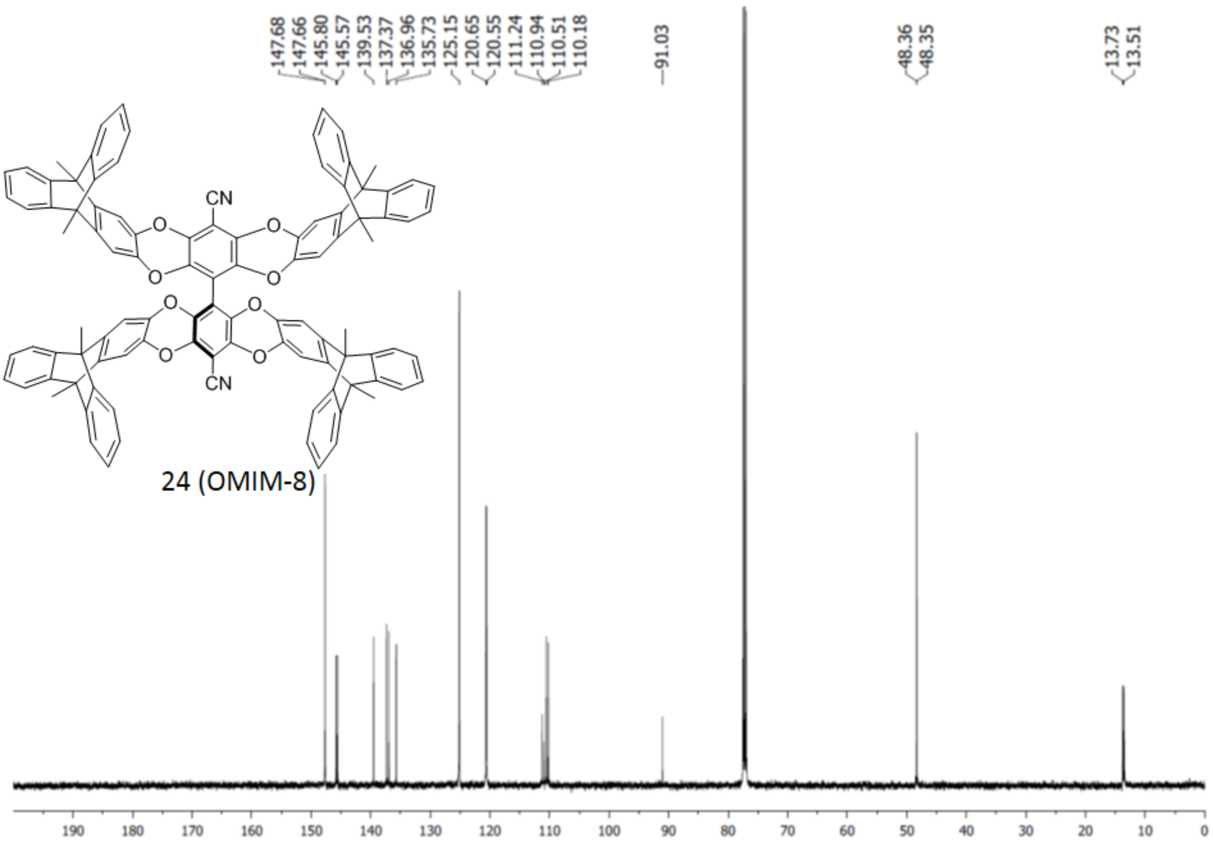
147.68
147.66
145.80
145.57
139.53
137.37
136.96
135.73
125.15
120.65
120.55
111.24
110.94
110.51
110.18
91.03
48.36
48.35
13.73
13.51
CN
24 (OMIM-8)

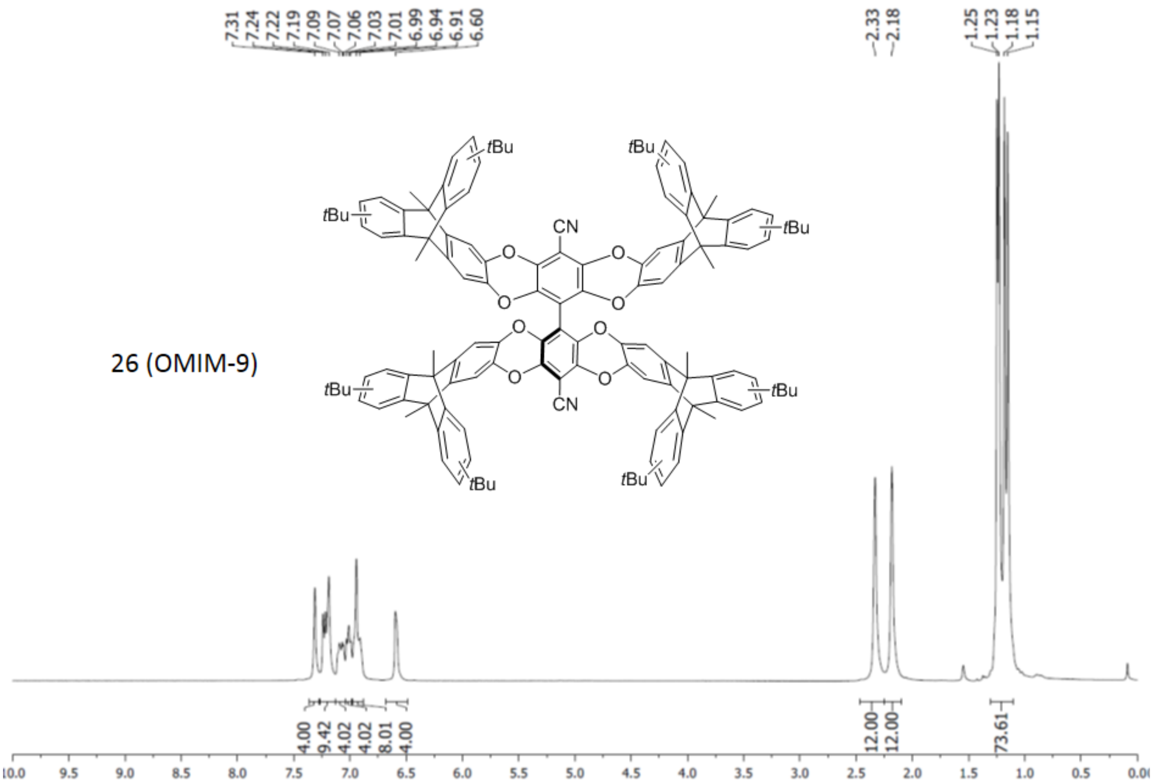
7.31
7.24
7.22
7.19
7.09
7.07
7.06
7.03
7.01
6.99
6.94
6.91
6.60
2.33
2.18
1.25
1.23
1.18
1.15
26 (OMIM-9)
CN
CN
tBu
4.00
9.42
4.02
4.02
8.01
4.00
12.00
12.00
73.61

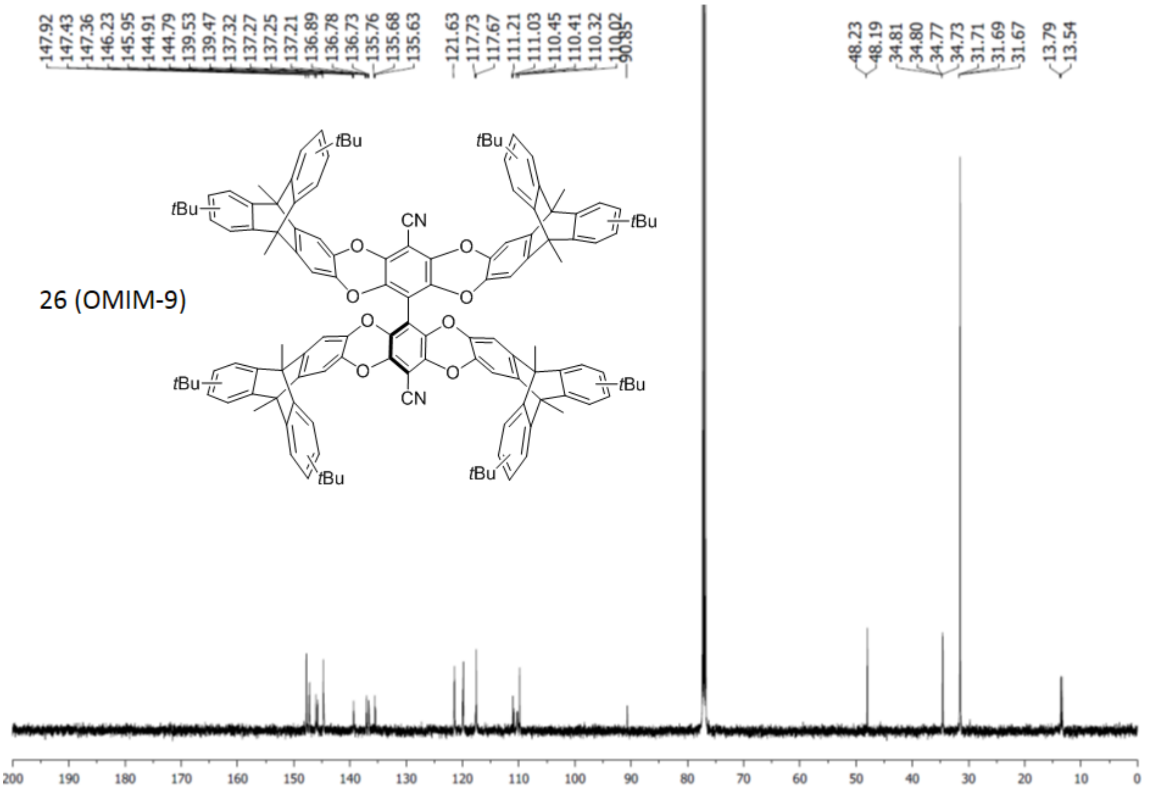
147.92
147.43
147.36
146.23
145.95
144.91
144.79
139.53
139.47
137.32
137.27
137.25
137.21
136.89
136.78
136.73
135.76
135.68
135.63
121.63
117.73
117.67
111.21
111.03
110.45
110.41
110.32
110.02
90.85
48.23
48.19
34.81
34.80
34.77
34.73
31.71
31.69
31.67
13.79
13.54
26 (OMIM-9)
CN
CN
tBu

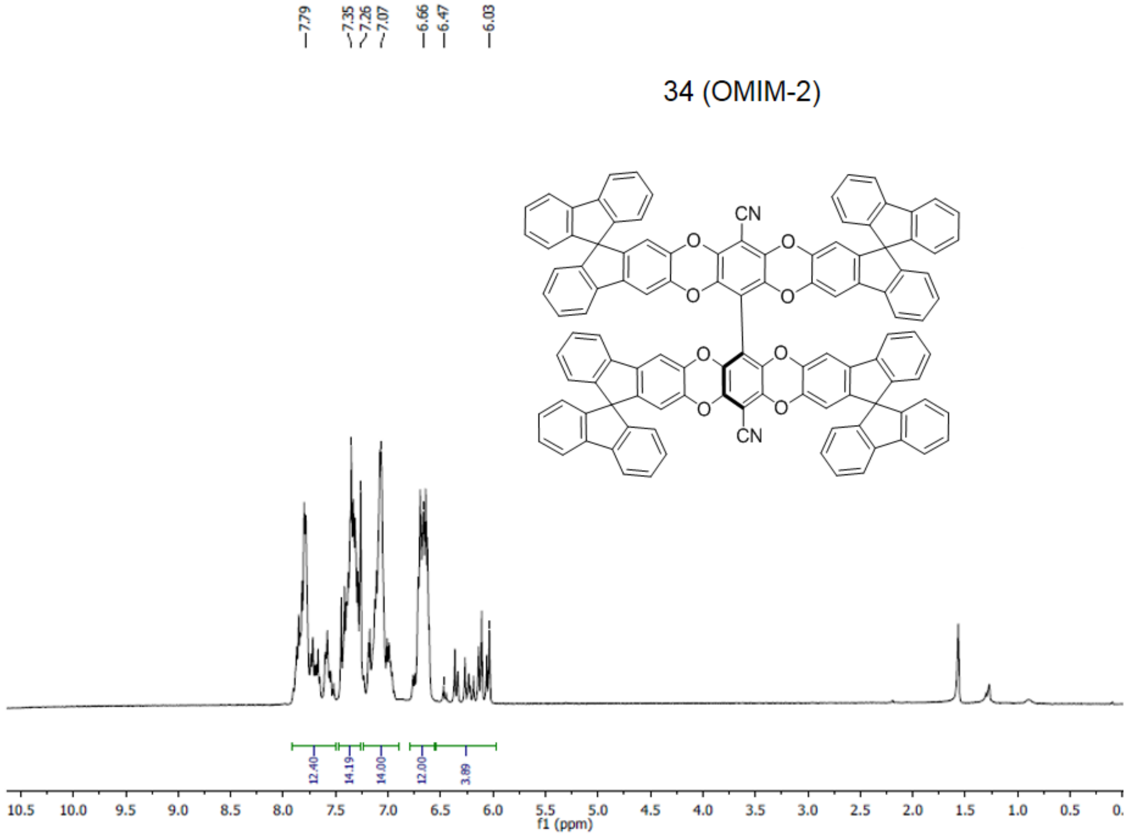
7.79
7.35
7.26
7.07
6.66
6.47
6.03
34 (OMIM-2)
CN
O
CN
12.40
14.19
14.00
12.00
3.89
f1 (ppm)

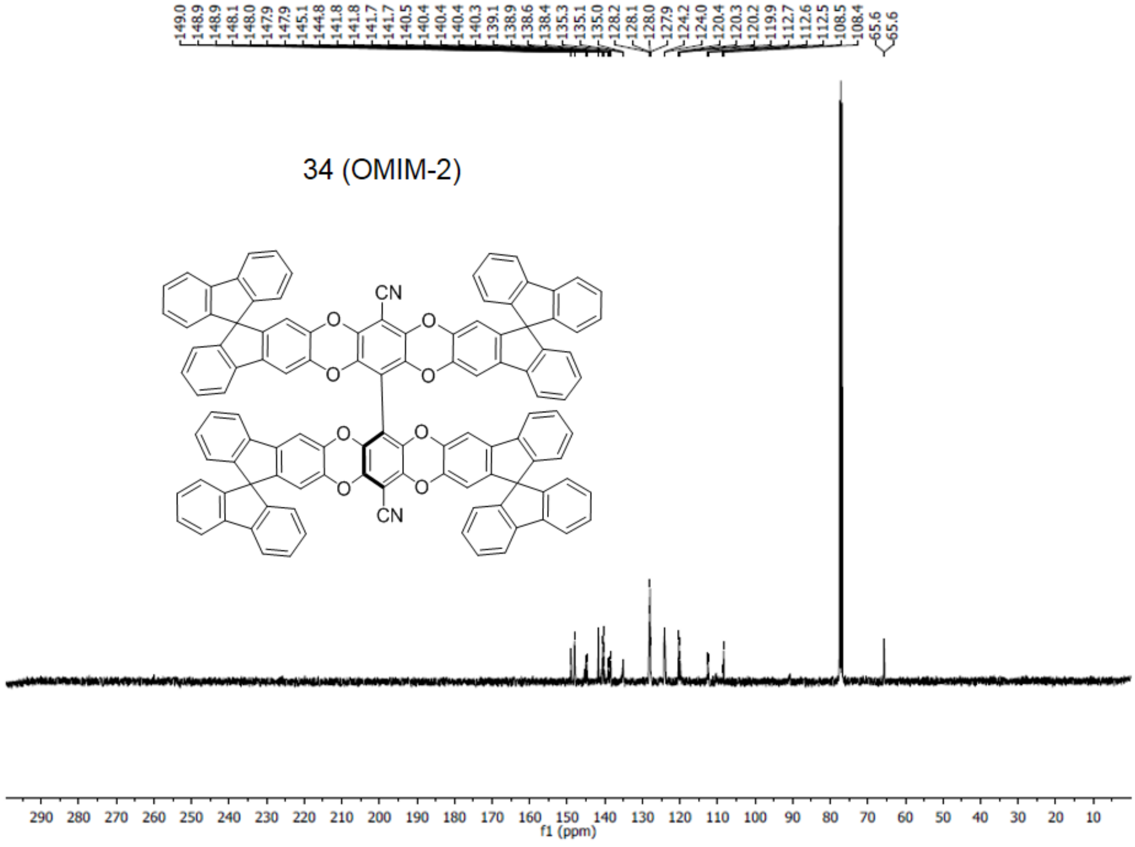
149.0
148.9
148.9
148.1
148.0
147.9
147.9
145.1
144.8
141.8
141.7
141.7
140.5
140.4
140.4
140.4
140.3
139.1
138.9
138.6
138.4
135.3
135.1
135.0
128.2
128.1
128.0
127.9
124.2
124.0
120.4
120.3
120.2
119.9
112.7
112.6
112.5
108.5
108.4
65.6
65.6
34 (OMIM-2)
CN
O
CN
f1 (ppm)

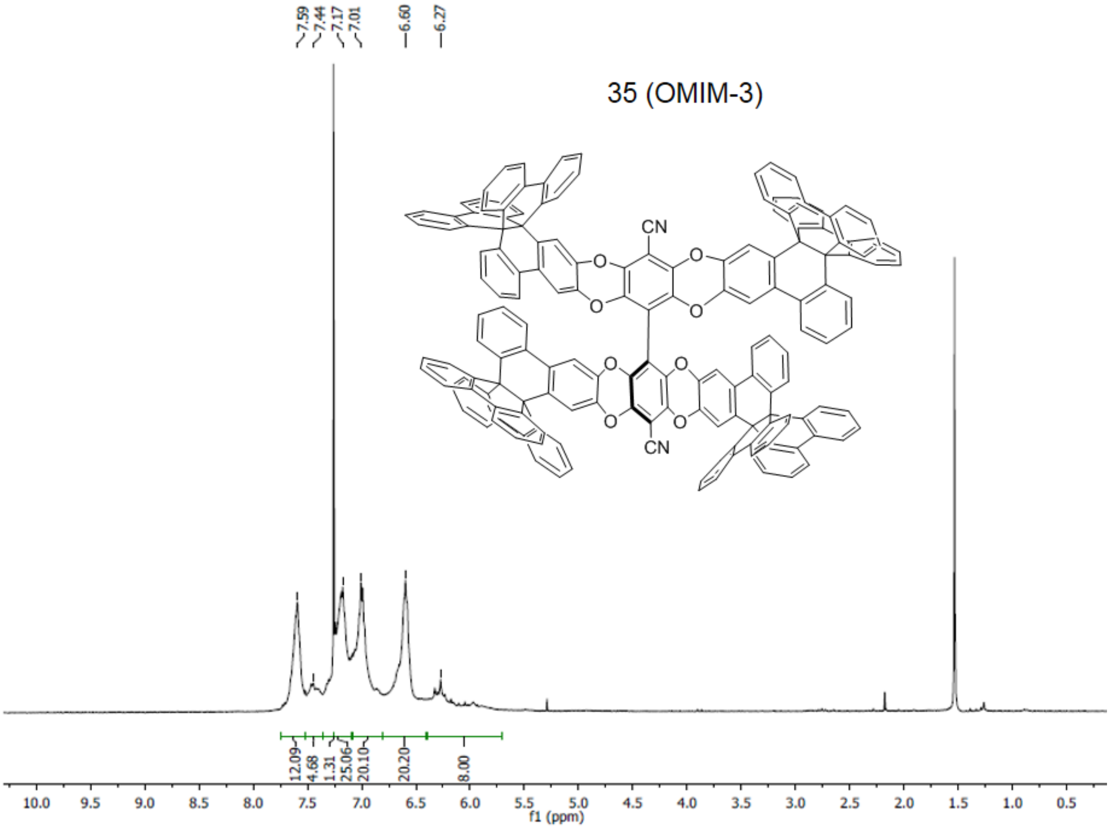
7.59
7.44
7.17
7.01
6.60
6.27
35 (OMIM-3)
CN
O
CN
12.09
4.68
1.31
25.06
20.10
20.20
8.00
f1 (ppm)

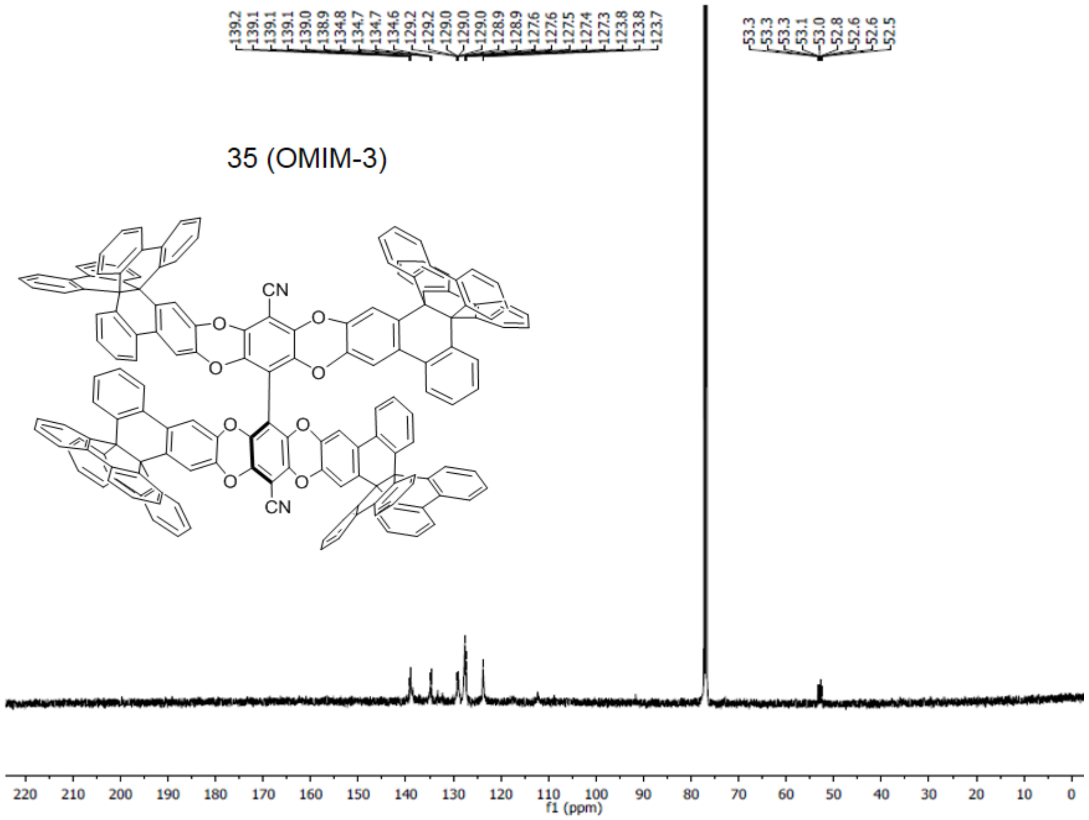
139.2
139.1
139.1
139.1
139.0
138.9
134.8
134.7
134.7
134.6
129.2
129.2
129.0
129.0
129.0
128.9
128.9
127.6
127.6
127.5
127.4
127.3
123.8
123.8
123.7
53.3
53.3
53.3
53.1
53.0
52.8
52.6
52.6
52.5
35 (OMIM-3)
CN
O
CN
f1 (ppm)

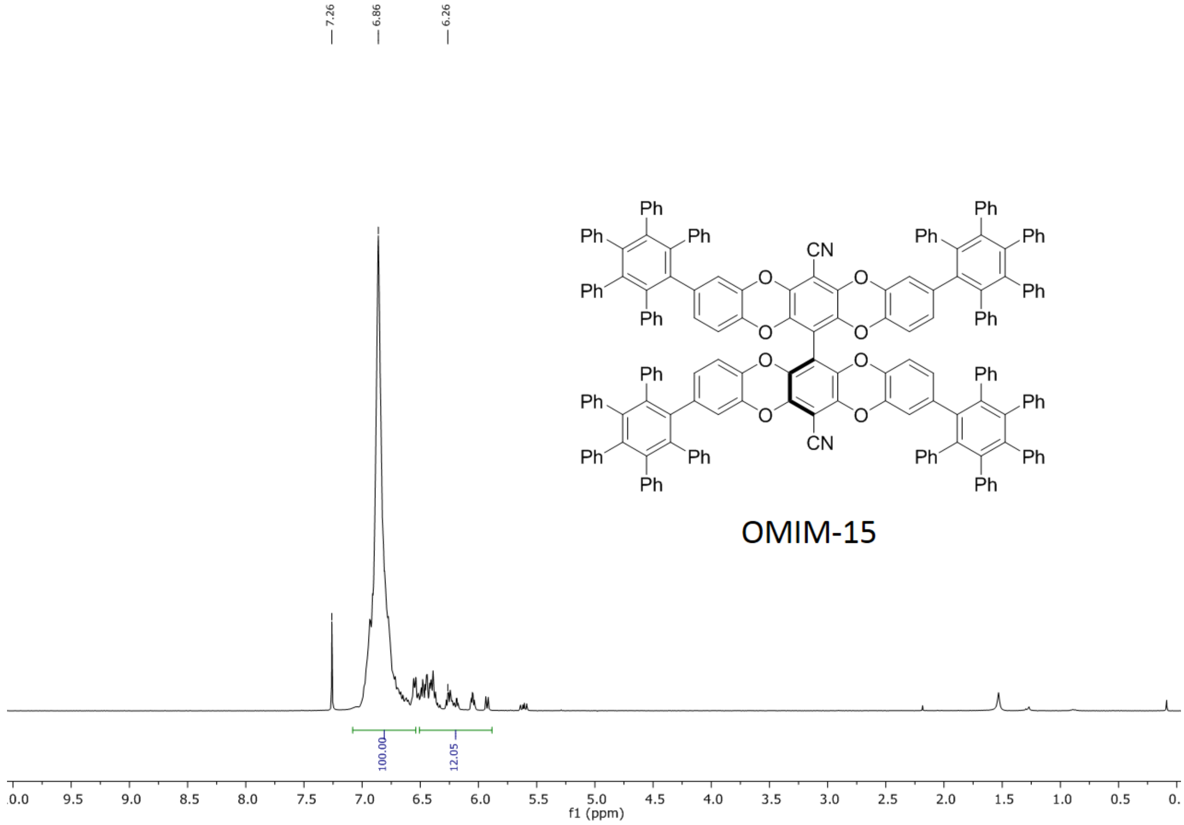
7.26
6.86
6.26
Ph
CN
O
OMIM-15
100.00
12.05
0.0 9.5 9.0 8.5 8.0 7.5 7.0 6.5 6.0 5.5 5.0 4.5 4.0 3.5 3.0 2.5 2.0 1.5 1.0 0.5 0.
f1 (ppm)

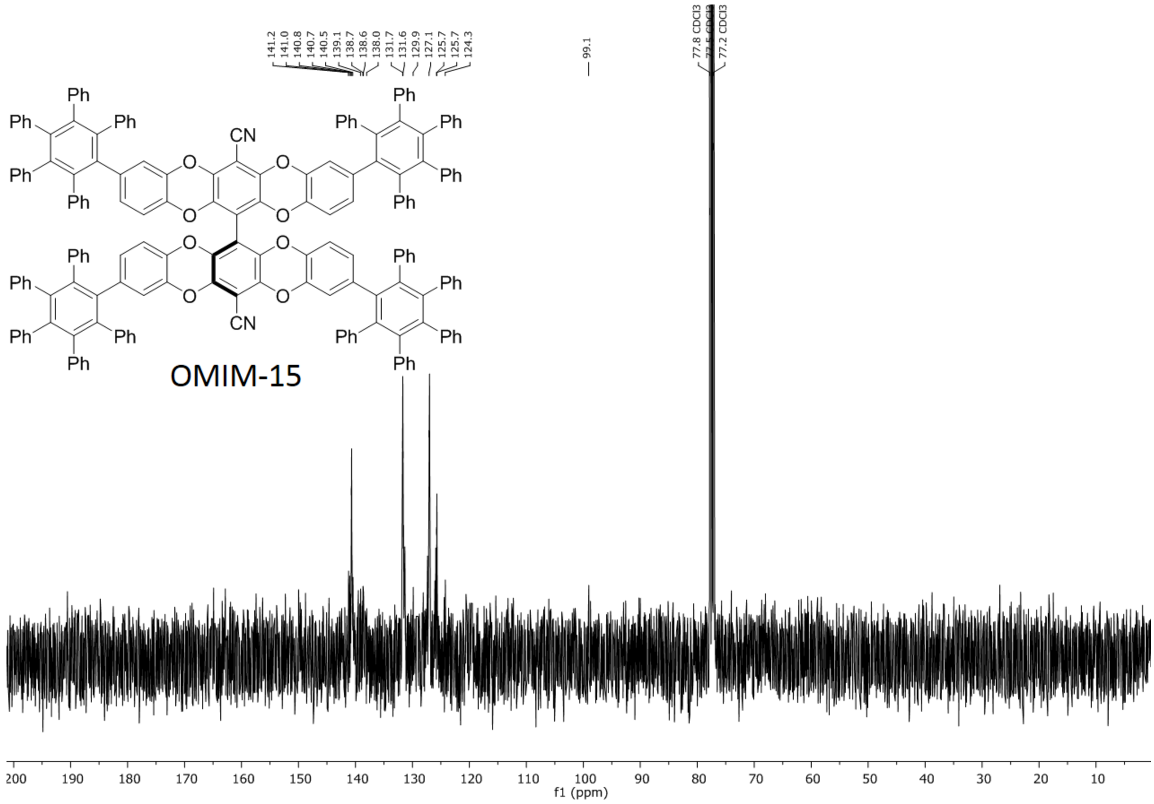
141.2
141.0
140.8
140.7
140.5
139.1
138.7
138.6
138.0
131.7
131.6
129.9
127.1
125.7
125.7
124.3
99.1
77.8 CDCl3
77.2 CDCl3
Ph
CN
O
OMIM-15
200 190 180 170 160 150 140 130 120 110 100 90 80 70 60 50 40 30 20 10
f1 (ppm)

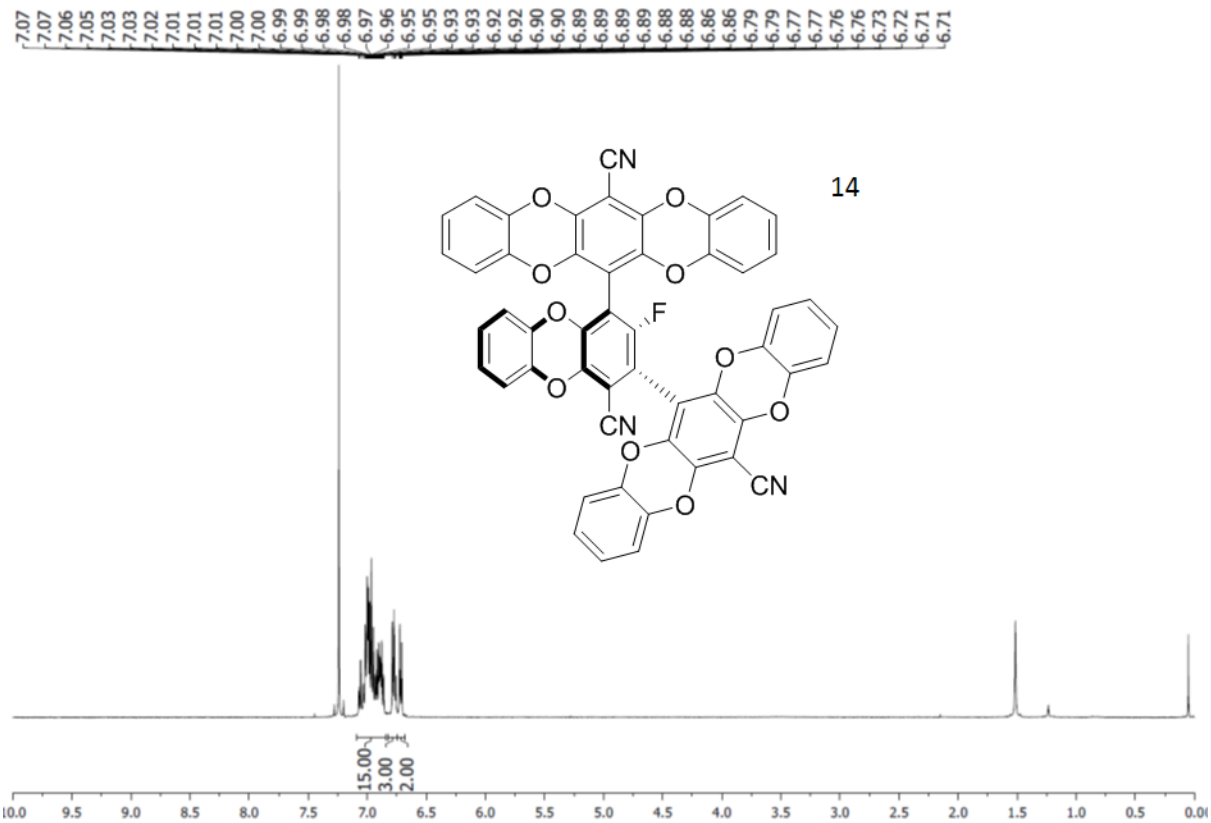
7.07 7.07 7.06 7.05 7.03 7.03 7.02 7.01 7.01 7.00 7.00 6.99 6.99 6.98 6.98 6.97 6.96 6.95 6.95 6.93 6.93 6.92 6.92 6.90 6.90 6.89 6.89 6.89 6.89 6.88 6.88 6.86 6.86 6.79 6.79 6.77 6.77 6.76 6.76 6.73 6.72 6.71 6.71
CN
O
F
14
15.00
3.00
2.00
10.0 9.5 9.0 8.5 8.0 7.5 7.0 6.5 6.0 5.5 5.0 4.5 4.0 3.5 3.0 2.5 2.0 1.5 1.0 0.5 0.0

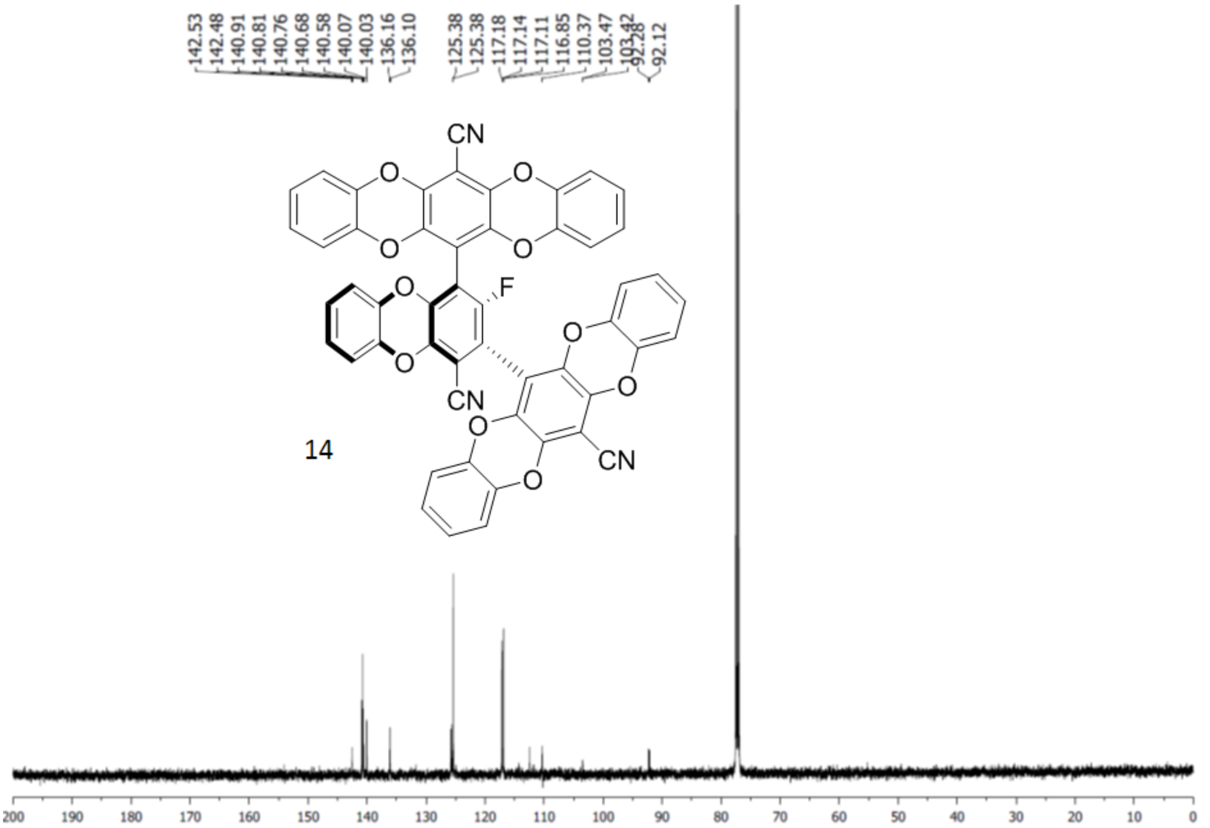
142.53 142.48 140.91 140.81 140.76 140.68 140.58 140.07 140.03 136.16 136.10 125.38 125.38 117.18 117.14 117.11 116.85 110.37 103.47 103.42 92.28 92.12
CN
O
F
14
200 190 180 170 160 150 140 130 120 110 100 90 80 70 60 50 40 30 20 10 0

15

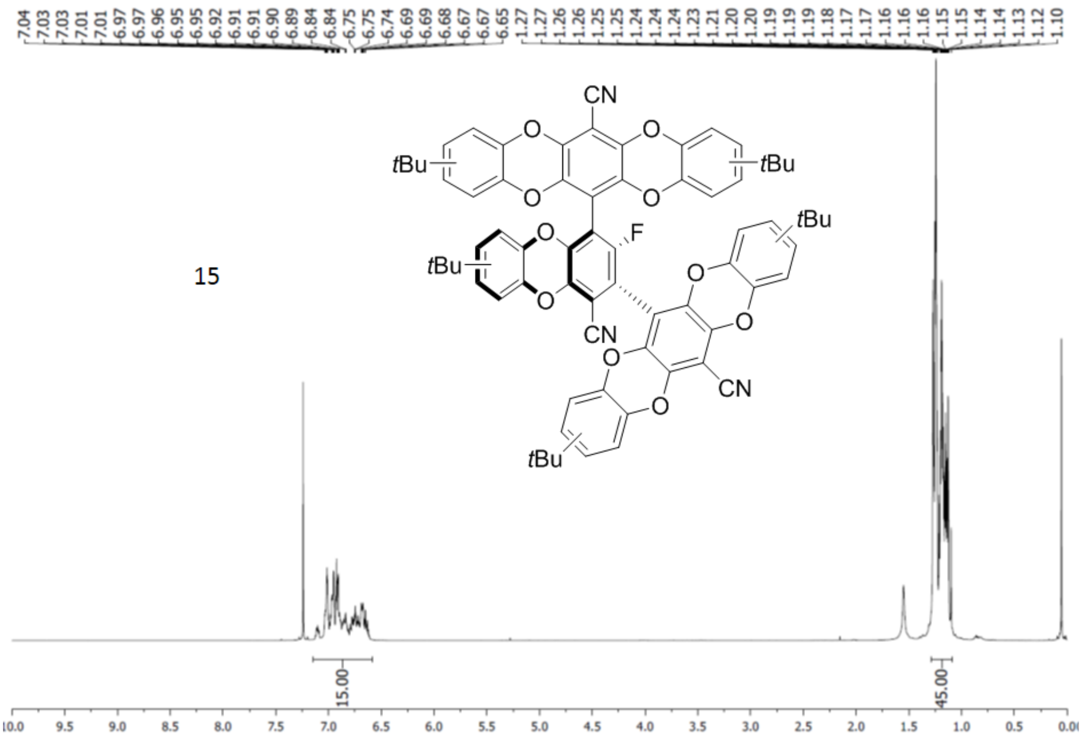

7.04 7.03 7.03 7.01 7.01 6.97 6.97 6.96 6.95 6.95 6.92 6.91 6.91 6.90 6.89 6.84 6.84 6.75 6.75 6.74 6.69 6.69 6.68 6.67 6.67 6.65 1.27 1.26 1.26 1.25 1.25 1.24 1.24 1.24 1.23 1.21 1.20 1.20 1.19 1.19 1.19 1.18 1.17 1.17 1.16 1.16 1.16 1.15 1.15 1.14 1.13 1.13 1.12 1.10
CN
tBu
F
O
15.00
45.00

15

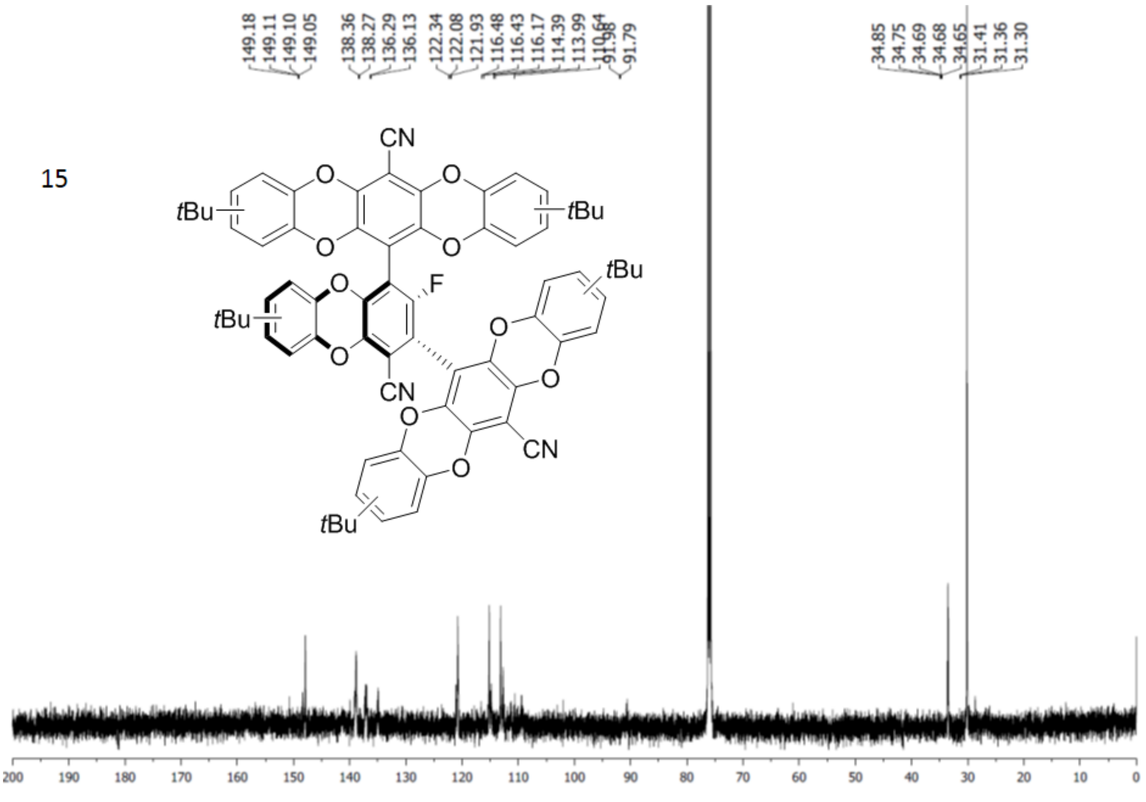

149.18 149.11 149.10 149.05 138.36 138.27 136.29 136.13 122.34 122.08 121.93 116.48 116.43 116.17 114.39 113.99 110.64 91.98 91.79
34.85 34.75 34.69 34.68 34.65 31.41 31.36 31.30
CN
tBu
F
O

15 ^{19}F

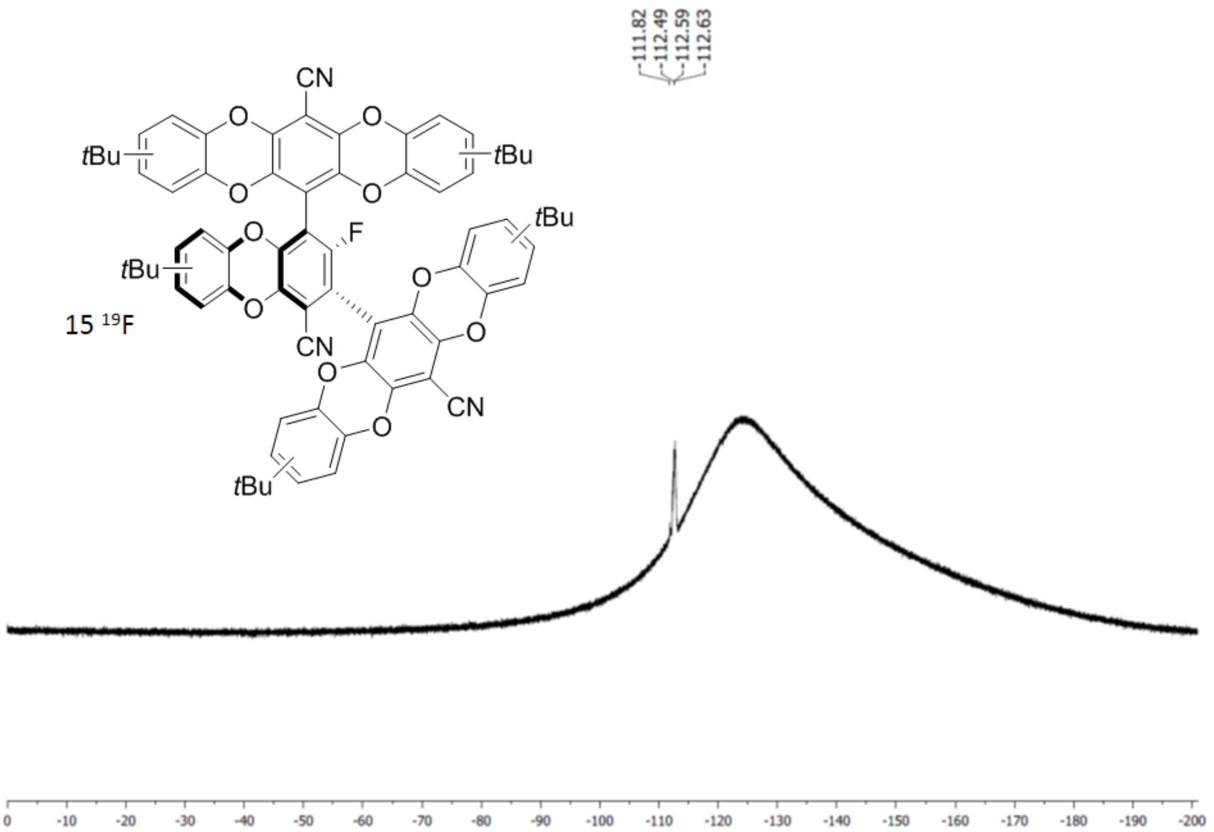

-111.82
-112.49
-112.59
-112.63
CN
tBu
F
0 -10 -20 -30 -40 -50 -60 -70 -80 -90 -100 -110 -120 -130 -140 -150 -160 -170 -180 -190 -200

16

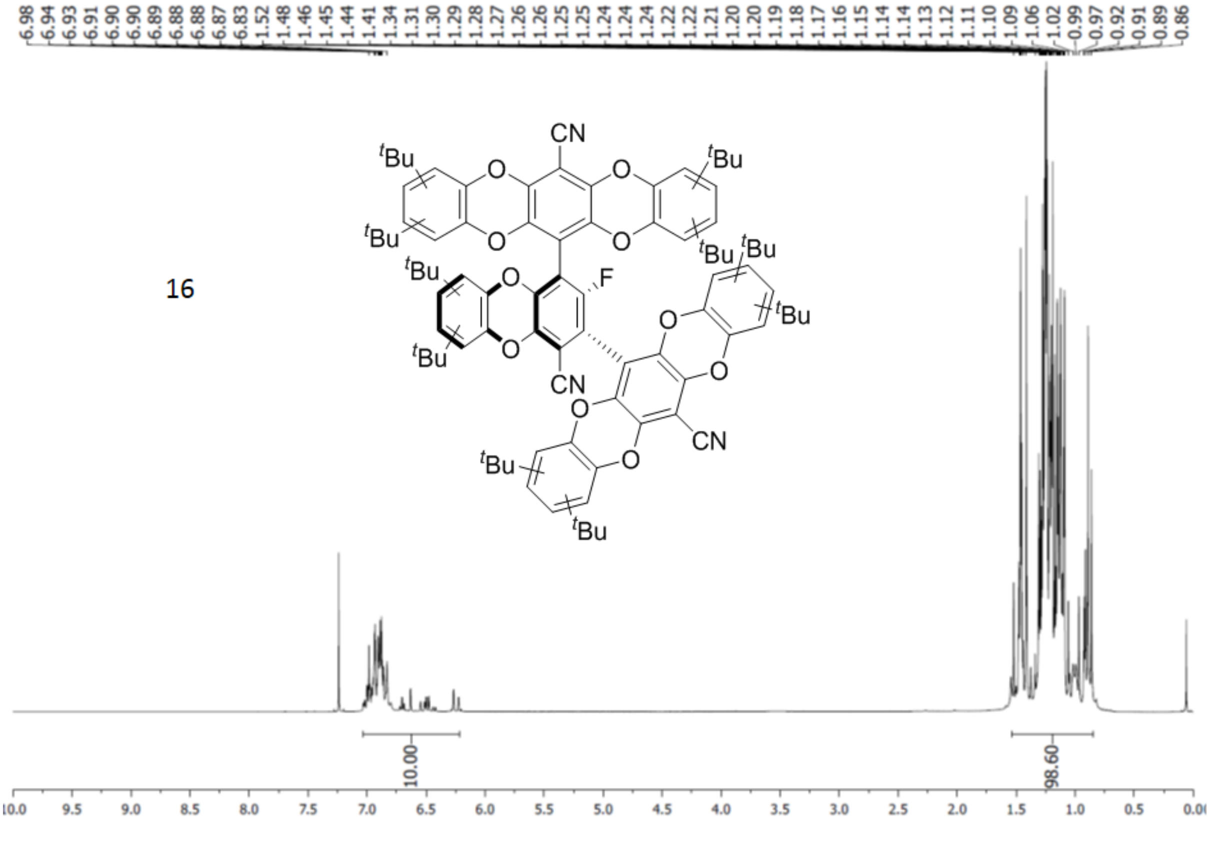

6.98 6.94 6.93 6.91 6.90 6.90 6.89 6.88 6.88 6.87 6.83 1.52 1.48 1.46 1.45 1.44 1.41 1.34 1.31 1.30 1.29 1.28 1.27 1.26 1.26 1.25 1.25 1.24 1.24 1.24 1.22 1.22 1.21 1.20 1.20 1.19 1.18 1.17 1.16 1.15 1.14 1.14 1.13 1.12 1.11 1.10 1.09 1.06 1.02 0.99 0.97 0.92 0.91 0.89 0.86
CN
^{t}Bu
F
10.00
98.60
10.0 9.5 9.0 8.5 8.0 7.5 7.0 6.5 6.0 5.5 5.0 4.5 4.0 3.5 3.0 2.5 2.0 1.5 1.0 0.5 0.0

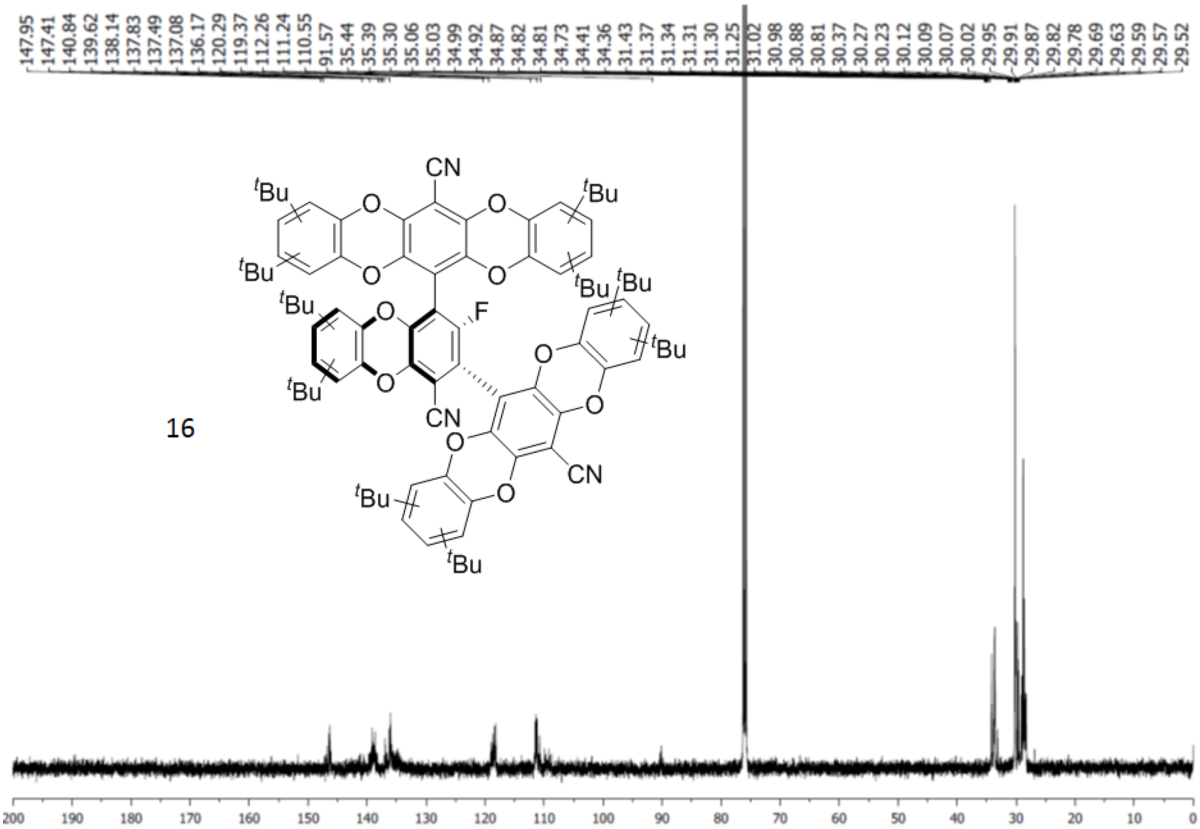
147.95
147.41
140.84
139.62
138.14
137.83
137.49
137.08
136.17
120.29
119.37
112.26
111.24
110.55
91.57
35.44
35.39
35.30
35.06
35.03
34.99
34.92
34.87
34.82
34.81
34.73
34.41
34.36
31.43
31.37
31.34
31.31
31.30
31.25
31.02
30.98
30.88
30.81
30.37
30.27
30.23
30.12
30.09
30.07
30.02
29.95
29.91
29.87
29.82
29.78
29.69
29.63
29.59
29.57
29.52
CN
tBu
O
F
CN
200 190 180 170 160 150 140 130 120 110 100 90 80 70 60 50 40 30 20 10 0

16

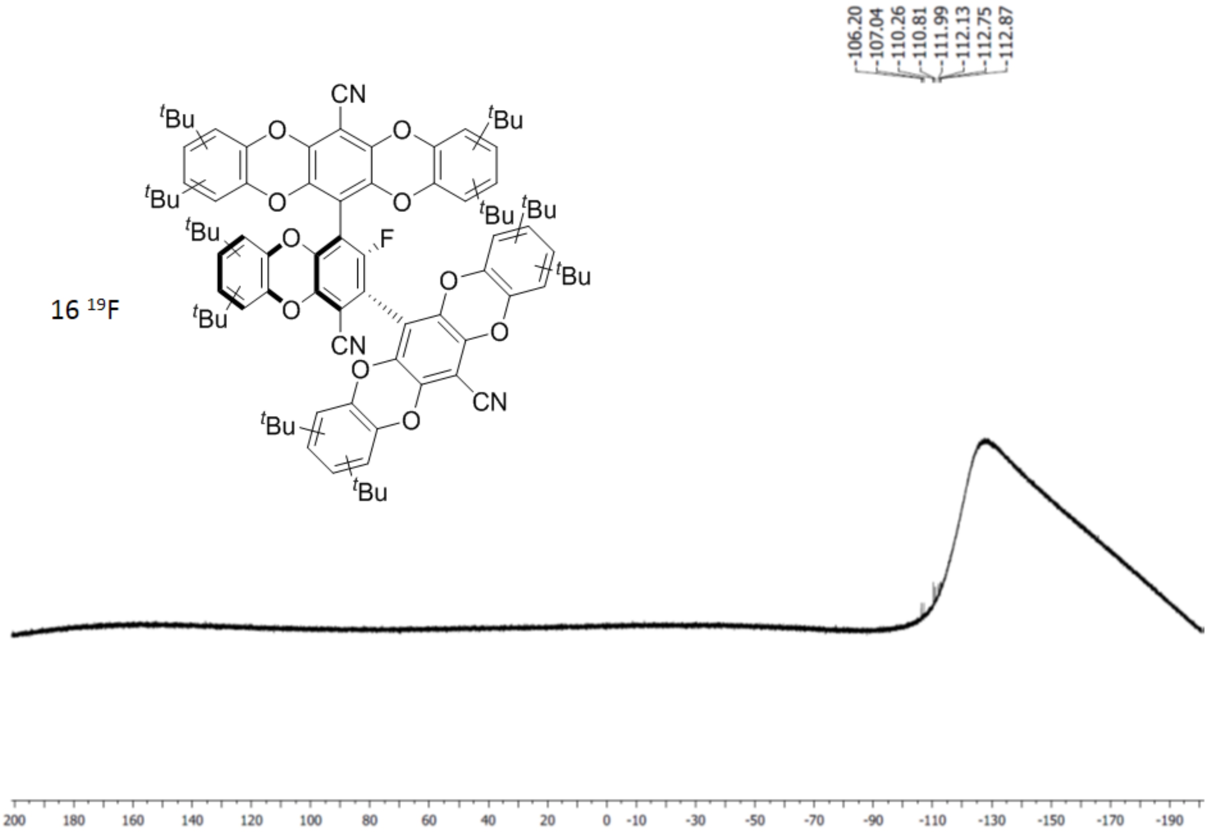
106.20
107.04
110.26
110.81
111.99
112.13
112.75
112.87
CN
tBu
O
F
200 180 160 140 120 100 80 60 40 20 0 -10 -30 -50 -70 -90 -110 -130 -150 -170 -190

16 ^{19}F

20

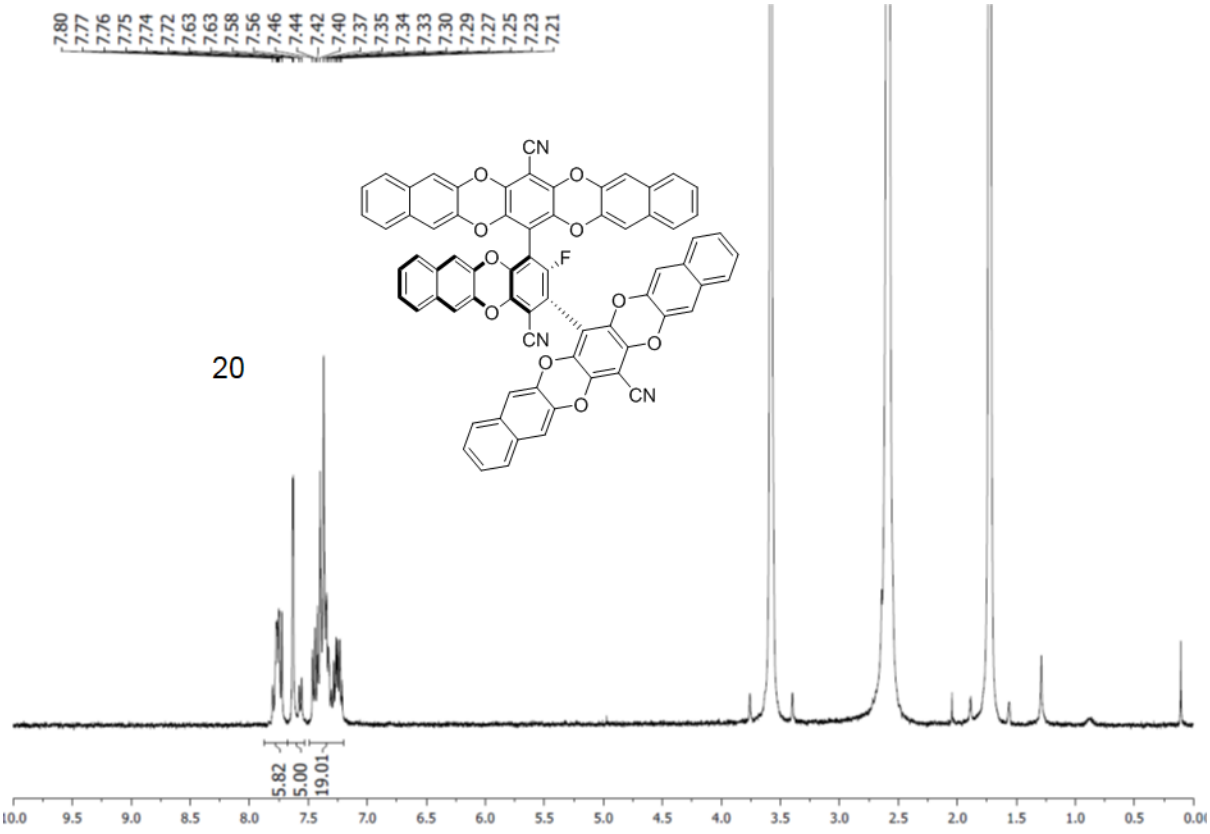
7.80
7.77
7.76
7.75
7.74
7.72
7.63
7.63
7.58
7.56
7.46
7.44
7.42
7.40
7.37
7.35
7.34
7.33
7.30
7.29
7.27
7.25
7.23
7.21
CN
F
CN
CN
5.82
5.00
19.01

20 ^{19}F

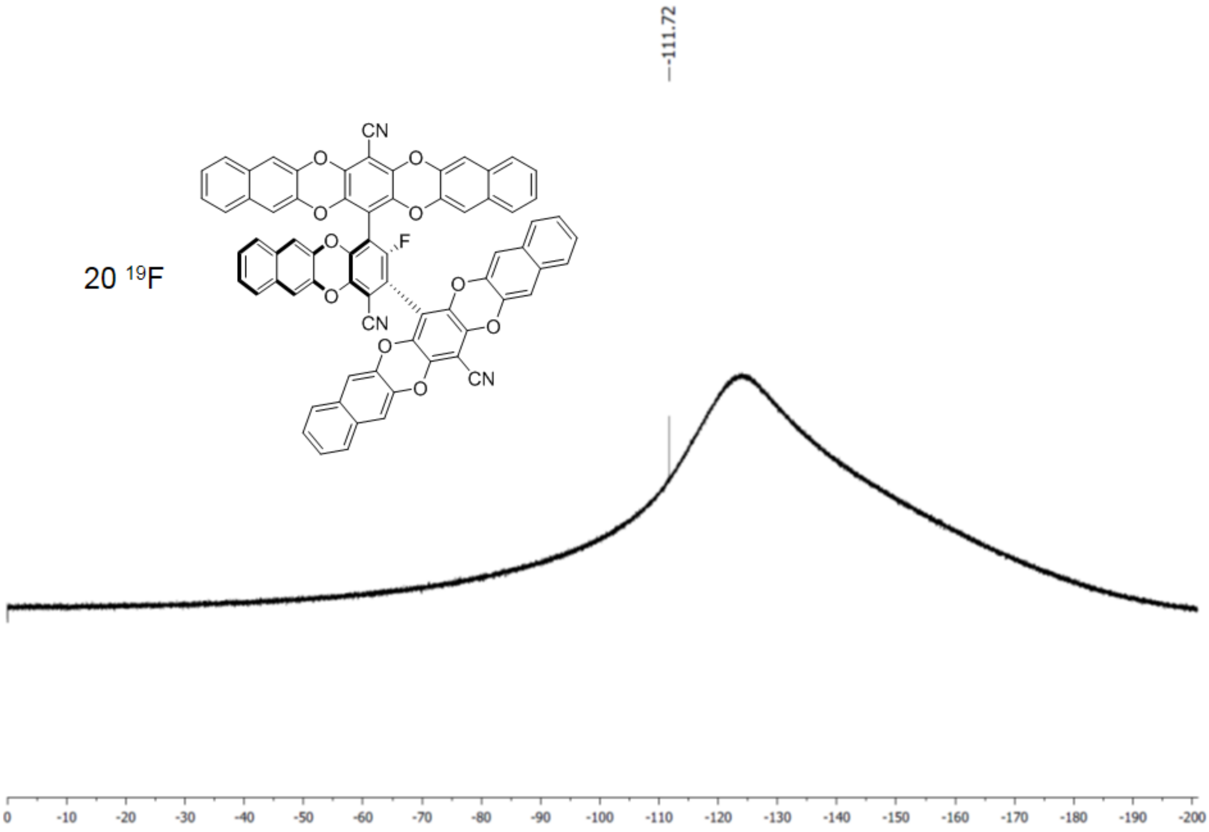
-111.72
CN
F
CN
CN

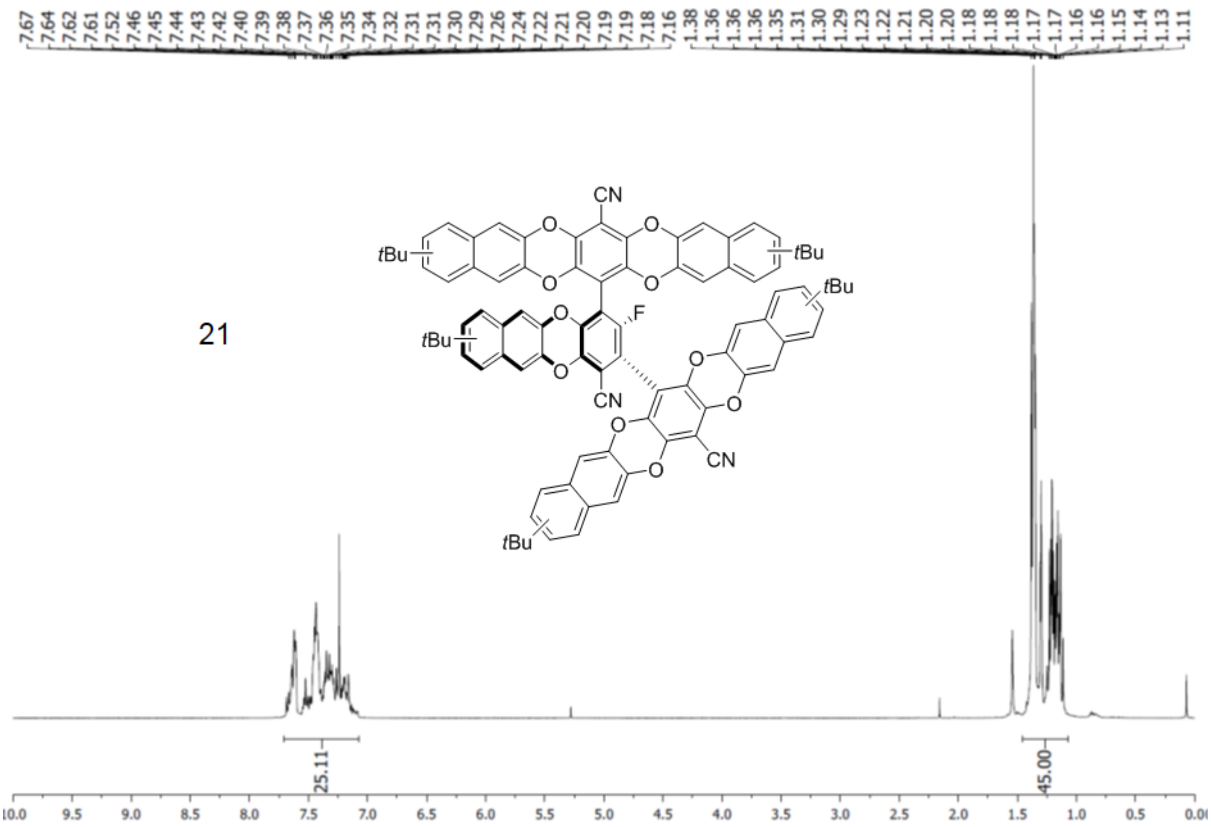
7.67 7.64 7.62 7.61 7.52 7.46 7.45 7.44 7.43 7.42 7.40 7.39 7.38 7.37 7.36 7.35 7.34 7.32 7.31 7.30 7.29 7.26 7.24 7.22 7.21 7.20 7.19 7.19 7.18 7.16 1.38 1.36 1.36 1.35 1.31 1.30 1.29 1.23 1.22 1.21 1.20 1.18 1.18 1.18 1.17 1.17 1.16 1.16 1.15 1.14 1.13 1.11
21
CN
tBu
F
CN
CN
25.11
45.00
10.0 9.5 9.0 8.5 8.0 7.5 7.0 6.5 6.0 5.5 5.0 4.5 4.0 3.5 3.0 2.5 2.0 1.5 1.0 0.5

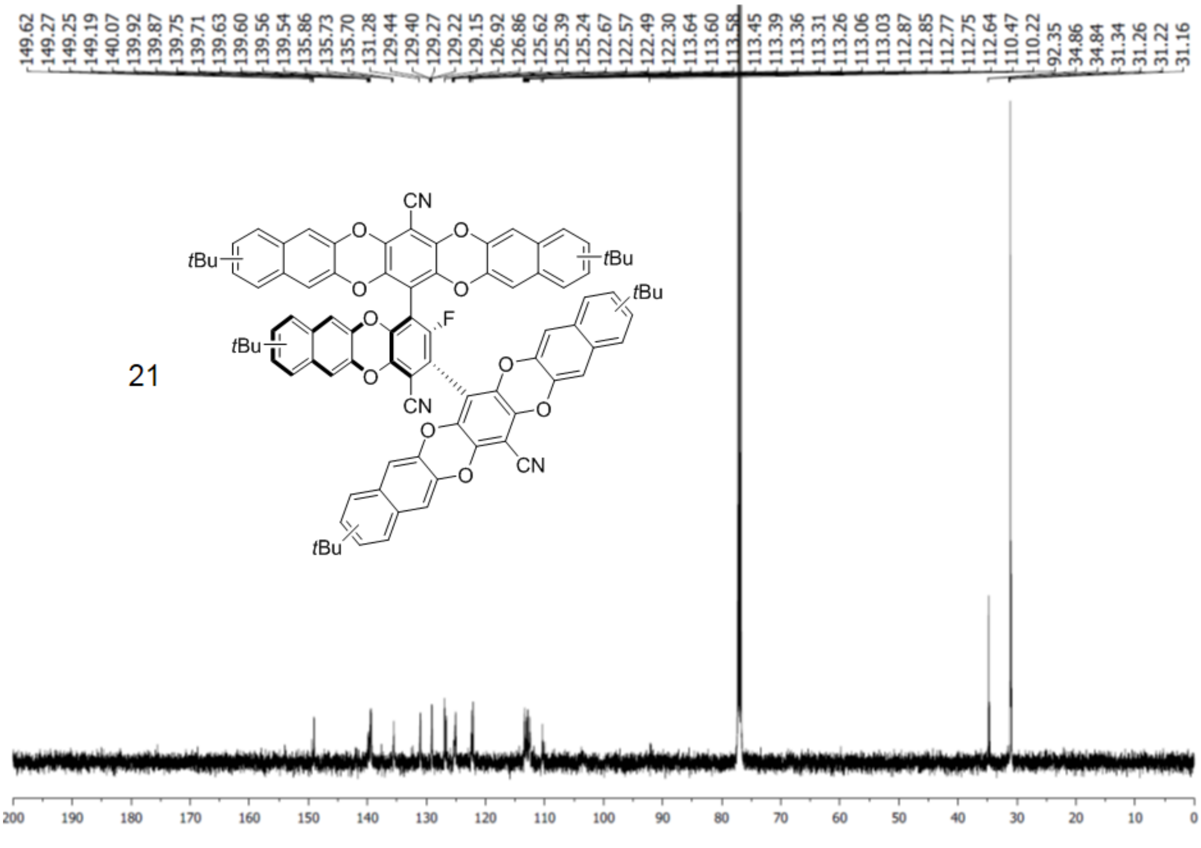
149.62 149.27 149.25 149.19 140.07 139.92 139.87 139.75 139.71 139.63 139.60 139.56 139.54 135.86 135.73 135.70 131.28 129.44 129.40 129.27 129.22 129.15 126.92 126.86 125.62 125.39 125.24 122.67 122.57 122.49 122.30 113.64 113.60 113.58 113.45 113.39 113.36 113.31 113.26 113.06 113.03 112.87 112.85 112.77 112.75 112.64 110.47 110.22 92.35 34.86 34.84 31.34 31.26 31.22 31.16
21
CN
tBu
F
CN
CN
200 190 180 170 160 150 140 130 120 110 100 90 80 70 60 50 40 30 20 10 0

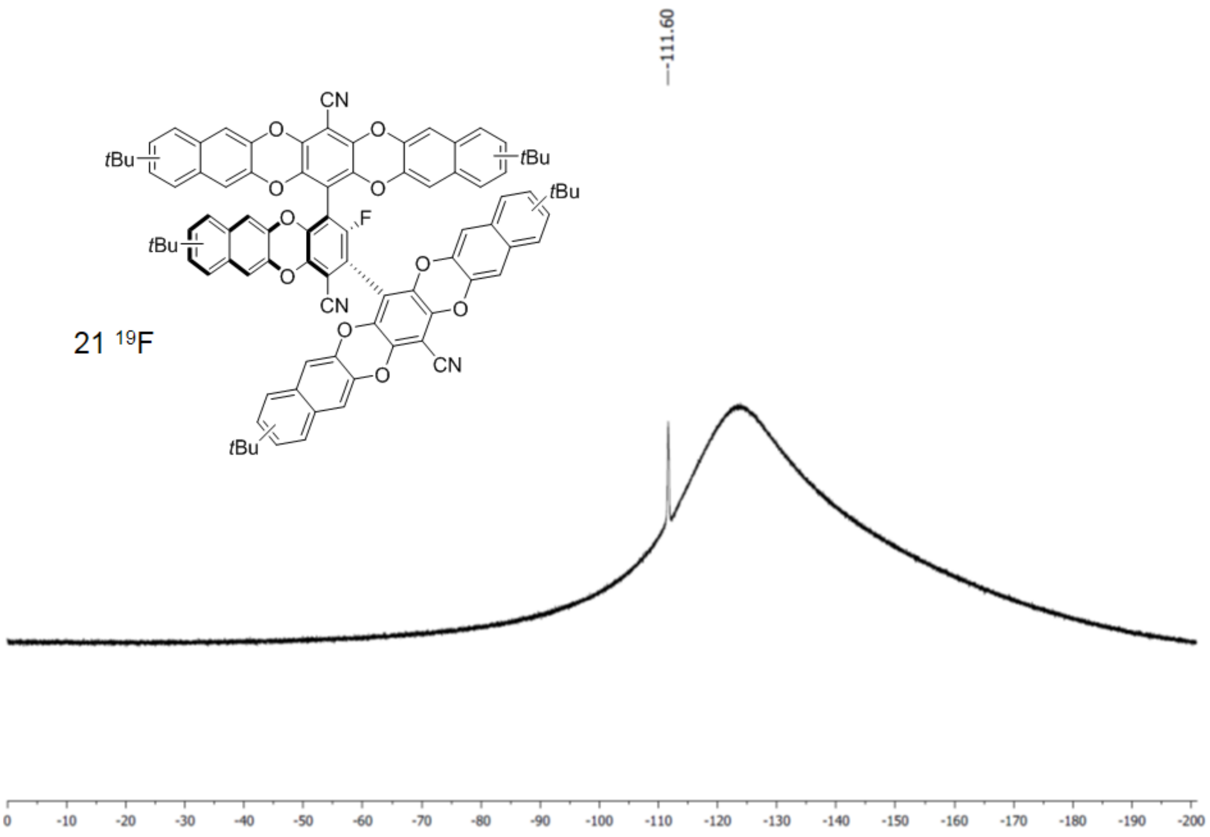

-111.60
CN
tBu
tBu
tBu
F
tBu
CN
CN
21 19F
tBu

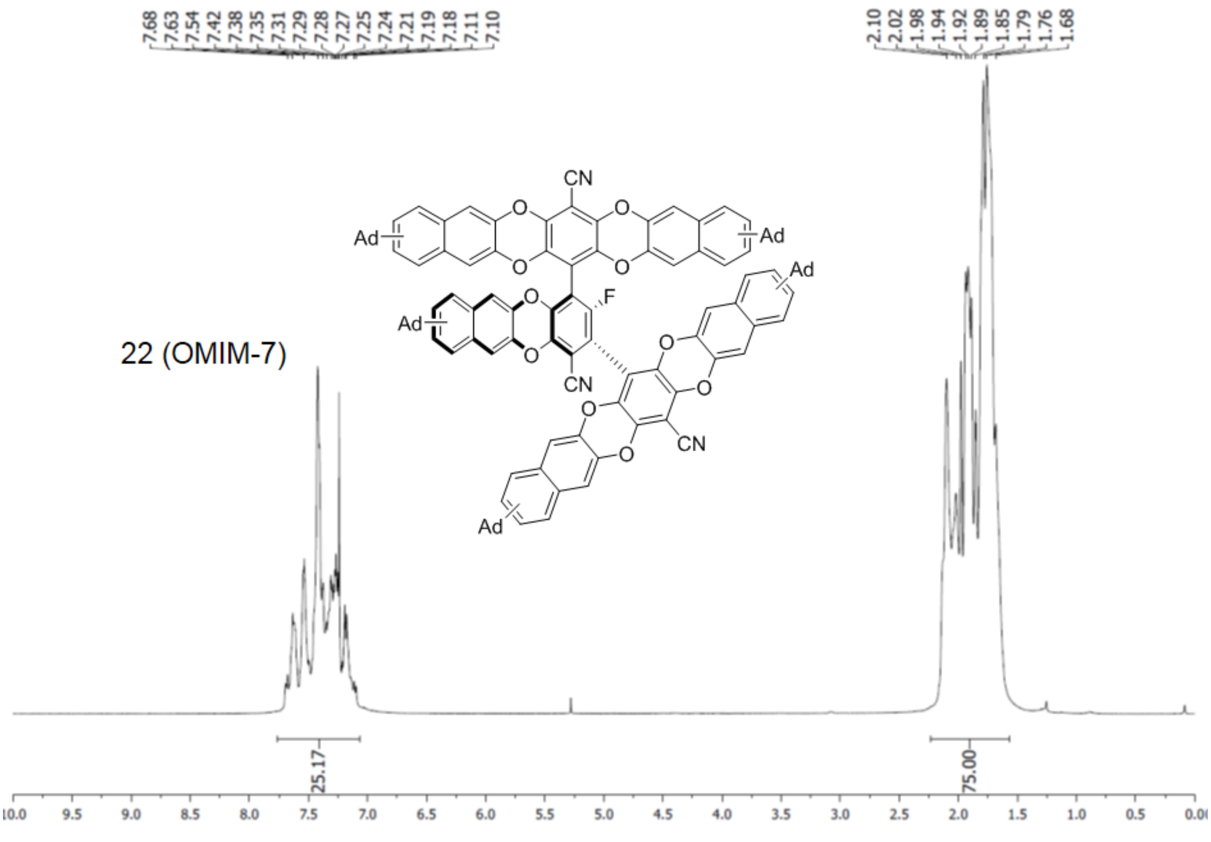

7.68 7.63 7.54 7.42 7.38 7.35 7.31 7.29 7.28 7.27 7.25 7.24 7.21 7.19 7.18 7.11 7.10
2.10 2.02 1.98 1.94 1.92 1.89 1.85 1.79 1.76 1.68
CN
Ad
Ad
Ad
F
Ad
22 (OMIM-7)
CN
CN
Ad
25.17
75.00

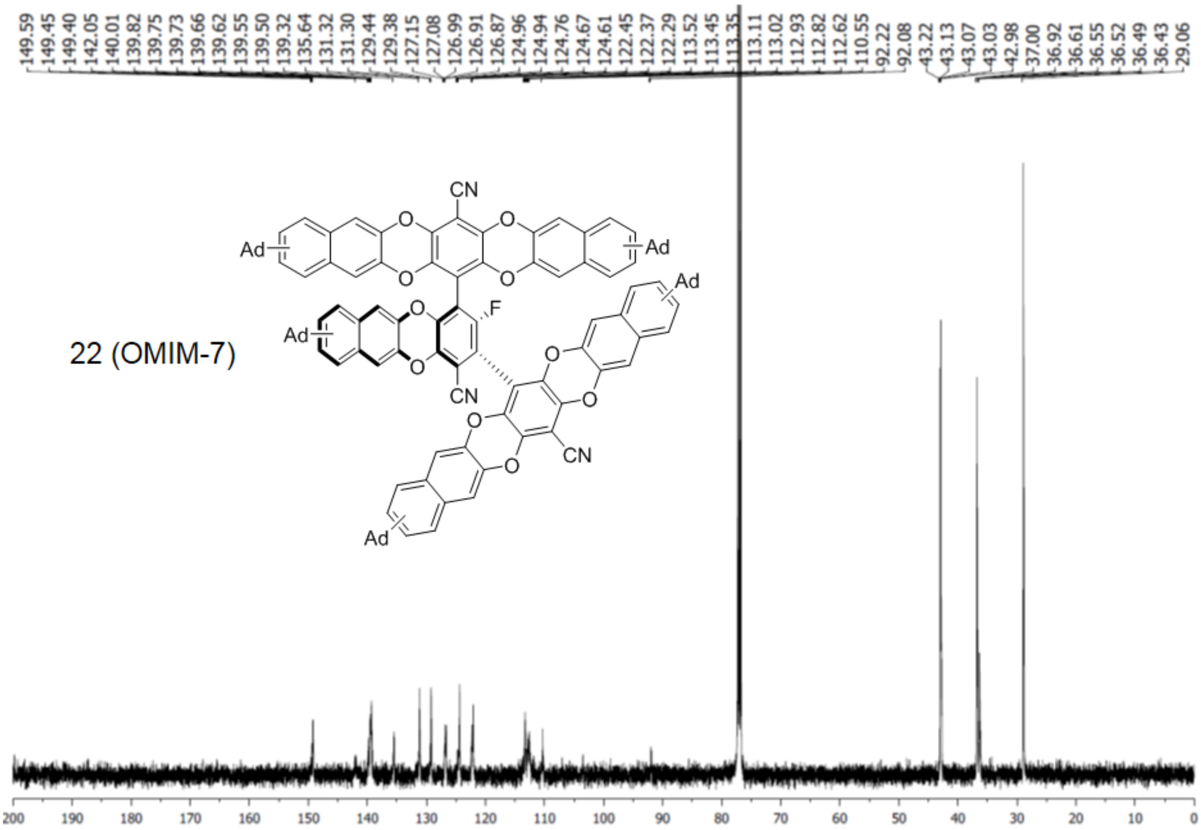
149.59
149.45
149.40
142.05
140.01
139.82
139.75
139.73
139.66
139.62
139.55
139.50
139.32
135.64
131.32
131.30
129.44
129.38
127.15
127.08
126.99
126.91
126.87
124.96
124.94
124.76
124.67
124.61
122.45
122.37
122.29
113.52
113.45
113.35
113.11
113.02
112.93
112.82
112.62
110.55
92.22
92.08
43.22
43.13
43.07
43.03
42.98
37.00
36.92
36.61
36.55
36.52
36.49
36.43
29.06
CN
Ad
Ad
F
Ad
Ad
CN
CN
Ad
Ad
22 (OMIM-7)
200 190 180 170 160 150 140 130 120 110 100 90 80 70 60 50 40 30 20 10 0

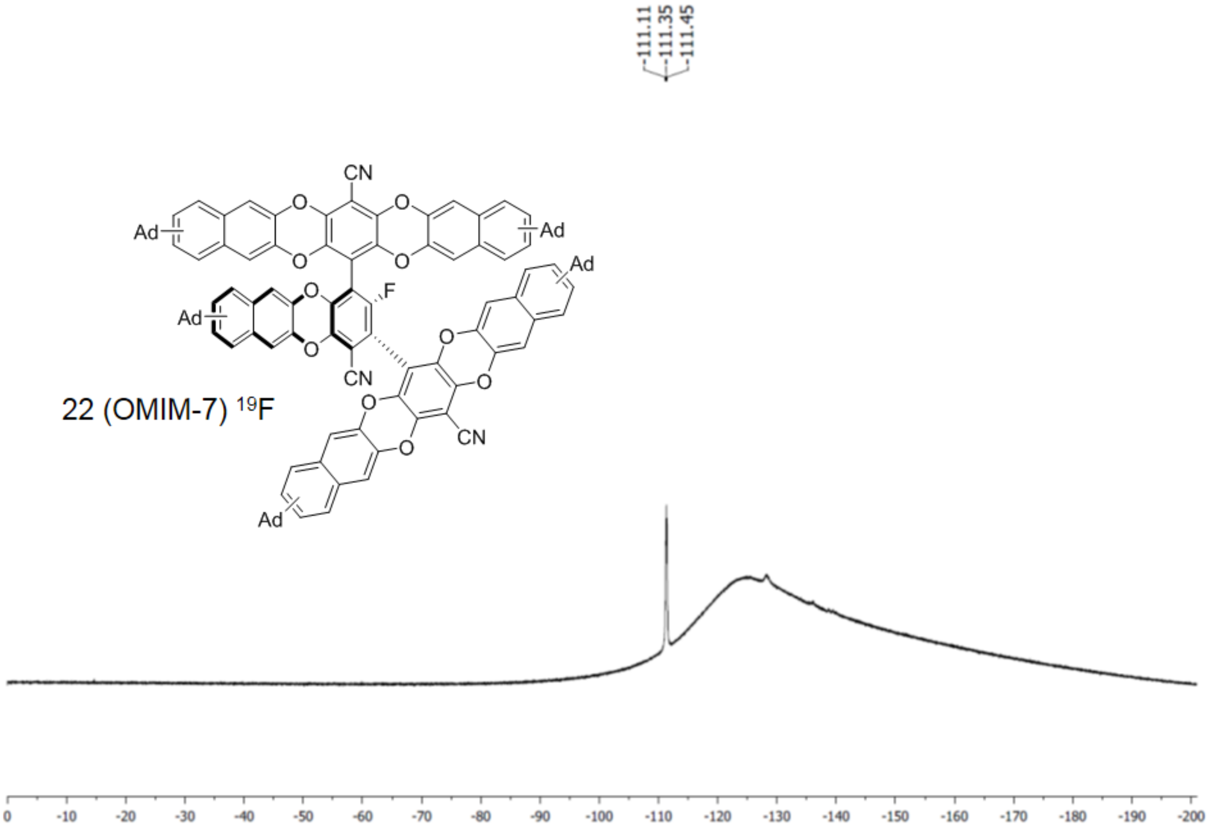
-111.11
-111.35
-111.45
CN
Ad
Ad
Ad
F
Ad
CN
22 (OMIM-7) 19F
CN
Ad
Ad
0 -10 -20 -30 -40 -50 -60 -70 -80 -90 -100 -110 -120 -130 -140 -150 -160 -170 -180 -190 -200

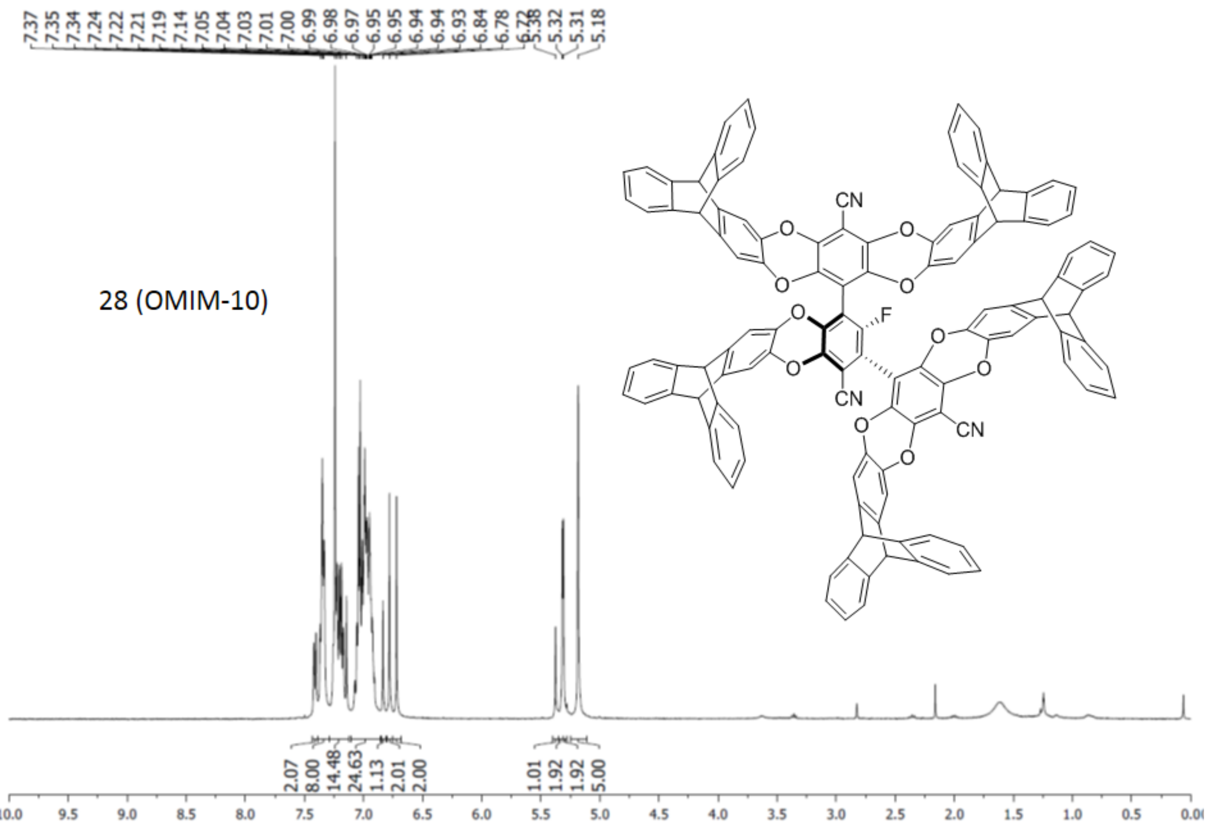
7.37 7.35 7.34 7.24 7.22 7.21 7.19 7.14 7.05 7.04 7.03 7.01 7.00 6.99 6.98 6.97 6.95 6.95 6.94 6.94 6.93 6.84 6.78 6.72 5.38 5.32 5.31 5.18
28 (OMIM-10)
CN
O
F
CN
2.07 8.00 14.48 24.63 1.13 2.01 2.00 1.01 1.92 1.92 5.00

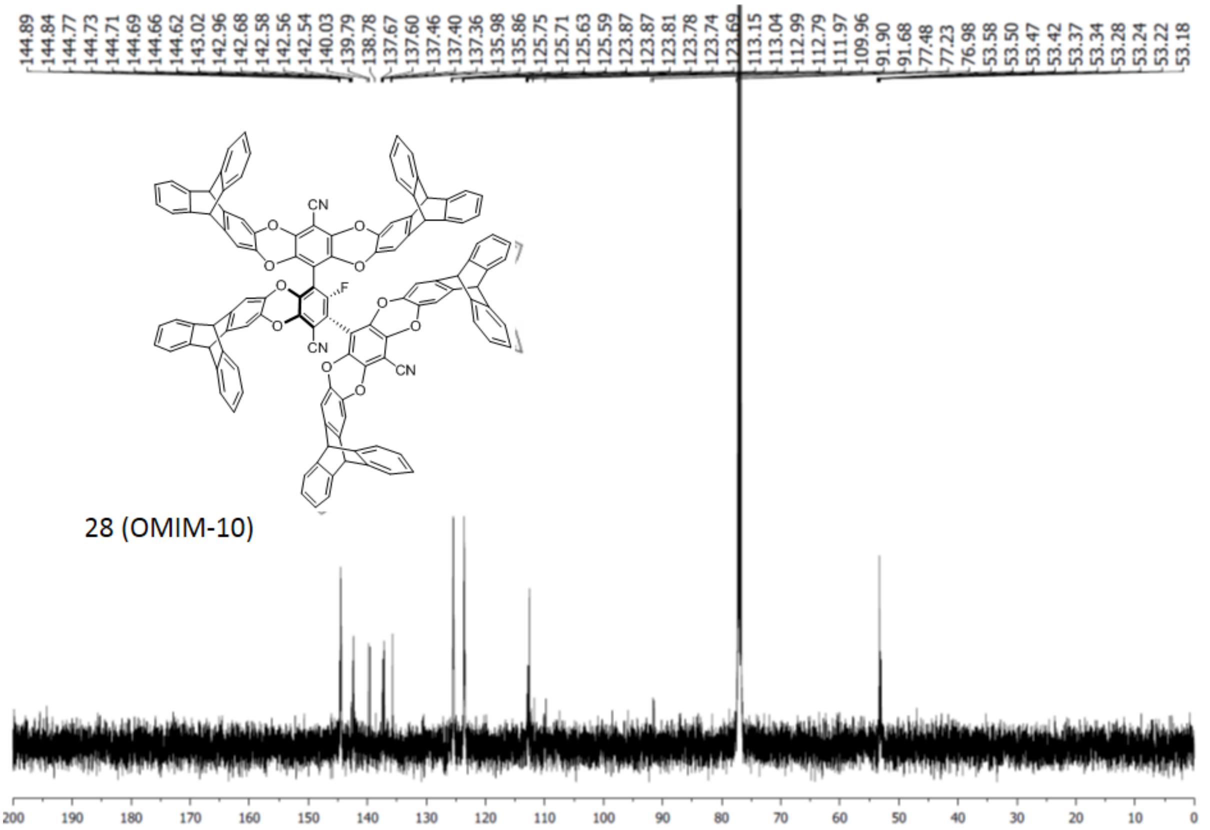
144.89 144.84 144.77 144.73 144.71 144.69 144.66 144.62 143.02 142.96 142.68 142.58 142.56 142.54 140.03 139.79 138.78 137.67 137.60 137.46 137.40 137.36 135.98 135.86 125.75 125.71 125.63 125.59 123.87 123.81 123.78 123.74 123.69 113.15 113.04 112.99 112.79 111.97 109.96 91.90 91.68 77.48 77.23 76.98 53.58 53.50 53.47 53.42 53.37 53.34 53.28 53.24 53.22 53.18
CN
O
F
CN
28 (OMIM-10)

28 (OMIM-10) ^{19}F

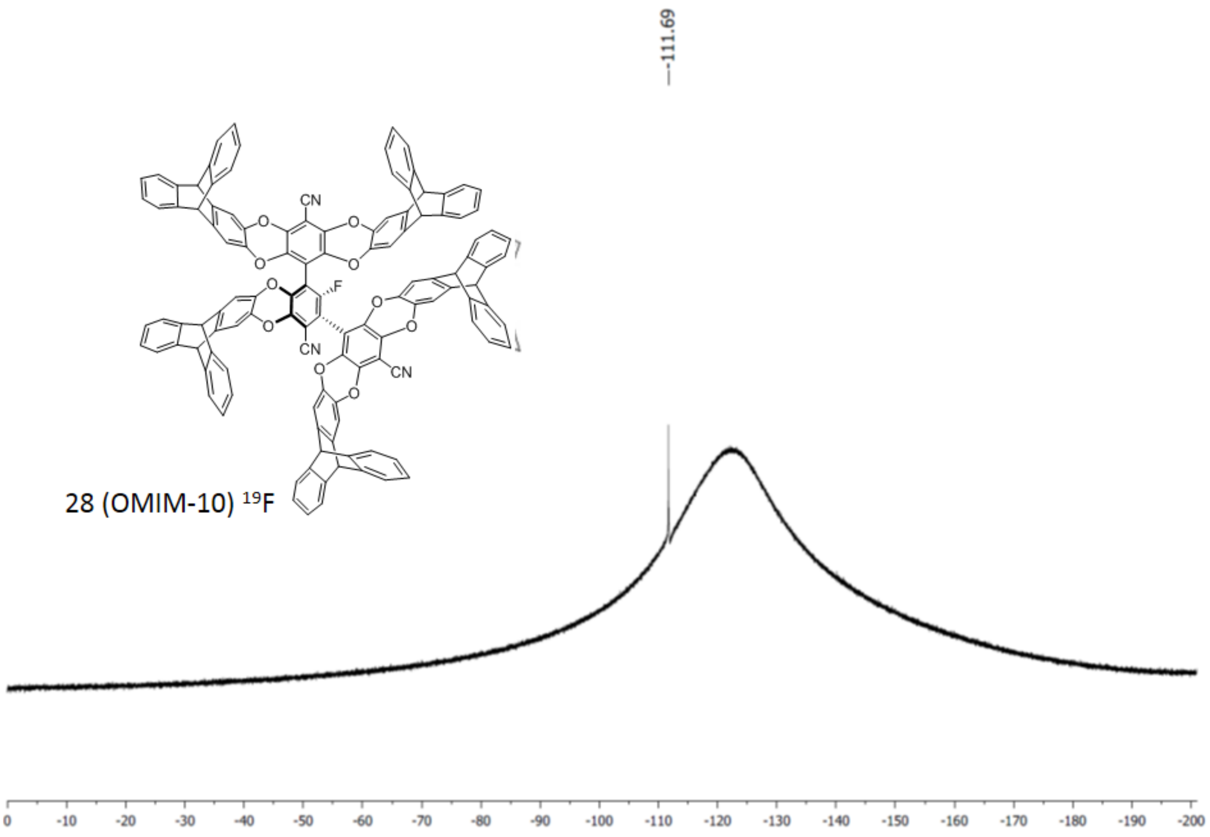

29 (OMIM-11)

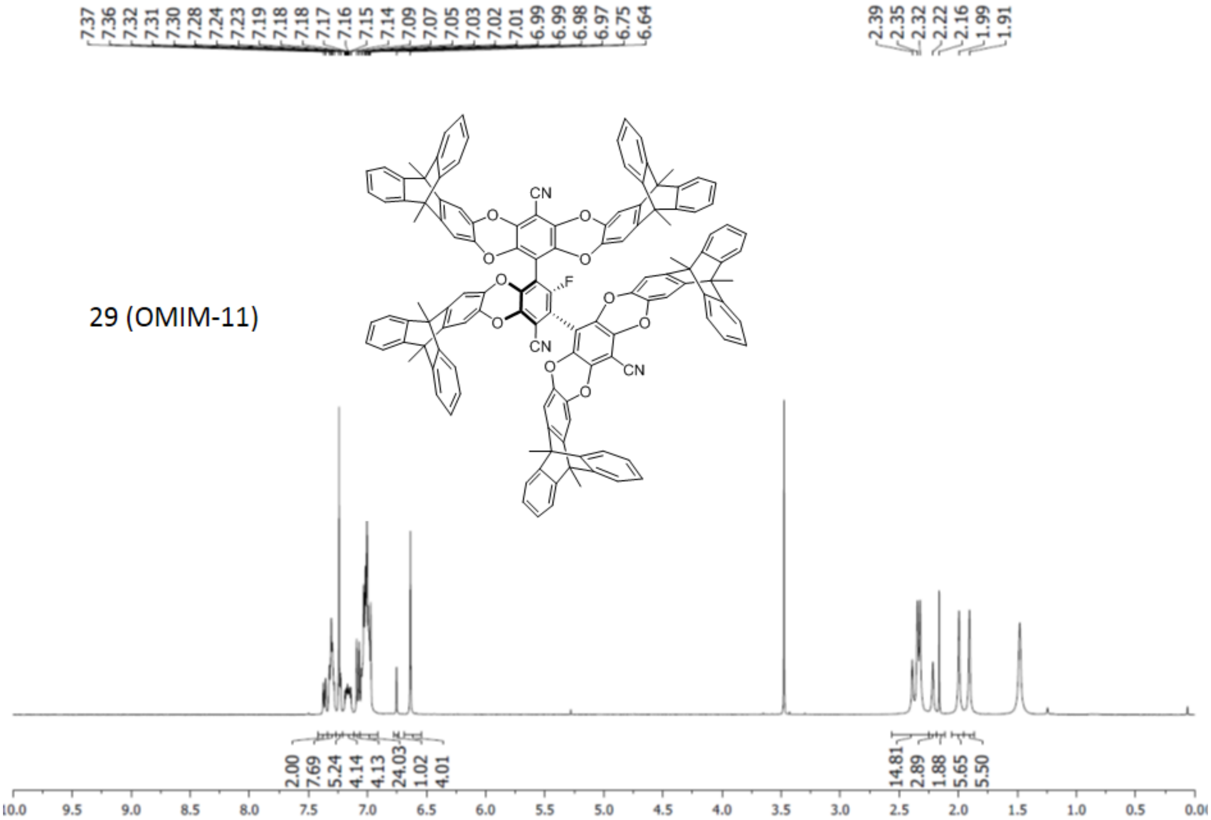

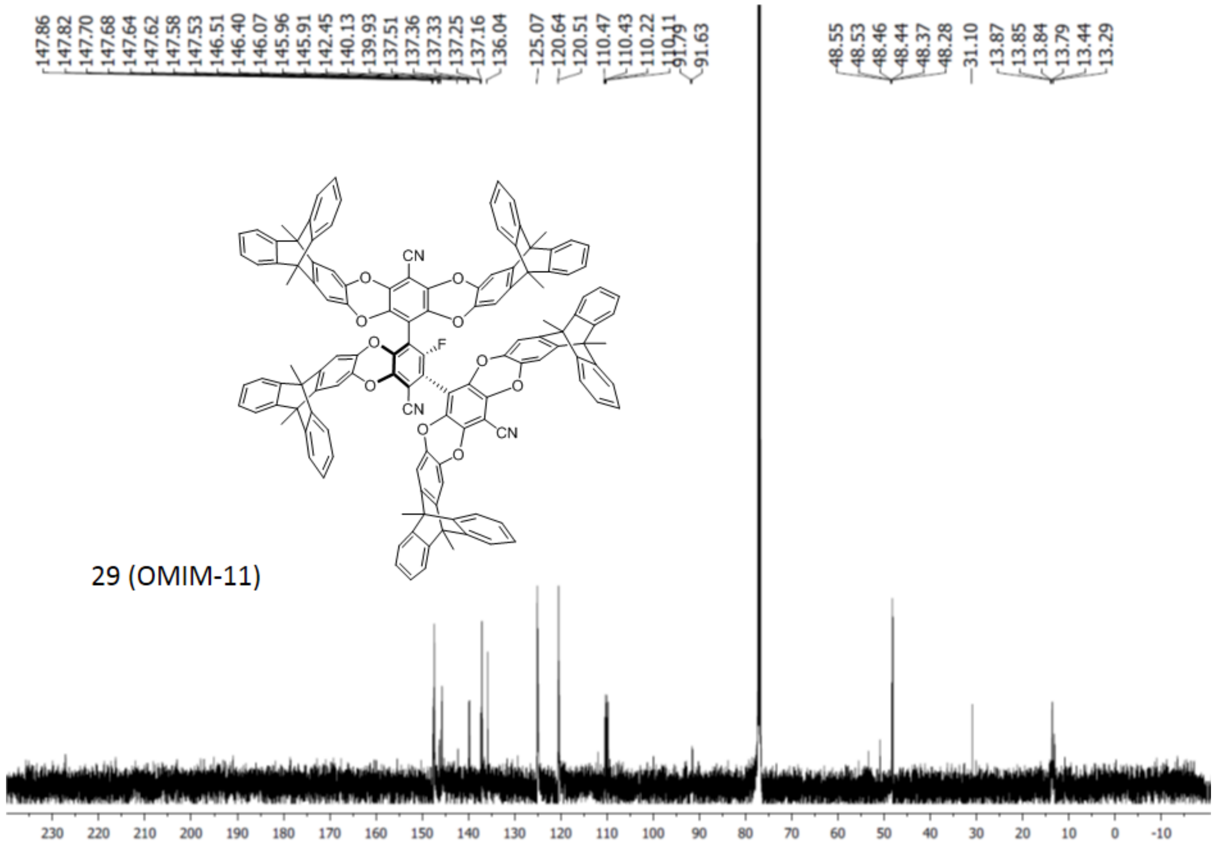

29 (OMIM-11)

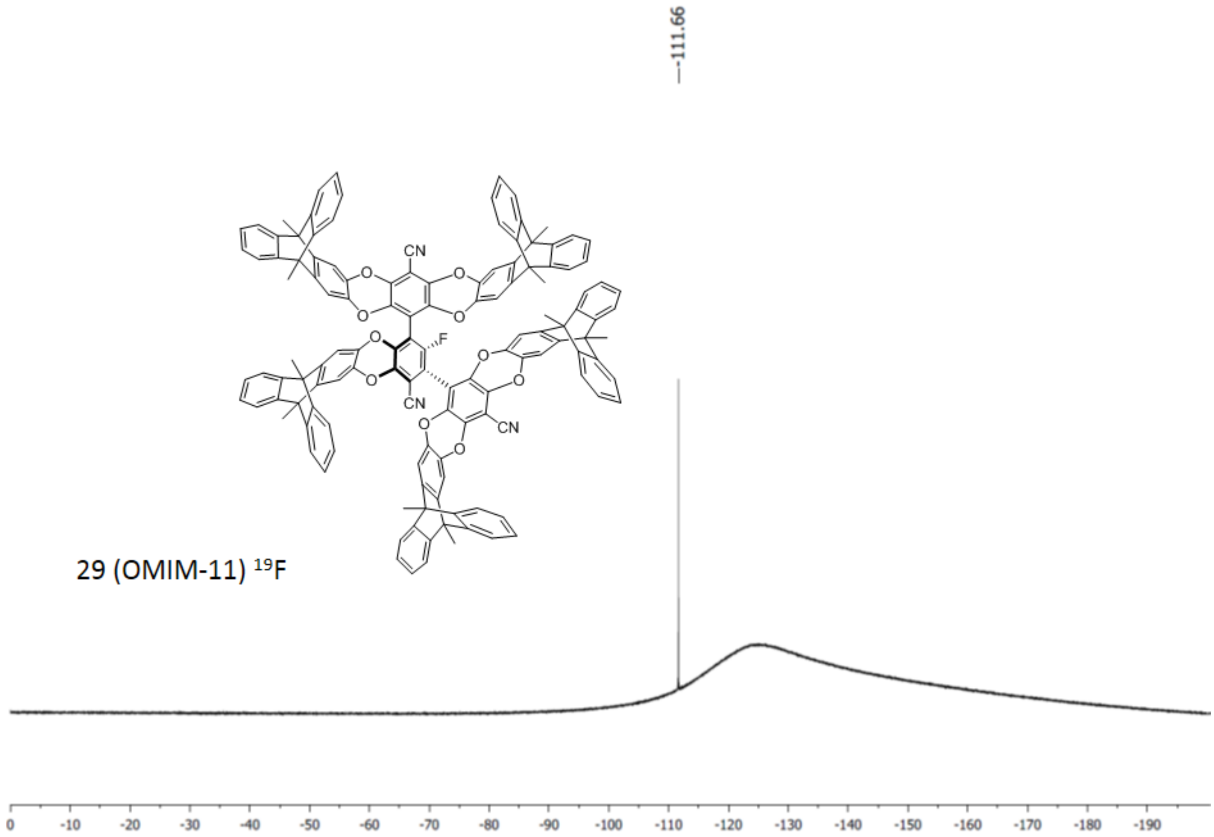

29 (OMIM-11) 19F

30 (OMIM-12)

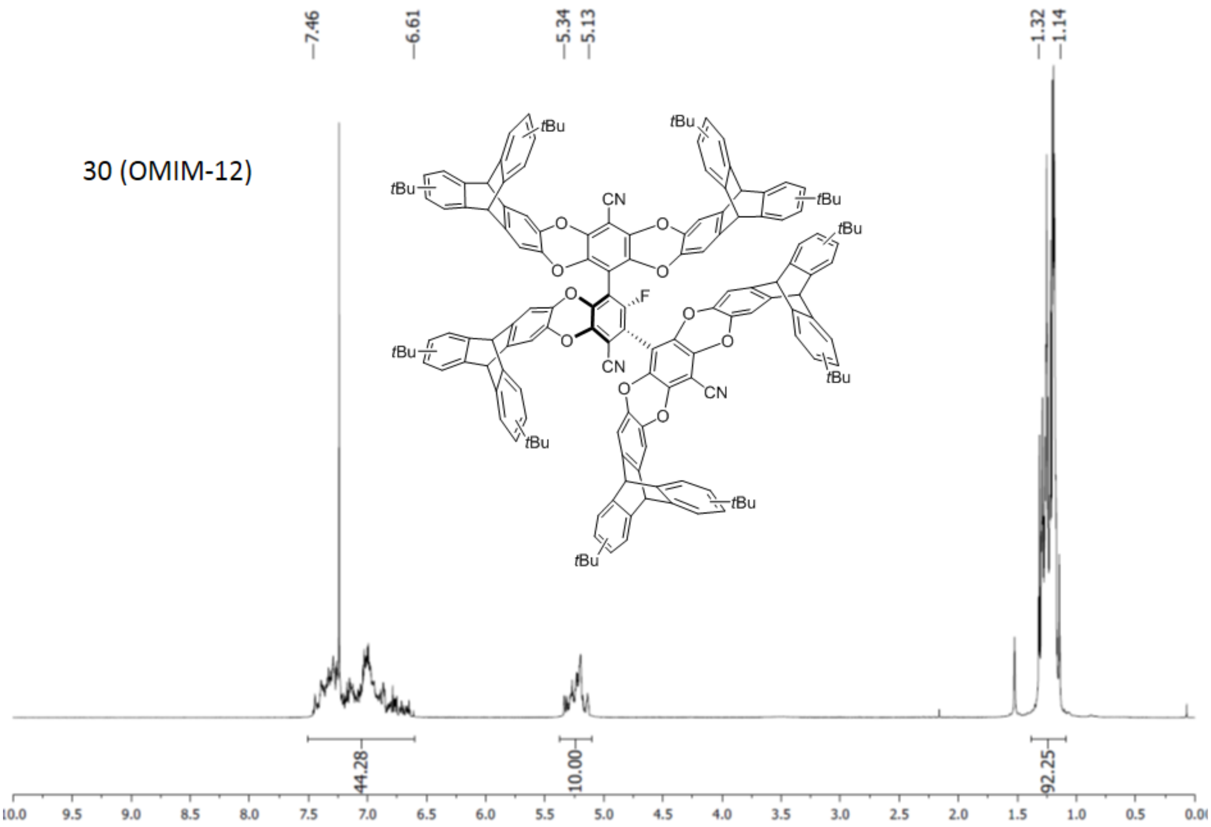

30 (OMIM-12)

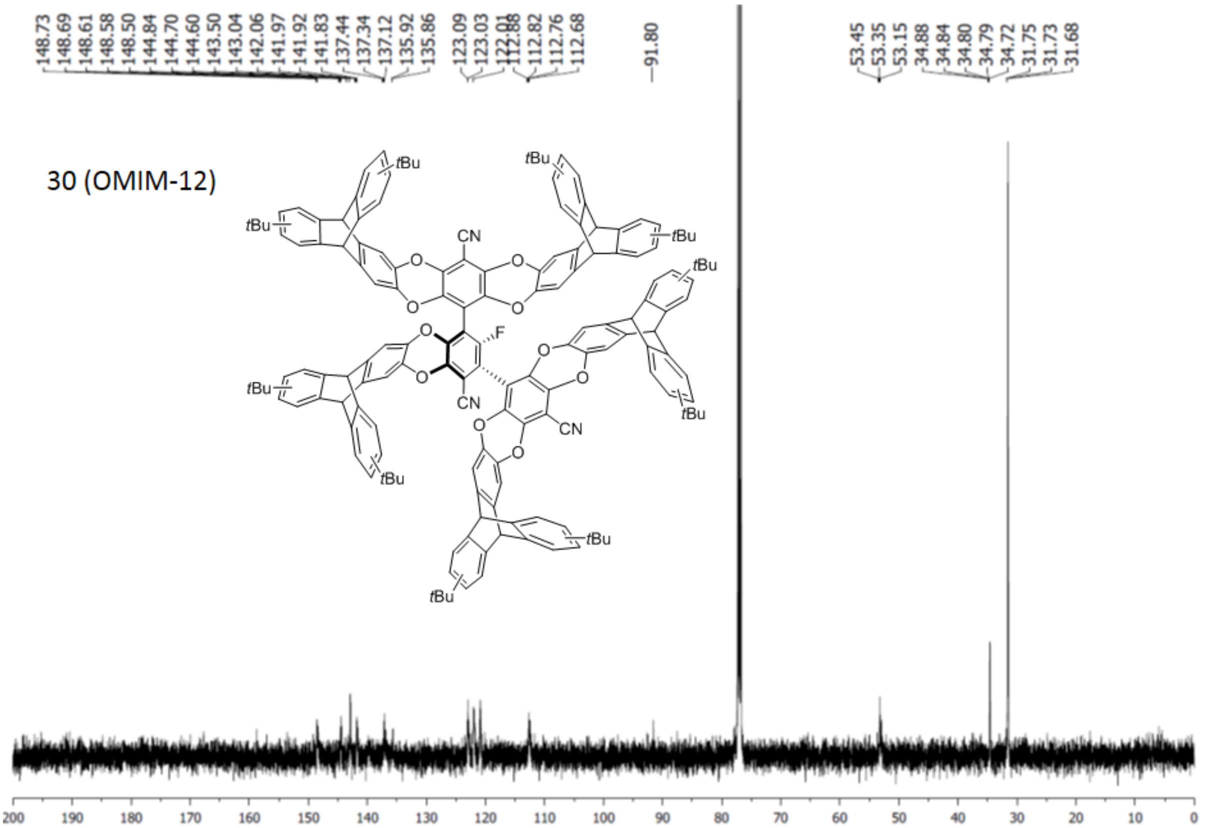

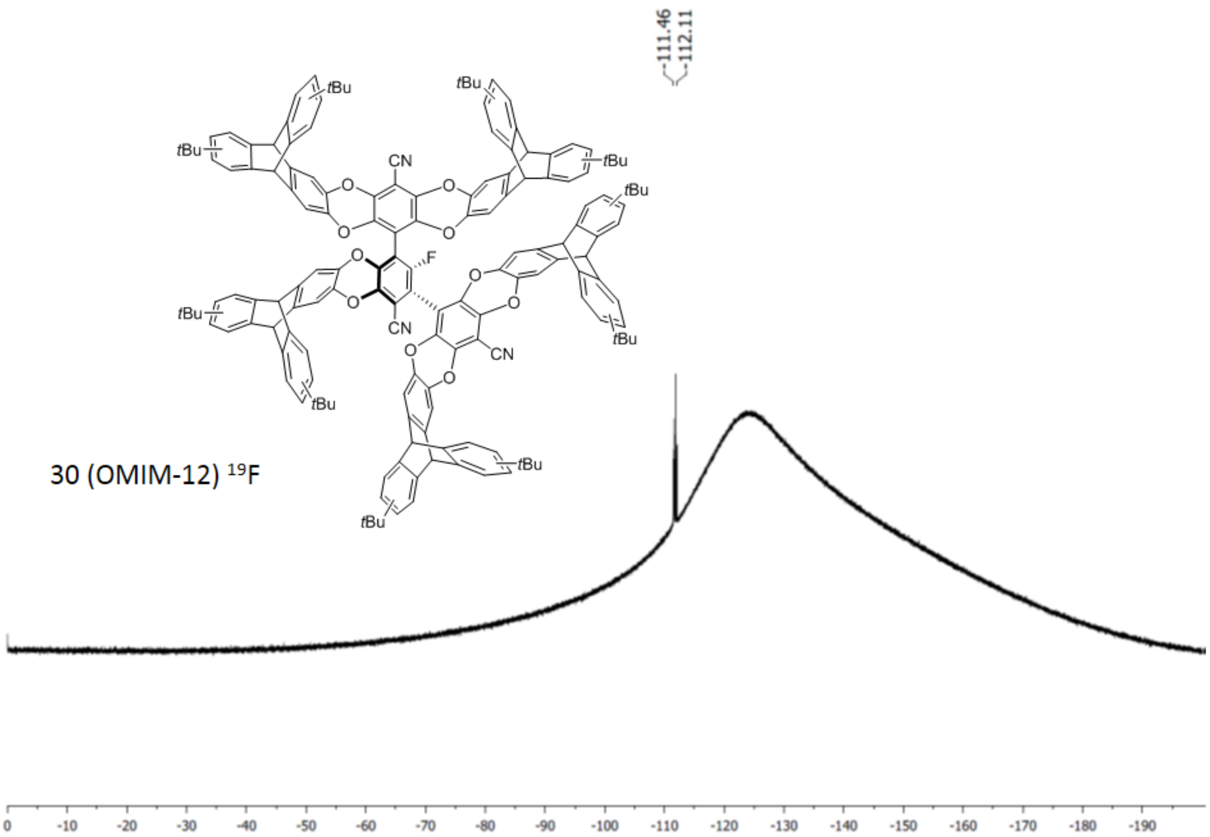

-111.46
-112.11
30 (OMIM-12) ^19F
CN
F
O
tBu

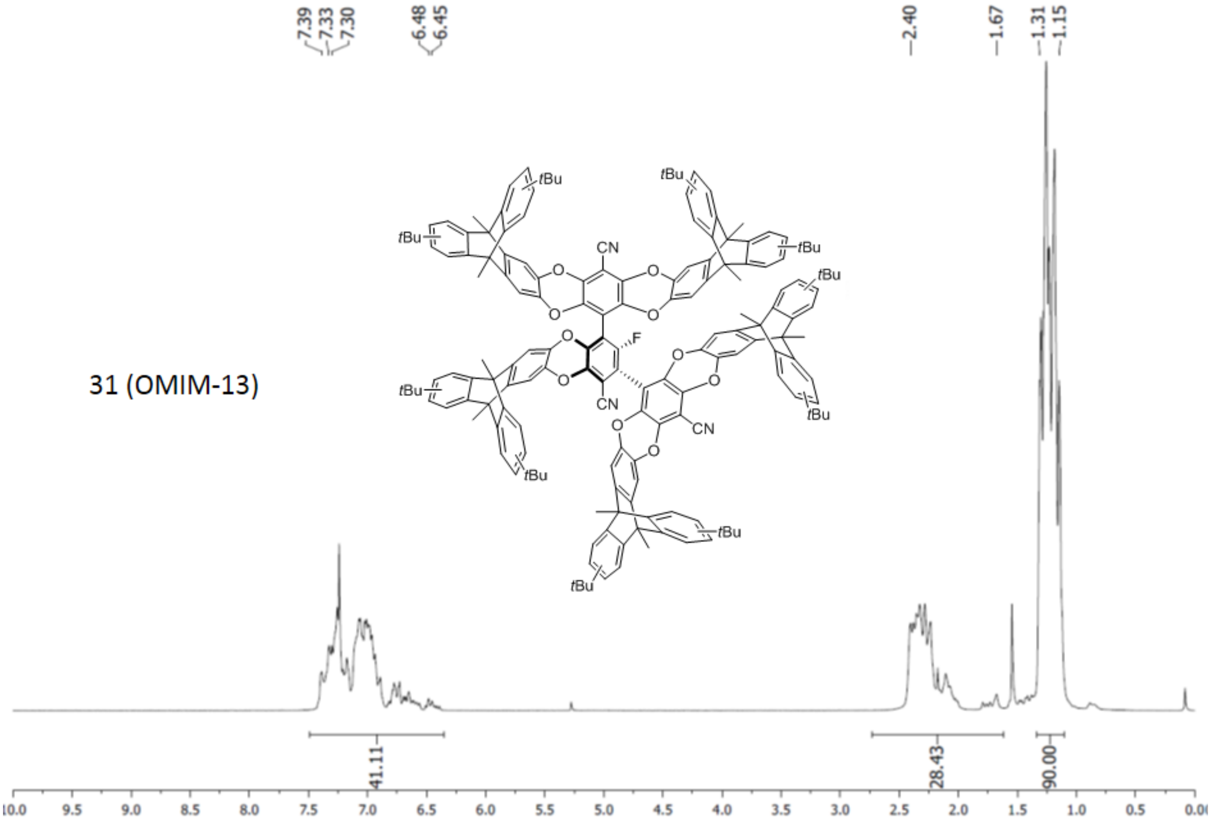

7.39
7.33
7.30
6.48
6.45
2.40
1.67
1.31
1.15
31 (OMIM-13)
41.11
28.43
90.00
CN
F
O
tBu

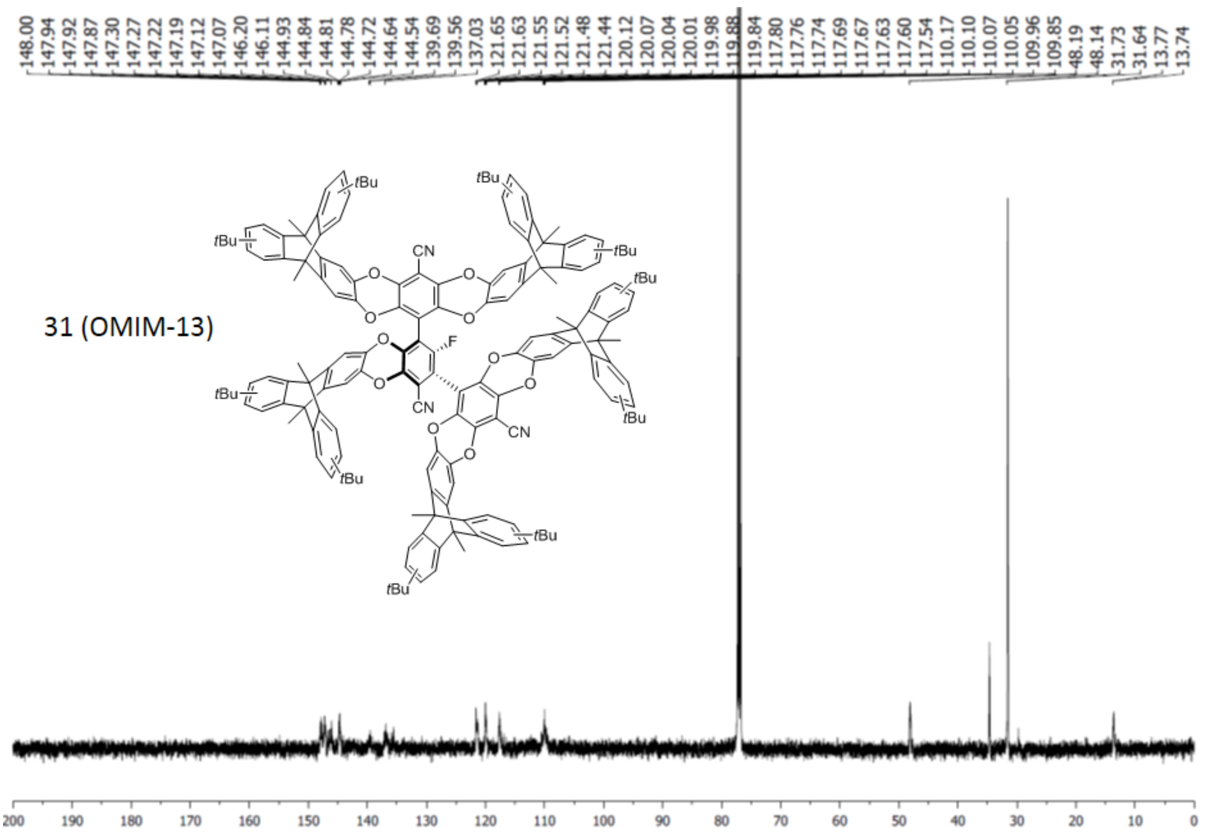
148.00
147.94
147.92
147.87
147.30
147.27
147.22
147.19
147.12
147.07
146.20
146.11
144.93
144.84
144.81
144.78
144.72
144.64
144.54
139.69
139.56
137.03
121.65
121.63
121.55
121.52
121.48
121.44
120.12
120.07
120.04
120.01
119.98
119.88
119.84
117.80
117.76
117.74
117.69
117.67
117.63
117.60
117.54
110.17
110.10
110.07
110.05
109.96
109.85
48.19
48.14
31.73
31.64
13.77
13.74
31 (OMIM-13)
CN
F
tBu
200 190 180 170 160 150 140 130 120 110 100 90 80 70 60 50 40 30 20 10 0

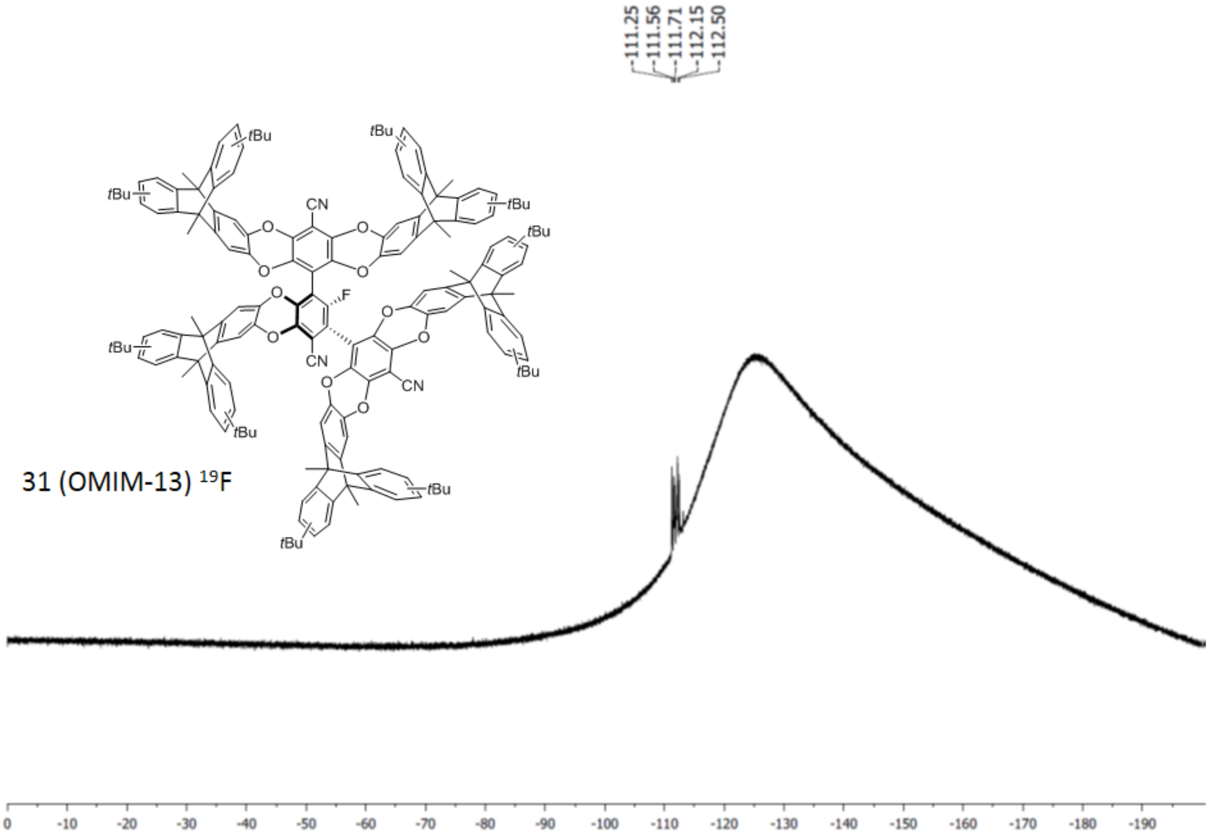
-111.25
-111.56
-111.71
-112.15
-112.50
31 (OMIM-13) 19F
CN
F
tBu
0 -10 -20 -30 -40 -50 -60 -70 -80 -90 -100 -110 -120 -130 -140 -150 -160 -170 -180 -190

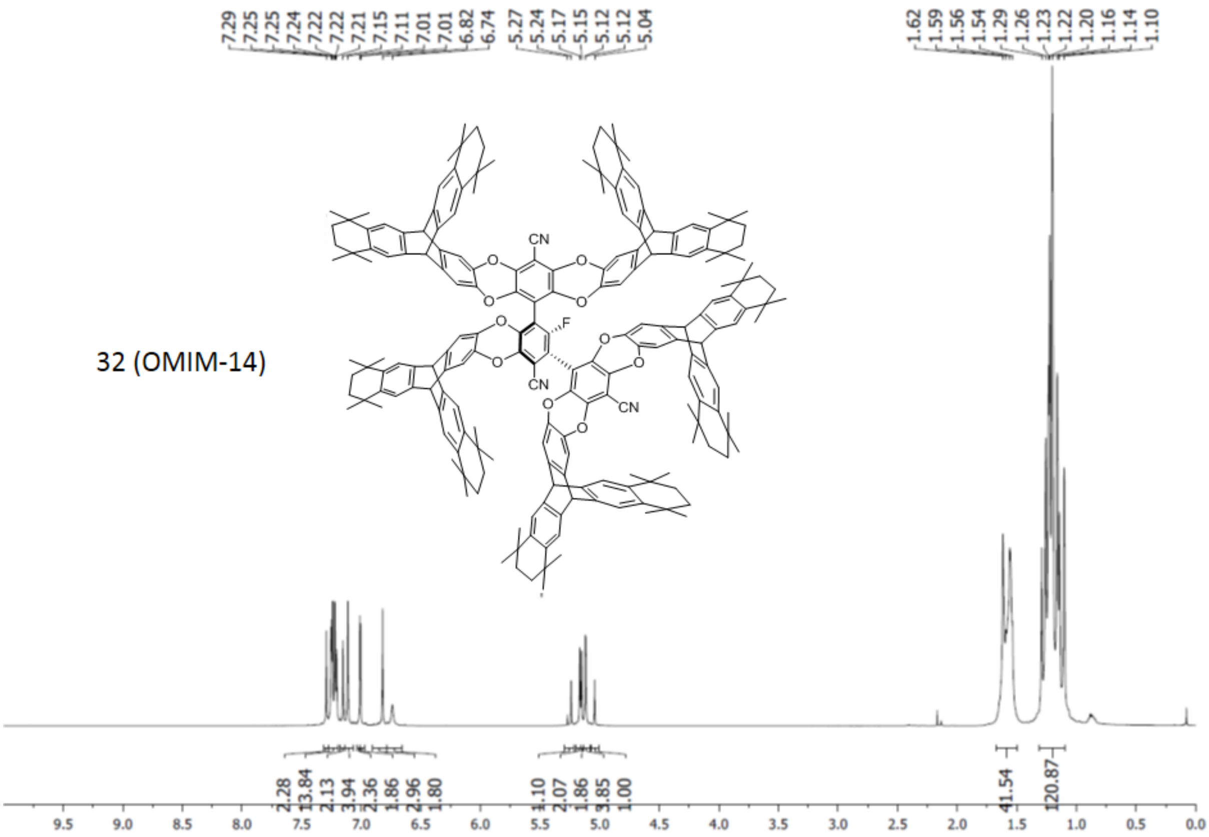
7.29 7.25 7.25 7.24 7.22 7.22 7.21 7.15 7.11 7.01 7.01 6.82 6.74 5.27 5.24 5.17 5.15 5.12 5.12 5.04
1.62 1.59 1.56 1.54 1.29 1.26 1.23 1.22 1.20 1.16 1.14 1.10
32 (OMIM-14)
2.28 13.84 2.13 3.94 2.36 1.86 2.96 1.80 1.10 2.07 1.86 3.85 1.00 41.54 120.87
9.5 9.0 8.5 8.0 7.5 7.0 6.5 6.0 5.5 5.0 4.5 4.0 3.5 3.0 2.5 2.0 1.5 1.0 0.5 0.0

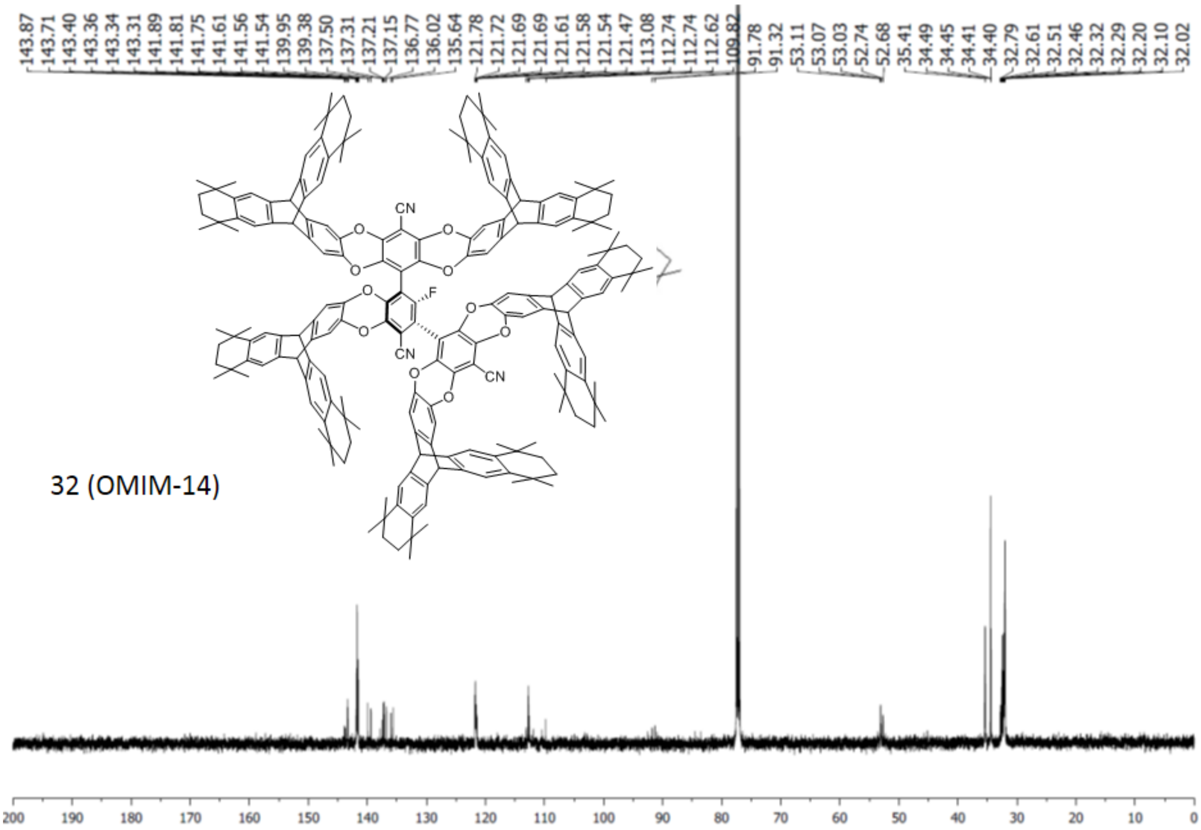
143.87 143.71 143.40 143.36 143.34 143.31 141.89 141.81 141.75 141.61 141.56 141.54 139.95 139.38 137.50 137.31 137.21 137.15 136.77 136.02 135.64 121.78 121.72 121.69 121.69 121.61 121.58 121.54 121.47 113.08 112.74 112.74 112.62 109.82 91.78 91.32 53.11 53.07 53.03 52.74 52.68 35.41 34.49 34.45 34.41 34.40 32.79 32.61 32.51 32.46 32.32 32.29 32.20 32.10 32.02
32 (OMIM-14)
200 190 180 170 160 150 140 130 120 110 100 90 80 70 60 50 40 30 20 10 0

32 (OMIM-14) ^{19}F

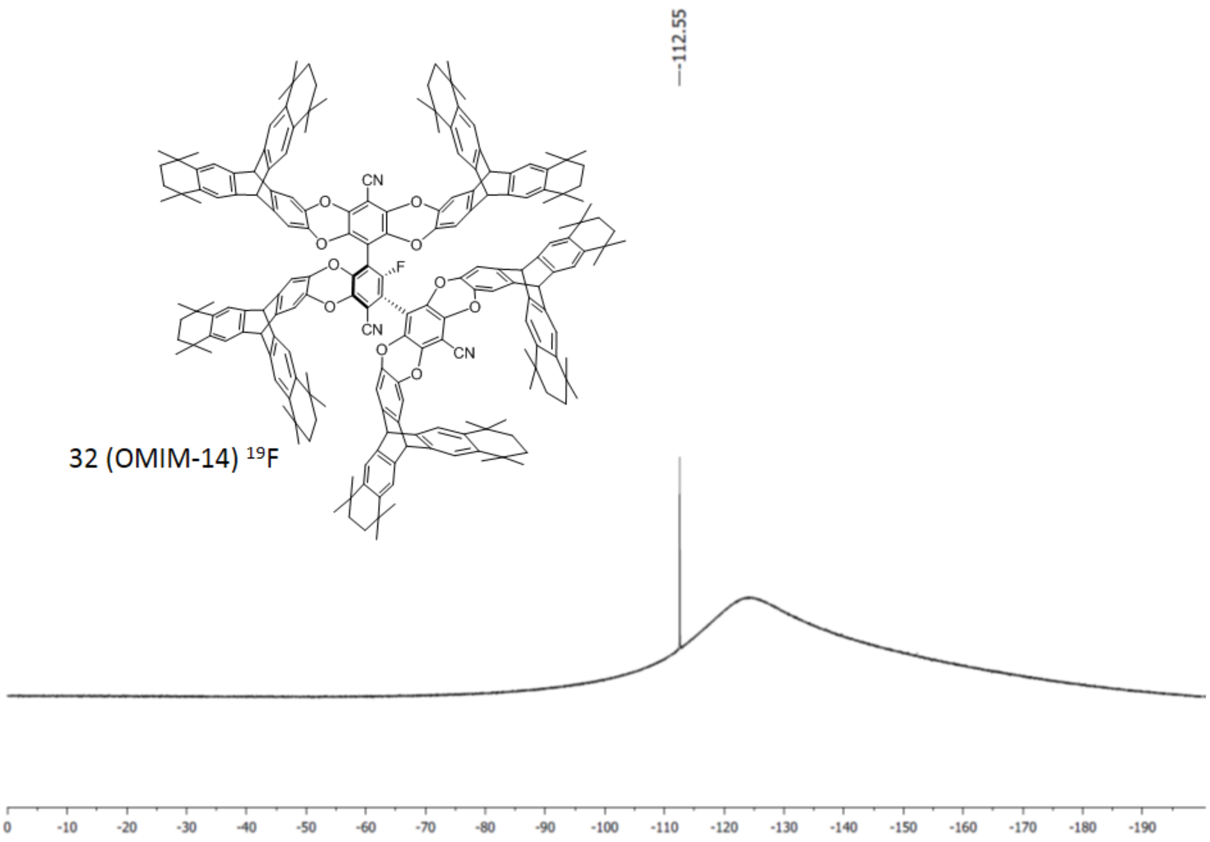

37 (OMIM-16)

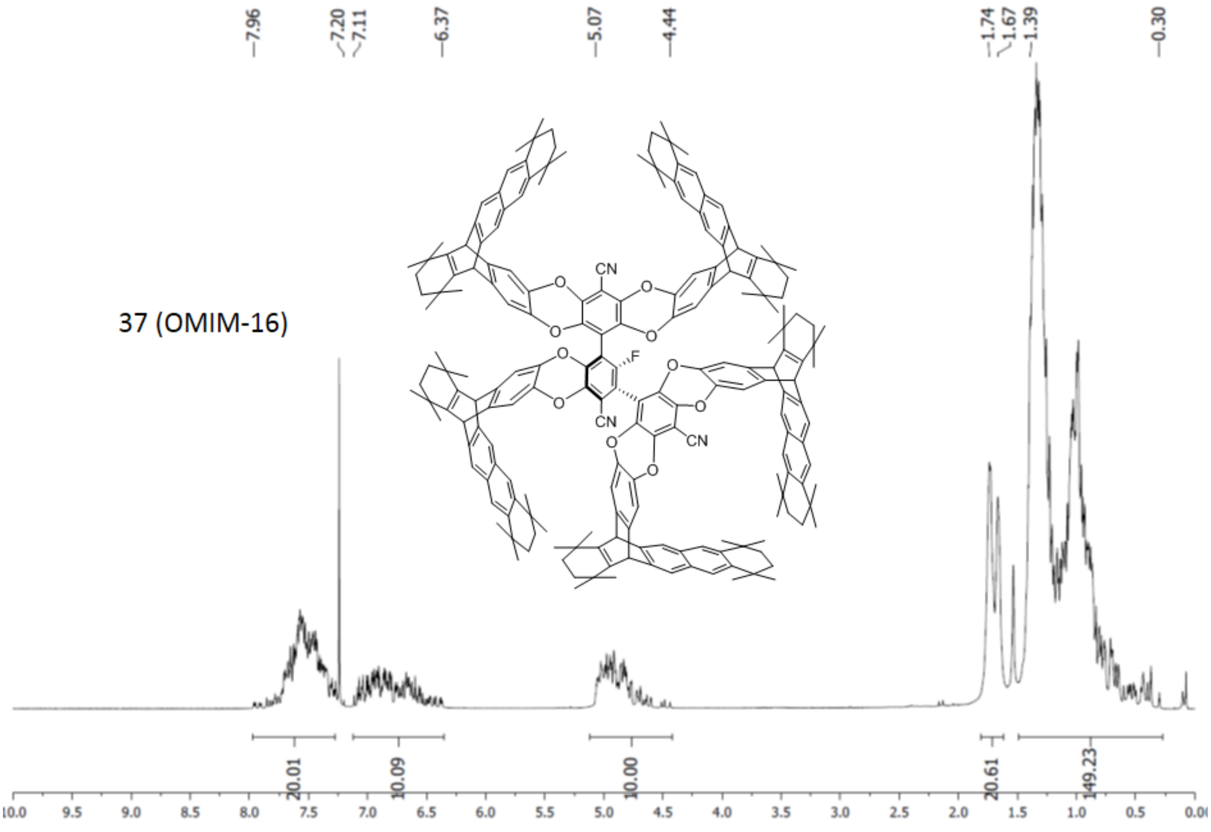

37 (OMIM-16)

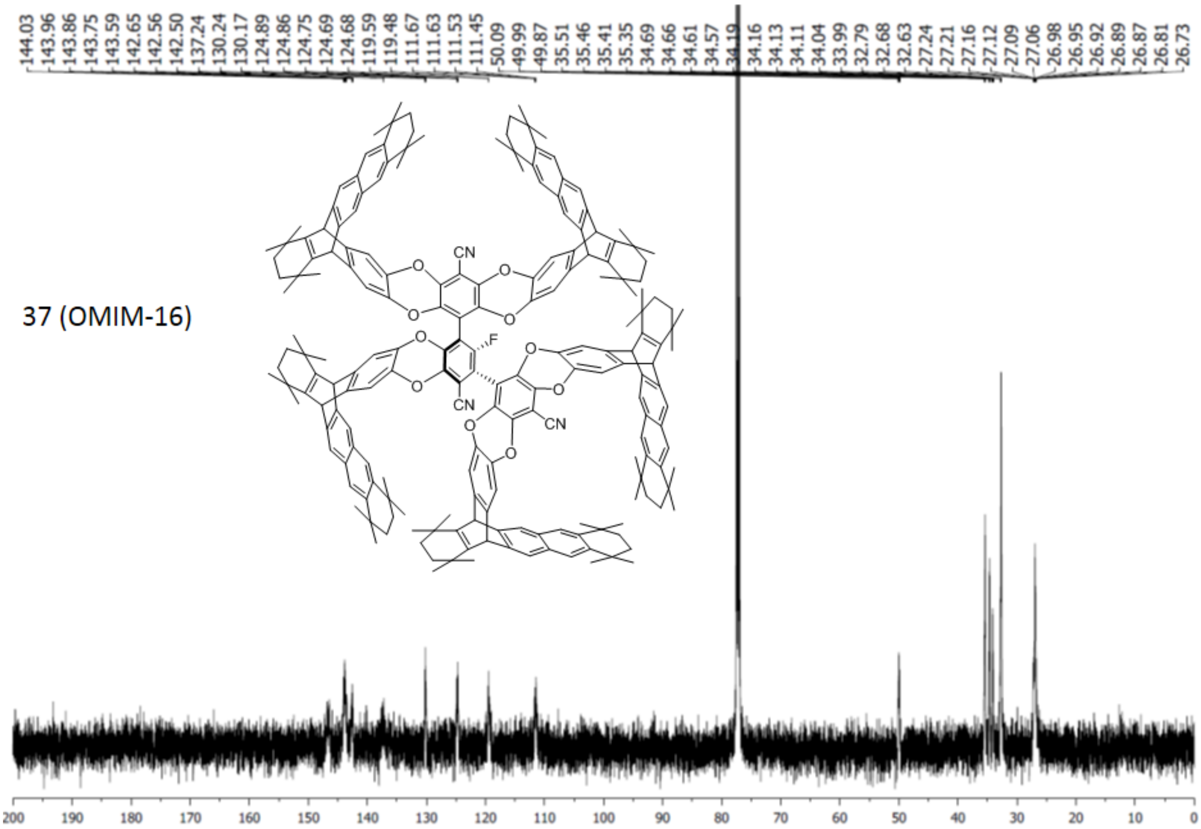

37 (OMIM-16) ^{19}F

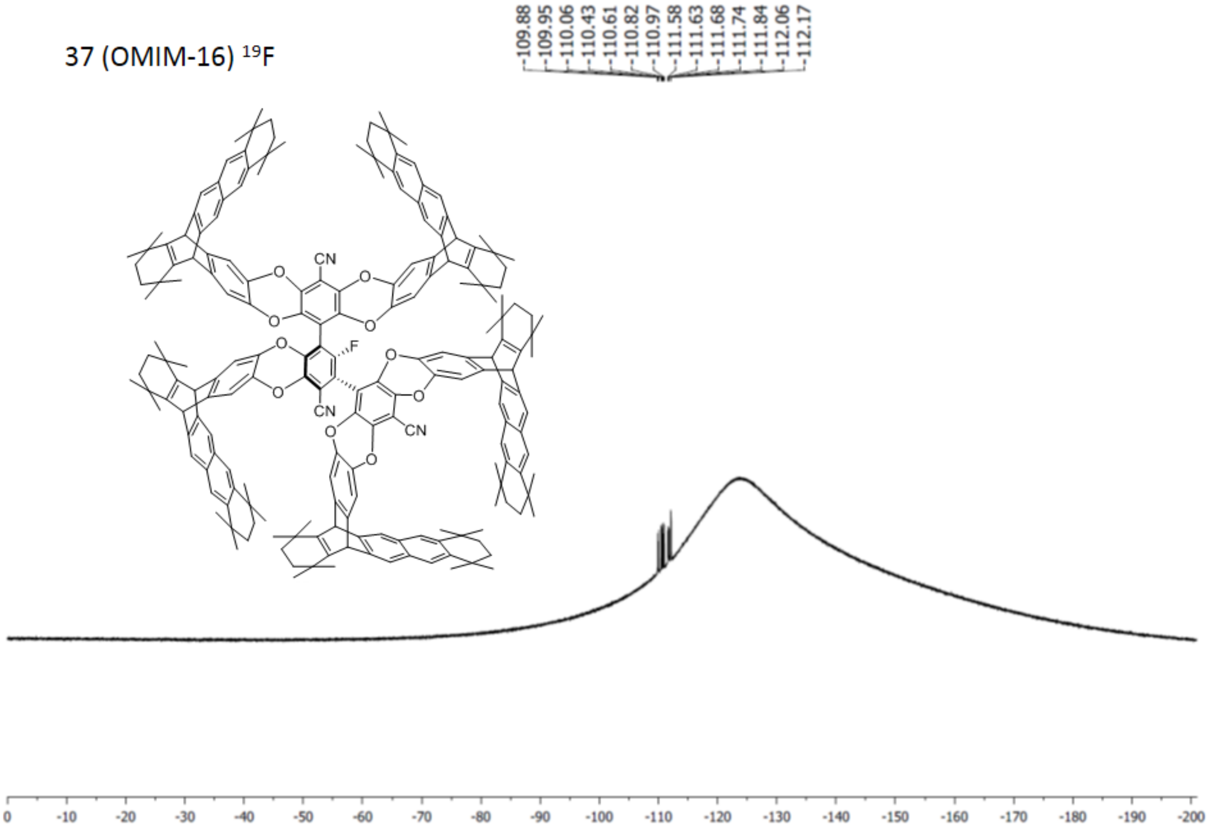

38 (OMIM-17)

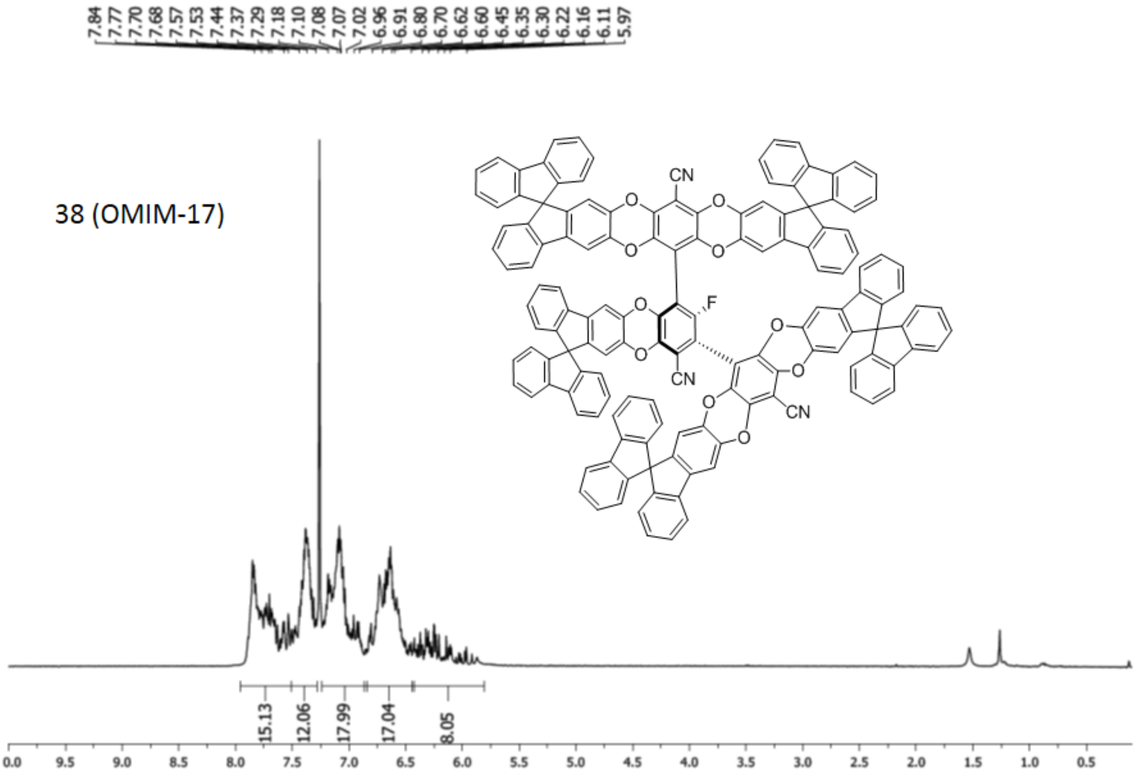

38 (OMIM-17)

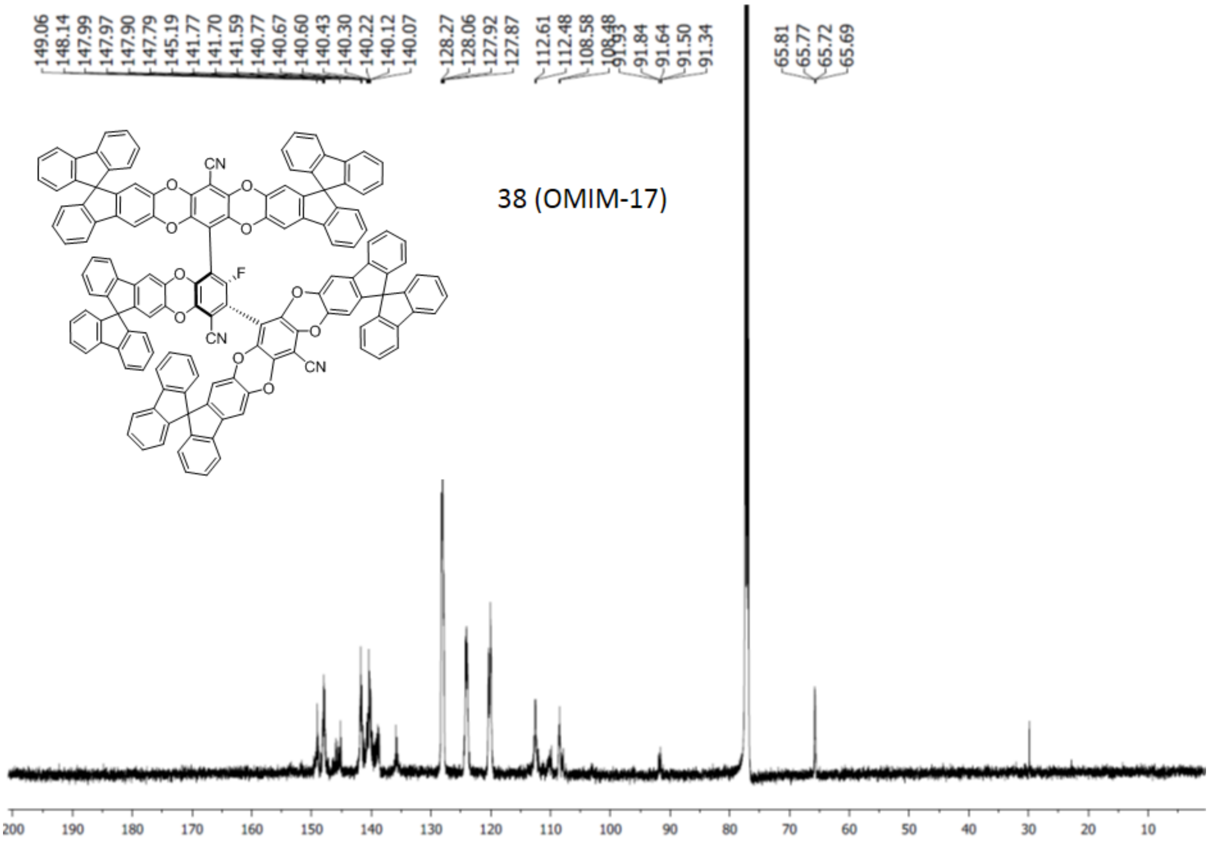

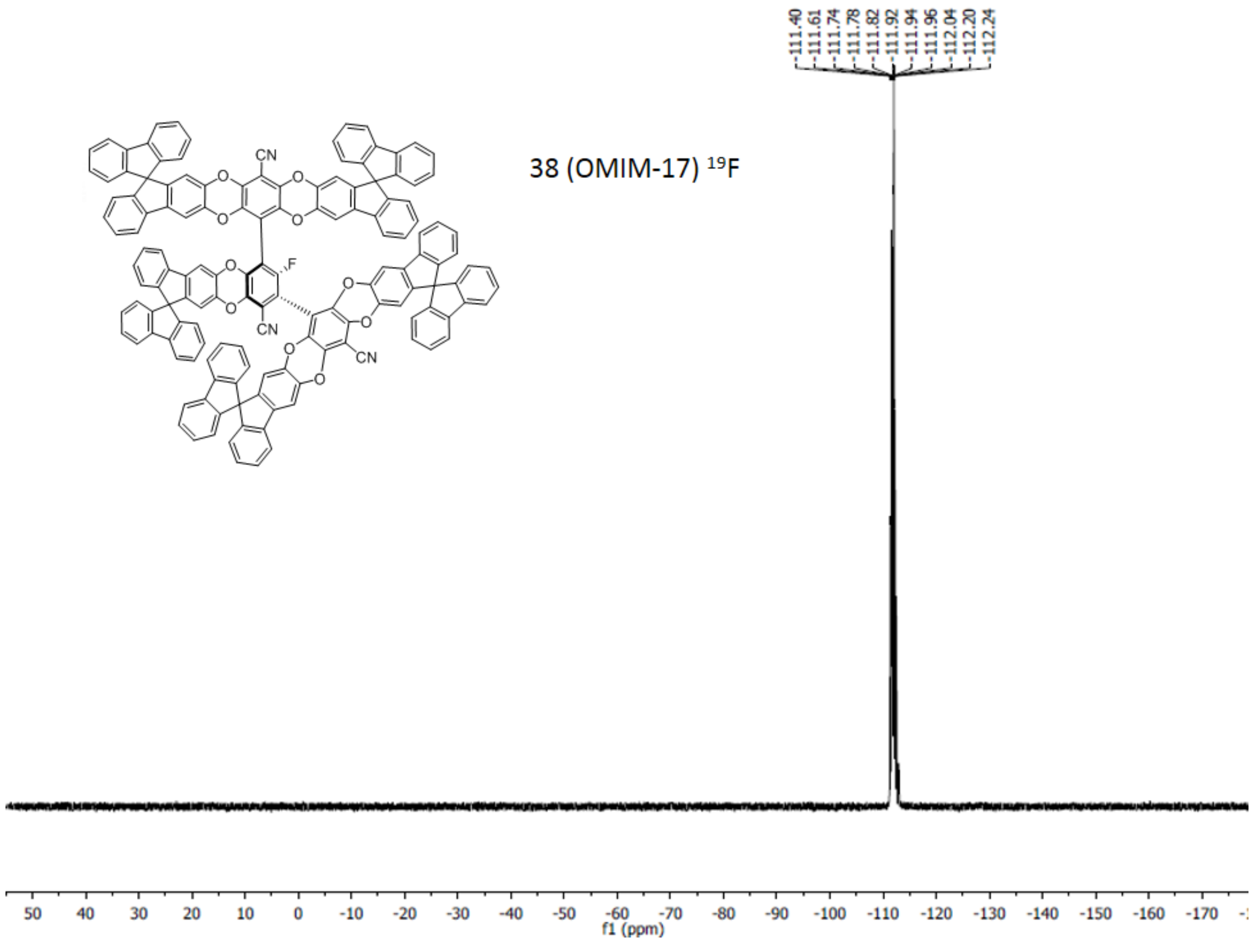
38 (OMIM-17) ¹⁹F
-111.40
-111.61
-111.74
-111.78
-111.82
-111.92
-111.94
-111.96
-112.04
-112.20
-112.24
f1 (ppm)

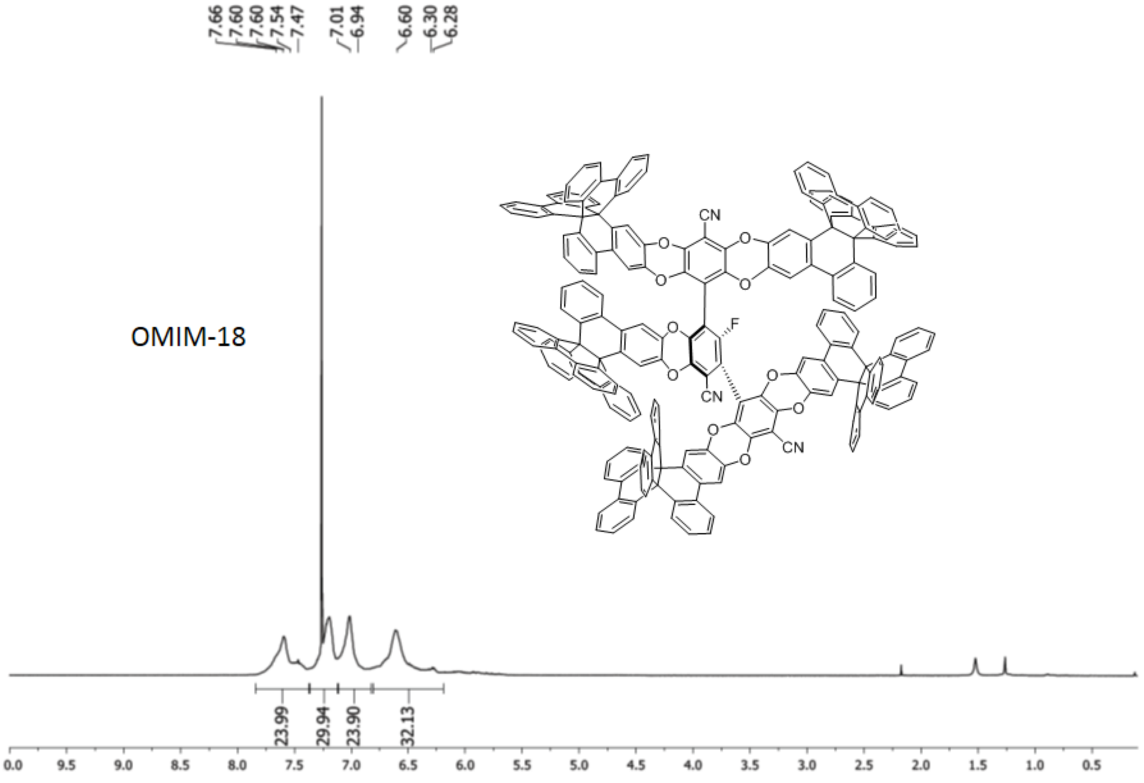
OMIM-18
7.66
7.60
7.60
7.54
7.47
7.01
6.94
6.60
6.30
6.28
23.99
29.94
23.90
32.13

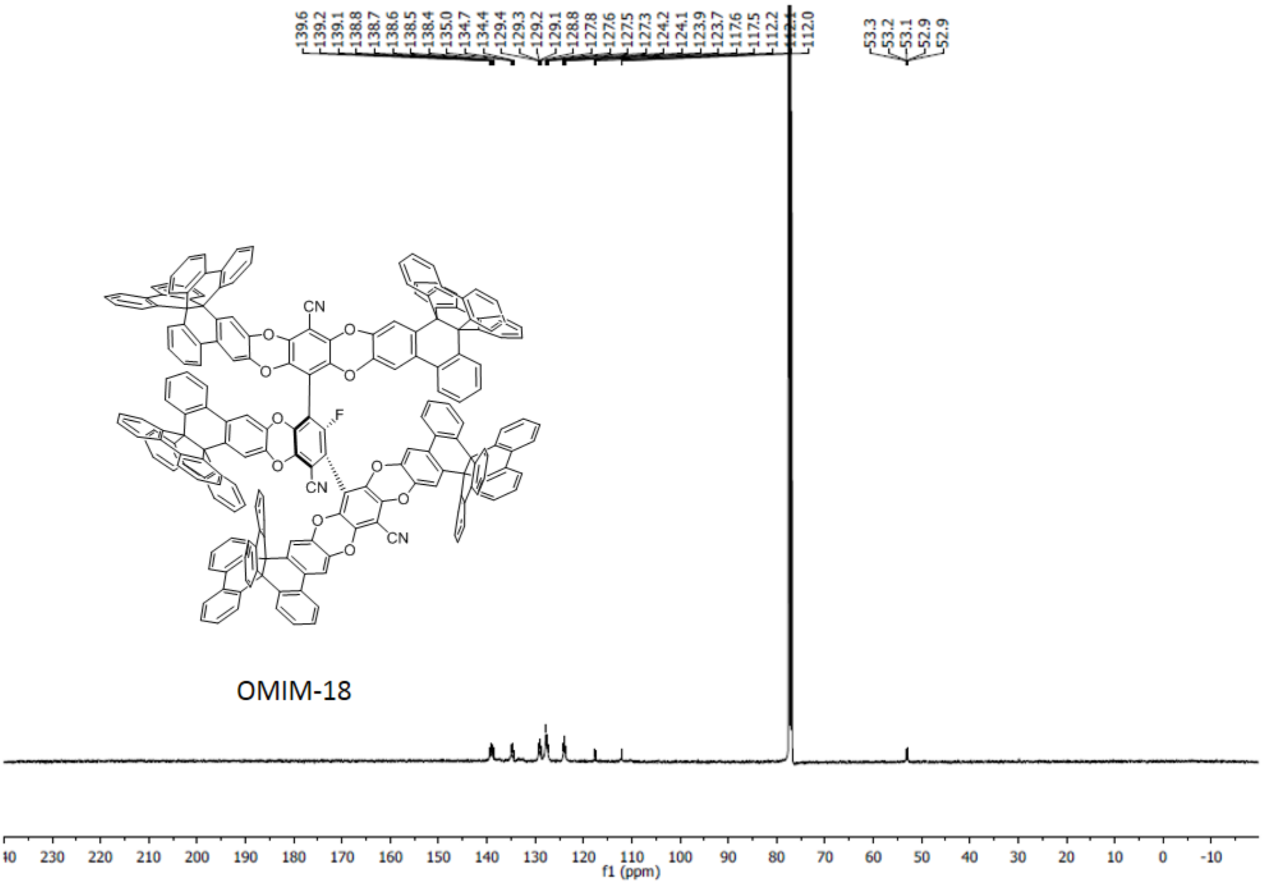
139.6
139.2
139.1
138.8
138.7
138.6
138.5
138.4
135.0
134.7
134.4
129.4
129.3
129.2
129.1
128.8
127.8
127.6
127.5
127.3
124.2
124.1
123.9
123.7
117.6
117.5
112.2
112.0
53.3
53.2
53.1
52.9
52.9
CN
F
CN
CN
OMIM-18
f1 (ppm)

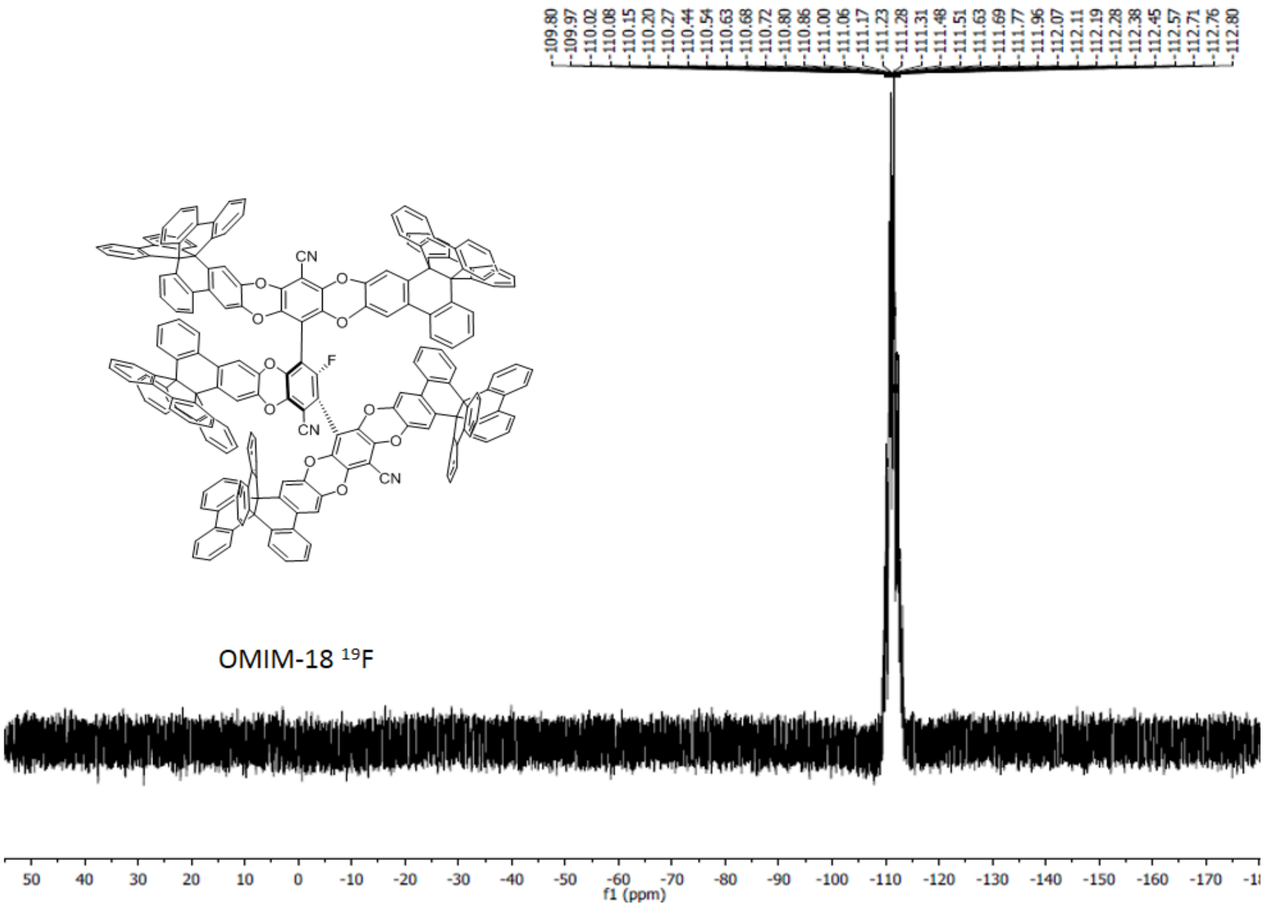
-109.80
-109.97
-110.02
-110.08
-110.15
-110.20
-110.27
-110.44
-110.54
-110.63
-110.68
-110.72
-110.80
-110.86
-111.00
-111.06
-111.17
-111.23
-111.28
-111.31
-111.48
-111.51
-111.63
-111.69
-111.77
-111.96
-112.07
-112.11
-112.19
-112.28
-112.38
-112.45
-112.57
-112.71
-112.76
-112.80
CN
F
CN
CN
OMIM-18 19F
f1 (ppm)

Mass Spectrometry Data

Single Mass Analysis (displaying only valid results)
Tolerance = 5.0 PPM / DBE: min = -2.0, max = 100.0
Element prediction: Off

Monoisotopic Mass, Odd and Even Electron Ions
16 formula(e) evaluated with 1 results within limits (all results (up to 1000) for each mass)
Elements Used:
C: 0-38 H: 0-16 N: 0-2 O: 0-8
R TAYLOR
20-Jun-2011
NBM_MS9145 321 (5.350) Cm (321-38x1.200)

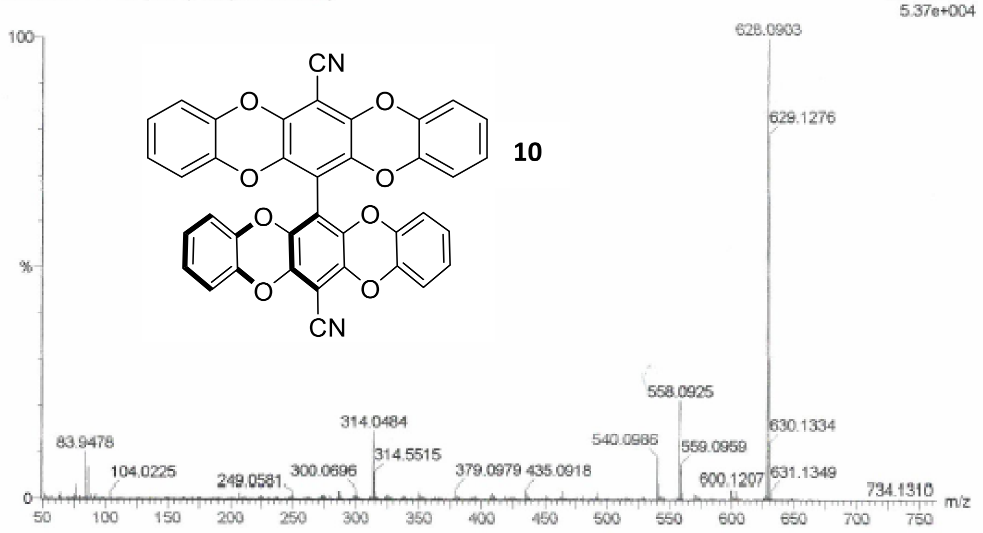

Minimum:				-2.0		
Maximum:		10.0	5.0	100.0		
Mass	Calc. Mass	mDa	PPM	DBE	i-FIT	Formula
628.0903	628.0907	-0.4	-0.6	32.0	3853.4	C38 H16 N2 O8

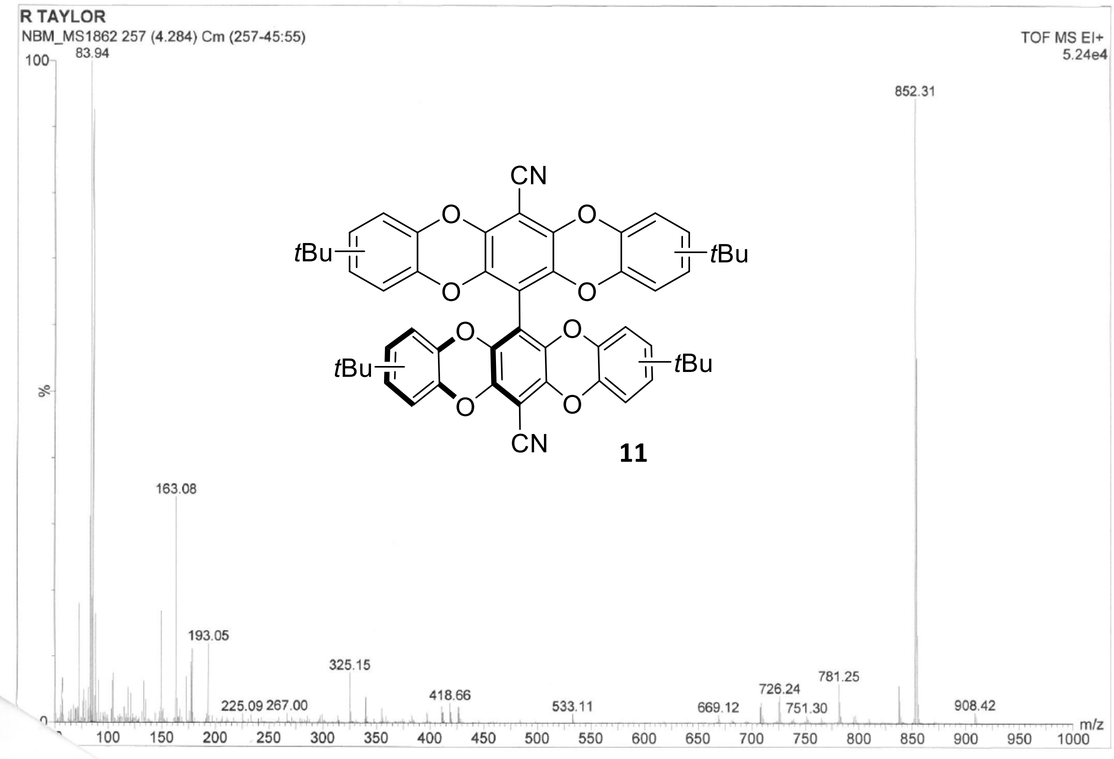

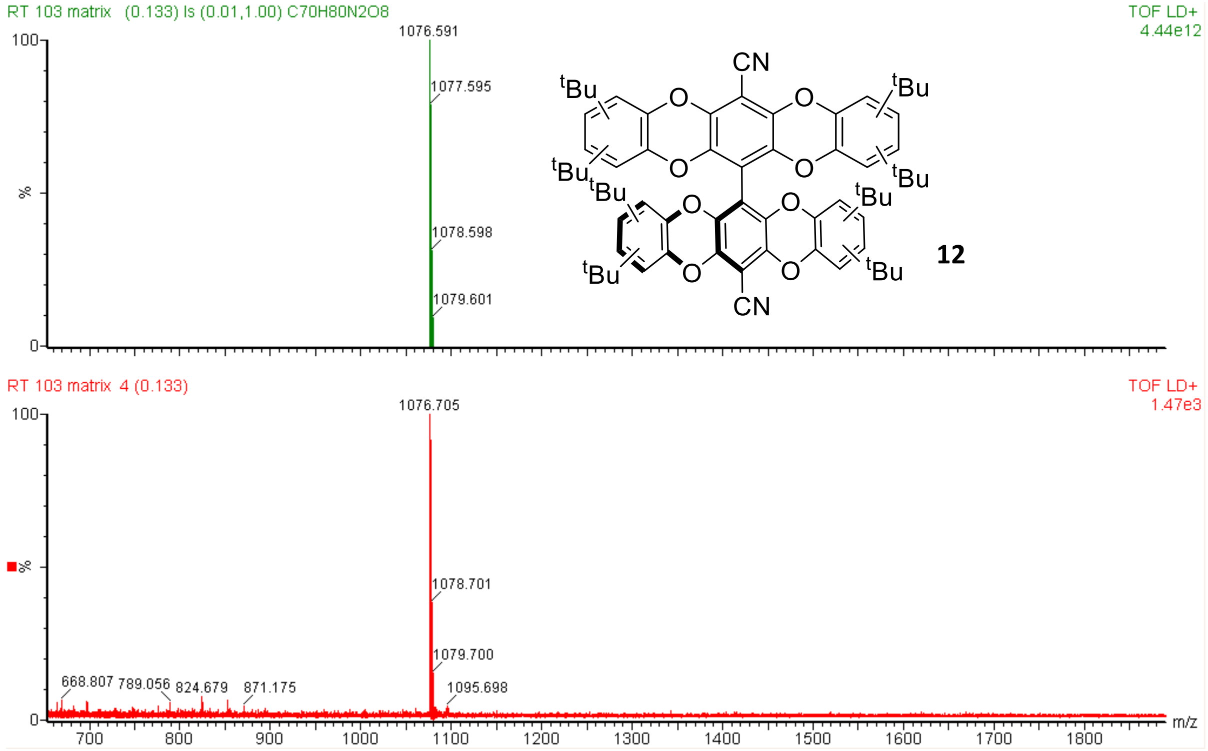
RT 103 matrix (0.133) Is (0.01,1.00) C70H80N2O8
TOF LD+
4.44e12
1076.591
1077.595
1078.598
1079.601
CN
tBu
O
12
RT 103 matrix 4 (0.133)
TOF LD+
1.47e3
1076.705
1078.701
1079.700
1095.698
668.807
789.056
824.679
871.175
%
m/z
700
800
900
1000
1100
1200
1300
1400
1500
1600
1700
1800

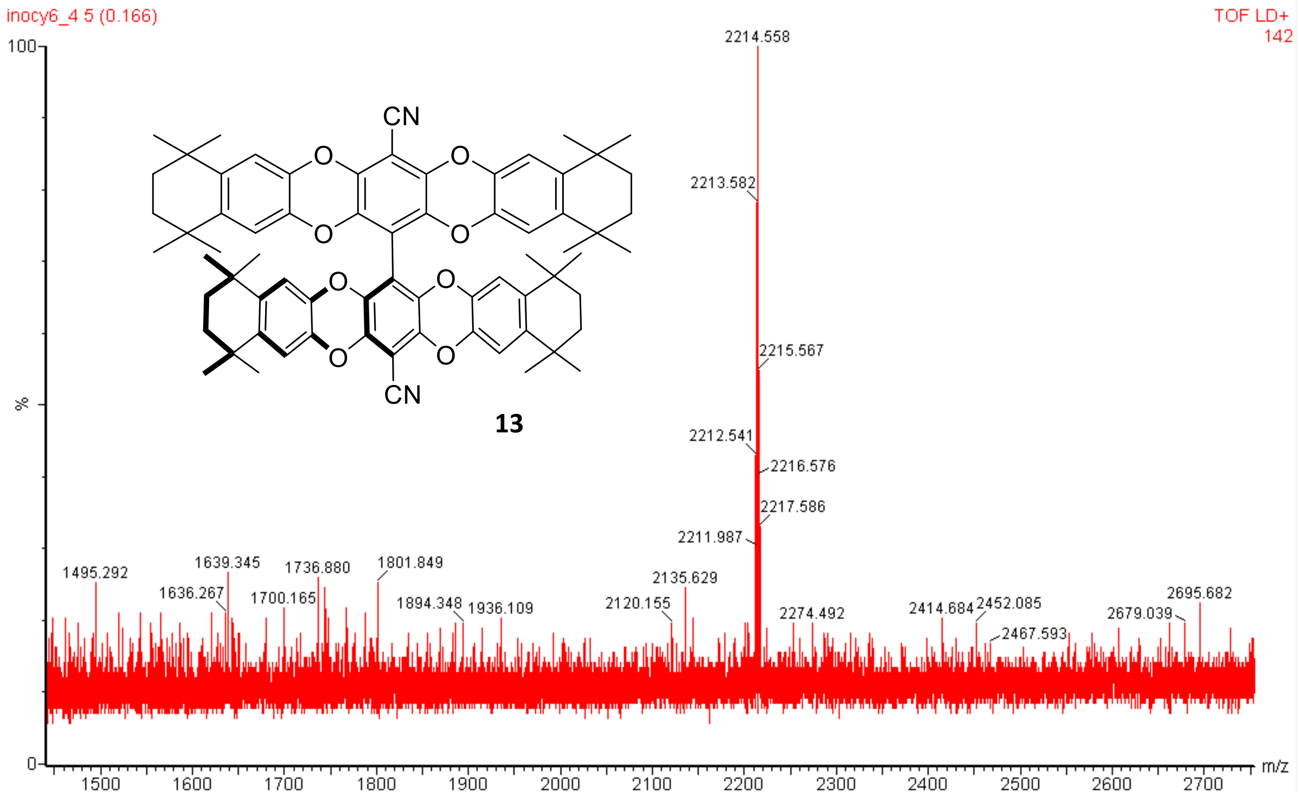
inocy6_4 5 (0.166)
TOF LD+
142
2214.558
2213.582
2215.567
2212.541
2216.576
2217.586
2211.987
2135.629
2120.155
2274.492
2414.684
2452.085
2467.593
2679.039
2695.682
1495.292
1639.345
1636.267
1736.880
1700.165
1801.849
1894.348
1936.109
CN
O
13
%
m/z
1500
1600
1700
1800
1900
2000
2100
2200
2300
2400
2500
2600
2700

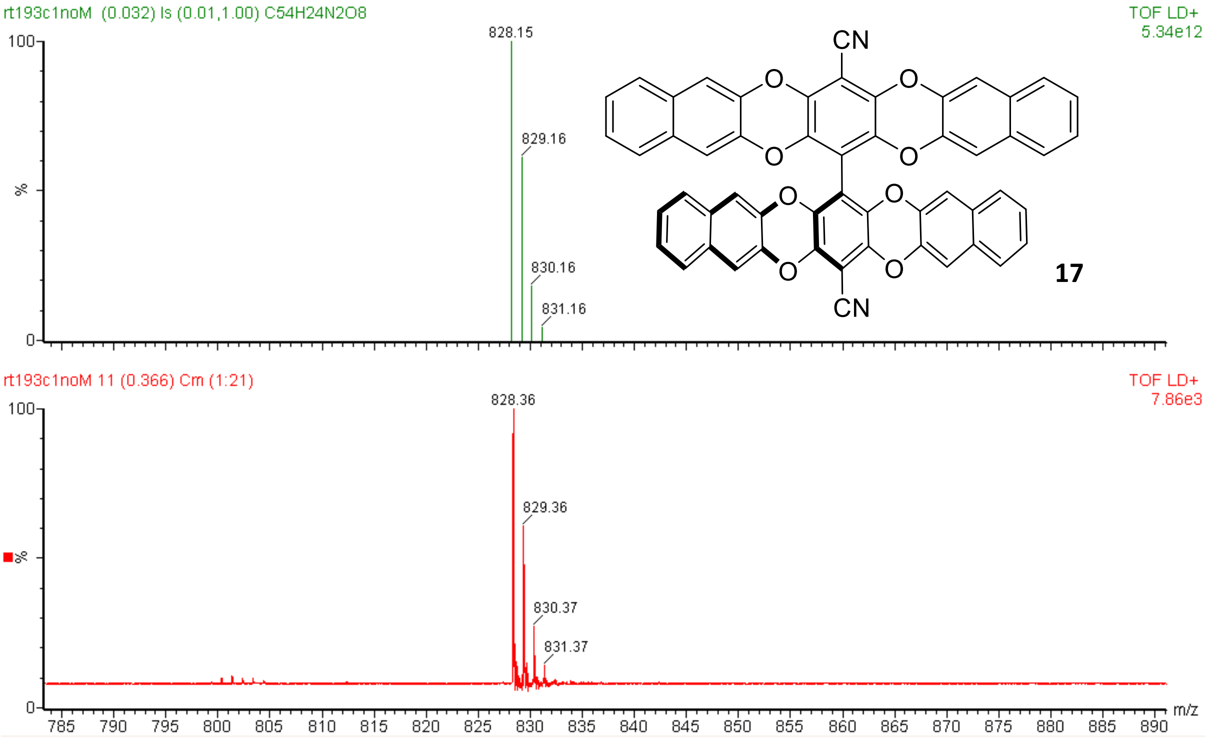

rt193c1noM (0.032) Is (0.01,1.00) C54H24N2O8
TOF LD+
5.34e12
828.15
829.16
830.16
831.16
CN
O
17
rt193c1noM 11 (0.366) Cm (1:21)
TOF LD+
7.86e3
828.36
829.36
830.37
831.37
m/z

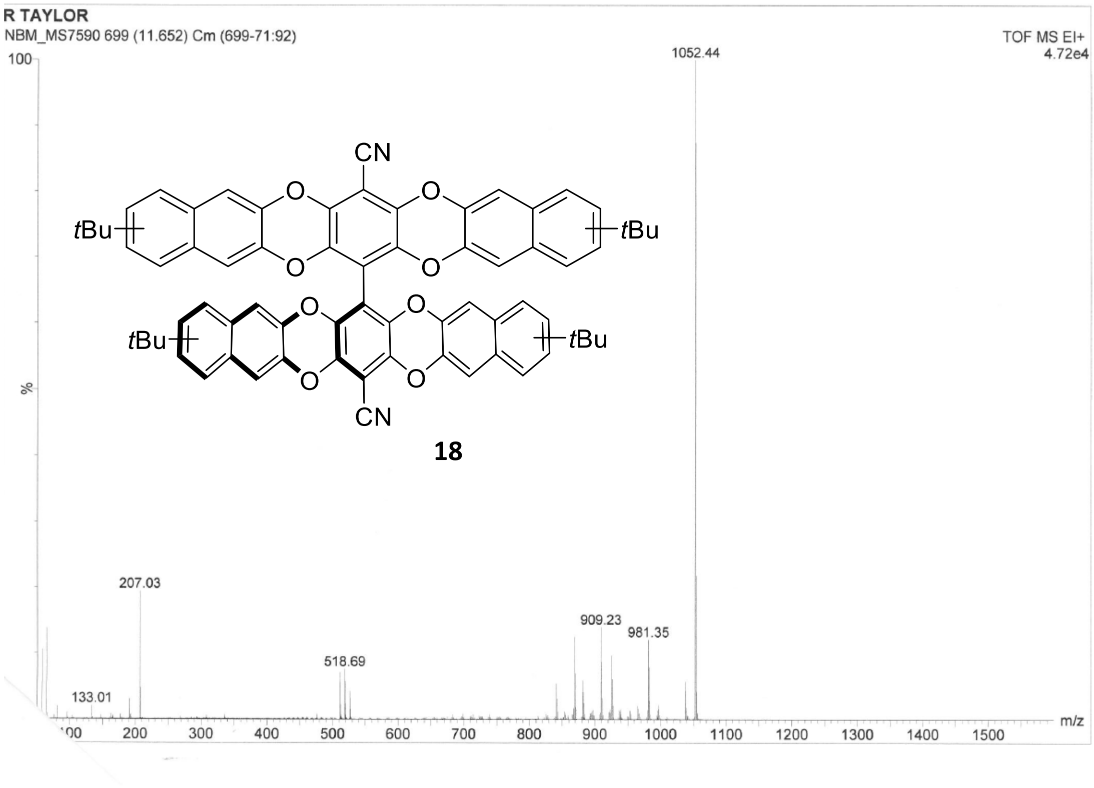

R TAYLOR
NBM_MS7590 699 (11.652) Cm (699-71:92)
TOF MS EI+
4.72e4
1052.44
CN
O
tBu
18
207.03
133.01
518.69
909.23
981.35
m/z

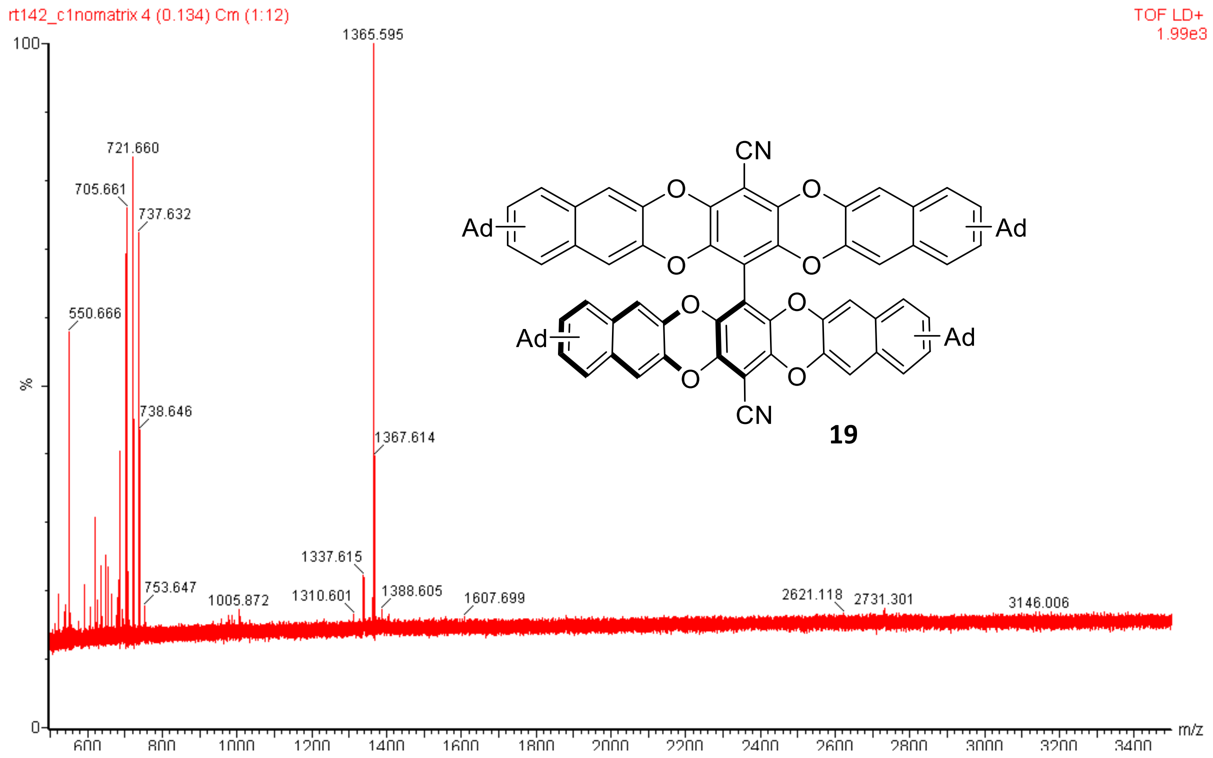

rt142_c1nomatrix 4 (0.134) Cm (1:12)
TOF LD+
1.99e3
1365.595
721.660
705.661
737.632
550.666
738.646
1367.614
1337.615
753.647
1005.872
1310.601
1388.605
1607.699
2621.118
2731.301
3146.006
%
m/z
CN
Ad
O
19

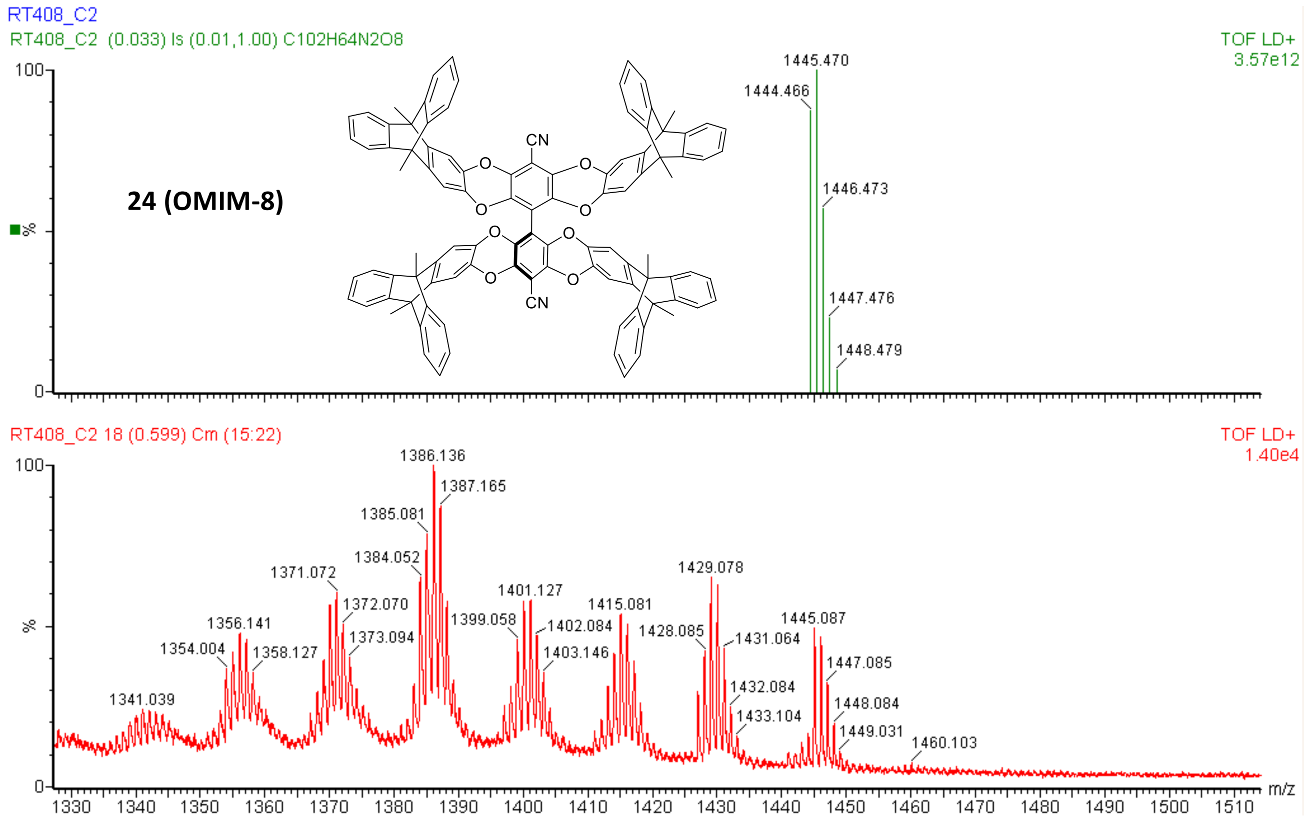

RT408_C2
RT408_C2 (0.033) Is (0.01,1.00) C102H64N2O8
TOF LD+
3.57e12
1445.470
1444.466
1446.473
1447.476
1448.479
24 (OMIM-8)
CN
O
RT408_C2 18 (0.599) Cm (15:22)
TOF LD+
1.40e4
1386.136
1387.165
1385.081
1384.052
1371.072
1372.070
1373.094
1356.141
1354.004
1358.127
1341.039
1401.127
1399.058
1402.084
1403.146
1415.081
1429.078
1428.085
1431.064
1432.084
1433.104
1445.087
1447.085
1448.084
1449.031
1460.103
%
m/z

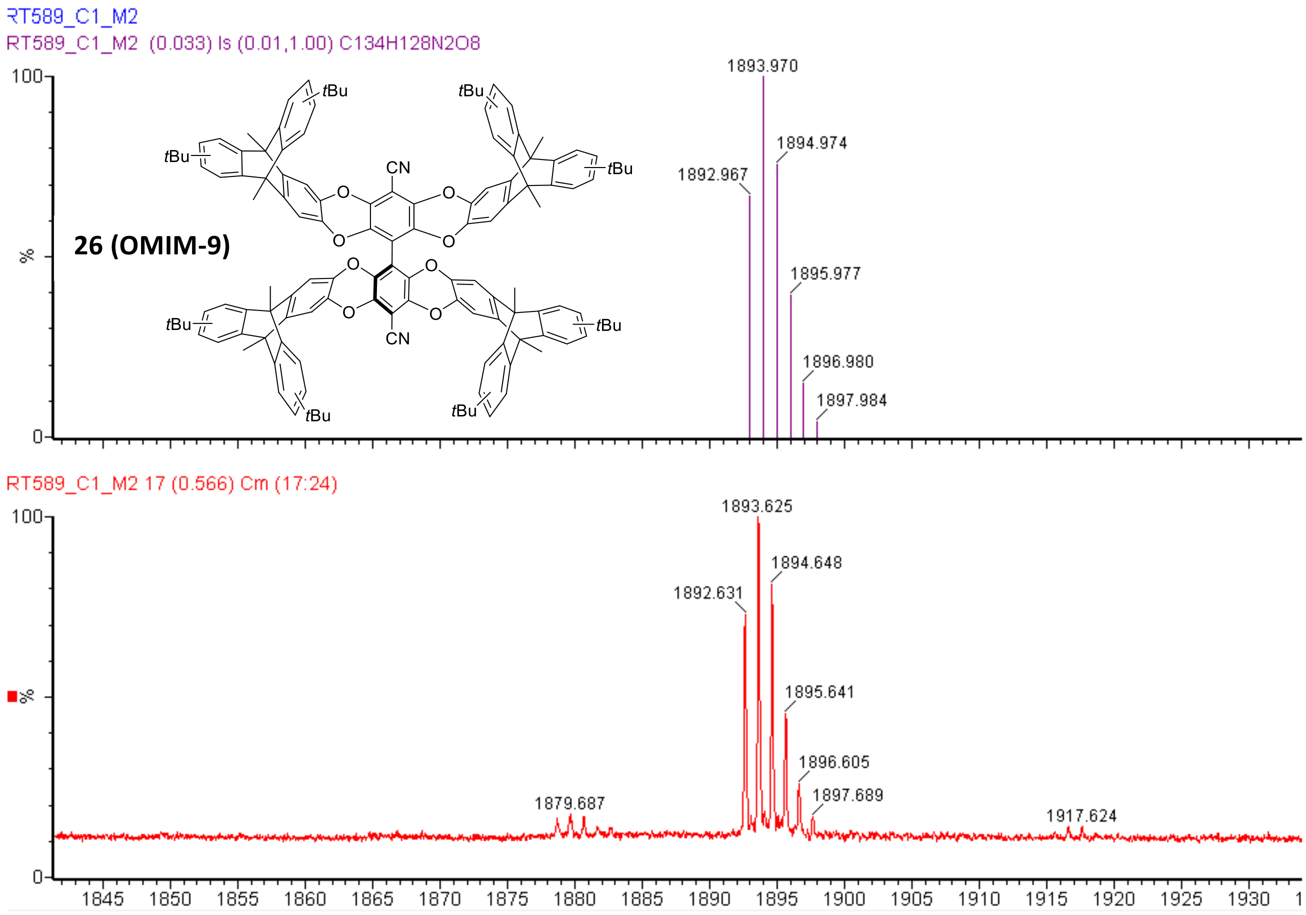
RT589_C1_M2
RT589_C1_M2 (0.033) Is (0.01,1.00) C134H128N2O8
26 (OMIM-9)
1893.970
1894.974
1892.967
1895.977
1896.980
1897.984
RT589_C1_M2 17 (0.566) Cm (17:24)
1893.625
1894.648
1892.631
1895.641
1896.605
1897.689
1879.687
1917.624
%

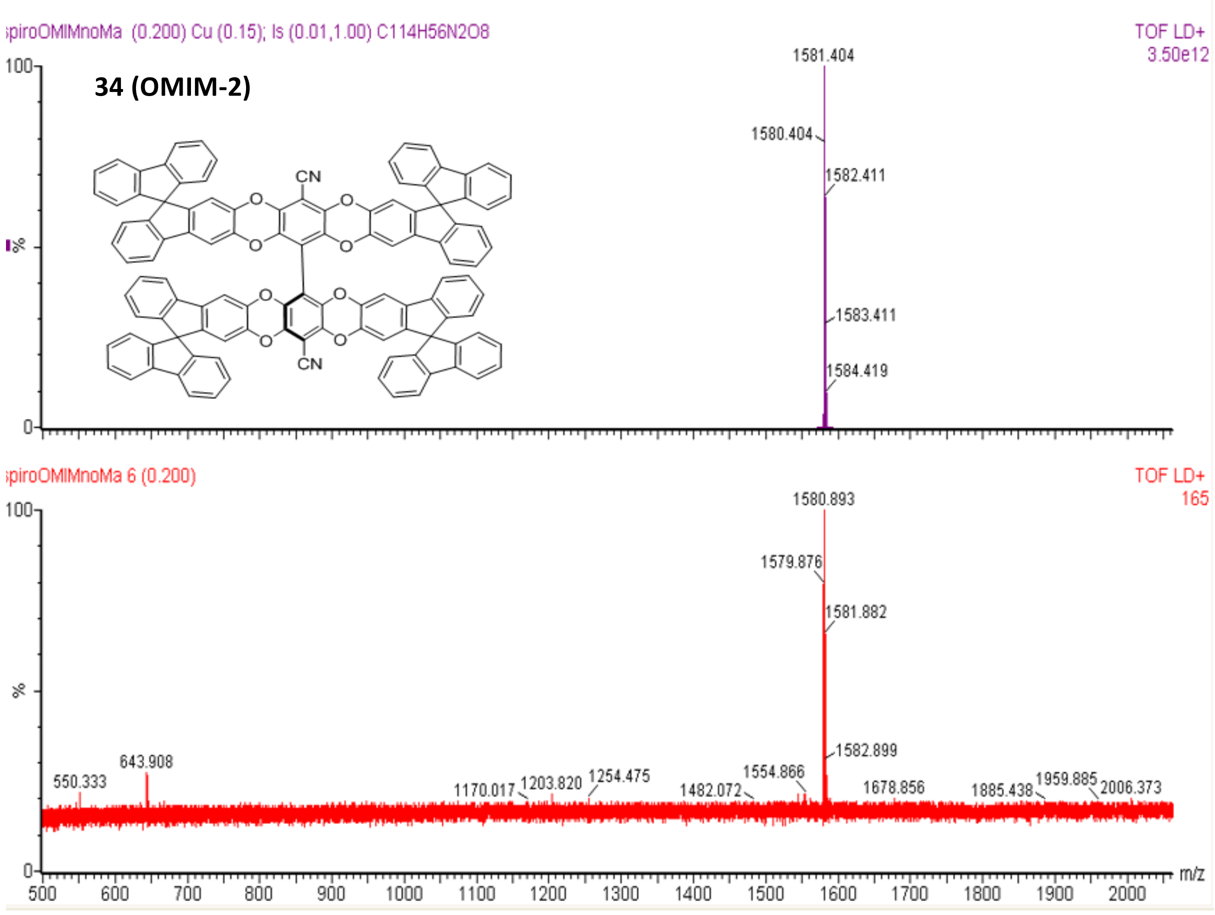
spiroOMIMnoMa (0.200) Cu (0.15); Is (0.01,1.00) C114H56N2O8
TOF LD+
3.50e12
34 (OMIM-2)
1581.404
1580.404
1582.411
1583.411
1584.419
spiroOMIMnoMa 6 (0.200)
TOF LD+
165
1580.893
1579.876
1581.882
1582.899
550.333
643.908
1170.017
1203.820
1254.475
1482.072
1554.866
1678.856
1885.438
1959.885
2006.373
m/z

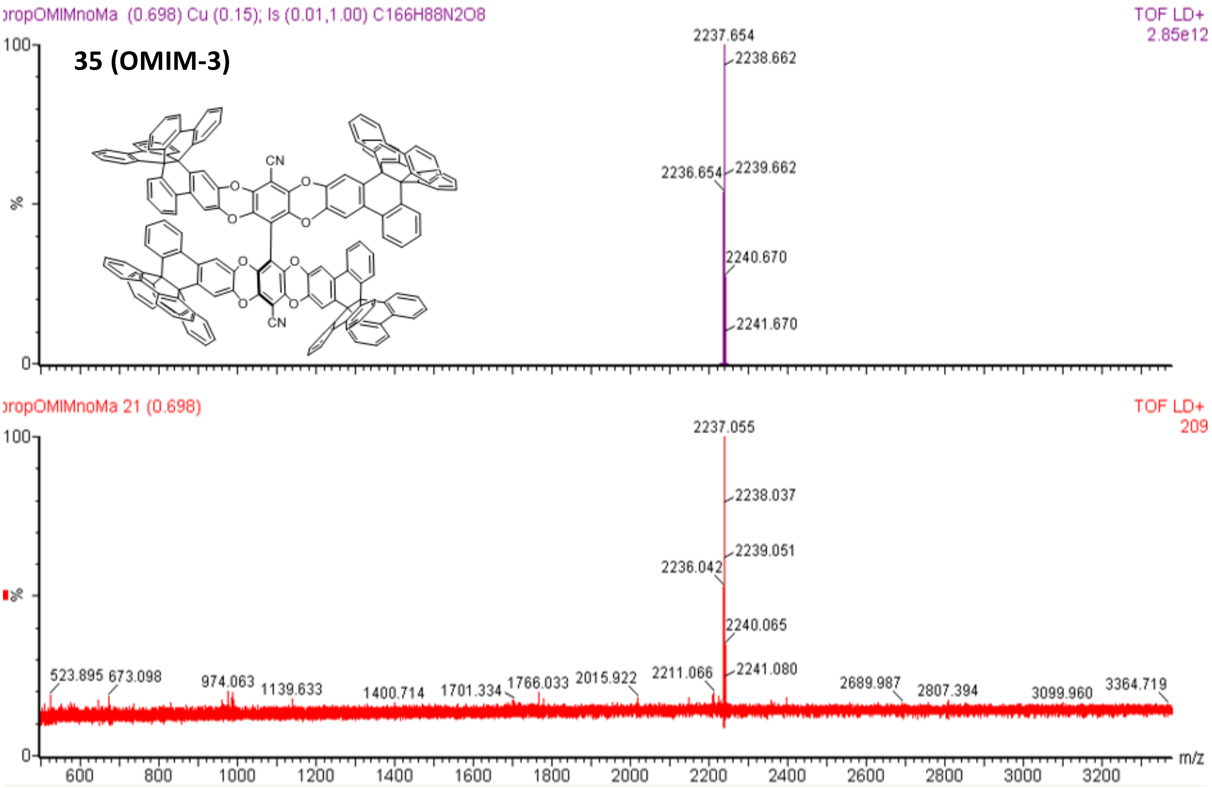
propOMIMnoMa (0.698) Cu (0.15); Is (0.01,1.00) C166H88N2O8
TOF LD+
2.85e12
35 (OMIM-3)
CN
CN
2237.654
2238.662
2236.654
2239.662
2240.670
2241.670
propOMIMnoMa 21 (0.698)
TOF LD+
209
2237.055
2238.037
2236.042
2239.051
2240.065
2241.080
523.895
673.098
974.063
1139.633
1400.714
1701.334
1766.033
2015.922
2211.066
2689.987
2807.394
3099.960
3364.719
m/z

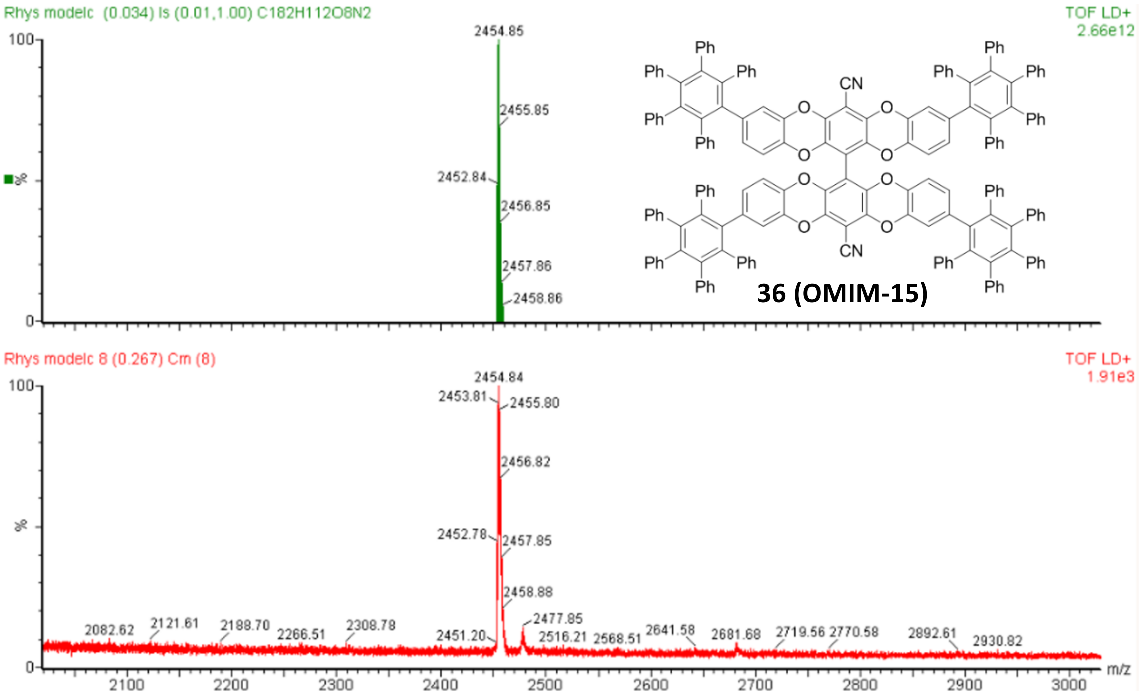
Rhys modelc (0.034) Is (0.01,1.00) C182H112O8N2
TOF LD+
2.66e12
2454.85
2455.85
2452.84
2456.85
2457.86
2458.86
36 (OMIM-15)
Ph
CN
Rhys modelc 8 (0.267) Cm (8)
TOF LD+
1.91e3
2454.84
2453.81
2455.80
2456.82
2452.78
2457.85
2458.88
2477.85
2082.62
2121.61
2188.70
2266.51
2308.78
2451.20
2516.21
2568.51
2641.58
2681.68
2719.56
2770.58
2892.61
2930.82
m/z

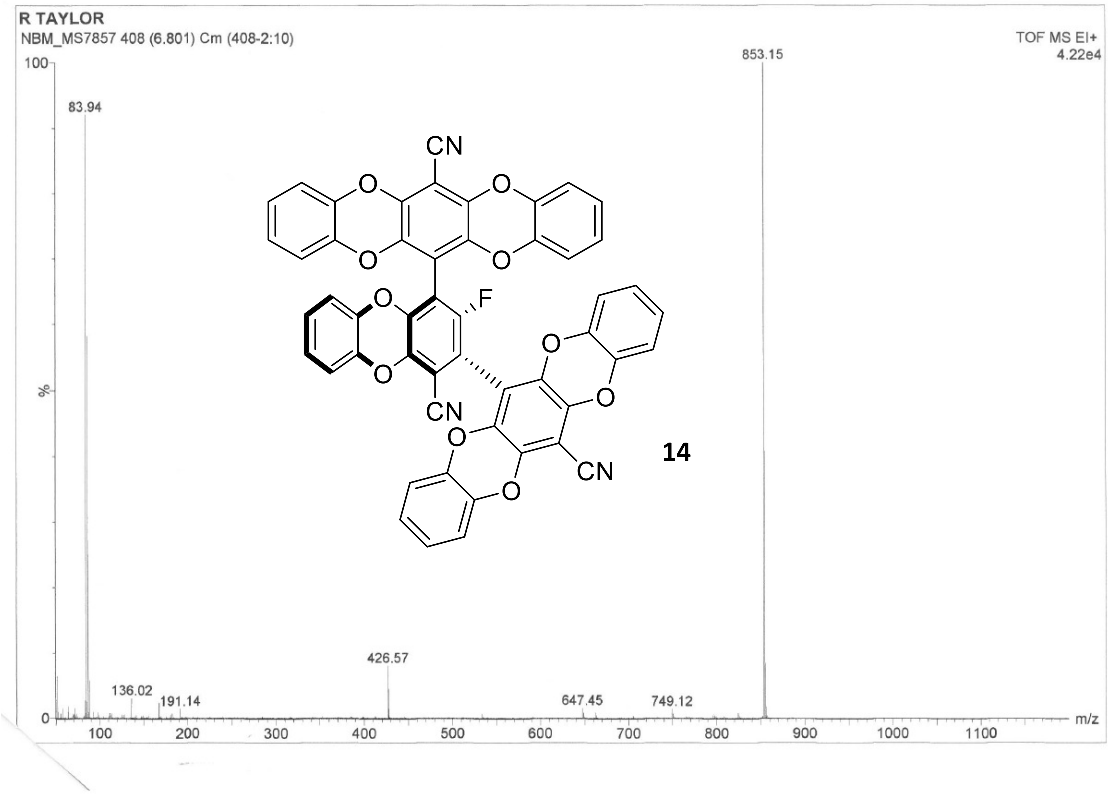
R TAYLOR
NBM_MS7857 408 (6.801) Cm (408-2:10)
TOF MS EI+
4.22e4
853.15
83.94
136.02
191.14
426.57
647.45
749.12
CN
O
F
14
m/z

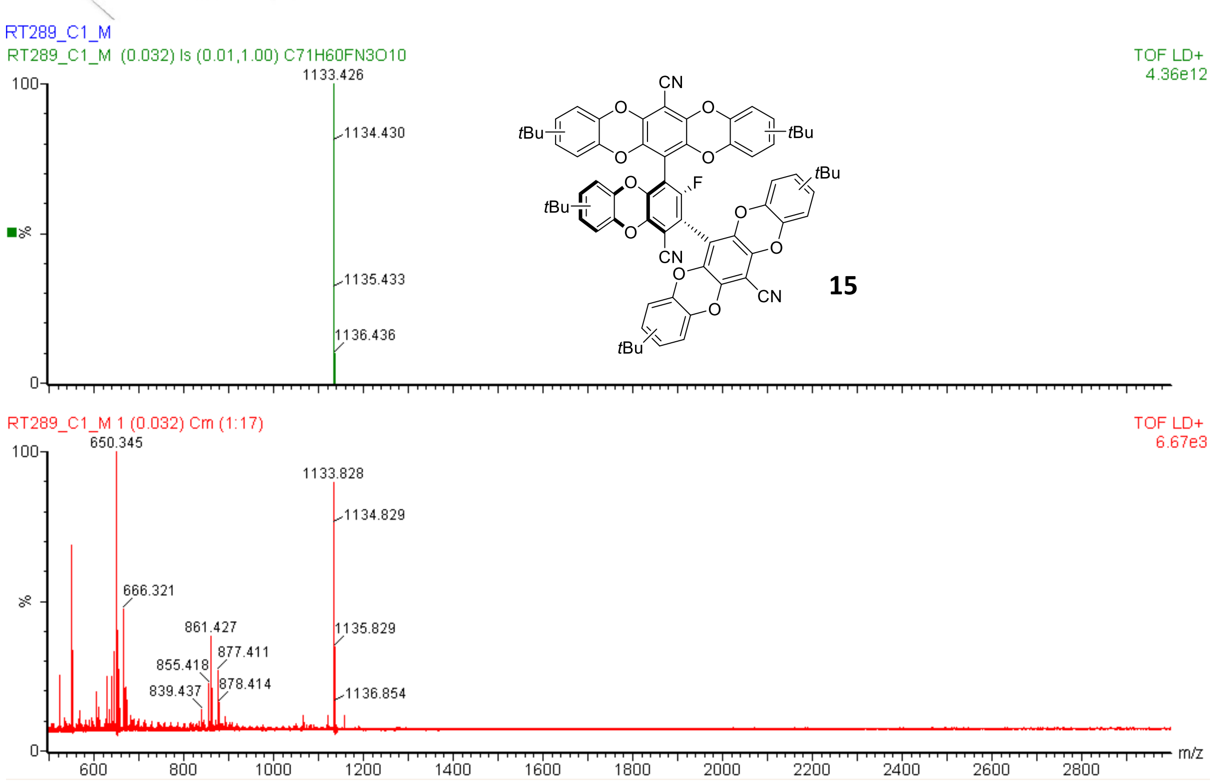
RT289_C1_M
RT289_C1_M (0.032) Is (0.01,1.00) C71H60FN3O10
TOF LD+
4.36e12
1133.426
1134.430
1135.433
1136.436
tBu
CN
F
15
RT289_C1_M 1 (0.032) Cm (1:17)
TOF LD+
6.67e3
650.345
666.321
861.427
855.418
877.411
839.437
878.414
1133.828
1134.829
1135.829
1136.854
m/z

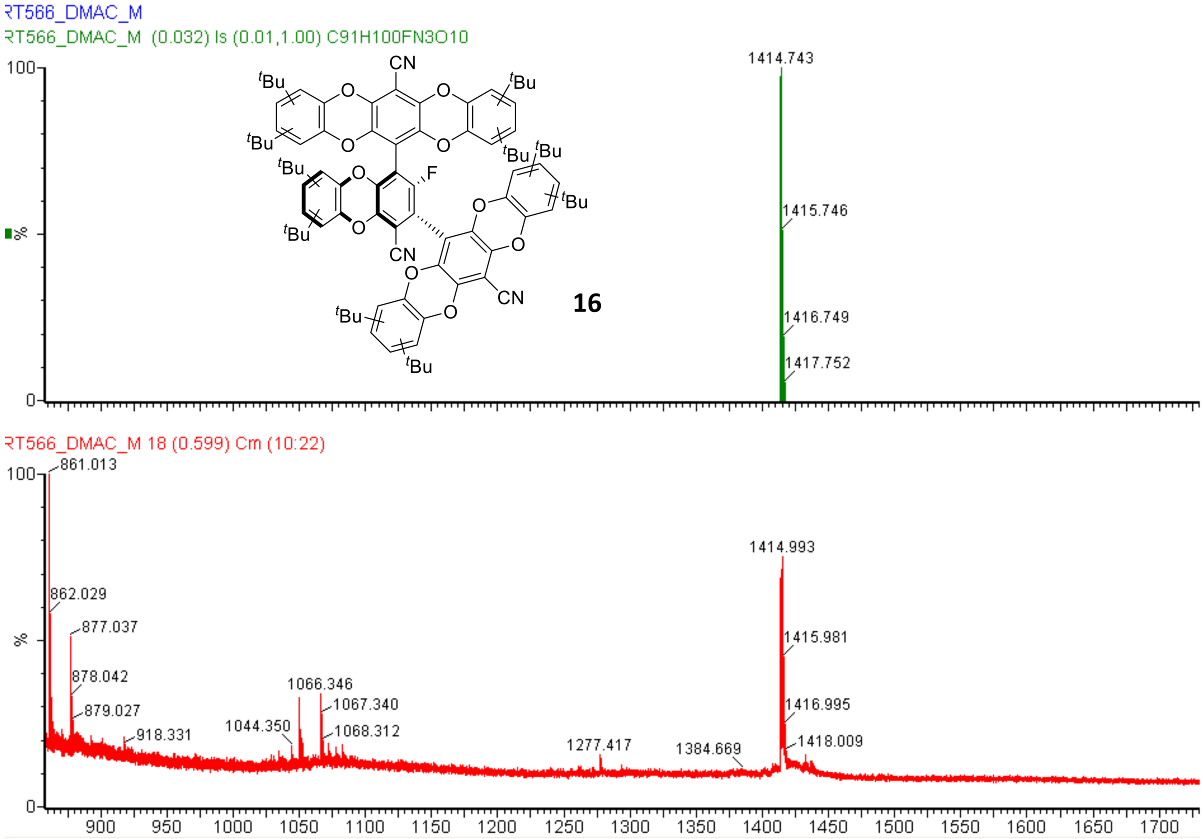
RT566_DMAC_M
RT566_DMAC_M (0.032) Is (0.01,1.00) C91H100FN3O10
1414.743
1415.746
1416.749
1417.752
16
RT566_DMAC_M 18 (0.599) Cm (10:22)
861.013
862.029
877.037
878.042
879.027
918.331
1044.350
1066.346
1067.340
1068.312
1277.417
1384.669
1414.993
1415.981
1416.995
1418.009

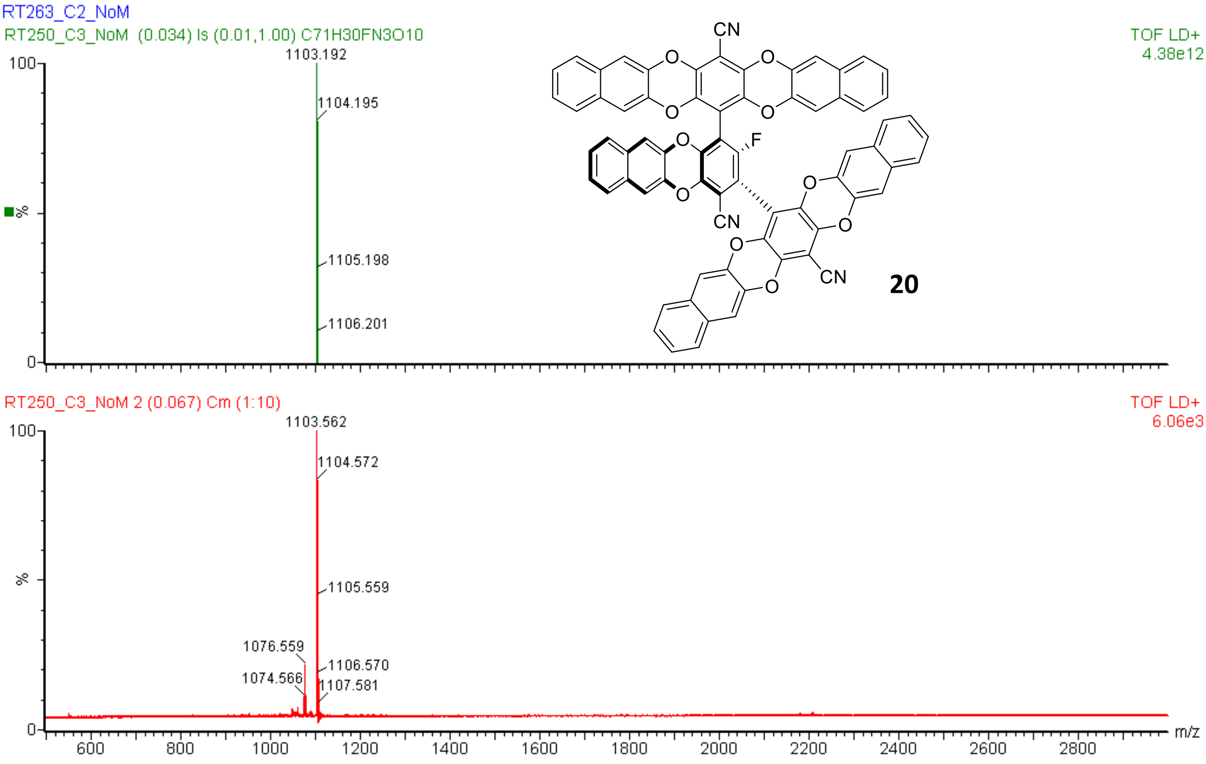
RT263_C2_NoM
RT250_C3_NoM (0.034) Is (0.01,1.00) C71H30FN3O10
TOF LD+
4.38e12
1103.192
1104.195
1105.198
1106.201
20
RT250_C3_NoM 2 (0.067) Cm (1:10)
TOF LD+
6.06e3
1103.562
1104.572
1105.559
1076.559
1074.566
1106.570
1107.581
m/z

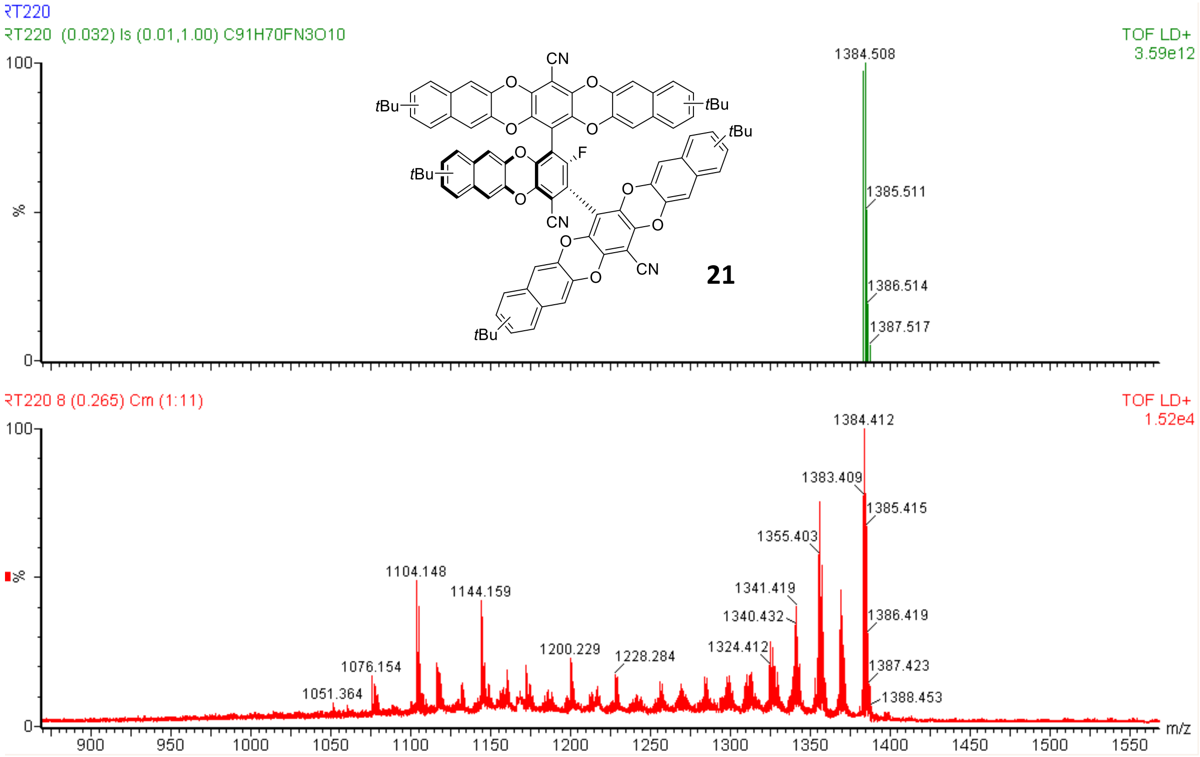
RT220
RT220 (0.032) Is (0.01,1.00) C91H70FN3O10
TOF LD+
3.59e12
1384.508
1385.511
1386.514
1387.517
CN
O
tBu
F
21
RT220 8 (0.265) Cm (1:11)
TOF LD+
1.52e4
1384.412
1383.409
1385.415
1355.403
1341.419
1340.432
1386.419
1324.412
1387.423
1388.453
1104.148
1144.159
1200.229
1228.284
1076.154
1051.364
m/z
%

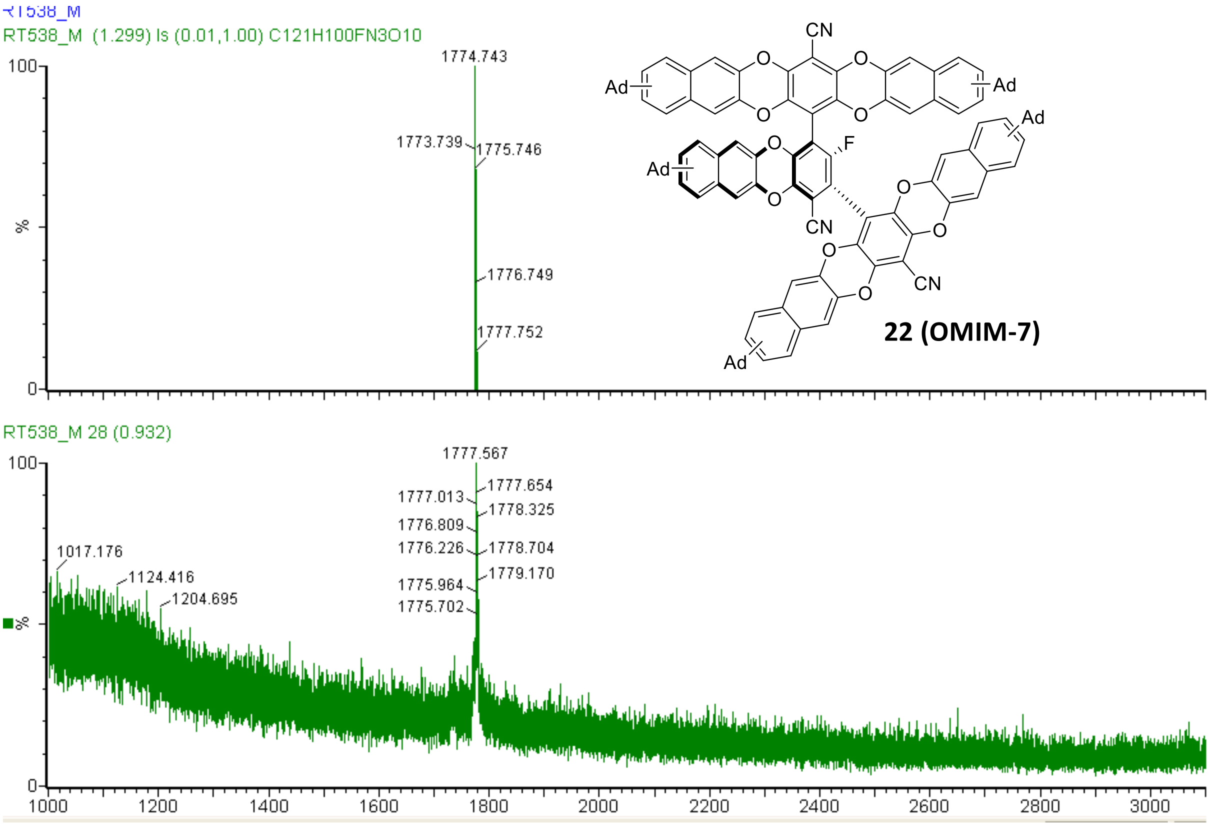
RT538_M
RT538_M (1.299) Is (0.01,1.00) C121H100FN3O10
1774.743
1773.739
1775.746
1776.749
1777.752
CN
O
Ad
F
22 (OMIM-7)
RT538_M 28 (0.932)
1777.567
1777.654
1777.013
1778.325
1776.809
1776.226
1778.704
1779.170
1775.964
1775.702
1017.176
1124.416
1204.695
%

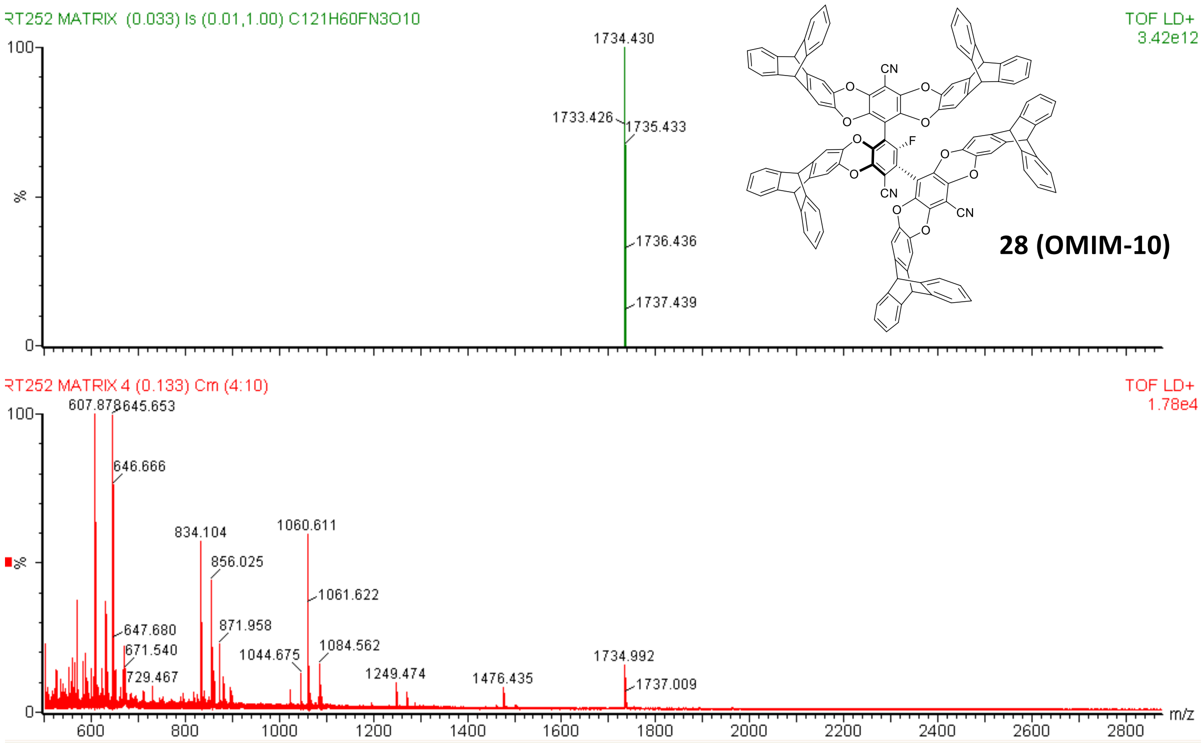
RT252 MATRIX (0.033) Is (0.01,1.00) C121H60FN3O10
TOF LD+
3.42e12
1734.430
1733.426
1735.433
1736.436
1737.439
CN
O
F
CN
28 (OMIM-10)
RT252 MATRIX 4 (0.133) Cm (4:10)
TOF LD+
1.78e4
607.878
645.653
646.666
834.104
1060.611
856.025
1061.622
647.680
871.958
671.540
1044.675
1084.562
729.467
1249.474
1476.435
1734.992
1737.009
m/z
%
600
800
1000
1200
1400
1600
1800
2000
2200
2400
2600
2800

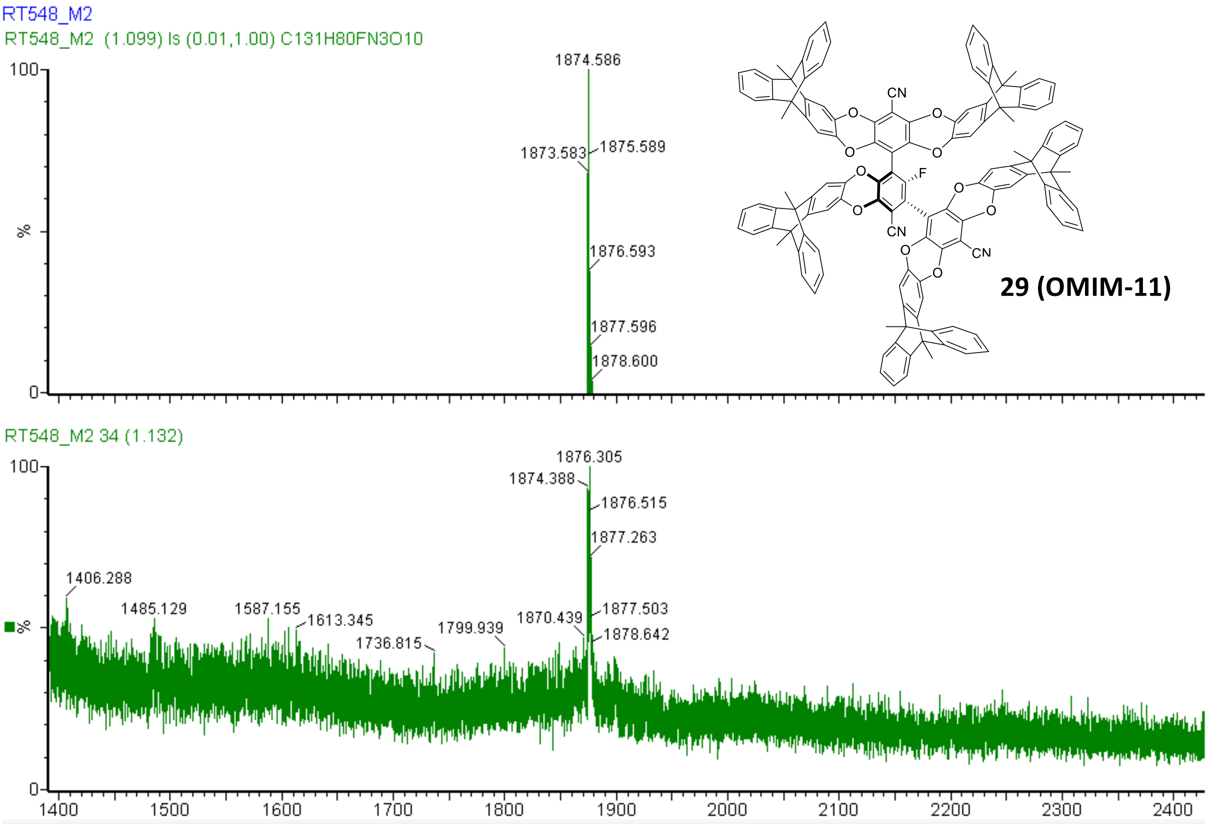
RT548_M2
RT548_M2 (1.099) Is (0.01,1.00) C131H80FN3O10
1874.586
1873.583
1875.589
1876.593
1877.596
1878.600
CN
O
F
29 (OMIM-11)
RT548_M2 34 (1.132)
1876.305
1874.388
1876.515
1877.263
1406.288
1485.129
1587.155
1613.345
1736.815
1799.939
1870.439
1877.503
1878.642
%
1400
1500
1600
1700
1800
1900
2000
2100
2200
2300
2400

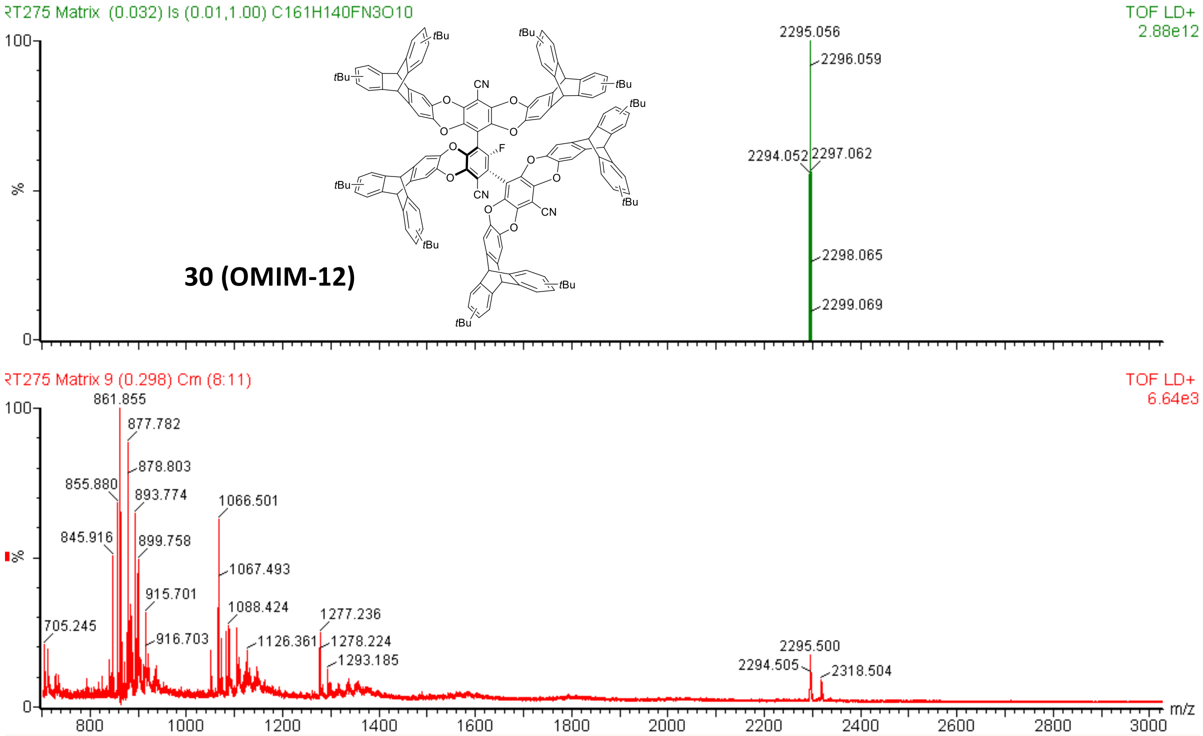

30 (OMIM-12)

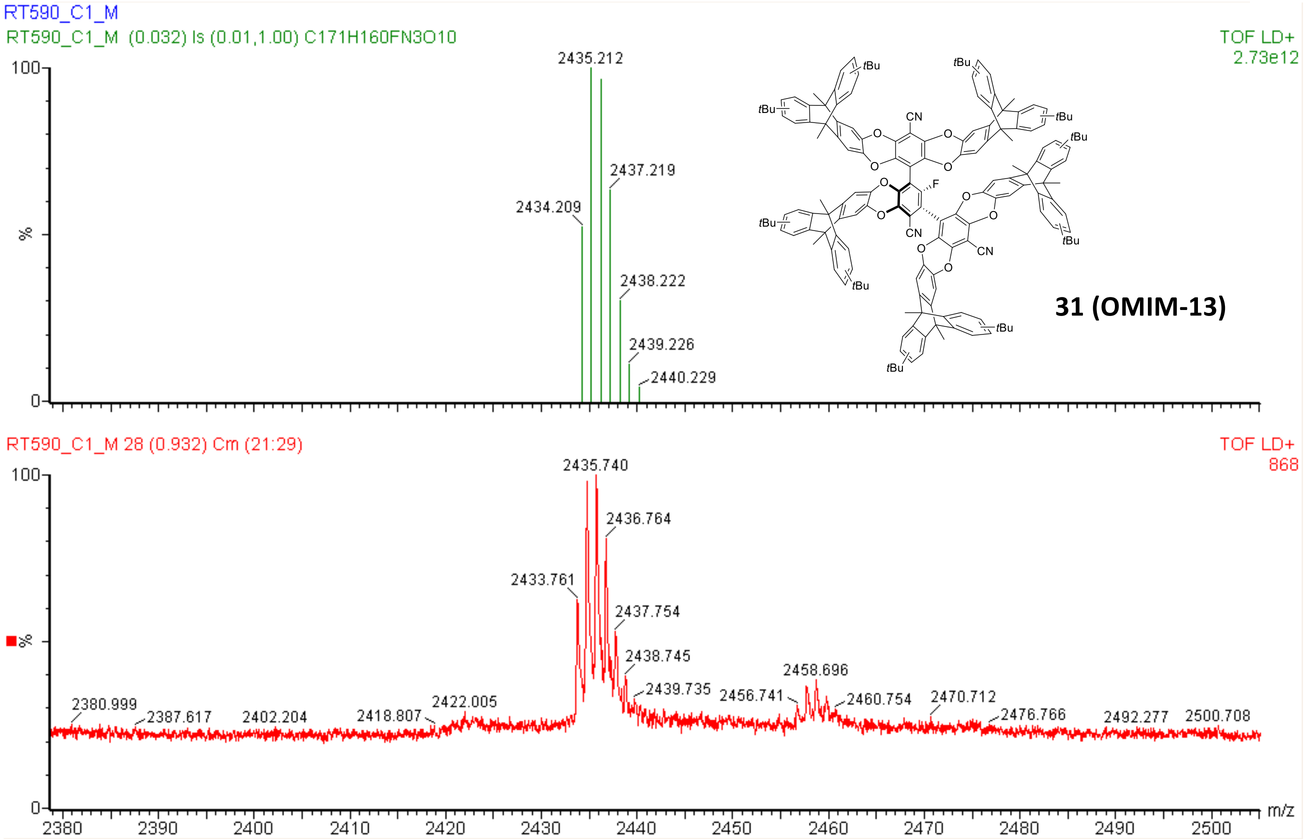

31 (OMIM-13)

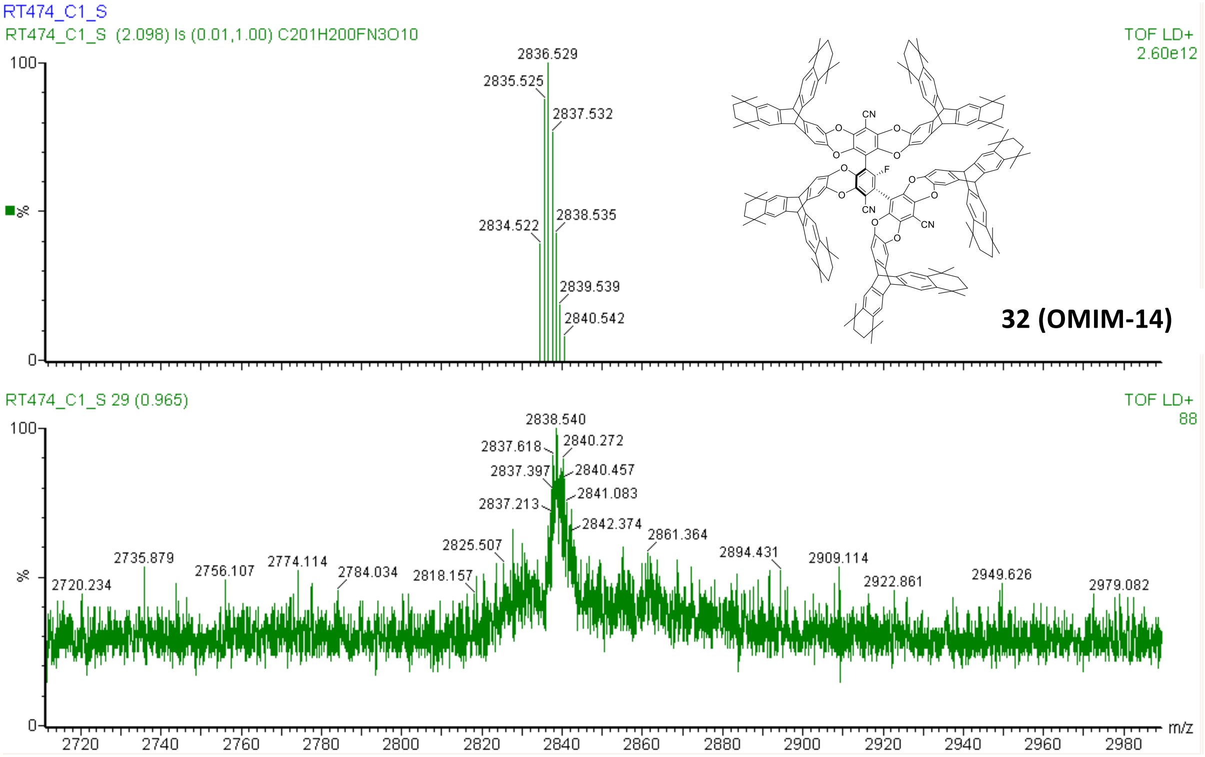
RT474_C1_S
RT474_C1_S (2.098) Is (0.01,1.00) C201H200FN3O10
TOF LD+
2.60e12
2836.529
2835.525
2837.532
2834.522
2838.535
2839.539
2840.542
32 (OMIM-14)
RT474_C1_S 29 (0.965)
TOF LD+
88
2838.540
2837.618
2840.272
2837.397
2840.457
2841.083
2837.213
2842.374
2861.364
2825.507
2894.431
2735.879
2909.114
2756.107
2774.114
2784.034
2818.157
2922.861
2949.626
2720.234
2979.082
m/z

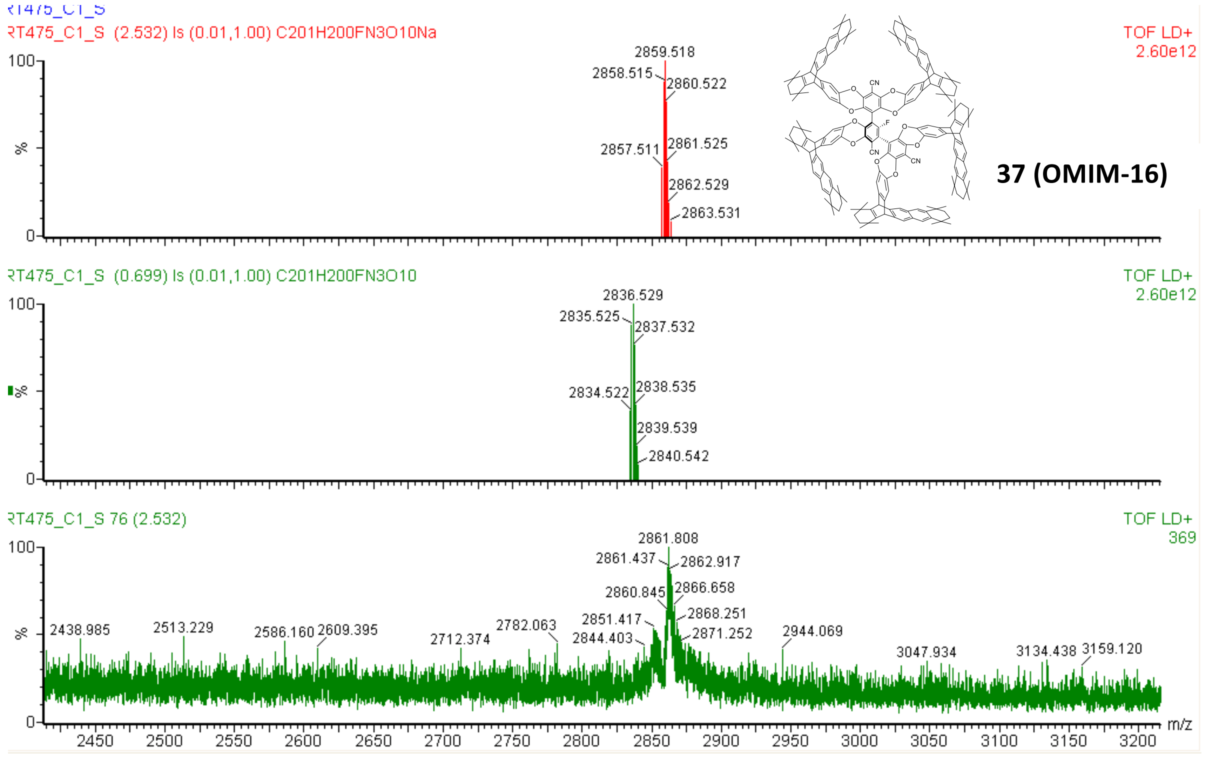
RT475_C1_S (2.532) Is (0.01,1.00) C201H200FN3O10Na
TOF LD+
2.60e12
2859.518
2858.515
2860.522
2857.511
2861.525
2862.529
2863.531
37 (OMIM-16)
RT475_C1_S (0.699) Is (0.01,1.00) C201H200FN3O10
TOF LD+
2.60e12
2836.529
2835.525
2837.532
2834.522
2838.535
2839.539
2840.542
RT475_C1_S 76 (2.532)
TOF LD+
369
2861.808
2861.437
2862.917
2860.845
2866.658
2851.417
2868.251
2844.403
2871.252
2438.985
2513.229
2586.160
2609.395
2712.374
2782.063
2944.069
3047.934
3134.438
3159.120
m/z

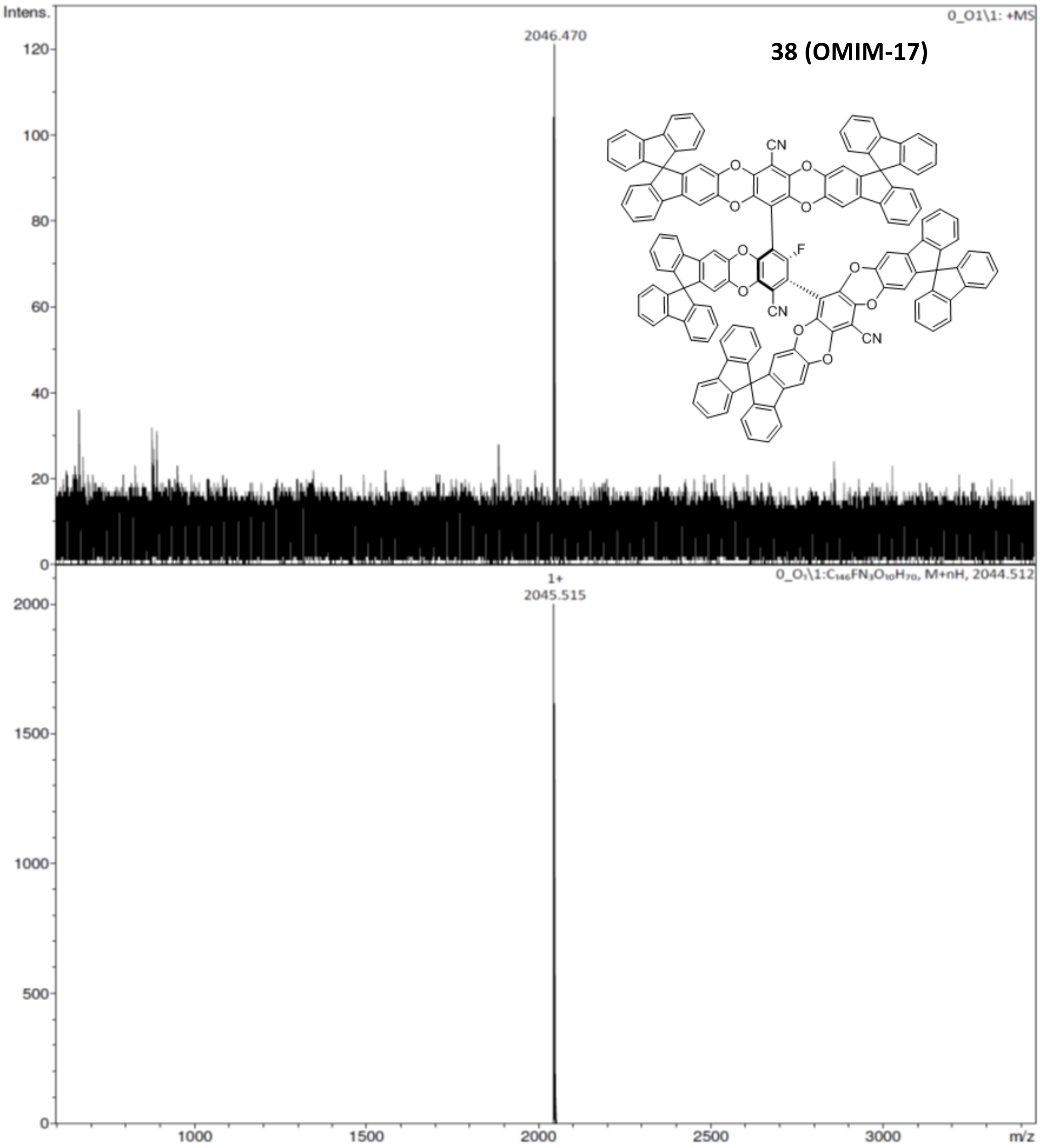
Intens.
0_O1\1: +MS
2046.470
38 (OMIM-17)
CN
F
0_O1\1:C146FN3O10H70, M+nH, 2044.512
1+
2045.515
m/z

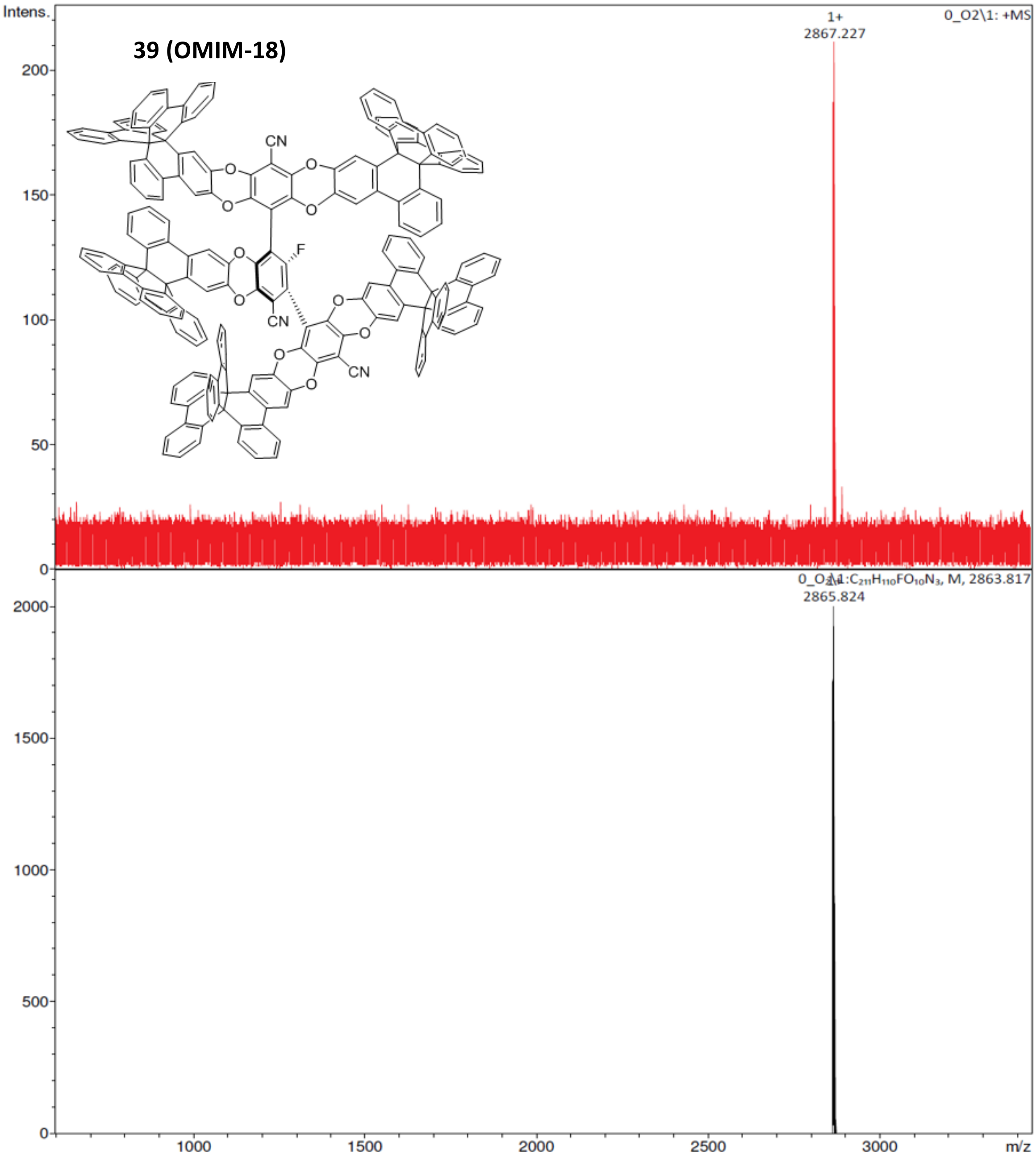
Intens.
39 (OMIM-18)
CN
O
F
CN
CN
1+
2867.227
0_O2\1: +MS
0_O2\1:C211H110FO10N3, M, 2863.817
2865.824
m/z

References

[1] H. E. Gottlieb, V. Kotlyar, A. Nudelman, *J. Org. Chem* **1997**, *62*, 7512-7515.
[2] J. P. Gunning, J. W. Levell, M. F. Wyatt, P. L. Burn, J. Robertson, I. D. W. Samuel, *Polym. Chem.* **2010**, *1*, 730-738.
[3] T. Kaneko*, Vol. JP 2006273766*, Tosoh Finechem Corporation, Japan, **2005**.
[4] A. Stepakov, A. Molchanov, R. Kostikov, *Russ. J. Org. Chem.* **2007**, *43*, 538-543.
[5] X.-X. Peng, H.-Y. Lu, T. Han, C.-F. Chen, *Org. Lett.* **2007**, *9*, 895-898.
[6] R. G. D. Taylor, M. Carta, C. G. Bezzu, J. Walker, K. J. Msayib, B. M. Kariuki, N. B. McKeown, *Org. Lett.* **2014**, *16*, 1848-1851.
[7] J. M. Birchall, R. N. Haszeldine, M. E. Jones, *J. Chem. Soc. C* **1971**, *0*, 1343-1348.
[8] A. Alberola, R. Less, F. Palacio, C. Pask, J. Rawson, *Molecules* **2004**, *9*, 771-781.
[9] V. J. Chebny, T. S. Navale, R. Shukla, S. V. Lindeman, R. Rathore, *Org. Lett.* **2009**, *11*, 2253-2256.
[10] H. A. Bruson, J. W. Kroeger, *J. Am. Chem. Soc.* **1940**, *62*, 36-44.
[11] L. E. Marinina, V. I. Alekseeva, L. P. Sawina, E. A. Luk'yanets, *Chem. Heterocycl. Compd.* **1988**, *24*, 220-225.
[12] B. H. Klanderman, T. R. Criswell, *J. Org. Chem* **1969**, *34*, 3426-3430.
[13] E. Yagodkin, C. J. Douglas, *Tetrahedron Lett.* **2010**, *51*, 3037-3040.
[14] P. P. Fu, R. G. Harvey, *J. Org. Chem* **1977**, *42*, 2407-2410.
[15] H. Hart, K. Harada, C. J. F. Du, *J. Org. Chem* **1985**, *50*, 3104-3110.
[16] C. G. Bezzu, M. Carta, A. Tonkins, J. C. Jansen, P. Bernardo, F. Bazzarelli, N. B. McKeown, *Adv. Mater.* **2012**, *24*, 5930-5933.
[17] M. Hoang, T. Gadosy, H. Ghazi, D.-F. Hou, A. C. Hopkinson, L. J. Johnston, E. Lee-Ruff, *J. Org. Chem.* **1998**, *63*, 7168-7171.
[18] M. L. N. Rao, D. N. Jadhav, P. Dasgupta, *Org. Lett.* **2010**, *12*, 2048-2051.
